# Nontarget Analysis of Polluted Surface Waters in Bangladesh Using Open Science Workflows

**DOI:** 10.1021/acs.est.2c08200

**Published:** 2023-04-21

**Authors:** Bénilde Bonnefille, Oskar Karlsson, May Britt Rian, Rubhana Raqib, Faruque Parvez, Stefano Papazian, M. Sirajul Islam, Jonathan W. Martin

**Affiliations:** † Department of Environmental Science, Exposure and Effects Unit, Science for Life Laboratory, Stockholm University, Stockholm 106 91, Sweden; ‡ Immunobiology, Nutrition and Toxicology Unit, Infectious Diseases Division, International Centre for Diarrhoeal Disease Research, Bangladesh (icddr,b), Dhaka 1212, Bangladesh; § Department of Environmental Health Sciences, Mailman School of Public Health, Columbia University, New York, New York 10032, United States; ∥ Laboratory of Food Safety and One Health, Laboratory Sciences and Services Division, International Centre for Diarrhoeal Disease Research, Bangladesh (icddr,b), Dhaka 1212, Bangladesh; @ National Facility for Exposomics, Metabolomics Platform, Science for Life Laboratory, Stockholm University, Solna 171 65, Sweden

**Keywords:** high-resolution mass spectrometry, nontarget analysis, electrospray ionization, atmospheric pressure chemical ionization, orbitrap, organic micropollutants, South Asia, water pollution

## Abstract

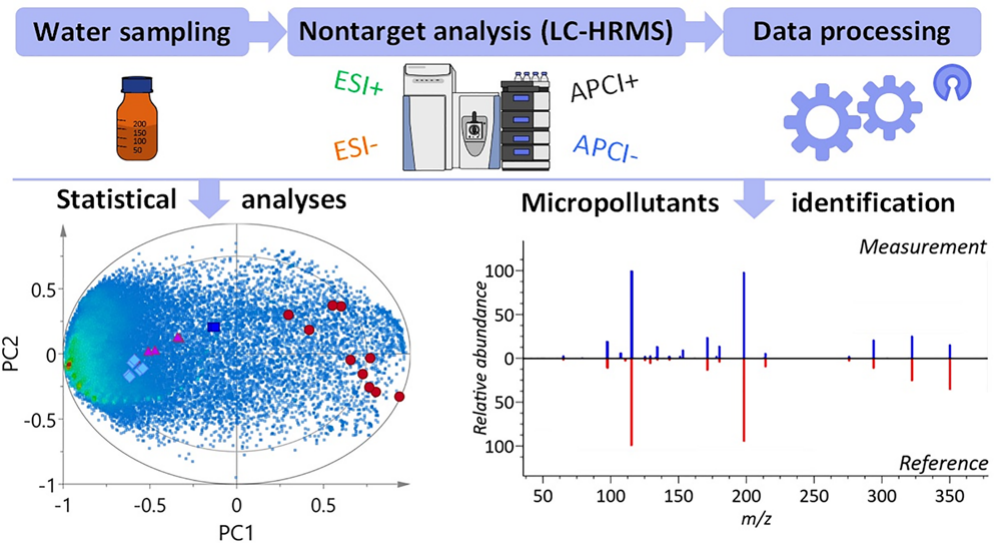

Nontarget mass spectrometry has great potential to reveal patterns of water contamination globally through community science, but few studies are conducted in low-income countries, nor with open-source workflows, and few datasets are FAIR (Findable, Accessible, Interoperable, Reusable). Water was collected from urban and rural rivers around Dhaka, Bangladesh, and analyzed by liquid chromatography high-resolution mass spectrometry in four ionization modes (electrospray ionization ±, atmospheric pressure chemical ionization ±) with data-independent MS2 acquisition. The acquisition strategy was complementary: 19,427 and 7365 features were unique to ESI and APCI, respectively. The complexity of water pollution was revealed by >26,000 unique molecular features resolved by MS-DIAL, among which >20,000 correlated with urban sources in Dhaka. A major wastewater treatment plant was not a dominant pollution source, consistent with major contributions from uncontrolled urban drainage, a result that encourages development of further wastewater infrastructures. Matching of deconvoluted MS2 spectra to public libraries resulted in 62 confident annotations (*i.e.*, Level 1-2a) and allowed semiquantification of 42 analytes including pharmaceuticals, pesticides, and personal care products. *In silico* structure prediction for the top 100 unknown molecular features associated with an urban source allowed 15 additional chemicals of anthropogenic origin to be annotated (*i.e.*, Level 3). The authentic MS2 spectra were uploaded to MassBank Europe, mass spectral data were openly shared on the MassIVE repository, a tool (*i.e.*, MASST) that could be used for community science environmental surveillance was demonstrated, and current limitations were discussed.

## Abstract 2

Nontarget LC-HRMS datasets of surface water from four ionization modes were combined in open science workflows, revealing great molecular complexity and multiclass contaminant discovery while also enabling community science.

## 1 Introduction

Surface waters have long been monitored for organic micropollutants, including in remote regions^1,2^ and urban and industrial areas,^3−9^ but most data are from high-income countries, with few studies in low- and middle-income countries where pollution is greatest.^5^ Clean water is a United Nations Sustainable Development Goal,^10^ and South Asian countries are among the most challenged due to high population densities, intense manufacturing and agriculture for global export,^11^ and underdeveloped water infrastructures. Despite continuing efforts to improve water sanitation in low-income countries, industrial water pollution still receives little international attention, and the total disease burden from chemical pollution is thought to be underestimated.^12,13^

Bangladesh is a lower-middle income country in South Asia with one of the highest population densities globally.^14^ With a relatively underdeveloped wastewater infrastructure^14,15^ and a high density of agriculture, aquaculture, and industry (e.g., textile, manufacturing, pharmaceutical), drinking water contamination by anthropogenic chemicals is a major issue.^5,15−17^ The few previous studies of surface water organic micropollutants in Bangladesh have mainly focused on selected pesticides and legacy polychlorinated biphenyls.^18−21^ A recent systematic review noted limited studies for emerging water pollutants in Bangladesh,^16^ although recent data are available for pharmaceuticals, personal care products, aquaculture chemicals, and per- and polyfluoroalkyl substances.^22−26^ A global monitoring study of targeted pharmaceuticals in surface waters recently reported that samples from Bangladesh contained high concentrations of 13 pharmaceuticals, including the highest observed levels of metronidazole, 300 times higher than the estimated safe levels for this single antimicrobial substance.^5^

Nontarget mass spectrometry analysis uses comprehensive data acquisition strategies without *a priori* knowledge of what compounds should be expected in a given sample.^27^ This approach usually relies on a liquid (LC) or gas chromatography separation with high resolution mass spectrometry (HRMS) to detect the diverse range of molecular features present; each unknown molecular feature is thus defined by a retention time and mass spectral information (e.g., molecular ion and fragments). Chemometric tools can be used to recognize patterns in the detected molecular features across samples, while spectral libraries and cheminformatic resources can be integrated for the high-throughput annotation of unknown features using open-source molecular discovery workflows.^28,29^ To our knowledge, unbiased nontarget analysis has not been previously applied to South Asian surface waters.

Suspect screening, a subcategory of nontarget analysis, is an approach using predefined lists (in-house or public, *e.g.*, NORMAN Suspect List Exchange^30^) to screen for specific compounds in samples based on mass-spectral properties (e.g., exact mass, isotopes).^27^ Suspect screening is often focused on certain chemical classes, such as pharmaceuticals or pesticides, and has usually been performed using electrospray ionization (ESI).^31^ One important study conducted on Bangladesh surface waters combined suspect screening and targeted analysis in ESI mode for the detection of pharmaceuticals and antibiotics.^23^ This revealed previously ignored antibiotics, antifungals, and their transformation products.^23^ Singh *et al.* compared ESI and atmospheric pressure chemical ionization (APCI), both in positive and negative modes, for the detection of 1264 substances and suggested that APCI can broaden the chemical space coverage, as it increased the number of detected compounds (*i.e*., + 44 target chemicals).^32^ Despite this, APCI is rarely used for water analysis today,^31^ and the relative benefits of ESI and APCI have not been reported for environmental water samples.

The current study aimed to explore the molecular complexity of polluted waters in the vicinity of Dhaka, Bangladesh, by using nontarget LC-HRMS combined with both ESI and APCI and an open-source workflow that could be reproduced or adopted by other investigators. To minimize analytical bias and sample handling, sample preparation was kept to a minimum by simple filtering and online solid-phase extraction (SPE), respectively. The relative overlap of molecular features in four ionization modes was compared (*i.e*., ESI ±, APCI ±) to estimate the increased coverage of the chemical space. The sheer complexity of polluted waters was evaluated, and multivariate analyses were used to examine the sample collection site variation and sources of molecular feature profiles. Finally, public MS2 spectral libraries were used to comprehensively identify or annotate unknown features, and all the acquired data were shared in FAIR repositories (Findable, Accessible, Interoperable, Reusable),^33^ including MassIVE^34^ and MassBank Europe,^35^ and an example application is demonstrated with MASST^36^ which could inspire community science environmental surveillance in the future. The open science aspects of the current work are consistent with the philosophy that all water science should be open science and that associated data should be traceable, freely accessible, and reusable by anyone.^37^

## 2 Materials and Methods

### Nontargeted Analysis Study Reporting Tool

The template developed by Peter *et al.*(38) was self-completed and reported in the Supporting Information (SI) (§SI-1) to systematically and transparently report details of methods and quality control in the current work.

### Sampling

Between December 2019 and March 2020, corresponding to the dry season, duplicate surface water samples were collected around Dhaka, the capital city of Bangladesh, from 12 sites along four rivers: Buriganga, Meghna, Shitalakshya, and Turag (Figure 2A, §SI-2). Surface water samples (100 mL) were collected according to a standard operating procedure into 250 mL amber borosilicate bottles precleaned with reverse osmosis water (Millipore Milli-Q Integral 3, Merck) and methanol (LC-MS grade, Optima, Fisher Chemical). Bottles were submersed to a depth of at least 10 cm, facing upstream, opened, and then sealed under water. This procedure was repeated three times with each bottle, discarding the first two rinsates. On the third fill, the excess sample was discarded to reach a final sample volume of 100 mL to accommodate sample preservation by freezing. The samples were kept on ice and stored at −20 °C upon return to the lab in Dhaka. Three field blanks, consisting of reverse osmosis water (Millipore Milli-Q Integral 3, Merck), were transported, stored, and shipped with real samples. Samples were shipped frozen to Sweden on dry ice and arrived frozen.

### Sample Preparation and Analysis

Samples and field blanks were thawed at 4 °C, and 12 mL aliquots were transferred to glass tubes, spiked with 27 isotope-labeled surrogate standards (Table S2), and vortexed prior to filtering with a 0.2 μm syringe filter (Sartorius Minisart RC 15 mm). A 10 mL aliquot of each filtrate was transferred to an injection vial and spiked with two injection isotope-labeled internal standards (Table S2) to monitor instrumental performance. A quality control (QC) sample was prepared by pooling equal volumes of all prepared samples and field blanks.

Instrumental analysis was conducted by online SPE (Oasis HLB, 80 Å, 15 μm, 2.1 × 20 mm, Waters) with analytical separation on a reversed phase column (Acquity UPLC BEH C18, 1.7 μm, 2.1 × 100 mm, 130 Å; Waters, with an Acquity UPLC BEH C18 VanGuard Precolumn 1.7 μm, 2.1 × 5 mm, 130 Å; Waters) at 40 °C. Sample injections (1 mL, full loop, 130 s loading time at 0.5 mL/min) were performed in randomized order for each ionization mode (ESI ± and APCI ±). For each ionization mode, QC injections (n = 5) were made after every 6–7 samples. The flow rate was 0.45 mL/min, and gradient elution was performed using (A) 1 mM ammonium fluoride in LC-MS grade water (Optima, Fisher Chemical) and (B) LC-MS grade methanol (Optima, Fisher Chemical). For all ionization modes, the elution gradient program started at 98% A for 4.2 min, with linear ramping to 100% B over 17.8 min and a hold of 3 min and a return to initial conditions in 0.1 min followed by a 4.9 min equilibration. A retention time index (RTI) mixture of standards^39^ was injected three times per ionization mode. Since the chromatography method was the same for ESI and APCI, the RTI data were combined for data retention time prediction models (§SI-4.6).

Data acquisition was performed in four ionization modes (ESI+, ESI-, APCI+, APCI-) on a QExactive Orbitrap HF-X (Thermo Fisher Scientific). In each mode, mass spectral acquisition was done by a parallel full scan (*m*/*z* range 90–1050, 120,000 resolution FWHM at 200 *m*/*z*) and data-independent MS2 acquisition (DIA; 30,000 FWHM) program with four sequential precursor isolation windows (width of *m*/*z* 245, window centers at *m*/*z* 210, 450, 690, and 930). To confirm molecular annotations, samples were reinjected using the same LC and full-scan methods and data-dependent MS2 acquisition (DDA). DIA was chosen for the nontarget MS2 acquisition as it is reported to be more comprehensive and more sensitive to low intensity features than the DDA approach, while the DDA acquisition was used for confirmation as it provides higher confidence and cleaner MS2 spectra.^40^ Additional information about the chemicals, internal standards, and MS parameters are in the SI (§SI-3.1 and §SI-3.2).

### Mass Spectral Data Processing

An open-source software (MS-DIAL v. 4.60^41^) was used for raw data preprocessing, including peak-picking, centroiding, MS1 extracted ion chromatogram integration, DIA MS2 spectral deconvolution, feature alignment, and gap filling (see all parameters in §SI-3.3.1). Feature peak areas were normalized by both surrogate standards and internal standards responses, specifically by removing systematic variation as evaluated in PCA models^42^ for each ionization mode (see example in §SI-4.1.1) for both field blanks and samples, prior to blank subtraction by the average field blank response.

Multivariate models including principal component analysis (PCA), partial least-squares regression (PLS), and orthogonal PLS directed analysis (OPLS-DA) were built in SIMCA (v. 16.0.1, Umetrics) using log-transformed unit variance-scaled data (*i.e*., normalized features’ areas), whereby each variable is mean-centered and scaled by its standard deviation.^43^ No removal of redundant features was performed, so as to not introduce any bias by prioritizing one ionization mode over the others. Venn diagrams were created in R (v. 4.1.1) to visualize dataset overlap between the four ionization modes based on each feature *m*/*z* (Δ*m*/*z* ± 0.001 Da) and retention time (Δ ± 0.1 min) (see R script in the SI). For simplicity, all of the features detected were assumed to be [M + H]^+^ or [M – H]^−^. We acknowledge that some of the features are adducts (e.g., 5% ammonium adducts according to MS-DIAL), including possibly [M]^+•^ in APCI, but Singh *et al.* have shown these are minor.^32^ The R package ggplot2 (v. 3.3.6) was used for all other data visualizations, and the stats package (v.4.2.1) was used to perform two-sided Welsh’s *t* tests.

### Annotation and Identification of Detected Features

No analytes were targeted, and no suspect lists were employed. An unbiased nontarget workflow was developed based on matching of deconvoluted MS2 spectra to the public databases of MS-DIAL (v. April.13th, 2021^44^) and MassBank Europe (v. 2021-03^45^), combined in a single library file (MSP format). Criteria for annotations with Level 2a confidence^46^ were an MS1 accurate mass error < 5 ppm and MS2 spectral similarity with a reversed match factor (RMF) > 700 (§SI-4.4). To increase confidence in annotations, experimental versus predicted RTI using the quantitative structure–retention relationship model and uncertainty levels developed by Aalizadeh *et al.*(47) were used as orthogonal information. All RTI calibration curves and RTI calculations and predictions^48^ are reported in Table 1 and the SI (§SI-4.6). Annotated features were manually curated for chromatographic peak integration and deconvoluted MS2 spectra quality. Moreover, when a feature was annotated in one ionization mode (ESI or APCI), the other mode and polarities were manually checked for a corresponding feature (retention time shift < 0.1 min and MS1 shift < 5 ppm). Authentic standards were purchased (Table S3) and analyzed to confirm some of the annotations to reach Level 1 confidence^46^ (§SI-3.2). For further confidence, a selection of samples was reanalyzed for confirmation, both nonspiked and spiked (5 μg/L) with the authentic standard, and retention times between the initial injections of the samples and the confirmation step were compared using the RTI mixture^39^ (§SI-4.6). These injections were also used to perform semiquantification (§SI-3.4). To account for intralaboratory retention drift, a linear regression model was used to correct the systematic retention time shift (§SI-4.6.3). In addition to the previous matching criteria, the difference between RTI (<25, Table S51) and MS2 spectra from spiked and nonspiked samples was manually evaluated. All MS2 spectral matches are reported in the SI (§SI-4.3 and §SI-4.4), and all the DDA MS2 spectra from authentic standards were shared on MassBank Europe (v.2022-12^49^). As a step toward the identification of relevant unknowns, we therefore assigned chemical formulas and conducted an MS2-guided in silico structural prediction (Level 3, §SI-3.3.4)^50,51^ for the features having the top 100 VIP scores in the OPLS-DA model.

**Table 1:** Annotated and Identified Compounds in Surface Waters of Bangladesh

class and subclass		compound	identification level^a^	ionization mode	observed *m*/*z* (Da)	Δppm	observed Rt (min)	observed RTI^b^	predicted RTI (uncertainty^c^)^b^	detection frequency	InChIKey
Pesticides and metabolites	Fungicide	Carbendazim	1	APCI+	192.0768	0.00	10.64	343.48	320.29 (1)	12/12	TWFZGCMQGLPBSX-UHFFFAOYSA-N
				ESI+	192.0767	–0.52	10.64				
	Herbicide	2-Hydroxyatrazine	1	APCI+	198.1352	+1.51	11.51	390.33	273.08 (2)	12/12	NFMIMWNQWAWNDW-UHFFFAOYSA-N
				ESI+	198.1342	–0.50	11.55				
		Atrazine	1	ESI+	216.1012	+0.95	14.31	536.66	523.37 (1)	12/12	MXWJVTOOROXGIU-UHFFFAOYSA-N
		Diuron	1	APCI+	233.0243	0.00	14.66	555.61	560.05 (1)	12/12	XMTQQYYKAHVGBJ-UHFFFAOYSA-N
				APCI-	231.0101	+1.73	14.67	541.90	526.26 (1)		
				ESI+	233.0242	–0.43	14.68				
				ESI-	231.0100	+1.30	14.68				
	Insecticide	Chlorpyrifos	1	APCI+	349.9336	0.00	19.41	805.12	863.7 (1)	10/12	SBPBAQFWLVIOKP-UHFFFAOYSA-N
				ESI+	349.9334	–0.57	19.41				
		Diazinon	1	APCI+	305.1083	0.00	17.73	717.21	742.5 (1)	7/12	FHIVAFMUCKRCQO-UHFFFAOYSA-N
				ESI+	305.1080	–0.98	17.74				
		Dimethoate	1	APCI+	230.0070	+0.43	9.94	306.63	310.01 (1)	9/12	MCWXGJITAZMZEV-UHFFFAOYSA-N
				ESI+	230.0069	0.00	9.94				
		Imidacloprid	1	APCI+	256.0598	+0.78	9.57	285.58	328.39 (1)	12/12	YWTYJOPNNQFBPC-UHFFFAOYSA-N
				APCI-	254.0450	0.00	9.51	248.73	306.95 (1)		
				ESI+	256.0896	0.00	9.55				
		Malathion	1	APCI+	331.0436	+0.60	16.22	638.26	473.18 (2)	8/12	JXSJBGJIGXNWCI-UHFFFAOYSA-N
				ESI+	331.0429	–1.51	16.25				
Pharmaceuticals and metabolites	Analgesic	Aspirin (acetylsalicylic acid)	1	APCI+	181.0495	0.00	11.13	370.33	297.48 (1)	12/12	BSYNRYMUTXBXSQ-UHFFFAOYSA-N
				ESI+	181.0497	–2.77	11.16				
		Paracetamol	1	APCI+	152.0706	0.00	6.71	137.67	219.49 (1)	11/12	RZVAJINKPMORJF-UHFFFAOYSA-N
				APCI-	150.0562	+0.67	6.74	88.15	310.91 (3)		
				ESI+	152.0710	+2.63	6.75				
				ESI-	150.0564	+2.00	6.73				
	Antihistaminic	Cetirizine	2a	APCI+	389.1624	–0.77	15.65	607.73	591.44 (1)	11/12	ZKLPARSLTMPFCP-UHFFFAOYSA-N
				ESI+	389.1629	+0.51	15.67	598.47	480.97 (2)		
				ESI-	387.1486	+.129	15.66				
		Fexofenadine	2a	APCI+	502.2952	0.00	14.34	538.77	599.38 (1)	12/12	RWTNPBWLLIMQHL-UHFFFAOYSA-N
				ESI+	502.2950	–0.40	14.36				
	Antibacterial	Sulfamethazine	1	APCI+	279.0909	–0.39	8.72	236.63	281.4 (1)	8/12	ASWVTGNCAZCNNR-UHFFFAOYSA-N
				ESI+	279.0908	–0.72	8.65				
		Sulfamethoxazole	1	APCI+	254.0593	–0.39	5.74	86.61	284.31 (3)	8/12	JLKIGFTWXXRPMT-UHFFFAOYSA-N
				ESI+	254.0596	+0.79	5.78				
		Trimethoprim	1	APCI+	291.1435	–5.84	10.00	310.32	251.68 (1)	12/12	IEDVJHCEMCRBQM-UHFFFAOYSA-N
				ESI+	291.1439	–4.47	10.04	275.59	357.73 (1)		
				ESI-	289.1298	–2.77	10.00				
	Antibiotic	Clarithromycin	1	ESI+	748.4832	–1.34	16.34	643.52	612.35 (1)	6/12	AGOYDEPGAOXOCK-KCBOHYOISA-N
		Cordycepin	2a	ESI+	252.1095	+1.59	6.76	139.25	121.51 (1)	10/12	OFEZSBMBBKLLBJ-BAJZRUMYSA-N
		Erythromycin	1	ESI+	734.4685	0.00	15.24	585.62	529.99 (1)	6/12	ULGZDMOVFRHVEP-RWJQBGvPGSA-N
	Anticonvulsant	Carbamazepine	1	APCI+	237.1022	0.00	13.87	514.56	482.79 (1)	12/12	FFGPTBGBLSHEPO-UHFFFAOYSA-N
				ESI+	237.1020	–0.84	13.90				
		Dihydroxycarbazepine	2a	APCI+	271.1075	–0.74	11.64	396.65	284.74 (2)	12/12	PRGQOPPDPVELEG-UHFFFAOYSA-N
				ESI+	271.1079	+0.74	11.65				
	Antifungal	Fluconazole	1	APCI+	307.1115	+0.65	10.47	334.53	278.18 (1)	12/12	RFHAOTPXVQNOHP-UHFFFAOYSA-N
				APCI-	305.0972	+1.31	10.47	301.88	357.86 (1)		
				ESI+	307.1113	0.00	10.47				
				ESI-	305.0969	+0.33	10.47				
	Antihypertensive	Losartan	1	APCI+	423.1693	–0.47	14.62	553.51	598.79 (1)	11/12	PSIFNNKUMBGKDQ-UHFFFAOYSA-N
				APCI-	421.1557	+1.90	14.61	539.61	737.2 (3)		
				ESI+	423.1692	–0.71	14.65				
		Olmesartan	2a	APCI+	447.2141	+0.45	11.59	393.49	575.28 (2)	12/12	VTRAEEWXHOVJFV-UHFFFAOYSA-N
				APCI-	445.1997	+0.67	11.55	365.88	650.12 (4)		
				ESI+	447.2140	+0.22	11.63				
		Telmisartan	2a	APCI-	513.2306	+1.95	16.56	656.15	766.02 (2)	11/12	RMMXLENWKUUMAY-UHFFFAOYSA-N
				ESI+	515.2452	+1.94	16.59	651.05	735.25 (1)		
				ESI-	513.2300	+0.78	16.59				
	Antiviral	Rimantadine	2a	APCI+	180.1748	+0.56	13.75	508.77	412.65 (2)	12/12	UBCHPRBFMUDMNC-UHFFFAOYSA-N
				ESI+	180.1748	+0.56	13.81				
	Bronchodilator	Salbutamol	1	APCI-	238.1453	+1.68	7.32	170.30	146.91 (1)	12/12	NDAUXUAQIAJITI-UHFFFAOYSA-N
				ESI+	240.1594	0.00	7.38	123.58	223.89 (2)		
	ß-blocker	Atenolol	1	APCI+	267.1704	+0.37	7.57	180.30	132.89 (1)	12/12	METKIMKYRPQLGS-UHFFFAOYSA-N
				ESI+	267.1707	+1.50	7.50				
	Hypoglycaemic agent (antidiabetic)	Metformin	1	ESI+	130.1086	–0.77	3.54	–30.25	–16.94 (1)	10/12	XZWYZXLIPXDOLR-UHFFFAOYSA-N
	Nonsteroidal anti-inflammatory drugs	Diclofenac	1	APCI+	296.040	0.00	15.19	583.51	729.02 (2)	12/12	DCOPUUMXTXDBNB-UHFFFAOYSA-N
				APCI-	294.0095	+0.34	15.16	572.18	587.04 (1)		
				ESI+	296.0239	–0.34	15.24				
				ESI-	294.0098	+1.36	15.21				
		Naproxen	2a	APCI+	231.1017	+0.43	12.69	454.02	587.56 (2)	10/12	CMWTZPSULFXXJA-VIFPVBQESA-N
				ESI+	231.1020	+1.73	12.79				
	Stimulant	Caffeine	1	APCI+	195.0877	0.00	8.56	233.99	220.8 (1)	12/12	RYYVLZVUVIJVGH-UHFFFAOYSA-N
				ESI+	195.0875	–1.03	8.56				
		Cotinine	1	APCI+	177.1025	+1.69	8.17	213.47	183.34 (1)	12/12	UIKROCXWUNQSPJ-VIFPVBQESA-N
				ESI+	177.1021	–0.56	8.17				
		Nicotine	1	APCI+	163.1230	0.00	8.58	230.84	124.14 (2)	10/12	SNICXCGAKADSCV-JTQLQIEISA-N
				ESI+	163.1231	+0.61	8.53				
Personal care products	Antimicrobial/preservative	Propylparaben	1	ESI-	179.0717	+1.68	14.56	535.61	448.3 (2)	10/12	QELSKZZBTMNZEB-UHFFFAOYSA-N
		Triclosan	1	ESI-	286.9441	+0.70	18.79	777.34	788.2 (1)	12/12	XEFQLINVKFYRCS-UHFFFAOYSA-N
	Skin conditioning agents	Panthenol	1	APCI+	206.1388	+0.49	6.84	143.98	134.34 (1)	11/12	SNPLKNRPJHDVJA-UHFFFAOYSA-N
				APCI-	204.1242	+0.49	6.83	95.01	231.27 (2)		
				ESI+	206.1391	+1.94	6.86				
				ESI-	204.1245	+0.98	6.86				
	Foaming agent	Lauryl diethanolamide	2a	APCI+	288.2534	+0.35	18.66	765.64	676.75 (2)	8/12	AOMUHOFOVNGZAN-UHFFFAOYSA-N
				ESI-	286.2390	+.070	18.66	769.91	674.97 (2)		
	UV filter	Oxybenzone	1	APCI+	229.0860	+0.44	17.07	682.47	637.58 (1)	11/12	DXGLGDHPHMLXJC-UHFFFAOYSA-N
				APCI-	227.0716	+0.88	17.08	679.62	521.59 (2)		
				ESI+	229.0864	+2.18	17.09				
				ESI-	227.0719	+2.20	17.08				
Industrial compounds	Surfactant	C10 linear alkyl benzenesulfonate (C10-LAS)	2a	APCI-	297.1527	–1.01	18.27	779.63	403.8 (4)	8/12	NANHIUZYPFDGJS-UHFFFAOYSA-N
				ESI-	297.1534	+1.35	18.28				
		C11 linear alkyl benzenesulfonate (C11-LAS)	2a	APCI-	311.1690	+1.29	18.82	779.63	455.06 (4)	7/12	FERBTPHUEYEGDN-UHFFFAOYSA-N
				ESI-	311.1687	+0.32	18.84				
	Dye	Indigo blue	2a	APCI+	263.0812	–1.14	10.76	351.90	507.08 (2)	12/12	QQILFGKZUJYXGS-UHFFFAOYSA-N
				ESI+	263.0816	+0.38	10.80	320.74	419.21 (2)		
				ESI-	261.0674	+1.53	10.76				
	Various use	1-Naphthalenesulfonic acid	1	APCI-	207.0125	+1.93	8.54	191.02	223.29 (1)	12/12	PSZYNBSKGUBXEH-UHFFFAOYSA-N
				ESI-	207.0124	+1.45	8.52				
		2-Naphthalenesulfonic acid	1	APCI-	207.0124	+1.45	9.09	224.73	230.07 (1)	11/12	KVBGVZZKJNLNJU-UHFFFAOYSA-N
				ESI-	207.0128	+3.38	9.14				
		Hydroxyquinoline (OH position not known)	2a	APCI+	146.0602	+1.37	10.23	322.43	328.99 (1)	11/12	
				APCI-	144.0456	+0.69	10.22	288.74	241.27 (1)		
				ESI+	146.0601	+0.68	10.23				
				ESI-	144.0459	+2.78	10.27				
		4-Methyl-1H-benzotriazole **and**5-Methyl-1H-benzotriazole (coelution)	1	APCI+	134.0713	0.00	10.85	354.01	359.35 (1) and 364.31 (1)	12/12	CMGDVUCDZOBDNL-UHFFFAOYSA-N **and** LRUDIIUSNGCQKF-UHFFFAOYSA-N
				APCI-	132.0568	+0.76	10.82	323.02	308.50 (1) and 336.97 (1)		
				ESI+	134.0714	+0.75	10.85				
				ESI-	132.0572	+3.79	10.84				
		Bis(2-ethylhexyl) phosphate	1	APCI+	323.2343	–0.93	19.11	790.38	856.58 (1)	9/12	SEGLCEQVOFDUPX-UHFFFAOYSA-N
				APCI-	321.2197	–0.93	19.18	796.77	857.00 (1)		
				ESI+	323.2347	+0.31	19.12				
				ESI-	321.2202	+0.62	19.12				
		Diphenyl phosphate	1	APCI+	251.0471	+1.19	11.85	407.70	401.74 (1)	11/12	ASMQGLCHMVWBQR-UHFFFAOYSA-N
				APCI-	249.0323	+0.40	11.83	381.31	307.00 (1)		
				ESI+	251.0470	+0.80	11.88				
				ESI-	249.0328	+2.41	11.86				
		Metazin = Hexa(methoxymethyl) melamine	2a	APCI+	391.2301	+0.26	14.06	524.56	563.72 (1)	10/12	BNCADMBVWNPPIZ-UHFFFAOYSA-N
				ESI+	391.2308	+2.04	14.09				
		Quinoline	1	APCI+	130.0653	+1.54	12.05	418.23	411.84 (1)	12/12	SMWDFEZZVXVKRB-UHFFFAOYSA-N
				ESI+	130.0652	+0.77	12.06				
		Sulfanilic acid	2a	APCI-	172.0077	+1.74	3.60	–90.15	185.37 (4)	7/12	HVBSAKJJOYLTQU-UHFFFAOYSA-N
				ESI-	172.0080	+3.49	3.61				
		Triisobutyl phosphate	2a	ESI+	267.1720	0.00	18.45	754.59	799.68 (1)	12/12	HRKAMJBPFPHCSD-UHFFFAOYSA-N
		Tris(2-butoxyethyl) phosphate	1	APCI+	399.2509	+0.75	19.00	784.06	861.66 (1)	12/12	WTLBZVNBAKMVDP-UHFFFAOYSA-N
				ESI+	399.2509	+0.75	19.01				
Miscellaneous	Endogenous steroid	Dehydroisoandrosterone sulfate	2a	ESI-	367.1589	+1.09	14.50	532.18	338.42 (3)	11/12	CZWCKYRVOZZJNM-USOAJAOKSA-N
	Isoflavone	Daidzein	1	APCI+	255.0656	+1.57	12.73	453.50	511.82 (1)	12/12	ZQSIJRDFPHDXIC-UHFFFAOYSA-N
				APCI-	253.0509	+1.19	12.71	431.03	474.21 (1)		
				ESI+	255.0649	–1.18	12.75				
				ESI-	253.0512	+2.37	12.73				
	Food additives, flavoring agent	2-methylindole	2a	ESI+	132.0809	+0.76	8.54	232.94	390.25 (2)	12/12	BHNHHSOHWZKFOX-UHFFFAOYSA-N
	Plant metabolite	Indole-3-carbinol	2a	APCI+	148.0757	0.00	10.89	356.64	249.17 (2)	12/12	IVYPNXXAYMYVSP-UHFFFAOYSA-N
				ESI+	148.0758	+0.68	10.89				
	Sweetener	Acesulfame	1	APCI-	161.9869	+1.23	3.77	–73.00	52.37 (2)	7/12	YGCFIWIQZPHFLU-UHFFFAOYSA-N
				ESI-	161.9871	+2.47	4.05				
		Saccharin	2a	APCI-	181.9920	+1.65	5.41	12.72	221.08 (3)	9/12	CVHZOJJKTDOEJC-UHFFFAOYSA-N
				ESI-	181.9923	+3.30	5.41				
		Sucralose	1	APCI-	395.0074	+0.25	9.07	221.87	276.86 (1)	11/12	BAQAVOSOZGMPRM-QBMZZYIRSA-N
				ESI-	395.0082	+2.28	9.07				
	Vitamin B6 metabolite	4-Pyridoxic acid	2a	ESI+	184.0605	+0.54	5.94	94.50	91.41 (1)	12/12	HXACOUQIXZGNBF-UHFFFAOYSA-N
				ESI-	182.0464	+2.75	5.88	41.29	63.15 (1)		

a Based on the scale of Schymanski *et al.*(46) Level 1 is a confirmed structure by reference standard, and Level 2a is a probable structure by library spectrum match.

b When two RTI values are reported, the first one is for positive ionization mode(s), and the second one is for negative ionization mode(s).

c Based on the QSRR model by Aalizadeh *et al.*(117) (1) candidate acceptable, (2) candidate acceptable, with an error, (3) candidate not reliable, and (4) candidate not reliable, outside model domain.

All mass spectrometry data were uploaded and made publicly available on the MassIVE^34^ repository (see the SI for details). We also used MASST^36^ to search for spectral matches between selected molecular features detected in the current water sampling campaign and all publicly available datasets on MassIVE using the following parameters: parent mass tolerance 0.002 Da, fragment ion mass tolerance 0.005 Da, 3 minimum matched peaks, and a matching score threshold of 0.7. The MS2 spectra selected for searching on MASST included the top ten unannotated features with highest average intensity among all samples, and the most intense Level 1 compounds of each contaminants’ classes, *i.e*. bis(2-ethylhexyl) phosphate, carbendazim, cotinine, daidzein, and oxybenzone, to show the interest of this tool. All precursor ions and DIA MS2 of the identified and unknown features from our samples are provided in the SI (Table S5 and Table S6).

## 3 Results and Discussion

### Complementary Use of 4 Ionization Modes

For the nontarget analysis of rural and polluted urban waters in Bangladesh, complementary molecular information was gained by analyzing each sample with four distinct ionization modes. After data curation and blank filtering, a total of 39,025 features were detected across all samples, specifically 15,402 in ESI+, 10,395 in ESI-, 6776 in APCI+, and 6452 in APCI-. Together, the two ESI modes resulted in almost twice as many feature detections (25,797 features) as the APCI modes (13,228 features). To some extent, this could be explained by fewer molecular ion adducts in APCI than ESI, particularly in positive ionization mode, as MS-DIAL does not perform a grouping step for the adducts.^52,53^ However, the results likely reflect the wide physicochemical properties of substances present and their relative abilities to be ionized by an APCI or ESI mechanism.^52,54,55^ For example, the average *m*/*z* of features in APCI- (*m*/*z* 279) was lower than ESI- (*m*/*z* 314, two-sided Welsh *t* test *p* < 0.001), and much lower in APCI+ (*m*/*z* 264) than in ESI+ (*m*/*z* 338, *p* < 0.001). Moreover, while 10% of ESI- and 14% of ESI+ features were in the range of 500–1050 Da, less than 3% of features in either APCI mode had *m*/*z* > 500. These systematic mass differences cannot be explained by adducts alone.

**Figure 1:**
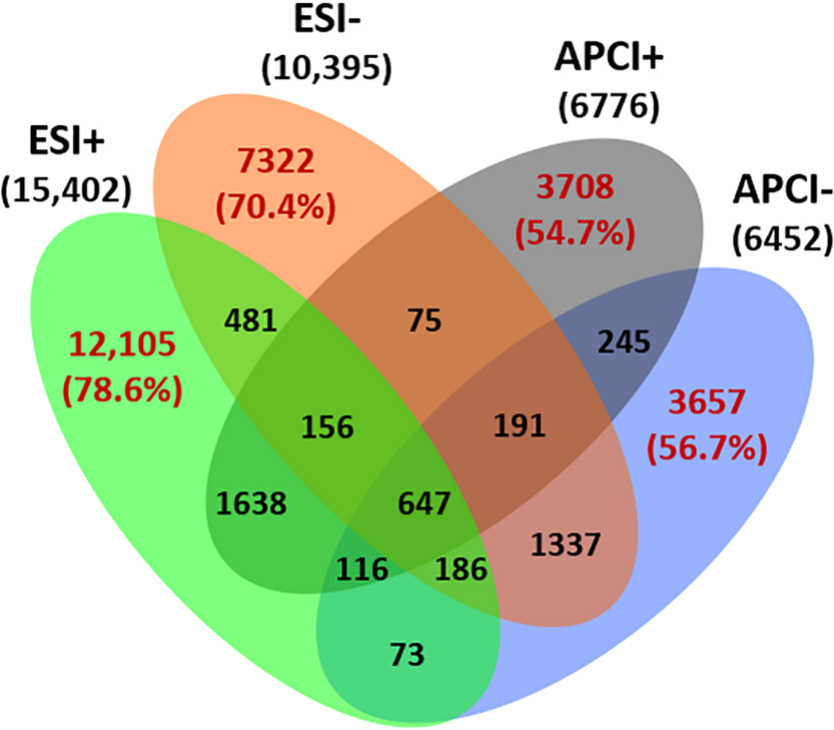
Venn diagram of 39,025 total nontarget features detected in Bangladesh surface waters in the four ionization modes (ESI ± and APCI ±). Highlighted in red font are the 26,792 unique features among each ionization mode, and percentages are respective to each ionization mode.

Feature redundancy was furthermore evaluated between the four ionization modes, and the majority of features in each ionization mode (*i.e.*, 54.7–78.6%) was unique (Figure 1). There were 26,792 unique features in total (68.6% of the dataset), corresponding to 12,105 and 7322 unique features in ESI+ and ESI-, respectively, and 3,708 and 3,657 unique features in APCI+ and APCI-, respectively (Figure 1). Singh *et al.* previously compared APCI ± and ESI ± modes for 1264 target spiked substances and reported a 4% increase in analyte coverage with additional use of APCI,^32^ but here, we report a 19.5% increase of nontarget features by using APCI in environmental water samples. To our knowledge, the current study is the first to test this complementary approach in environmental waters, and the results emphasize the advantages of combining both ionization sources to increase chemical space coverage and reduce bias in nontarget water analysis.

### Unsupervised Multivariate Analysis

**Figure 2:**
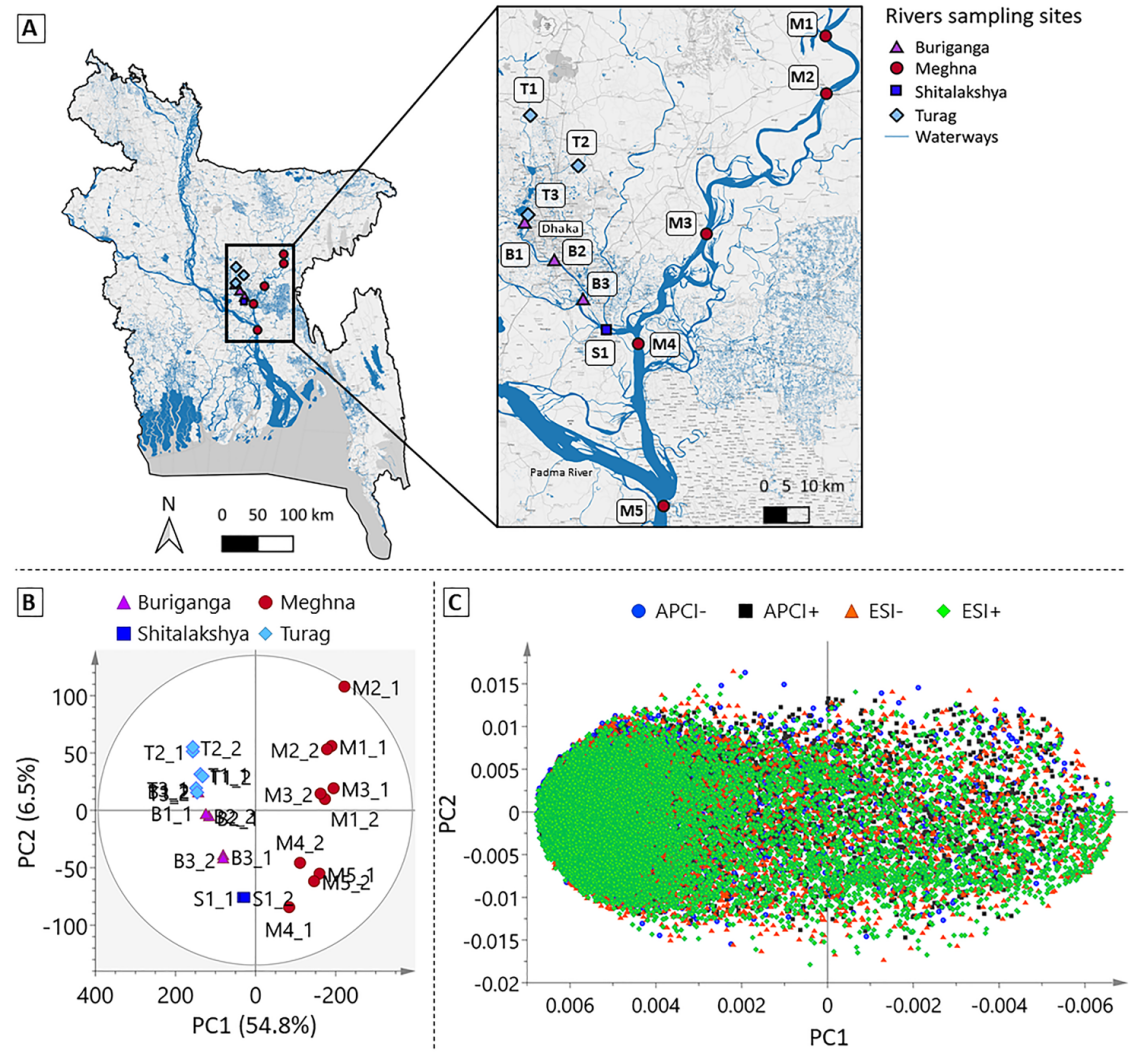
Location of the sampling sites, labeled corresponding to the river name (B: Buriganga, M: Meghna, S: Shitalakshya, T: Turag) and numbered from upstream to downstream (e.g., 1–5) with duplicates shown (e.g., _1, _2), and PCA of their nontarget feature profiles. (A) Sampling sites on four major rivers around Dhaka, Bangladesh (© OpenStreetMap contributors, CC-BY-SA, see §SI-2 for details about the map creation). (B) Surface water samples by site projected in a PCA (2 components) scores plot and (C) total nontarget features (*n* = 39,025) from four ionization modes projected on a loadings plot. Note the *x*-axis directionality inverted in scores and loadings plots.

The full dataset consisting of all four ionization modes combined, without removal of redundant signals, was examined in a PCA (Figure 2B). The scores plot revealed two groups of samples, corresponding to a major east–west separation of the Bangladesh rivers between highly urbanized (*i.e*., west) and more rural (*i.e*., east) sampling sites along the first principal component (PC1), explaining 54.8% of total variation in the dataset. A secondary upstream-downstream distribution was evident along PC2 (6.4% of variation), suggesting an increasing pollution gradient along the flow paths of both urban and rural rivers. The associated loadings plot (Figure 2C) shows that the urban rivers flowing through Dhaka had the greatest chemical complexity, with higher numbers of features detected in all modes (see separate loadings plots for each ionization mode in Figure S3).

### Supervised Multivariate Models

**Figure 3:**
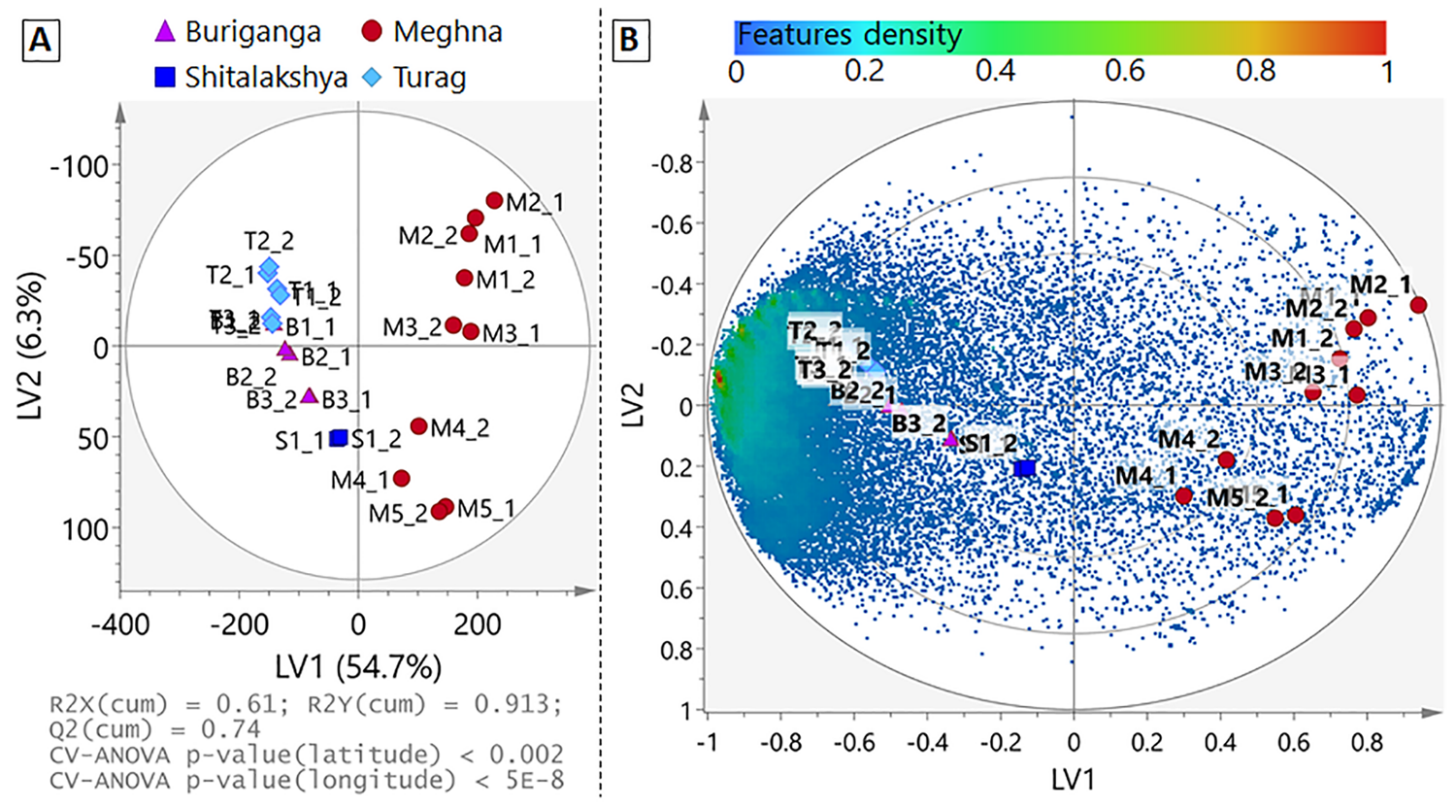
PLS model (2 latent variables, LV) displaying association between the site location (latitude and longitude set as Y variables) and total molecular features. (A) Scores plot of surface water sampling sites and (B) the corresponding biplot with each square marker representing a molecular feature (*n* = 39,025), colored by density distribution, increasing from blue, to green, to red. Note the *y*-axis directionality inverted in the scores plot and the biplot.

Based on results of the PCA, a PLS model was constructed using latitude and longitude coordinates of the sampling sites as response parameters (Y variables). This further confirmed that geographical location was a main factor in the data distribution (R2Y(cum) = 91.3%, Figure 3A), as the PLS biplot showed the greatest feature density correlated to the urban sampling locations (T1-T3, B1-B3, S1, Figure 3B). Nevertheless, it is important to note that many nontarget features were correlated to the more rural sampling locations of the upper Meghna (e.g., sites M1 to M3, Figure 3B), and that moving downstream there was a shift toward more complex nontarget feature profiles with a greater density of features (*i.e*., M1-M3 each had approximately 21,000 features, while M4 had >26,000). Based on internal standards responses among samples, the trend in feature numbers along the Meghna River cannot be explained by matrix effects (e.g., Figure S1A) nor by any instrumental sensitivity drift, as the samples were injected in random order (both between sampling sites and ionization modes).

**Figure 4:**
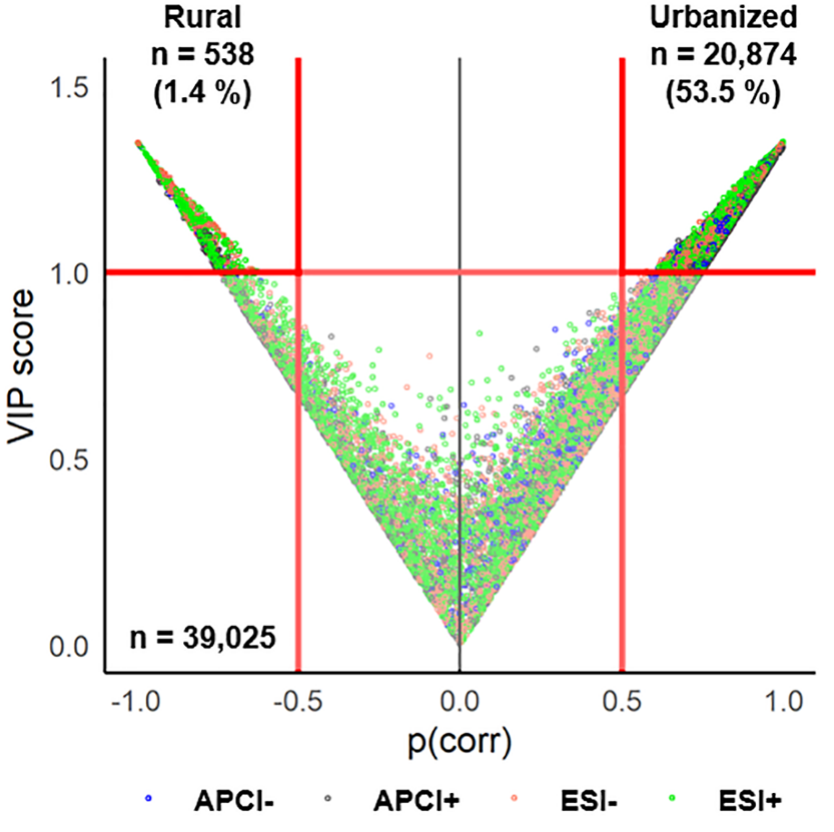
Scatterplot of the OPLS-DA model (1+1+0) results, showing the contribution of each nontarget feature (*n* = 39,025) to urban (T1-T3, B1-B3, S1) or more rural (M1-M5) sampling site locations. Features in the upper left and right quadrants have the highest model correlation coefficients (|p_corr_| > 0.50) and variable importance for the projection scores (VIP > 1.0). The features are colored by ionization mode, and separate plots for each ionization mode are available in the SI (Figure S6).

To objectively group the nontarget features that most strongly correlated with the urban sites (T1-T3, B1-B3, S1), or with more rural sites (M1-M5), an OPLS-DA model was constructed (§SI-4.2.3, Figure S5). The relative influence of each nontarget feature in the model was visualized by a plot of the variable importance for the projection (VIP) scores versus model-correlation coefficients (p_corr_), and the two regions with the highest VIP and p_corr_ are defined by the upper left and right quadrants (Figure 4). This analysis confirmed not only that most detected features (n = 20,874, 53.5% of the dataset) were indeed correlated with the urban locations but also that numerous other features were correlated with the rural locations (n = 538, 1.4%) (Figure 4).

### Implications of Nontarget Feature Profiling

As anticipated, these results support that anthropogenic activities have a great impact on the chemical composition and quality of surface waters in Dhaka. Some of the detected features may be natural compounds; however, the water of the Turag and Buriganga Rivers has been reported as heavily polluted and in an “ecological critical state”, mostly because of industrial discharges.^17,56^ Only about 30% of Dhaka’s population is connected to any form of sewage collection system,^57^ and another 30% of the population releases septic tank water through drainage networks or in open channels,^58−60^ leading to widespread nonpoint surface water contamination.

Notably, a wastewater treatment plant servicing approximately 20% of Dhaka’s population (approximately 5 million people) with a treatment capacity of 120,000 m^3^ of wastewater per day^61−63^ discharges between sampling sites B2 and B3 on the Buriganga River. The Buriganga River flow rate has been reported to be between 20 and 200 m^3^/s during the dry season,^64^ and the wastewater effluent discharges at an average flow rate of 0.35 m^3^/s (Taqsem A. Khan, Managing Director of Dhaka Water Supply and Sewerage Authority, personal communication), giving an effluent dilution factor varying from 60 to 570. In high-income countries, wastewater treatment plants are major point sources of complex contaminant mixtures to surface waters;^65−69^ thus, it is interesting that the nontarget feature profile was actually shifted toward molecular profiles of less complex rural samples at site B3, both in the PCA and in the PLS model first components (Figures 2 and 3). These important observations on the Buriganga River in Dhaka suggest that treated wastewater was less chemically complex than upstream surface water, with the implication that unregulated upstream releases to the Turag and Buriganga Rivers are the major sources of most substances detected here by nontarget analysis. These first empirical results by nontarget analysis are therefore encouraging that improved surface water quality around Dhaka can be achieved by improving sewage collection and wastewater treatment infrastructures, as previously suggested.^15−17,23,70−72^

Contamination of urban rivers in Dhaka not only threatens the environment but also poses risks to human health through various exposure pathways. Both the Buriganga and Shitalakshya Rivers are used as drinking water sources, as will the water from the Meghna River in the future.^73^ Moreover, surface water seepage to groundwater has previously been reported as a mode of contamination to the underlying Dupi Tila aquifer in Dhaka,^74,75^ which provides drinking water to 78% of the local population.^73^ Water from the Turag River is also used for irrigation in agriculture,^17^ which could result in contamination of food crops, and this continues to be a knowledge gap in terms of human health impacts of chemical contaminants.^76−83^

### Nontarget Annotation and Identification of Organic Micropollutants

All detected features in the current study had an associated deconvoluted MS2 spectrum from DIA MS2 acquisition that was searched against public spectral libraries (MassBank, MS-DIAL). This resulted in 62 preliminary annotations at Level 2a confidence, including 25 pharmaceuticals (e.g., diclofenac), 15 industrial chemicals (e.g., diphenyl phosphate), 9 pesticides (e.g., diuron), 5 personal care products (e.g., oxybenzone), and 8 miscellaneous compounds such as sweeteners and endogenous human metabolites (Table 1). Authentic standards (n = 44) were subsequently obtained and analyzed with the current analytical method, and 41 of these were confirmed with Level 1 confidence (Table 1, §SI-4.3). Only two annotations were false positives (memantine and tri-*n*-butyl phosphate) based on unmatched retention times, and one remains inconclusive (indigo blue, no increase in the corresponding peak’s intensity after spiking the sample with indigo blue at 5 μg/L). The two false positives were later annotated as isomers of the original matches. Among the 62 annotations overall, 29 Level 1 identifications and 15 Level 2a annotations were correlated (VIP > 1 and p_corr_ > 0.50 in the OPLS-DA model) to the urban impacted samples in at least one ionization mode (Table S46), while no annotated compounds were correlated to the more rural sampling sites. We prioritized the 100 most relevant features based on the OPLS-DA model (top VIP scores) for formula assignments and structural predictions, and 30 of these had a chemical formula successfully predicted with the applied criteria (§SI-4.7.2), most of which were detected in negative mode. Structural predictions suggested that 15 of these may be anthropogenic (e.g., industrial compounds), while 7 were prospective human endogenous metabolites. One structure predicted to be methyl 2,2-dichloropropionate (C_4_H_6_Cl_2_O_2_, a metabolite of the herbicide Dalapon) was correlated to the Meghna River sample locations. The structures predicted were not further evaluated using authentic standards and therefore are assigned Level 3 confidence.

Previous LC-(HR)MS based studies of organic micropollutants in surface waters of Bangladesh have mainly focused on targeted analysis of pharmaceuticals^5,26^ and pesticides,^21^ with the exception of one study that also performed suspect screening.^23^ Specifically, in samples coming from Dhaka and the administrative area of Matlab, Angeles *et al.* performed quantification of 13 target pharmaceuticals (mainly antibiotics) and a retrospective suspect screening based on suspect lists from the NORMAN network and the US EPA (1156 substances).^23^ The authors screened for suspect compounds by precursor *m*/*z* and later attempted matching when the DDA MS2 spectra could be compared to the mzCloud database.^23^ In addition to the 13 detected target analytes, they annotated 28 additional compounds using suspect screening, which were mostly pharmaceuticals but also included 3 pesticides (carbendazim, DEET, diuron).^23^ Of the total 41 analytes that they confirmed or annotated (13 Level 1; 28 Level 2a), 15 of these substances were identified in the current study at Level 1 confidence using the nontarget workflow which is less biased but still limited to spectral matches in open MS2 libraries.

More recently, Wilkinson *et al.*(5) reported 61 target pharmaceuticals in global surface waters. Among the 11 pharmaceuticals they detected in Bangladesh, we detected 7 of these at all sampling sites using the current nontarget analysis (*i.e.*, carbamazepine, fexofenadine, fluconazole, metformin, paracetamol, sulfamethoxazole, and trimethoprim; Table 1). Hossain *et al.* targeted 12 pharmaceuticals and detected 9 at concentrations up to 17.2 ng/L (for trimethoprim) in samples from 20 sampling sites on the Brahmaputra River.^26^ Although they detected metronidazole and trimethoprim with the highest detection frequency (100% and 95%, respectively),^26^ only trimethoprim was identified in the current campaign by nontarget analysis (100% detection frequency, maximum concentration of 2.8 ng/L, Table S53). The nondetection of metronidazole in our study could be due to its zwitterionic nature at our samples pH (Table S1) and mobile phase pH, limiting the online SPE concentration step. Among the other pharmaceuticals semiquantified, 6 had maximum concentrations higher than 500 ng/L (e.g., caffeine, 541 ng/L; paracetamol, 1979 ng/L; and nicotine, 3738 ng/L; Table S53). In other studies of LC-amenable pesticides in Bangladesh, often ultraviolet or diode-array detectors were used, and carbofuran, chlorpyrifos, diazinon, and malathion have been reported in drinking water (*i.e.*, tube-well) and surface waters at very high concentrations (*i.e.*, tens to hundreds of μg/L), presenting known risks to human health.^16,21,84−86^ Of these, chlorpyrifos, diazinon, and malathion were identified (Level 1) in surface waters in the current nontarget study at maximum concentration of 30, 65, and 8 ng/L, respectively (Table S53), and diazinon was correlated to the urban samples (VIP = 1.147, p_corr_ = 0.839). The semiquantified pesticides with the highest concentrations in the current study were imidacloprid and diuron, having maximum concentrations of 300 and 100 ng/L, respectively (Table S53).

Some industrial compounds were detected in all river samples, such as triisobutyl phosphate (Level 2a, Table 1, Figure S51). Triisobutyl phosphate has widespread uses,^87^ e.g. flame-retardant and plasticizer.^88^ To the best of our knowledge, triisobutyl phosphate is not a concern for the environment and human health,^89,90^ despite dust concentrations higher than 1 μg/g in several studies.^90,91^ However, it was detected along with tris(2-butoxyethyl) phosphate (Level 1) in the current samples (median concentration 3300 ng/L), another organophosphate compound with widespread uses. Tris(2-butoxyethyl) phosphate has shown concerning effects for the environment,^90^ e.g. endocrine disruption for zebrafish,^92,93^ at environmentally relevant concentrations.^94^ These two organophosphates might co-occur with other organophosphates in the samples, raising concern for both the environment and human health: some are used as flame retardants to replace polybrominated diphenyl ethers, with associated health concerns.^95^ Indigo blue dye (Level 2a), another industrial compound, is widely used in the textile industry to dye denim fabrics^96^ and can be mixed with other dyes and mordents that could be highly toxic.^97^ Its degradation during wastewater treatment has proven challenging, and it can degrade to more toxic products.^96,98,99^ While indigo blue has been assessed as a low potential risk for human health and the environment,^100,101^ its detection in all current samples is concerning as it may be a marker of other dyes and organic micropollutants related to the dyeing industry, which could be harmful for both the environment and human health.

**Figure 5:**
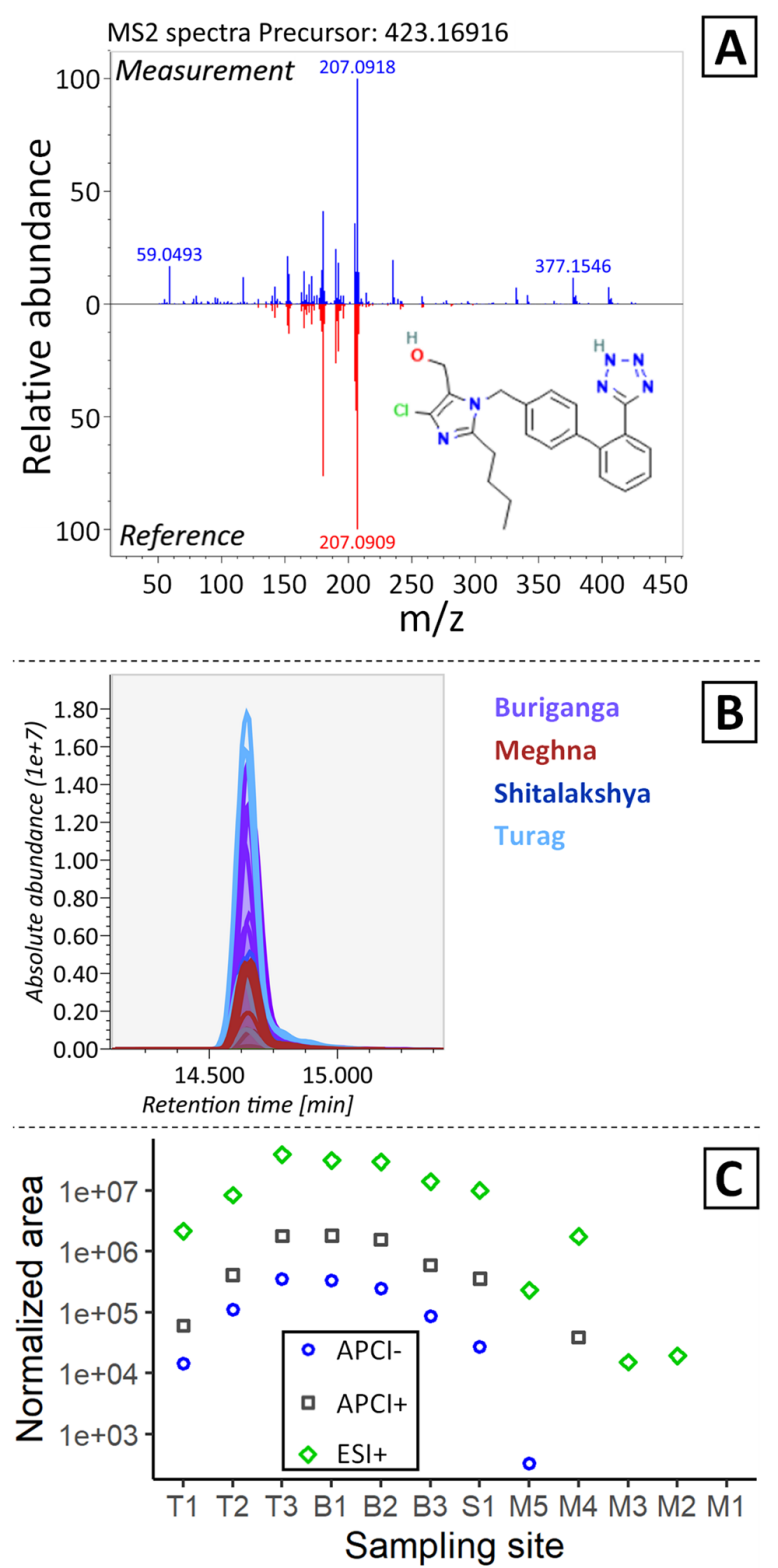
Losartan Level 2a annotation (later confirmed as Level 1) and distribution among sites. (A) MS2 spectrum in the samples in ESI+ (blue), compared to the reference spectrum of losartan ([2-butyl-5-chloro-3-[[4-[2-(2H-tetrazol-5-yl)phenyl]phenyl]methyl]imidazol-4-yl]methanol) in ESI+ from a public database (red, from GNPS using MS-DIAL) and (B) aligned extracted ion chromatograms from all samples. (C) Spatial distribution is shown by the average normalized area at each sampling site (*n* = 2) for the three ionization modes where this feature was detected.

Most of the detected compounds showed similar spatial patterns as the antihypertensive compounds losartan (Level 1, Figure 5), olmesartan, and telmisartan (both Level 2a, Figure S54). They showed greater levels in the Turag, Buriganga, and Shitalakshya Rivers (Figure 5C, Figure S54), consistent with results of the PLS model for thousands of nontarget features (Figure 3). We detected the antihypertensives with higher intensity at the urban sites closest to Dhaka (*i.e*., sites T1 to T3, B1 to B3, and S1; Figure 5C), but it was also elevated in the lower Meghna River (*i.e*., site M4) after confluence and dilution by the rivers flowing from Dhaka, suggesting that the main sources of contamination are urban.

A few annotated substances (Level 1 or Level 2a) demonstrated different spatial patterns than the overall trend for most features. For example, the antidiabetic drug metformin (Level 1, Figure S32) had greatest levels in the Shitalakshya River (site S1), as well as at the confluence of the Meghna River and rivers flowing from Dhaka (site M4) (Figure 2A, Figure S54). The personal care products annotated (e.g., propylparaben, triclosan, and oxybenzone), as well as 2-hydroxyatrazine, malathion, and rimantadine, had unique distribution patterns (Figure S54). They were detected at similar or higher levels in some of the Meghna River samples compared to the urban rivers (Figure S54) and thus are expected to have major sources outside Dhaka. Malathion is approved to treat crops in Bangladesh,^102^ which could explain its detection in the upper Meghna, whereas the urban sources may include its use for mosquito control.^103^

The extensive use of antibiotics and antimicrobials for human prescriptions, consumer products, aquaculture, and for veterinary purposes has been raised as a concern in Bangladesh,^16,23,104^ not only due to their potential toxicity but also due to their potential to increase antibiotic-resistant pathogens.^105,106^ Other organic micropollutants may also involuntarily trigger antibiotic-resistance in bacterial populations. A recent study highlighted DNA mutations leading to multiple-antibiotic resistance in *Escherichia coli* after an exposure to metformin, a pharmaceutical intended for type 2 diabetes treatment, after only 1 day of exposure at low concentrations from 1 ng/L.^107^ In addition to various antibiotics and antimicrobials (e.g., clarithromycin, cordycepin, sulfamethoxazole, and triclosan), metformin was detected in most of the current samples (*i.e.*, 10/12, Table 1) at a maximum concentration of 870 ng/L (Table S53).

In addition to various pesticides and pharmaceuticals, the two linear alkyl benzenesulfonate (LAS) surfactants C10-LAS and C11-LAS were detected (Level 2a, Table 1 and Figure S50). Their abundance was correlated to urban sample locations (Table S46), as well as to some of the detected pesticides (e.g., carbendazim, diazinon, dimethoate, diuron, and imidacloprid, Figure S60). Target monitoring studies rarely examine surfactants and pesticides together, despite that surfactants are mixed with pesticides to improve pest control formulations.^108^ Unfortunately, the co-occurrence of surfactants can increase pesticide mobility through the soil,^109^ increasing the risk that pesticides will reach groundwater and drinking water resources. Surfactants can also increase the toxicity of pesticides toward both developing and adult aquatic organisms^110^ and slow their degradation and have similarly been shown to slow the degradation kinetics of pharmaceuticals.^111^

Recent publications clearly highlight the benefits of increased monitoring for pharmaceuticals in surface waters around the world from diverse geographic and socioeconomic zones.^3,24^ However, multiclass or wide-scope target analysis, suspect screening, and nontarget approaches could be combined with such efforts for broader understanding of total chemical pollution and to identify emerging contaminants in today’s major global source regions that include low- and middle-income countries producing extensive goods for export to high-income countries. A great strength of nontarget analysis is the possibility to collect information about contaminants belonging to different chemical families and with a wide variety of applications. This can provide essential data on chemical mixtures that need to be increasingly evaluated for risks to humans and wildlife. The current study reveals the molecular complexity of anthropogenic emissions to water, most of which is not identified and remains unannotated, representing great opportunity for future water monitoring, including by community science projects.

### Open Science Perspectives and Contributions

Among the total 39,025 features detected in this study, only 62 were annotated, leaving the structures of most detected features unknown. An acknowledged major limitation of unbiased nontarget analysis today is that annotation is only possible when an authentic MS2 spectrum is available for matching in an accessible spectral library. These resources are still rather limited in coverage but are growing through community contributions. A further limitation of the current work, which acquired thousands of unique spectra in APCI mode, is that the majority of public spectral records are from ESI mode. Fragmentation patterns may be similar between ESI and APCI modes, but expanding the number of APCI MS2 spectra in open libraries will improve annotation capacity in future studies, especially for compounds that ionize poorly with ESI. To this end, we uploaded 107 MS2 spectra to MassBank Europe, in both ESI (n = 57) and APCI (n = 50).

The importance of open science approaches and FAIR data management is elevated for publicly funded research in low-income countries, as this is a step toward ensuring equitable use of large datasets for the benefit of all people, including marginalized populations.^112^ The current mass spectrometry data were uploaded and made publicly available on MassIVE^34^ repository. This not only allows the data to be shared and reused by any researcher but also opens use to other powerful open science tools, including feature based molecular networking^113^ which we have previously applied to other environmental datasets.^114−116^ Another applicable community science tool that we investigated with the current dataset is MASST.^36^ This is a web-based tool that searches the public MassIVE repository for a single MS/MS spectrum of interest, including known and unknown features. First, we tested this with our deconvoluted MS2 spectra of the 5 most intense Level 1 compounds, and in each case, it proved successful. For example, the spectrum of our identified feature bis(2-ethylhexyl) phosphate (a compound with various use, e.g. lubricant additive, cleaning and furnishing care products) was matched to 1 dataset from human saliva samples, while daidzein had 96 matches, mostly in plant and food samples (25 matches), which is expected as it is a natural isoflavone in many dietary plants. Our spectral feature identified as oxybenzone (a UV filter) had matches in 41 datasets, including in clothing and environmental samples (e.g., sweater, soil samples), maize, honey, and in human samples (e.g., saliva, skin). The spectral feature identified as cotinine had 10 matches, mostly in human samples (e.g., breast milk, serum) but also in environmental samples (e.g., water). The identified spectrum of carbendazim had 3 relevant matches, including in mattress dust samples, drinking fountain water, and apple samples.

MASST was then applied to the 10 unknown features showing the highest average intensity in the samples (after blank subtraction). Among these, two spectra matched with other public datasets in MassIVE. One was matched with datasets from marine dissolved organic matter and algal extracts that were acquired at two different times. The second feature matched with 11 datasets, among which 4 were metabolomics studies of the gastrointestinal tract and 4 were metabolomics studies of microorganisms. This latter feature could therefore be related to microorganisms present in the current samples, possibly from human sewage.

Although the 8 other features did not find a match, this is likely because of the low number of environmental datasets currently on MassIVE, which is primarily a metabolomics resource. Therefore, MASST remains a promising open science tool that can feasibly be used for environmental surveillance of known substances and “known unknown” molecular features as more environmental datasets are deposited, yet simultaneously screening a wide variety of datasets from other experimental and sample matrices is advantageous compared to an isolated environmental mass spectral repository. Nevertheless, current limitations of MASST are that batch searching on MS2 data is not yet available, and it is not possible to check if the features that were matched to a public dataset were annotated/identified, as the authors do not always provide links to their preprint or published paper. Moreover, datasets on MassIVE are raw data and should be blank corrected when accessed for environmental purposes, but the (method) blanks are not always provided. Journals in environmental chemistry do not yet demand that mass spectral datasets to be deposited in repositories as a condition of publication, as is common in genomics, transcriptomics, proteomics, and metabolomics, but here we demonstrate that there are already infrastructures to support this when such policies inevitably develop.

## Data Availability

Mass spectrometry datasets are made available at the GNPS Mass Spectrometry Interactive User Environment (MassIVE) database under the following access numbers: APCI+ MSV000089703, APCI- MSV000089704, ESI+ MSV000089705, and ESI- MSV000089706.

## Supporting Information Available

Additional information consists of the Venn diagram R script (10.6084/m9.figshare.21493695), mass spectrometry datasets, and a pdf file (PDF)
