# Supplementary material for: Nontarget Analysis of Polluted Surface Waters in Bangladesh Using Open Science Workflows
Source: Environ Sci Technol. 2023 Apr 21;57(17):6808–24. doi: 10.1021/acs.est.2c08200 (PMC10157886; doi:10.1021/acs.est.2c08200)
Supplement: Supplementary file 1 — es2c08200_si_001.pdf [file es2c08200_si_001.pdf]

Supporting information for:

Nontarget Analysis of Polluted Surface Waters in Bangladesh using Open Science Workflows

Bénilde Bonnefille[1], Oskar Karlsson[1], May Britt Rian[1], Rubhana Raqib[2], Faruque Parvez[3], Stefano Papazian[1], M. Sirajul Islam[4], and Jonathan W. Martin[1]*

[1] Department of Environmental Science, Exposure and Effects Unit, Science for Life Laboratory, Stockholm University, Stockholm, 106 91, Sweden

[2] Immunobiology, Nutrition and Toxicology Unit, Infectious Diseases Division, International Centre for Diarrhoeal Disease Research, Bangladesh (icddr,b), Dhaka, 1212, Bangladesh

[3] Department of Environmental Health Sciences, Mailman School of Public Health, Columbia University, New York, NY, 10032, USA

[4] Laboratory of Food Safety and One Health, Laboratory Sciences and Services Division, International Centre for Diarrhoeal Disease Research, Bangladesh (icddr,b), Dhaka, 1212, Bangladesh

Corresponding author: Jonathan W. Martin; Email: jon.martin@aces.su.se

Summary: this Supporting Information document (SI) consists of 138 pages and contains 53 tables and 60 figures.

All datasets have been deposited as mzXML files at Mass Spectrometry Interactive User Environment (MassIVE) database with the following identifications.

APCI+	MSV000089703
APCI-	MSV000089704
ESI+	MSV000089705
ESI-	MSV000089706

The Venn diagram R script is available here: https://doi.org/10.6084/m9.figshare.21493695

Table of content

SI-1. NTA Study Reporting Tool S6

SI-2. Sampling sites information S10

SI-3. Material and Methods S10

SI-3.1. Chemicals and solvents S10

SI-3.2. MS additional information S13

SI-3.3. Data processing S13

SI-3.3.1. MS-DIAL parameters (version 4.60) S13

SI-3.3.2. Multivariate statistical analysis S15

SI-3.3.3. Precursors and MS2 data provided to MASST S16

SI-3.3.4. Chemical formula and structure prediction S18

SI-3.4. Semi-quantification S18

SI-4. Results S19

SI-4.1. Data quality S19

SI-4.1.1. Labelled standards' detection and areas before and after normalization in the different samples S19

SI-4.1.2. PCA model before and after internal standard normalization S21

SI-4.2. Unsupervised and supervised multivariate analyses S24

SI-4.2.1. PCA loading plots for each ionization mode S24

SI-4.2.2. PLS model validation S25

SI-4.2.3. OPLS-DA model and related results S26

SI-4.3. Level 1 identifications S28

SI-4.3.1. 1-and 2-naphthalenesulfonic acid S28

SI-4.3.2. 2-hydroxyatrazine S30

SI-4.3.3. 4- and 5-methyl-1H-benzotriazole S32

SI-4.3.4. Acesulfame S34
SI-4.3.5. Aspirin S36
SI-4.3.6. Atenolol S38
SI-4.3.7. Atrazine S41
SI-4.3.8. Bis(2-ethylhexyl) phosphate S43
SI-4.3.9. Caffeine S45
SI-4.3.10. Carbamazepine S47
SI-4.3.11. Carbendazim S49
SI-4.3.12. Chlorpyrifos S51
SI-4.3.13. Clarithromycin S53
SI-4.3.14. Cotinine S55
SI-4.3.15. Daidzein S57
SI-4.3.16. Diazinon S59
SI-4.3.17. Diclofenac S61
SI-4.3.18. Dimethoate S63
SI-4.3.19. Diphenyl phosphate S65
SI-4.3.20. Diuron S67
SI-4.3.21. Erythromycin S69
SI-4.3.22. Fluconazole S71
SI-4.3.23. Imidacloprid S73
SI-4.3.24. Losartan S75
SI-4.3.25. Malathion S78
SI-4.3.26. Metformin S80
SI-4.3.27. Nicotine S82
SI-4.3.28. Oxybenzone S84

SI-4.3.29. Panthenol S86
SI-4.3.30. Paracetamol S88
SI-4.3.31. Propylparaben S90
SI-4.3.32. Quinoline S92
SI-4.3.33. Salbutamol S94
SI-4.3.34. Sucralose S96
SI-4.3.35. Sulfamethazine S98
SI-4.3.36. Sulfamethoxazole S100
SI-4.3.37. Triclosan S102
SI-4.3.38. Trimethoprim S105
SI-4.3.39. Tris(2-butoxyethyl) phosphate S107
SI-4.4. Level 2 annotations and there MS/MS spectrum match S109
SI-4.4.1. Pharmaceuticals S109
SI-4.4.2. Personnal care products S111
SI-4.4.3. Industrial compounds S112
SI-4.4.4. Miscellaneous S114
SI-4.5. Annotated compounds' correlation with sampling sites and detection trends S116
SI-4.6. Retention time index model S123
SI-4.6.1. Calibration curves obtained for samples injections S123
Positive ionization mode S123
Negative ionization mode S124
SI-4.6.2. Comparison between exprimental and predicted RTI S126
SI-4.6.3. Calibration curves obtained for annotations confirmation S126
Positive ionization mode S126
Negative ionization mode S128

SI-4.6.4. Comparison between the RTIs between non-spiked and spiked samples for Level 1 confirmations S132

SI-4.7. Further investigation of detected compounds S133

SI-4.7.1. Correlation between compounds S133

SI-4.7.2. Chemical formula prediction S134

SI-4.7.3. Semi-quantification results S136

References S137

SI-1. NTA Study Reporting Tool

Score	Description	Example 1 – Analytical Sequence	Example 2 – Statistical & Chemometric Analysis	Example 3 – Data Processing & Analysis QA/QC
0	**No elements of relevant reporting are present.**	No details are provided regarding analysis order and batch information. Based on reporting, the experiment could not be replicated.	Statistical analyses were performed, but no information was provided about software, methods, assumptions, and thresholds.	No details are provided about the quality, boundary, accuracy, and precision of the data processing and analysis method(s). Based on reporting, the overall performance of the processing and analysis method(s) is unclear.
1	**Some elements of relevant reporting are present, but major improvements are needed**	Some details are provided regarding analysis order and batch information, but major details that would assist in the interpretation of the results are lacking (e.g., the authors reported that blanks were analyzed, but no information about frequency/injection or sample order within the acquisition sequence was provided).	Statistical analyses were performed, but gaps in method details limit method reproducibility.	Some details are provided about the quality, boundary, accuracy, and precision of the data processing and analysis method(s), but major gaps limit understanding of overall performance (e.g., impacts of method choices on observed chemical space are discussed, but true/false positive rates, workflow QC checks, and reproducibility of identification are not reported).
2	**Most elements of relevant reporting are present, but minor improvements are needed.**	The majority of analysis order and batch information is provided, but some details are missing that could assist interpretation of the results (e.g., analysis order was reported, including sample randomization and the frequency/order of external reference standards and method blanks; however, information about multiple analytical batches was unclear).	Statistical analyses were performed, and the majority of method details are provided (e.g., the authors clearly reported the software, methods, and assumptions, but minor details regarding method thresholds are missing that would improve the reader's ability to interpret the results).	Detailed information is provided about the quality, boundary, accuracy, and precision of the data processing and analysis method(s), but minor details that could improve the reader's ability to interpret the impact on results are excluded (e.g., the authors do not discuss possible sources of variability in the outcomes of their identification workflow).
3	**All elements of relevant reporting are present.**	All details regarding sample randomization, replicate injections, the inclusion of blanks and QC samples in the acquisition sequence, and information about analytical batches are provided so that the experiment could be reproduced by an outside researcher.	Statistical analyses were performed, and all method details are reported in sufficient detail to allow a reader to reproduce the method and clearly understand the impact of method choices on the analysis results.	All necessary detailed information about the quality, boundary, accuracy, and precision of the data processing and analysis method(s) are provided and the implications are discussed.
NA	**Reporting not relevant to the study.**	Analytical sequence and batch information should be reported for all studies; however, it is less critical for studies that do not rely on comparisons of measurement data across samples.	Statistical analyses were not performed (e.g., the study is solely focused on chemical annotation & identification).	Reporting the quality, boundary, accuracy, and precision of the data processing & analysis methods is informative for all studies.

Category		Sub-Category	Example Information to Report	Score (use drop-down menu)<br>NA 0 1 2 3	Rationale for score
Methods	Study Design	Objectives & Scope	• Study goals and hypotheses<br>• Scope of the study with respect to use of NTA / suspect screening<br>• Expected chemical coverage of approach and potential limitations	3	
		Sample Information & Preparation	• Sample collection/replication, handling/storage, preparation, extraction, & clean-up methods (and related QA practices)<br>• Intended use of samples (e.g., method development, compound identification, etc.)<br>• Development and intended use of blanks	3	All the information about sample preparation is given in the manuscript, information about standards and labelled standards is given in supplementary information (SI).<br>The intended use of the samples is given in the study goals.<br>Field blanks were prepared as described in the manuscript and treated as normal samples.
		QC Spikes & Samples	• Development of QC spikes/samples (e.g., isotopically labeled standards/spikes, native standard spikes, matrix pools)<br>• Intended use of QC spikes/samples (e.g., to monitor instrument performance, data normalization, etc.)	3	
	Data Acquisition	Analytical Sequence	• Sample randomization and use of replicate injections<br>• Inclusion of blanks and QC samples in the acquisition sequence<br>• Information about single vs. multiple analytical batches	3	No replicate injections of the samples themselves were performed, but we had duplicate samples (triplicate for field blanks), and a QC (pool of samples) that was injected repeatedly all along the analytical sequences
		Chromatography	• Instrument specifications<br>• Method settings (e.g., column/guard, mobile phases, gradient, injection techniques)	3	LC information is provided in material & method in the manuscript, and more details are provided in supplementary data.
		Mass Spectrometry	• Instrument specifications<br>• Instrument calibration and/or tuning procedures<br>• Method settings (e.g., acquisition parameters, such as polarity, resolution, data-dependent vs. data-independent)	3	MS information is provided in material & method in the manuscript, and more details are provided in supplementary data.

Category	Sub-Category	Example Information to Report	Score (use drop-down menu) NA 0 1 2 3	Rationale for score
Data Processing & Analysis	Data Processing	• File conversion information (e.g., to open-source format, centroiding)<br>• Software program(s) used<br>• Workflow steps (e.g., peak picking, RT calibration, alignment, gap filling) and settings<br>• Feature detection thresholds (e.g., replicate detection criteria; min height, area, or S/N levels; comparison to occurrence/abundance in blanks)<br>• Data correction or normalization methods (e.g., peak area/height normalization or scaling, blank subtraction)	**3**	We used MS-DIAL for the data processing. No conversion of the file was needed: MS-DIAL is able to handle most of the proprietary format. The workflow steps are described in the material & method part of the manuscript, and the parameters are available in SI.<br>The S/N level is given as an MS-DIAL parameter, data correction and normalization methods are described in the material & method paragraph.
	Statistical & Chemometric Analysis	• Software programs(s)/package(s) used & samples/sample groups to which analyses were applied<br>• Basic statistical analysis method goals (e.g., summarize data, evaluate variability, hypothesis testing), type (e.g., Wilcoxon rank sum test, Chi-square test), assumptions, and settings/thresholds<br>• Chemometric analysis method goals (e.g., prioritize features, compare/classify samples, evaluate relationships between features), type (e.g., differential analysis, hierarchical clustering, dimensionality reduction), assumptions, and settings/thresholds	**3**	The software programs used are reported in the manuscript material & method.<br>PCA and PLS were performed on the samples, with details about the samples included in the manuscript
	Annotation & Identification	• Software program(s) used (or description of manual annotation/identification efforts)<br>• Libraries and databases used (including details such as chemical coverage, resolution, metadata inclusion; information about in-house databases)<br>• Workflow steps (e.g., formula assignment, suspect screening, MS/MS spectral interpretation or library matching)<br>• Workflow methods & settings (e.g., formula prediction method, scoring algorithms; mass error/RT tolerances, accepted match scores)	**3**	The parameters used in MS-DIAL to perform annotations (as well as the library used) are given in the material & method § 2.3, and in SI, in the MS-DIAL parameters table.<br>More information is available in § 2.5 about confirming the annotations.
Results	Data Outputs	Statistical & Chemometric Outputs	• Basic statistical outputs (e.g., adj. p-values, standard deviations, test statistics)<br>• Results of chemometric analyses (e.g., reported classifications/groupings of features or samples, observed trends in the data)<br>• Visuals/plots (e.g., Venn diagrams, heatmaps, clustering dendrograms, volcano plots, network diagrams, PCA and loading plots)<br>• New statistical metrics, algorithms, packages, and/or scripts	**3**	Various visuals/plots are provided in the results, including a Venn diagram, a PCA score plot and its related loading plot, and a PLS score plot and its related biplot. More visuals/plots are provided in SI to support the data provided in the manuscript.<br>The script used for the Venn diagram is provided in SI.

Category	Sub-Category	Example Information to Report	Score (use drop-down menu)<br>NA 0 1 2 3	Rationale for score
QA/QC Metrics	Identification & Confidence Levels	• Reported identifications and associated confidence levels (e.g., levels described by Schymanski et al., *ES&T*, 2014)<br>• Supporting data for annotation/identification (e.g., formula match scores, fine isotope pattern, retention time match, MS/MS match scores, source of MS/MS spectra)<br>• For features with lower confidence IDs, (i.e., not standard-confirmed), proposed tentative structures and other annotated data<br>• Semi-quantification or quantification data<br>• Exported MS/MS spectra (e.g., as a library, database, or deposition into online repository)	**3**	Reported identifications and annotations and their associated confidence levels are provided in a table in the results.<br>MS/MS match data (samples vs library) are provided in SI, retention time prediction was performed (see material & method paragraph, as well as the results).<br>No semi-quantification or quantification was performed in this study (not the purpose).
	Data Acquisition QA/QC	• Quality: Adherence to QA/QC protocols for sample preparation and data acquisition<br>• Boundary: Description of the potential impacts of methods (sample prep, chromatographic, MS) on observable chemical space<br>• Accuracy: Reported chromatographic and mass accuracy<br>• Precision: Variability of observed retention time, precursor mass error, and abundance	**3**	Labelled standards retention times shifts were evaluated during the injections considering we had 4 batches of samples (see § 3.1), as well as their area variation for each ionization mode before and after normalization (see § 3.1 and SI).<br>The chemical coverage space is discussed in § 3.2.
	Data Processing & Analysis QA/QC	• Quality: Outcomes of QC checks along the data processing & analysis workflow<br>• Boundary: Impact of data processing & analysis method(s) on observed chemical space, observed limits of detection/ID<br>• Accuracy: Performance measures (True Positive Rate, False Positive Rate, etc.) for known compounds or samples with known classification<br>• Precision: Reproducibility/repeatability of performance measures for known compounds or samples with known classification; Calculations such as False Discovery Rate, F1 score, etc.	**3**	Both labelled standards information from the samples and QC injections were considered before to further process the data (§3.1)<br>The chemical coverage space is discussed in § 3.2.<br>Mass accuracy information is available for the annotation in a table in the results.

SI-2. Sampling sites information and map creation

Table S1. Additional information about the sampling sites including the river, their names, coordinates, and sampling date

Sampling site number	River	Name of the site	Latitude	Longitude	Sampling date	pH
M1	Meghna	Aruail	24°09'10.3''N	90°59'13.1''E	31-dec-19	7.4
M2	Meghna	Bhairab	24°02'20.8''N	90°59'18.6''E	31-dec-19	7.31
M3	Meghna	Bishnanadi ferry ghat	23°45'42.1''N	90°43'43.6''E	21-Jan-20	7.70
M4	Meghna	Gazaria	23°32'38.1''N	90°34'51.9''E	21-Jan-20	7.37
M5	Meghna	Chandpur Puranghat	23°13.3150'N	90°38.1530'E	04-feb-20	6.89
S1	Shitalakshya	Akij Match Factory	23°34'17.3''N	90°31'53.8''E	19-feb-20	7.52
B1	Buriganga	Gabtoly Bridge	23°47'03.7''N	90°20'05.9''E	09-mar-20	7.22
B2	Buriganga	Mitford Ghat	23°42'36.5''N	90°23'57.7''E	26-feb-20	7.33
B3	Buriganga	Molla Salt Factory	23°37'57.6''N	90°27'42.3''E	26-feb-20	7.32
T1	Turag	Kodda Bridge	23°59'46.3"N	90°20'50.6"E	19-feb-20	7.27
T2	Turag	Ashulia Bridge	23°53'47.0"N	90°20'03.7"E	09-mar-20	7.51
T3	Turag	Sinnirtek Landing	23°47'58.1''N	90°20'32.2''E	09-mar-20	7.43

A map of the sampling sites created with QGIS (v3.28) is provided in Figure 2 of the main paper. The background layer, the boundary layer and the water layers used were publicly available from OpenStreetMap[4] (© OpenStreetMap contributors, CC-BY-SA).

SI-3. Material and Methods

SI-3.1. Chemicals and solvents

LC-MS grade methanol (Optima Grade, Fisher Scientific), LC-MS grade water (Optima Grade, Fisher Scientific), and ammonium fluoride (99.9% purity, Honeywell Fluka) were used as LC-MS eluents. Detailed information for all the standards and labelled standards used in this study, including CAS number, purity and manufacturer, is reported in the tables hereafter.

Table S2. Labelled standards used as surrogate standards (S) or internal standards (IS) during the study, and their related information *

Name		CAS number	Purity (%)	Manufacturer
2,4-Dichlorophenoxyacetic Acid-13C6	(S)	150907-52-1	98.85	Toronto Research Chemicals
Acephate-d3	(S)	2140327-70-2	99.70	Sigma-Aldrich
Atrazine-2-hydroxy-d5	(IS)	1276197-25-1	99.00	A2S certified
Atrazine-d5	(S)	163165-75-1	99.70	Sigma-Aldrich
Bisphenol A-13C12	(S)	263261-65-0	98.00	Toronto Research Chemicals
Carbamazepine-d10	(S)	132183-78-9	99.20	Cerilliant

Name		CAS number	Purity (%)	Manufacturer
Carbendazim-d4	(S)	291765-95-2	97.80	Sigma-Aldrich
DEET-d10	(S)	291759-05-2	98.00	Toronto Research Chemicals
Diclofenac-d4	(S)	1215576-01-4	98.00	Santa Cruz Biotechnology
Diphenyl Phosphate-d10	(S)	93952-11-5	95.00	Toronto Research Chemicals
Diuron-d6	(IS)	153466-65-0	99.00	Santa Cruz Biotechnology
Metformin-d6	(S)	1185166-01-1	99.60	Sigma-Aldrich
Nor Harmane-d7	(S)	1219806-03-7	98.00	Toronto Research Chemicals
Oxybenzone-d3 (S)	(S)	No CAS number	98.00	Toronto Research Chemicals
Pentachlorophenol-13C6	(S)	85380-74-1	98.00	Toronto Research Chemicals
Perfluoro-n-[13C4]-butanoic acid	(S)	Bought as a mix: no CAS number		Wellington laboratories
Perfluoro-n-[13C5]-pentanoic acid	(S)			Wellington laboratories
Perfluoro-n-[1,2,3,4,6-13C5]-hexanoic acid	(S)			Wellington laboratories
Perfluoro-n-[1,2,3,4-13C4]-heptanoic acid	(S)			Wellington laboratories
Perfluoro-n-[13C8]-octanoic acid	(S)			Wellington laboratories
Perfluoro-n-[13C9]-nonanoic acid	(S)			Wellington laboratories
Perfluoro-n-[1,2,3,4,5,6-13C6]-decanoic acid	(S)			Wellington laboratories
Perfluoro-n-[1,2,3,4,5,6,7-13C7]-undecanoic acid	(S)			Wellington laboratories
Perfluoro-n-[1,2-13C2]-dodecanoic acid	(S)			Wellington laboratories
Perfluoro-n-[1,2-13C2]-tetradecanoic acid	(S)			Wellington laboratories
Perfluoro-1-[2,3,4-13C3]-butanesulfonate	(S)			Wellington laboratories
Perfluoro-1-[1,2,3-13C3]-hexanesulfonate	(S)			Wellington laboratories
Perfluoro-1-[13C8]-octanesulfonate	(S)			Wellington laboratories
Triclosan-13C6	(S)	No CAS number	99.22	Toronto Research Chemicals

* All the labelled standards were spiked before sample preparation (surrogate standards), except atrazine-2-hydroxy-d5 and diuron-d6 that were spiked before injection (internal standards).

Table S3. Standards used for identity confirmation and their related information

Name	CAS number	Purity (%)	Manufacturer
1-Naphthalenesulfonic acid	85-47-2	99.60	Chemtronica
2-Hydroxyatrazine	2163-68-0	99.00	Sigma-Aldrich
2-Naphthalenesulfonic acid	76530-12-6	99.90	TCI
4-Methyl-1H-benzotriazole	29878-31-7	93.80	Merck
5-Methyl-1H-benzotriazole	136-85-6	100.00	Merck
Acesulfame	55589-62-3	98.70	TCI
Aspirin (acetylsalicylic acid)	50-78-2	99.90	Sigma-Aldrich
Atenolol	29122-68-7	98.00	Acros Organics
Atrazine	1912-24-9	99.50	Sigma-Aldrich
Bis(2-ethylhexyl) phosphate	298-07-7	100.00	Merck
Caffeine	58-08-2	100.00	Santa Cruz Biotechnology
Carbamazepine	298-46-4	99.80	Santa Cruz Biotechnology
Carbendazim	10605-21-7	99.00	Honeywell
Chlorpyrifos	2921-88-2	99.00	Sigma-Aldrich
Clarithromycin	85721-33-1	97.60	Acros Organics
Cotinine	486-56-6	99.90	Toronto Research Chemicals
Daidzein	486-66-8	98.00	Toronto Research Chemicals
Diazinon	333-41-5	98.60	Honeywell
Diclofenac	15307-79-6	100.00	Sigma-Aldrich
Dimethoate	60-51-5	99.90	Honeywell
Diphenyl phosphate	32586-82-6	99.56	Toronto Research Chemicals
Diuron	330-54-1	99.10	Sigma-Aldrich
Erythromycin	114-07-8	97.20	MP Biomedicals
Fluconazole	86386-73-4	99.30	Santa Cruz Biotechnology
Indigo blue	482-89-3	100.00	Merck
Imidacloprid	138261-41-3	99.90	Honeywell
Losartan	124750-99-8	99.90	Merck
Malathion	121-75-5	99.20	Sigma-Aldrich
Memantine	41100-52-1	98.00	Combi-blocks
Metformin	1115-70-4	99.40	Santa Cruz Biotechnology
Nicotine	54-11-5	100.00	Sigma-Aldrich
Oxybenzone	131-57-7	98.00	Toronto Research Chemicals
Panthenol	81-13-0	100.00	Merck
Paracetamol	103-90-2	100.00	Sigma-Aldrich
Propylparaben	94-13-3	99.40	Toronto Research Chemicals
Quinoline	91-22-5	98.00	Alfa Aesar
Salbutamol	18559-94-9	100.00	European Pharmacopeia Reference Standard
Sucralose	56038-13-2	98.79	De Ehrenstorfer
Sulfamethazine	57-68-1	99.30	Acros Organics

Name	CAS number	Purity (%)	Manufacturer
Sulfamethoxazole	723-46-6	99.90	Santa Cruz Biotechnology
Triclosan	3380-34-5	98.00	Toronto Research Chemicals
Trimethoprim	738-70-5	99.80	MP Biomedicals
Tri-N-butyl phosphate	126-73-8	100.00	Merck
Tris(2-butoxyethyl) phosphate	78-51-3	98.10	Acros Organics

SI-3.2. MS additional information

The mass spectrometer was calibrated before each batch of samples injections for the relevant ionization mode. Before APCI analysis, the calibrations were performed in ESI before switching to the APCI probe.

Full MS analyses were performed with an AGC target of 10^6, a maximum injection time of 50 ms. DIA analyses were performed using combination of 20 and 70 eV normalized collision energy. DDA analyses were performed with a resolution of 30,000 (FWHM), an AGC target of 10^5, a maximum injection time of 50 ms, an isolation window of 0.5 m/z, and a combination of 20 and 70 eV normalized collision energy.

SI-3.3. Data processing

SI-3.3.1. MS-DIAL parameters (version 4.60)

See table next page

Table S4. MS-DIAL parameters applied for the data pre-processing

Step	Value
Data Collection	MS1 mass range begin: 90
	MS1 mass range end: 1050
	MS/MS mass range begin: 50
	MS/MS mass range end: 1050
	Processing threads: 12
Mass accuracy	MS1 tolerance: 0.001 Da
	MS2 tolerance: 0.003 Da
Maximum charged number	2
Consider Cl and Br elements	TRUE
Execute retention time corrections	FALSE
Minimum peak height	50,000
Mass slice width	0.07 Da
Smoothing method	Linear weighted moving average
Smoothing level	3
Minimum peak width	8
Exclusion mass list	No
Sigma window value	1
Abundance cut off	0
Exclude after precursor ion	TRUE
Keep isotopic ions until	5 Da
Keep isotopic ions w/o MS2Dec	TRUE
MSP file (library), used for initial screening	MS-DIAL library (v.15)
Accurate mass tolerance (MS1):	0.002 Da
Accurate mass tolerance (MS2):	0.005 Da
Identification score cut off:	80%
Use retention time for:	Scoring: false, filtering: false
Abundance cut off	0%
Adduct ion setting	**ESI+**: [M+H]+, [M+NH4]+, [M+Na]+, [2M+H]+, [2M+NH4]+, [M+2H]2+, [M+3H]3+, [M]+<br><br>**ESI-**: [M-H]-, [M+Cl]-, [2M-H]-, [M-2H]2-, [M-3H]3-, [M+F]-
Reference File	Sample T2_2 (most complex one for the 4 datasets)
Retention time tolerance	0.2 min
MS1 tolerance	0.002 Da
Retention time factor	0.5
MS1 factor	0.5
Peak count filter	0%
N% detected in at least one group	0%
Remove features based on blank information	TRUE
Sample max/blank average	10-fold change
Keep "reference matched" metabolite features	FALSE
Keep "suggested (w/o MS2)" metabolite features	FALSE
Keep removable features and assign the tag	TRUE
Gap filling by compulsion	TRUE
Data Export/Post processing	
Peak area tables for modelling, profiling	
Exported table used for building data sets for modelling	Raw Data Matrix (area)
Order of steps	Export -> normalization by internal standards -> Blank Subtraction
Blank subtraction	Average of field-blanks, negative values set to 0

SI-3.3.2. Multivariate statistical analysis

A principal component analysis (PCA) was performed before and after internal standard normalization for each dataset to evaluate if QC-sample normalization should be considered in case of sensitivity loss along the analytical sequence (§SI 3.1.3). After internal standard normalization and blank subtraction of samples, the QCs and field blanks were removed for further data processing and statistical analysis.

SI-3.3.3. Precursors and MS2 data provided to MASST

Table S5. Precursor and MS2 data of identified compounds provided to MASST

**Class**	**Industrial compound**	**Miscellaneous**	**Personal care product**	**Pesticide**	**Pharmaceutical**
**Compound**	**Bis(2-ethylhexyl) phosphate**	**Daidzein**	**Oxybenzone**	**Carbendazim**	**Cotinine**
**Ionization mode**	**ESI-**	**ESI+**	**ESI+**	**ESI+**	**ESI+**
**Precursor**	321.2202	255.0649	229.0864	192.0767	177.1021
**Fragments**<br>**(mass, Da; intensity)**	62.9641, 7155282<br>78.95919, 655108224<br>100.9679, 10035704<br>209.09512, 42501676<br>321.21884, 171520768<br>322.22235, 31899982<br>325.18588, 12497174	119.04878, 1056401<br>128.06161, 714545<br>129.06976, 1266744<br>131.08533, 561254<br>133.02844, 872864<br>137.02327, 9702990<br>138.02663, 722387<br>143.08559, 779342<br>145.02837, 1686087<br>153.07002, 1910439<br>157.0648, 1567218<br>171.07993, 1605596<br>181.06517, 3893566<br>182.06732, 444995<br>197.0592, 381832<br>199.07507, 11759070<br>200.07883, 1720801<br>209.05917, 563136<br>212.14043, 383063<br>221.15312, 724789<br>227.06937, 4428898<br>228.07368, 681012<br>237.05508, 1952329<br>255.06549, 32402564<br>256.06793, 5901520<br>257.0715, 608588	53.03888, 259303<br>55.05464, 394679<br>59.04953, 488514<br>65.03879, 170285<br>67.05447, 153821<br>69.03365, 173286<br>69.07003, 1758397<br>70.07351, 125398<br>77.04114, 121797<br>91.05935, 186062<br>95.05366, 1974080<br>96.05689, 152199<br>99.04737, 142538<br>102.00297, 139782<br>105.03498, 3878582<br>105.04594, 247267<br>111.11702, 1100956<br>133.10056, 239628<br>135.04427, 164944<br>137.06021, 117554<br>140.00644, 115885<br>143.03407, 456768<br>149.02373, 659517<br>149.05991, 744962<br>149.07944, 143764<br>151.03922, 11550647<br>152.04257, 725673<br>172.1696, 619979<br>189.16451, 309165<br>195.12218, 121321<br>207.1748, 369374<br>217.15939, 137074<br>229.08667, 4687812<br>230.08965, 665463<br>232.10535, 148371	92.05448, 50409020<br>105.04599, 39177820<br>132.05522, 273997376<br>133.06386, 33574084<br>135.05534, 42176500<br>160.05048, 2175623168<br>161.05411, 186303312<br>192.07652, 459820288<br>193.07982, 42315124	70.065, 6065166<br>80.05439, 115168912<br>81.06023, 6240682<br>98.06312, 31362740<br>99.06649, 1528792<br>118.06512, 3110031<br>119.05995, 11513813<br>120.08058, 1912565<br>146.05949, 3554120<br>177.10254, 50558696<br>178.10526, 5118215

Table S6. Precursor and MS2 data of unknown features provided to MASST

**Ionization mode**	**ESI-**	**APCI-**	**ESI-**	**ESI+**	**ESI+**	**APCI+**	**ESI-**	**ESI+**	**ESI+**	**ESI-**
**Retention time**	**2.73**	**2.72**	**7.38**	**5.75**	**6.04**	**6.19**	**18.09**	**2.87**	**6.18**	**19.34**
**Precursor**	**96.9603**	**96.9602**	**186.1140**	**202.0530**	**202.0530**	**184.0430**	**297.1530**	**128.9540**	**202.0530**	**325.1840**
**MASST matches**	**0**	**0**	**3**	**0**	**0**	**0**	**0**	**0**	**0**	**11**
**Fragments (mass, Da; intensity)**	63.96244, 63704184<br>79.9575, 2656981504<br>81.95352, 123905528<br>96.96033, 1616088960<br>98.95592, 72923320	63.96259, 50968168<br>79.95738, 2397978112<br>81.95343, 111388912<br>95.95254, 55578116<br>96.9602, 1451393664<br>98.95631, 71084528<br>100.96777, 28444184	52.65165, 286334<br>75.96229, 468894<br>97.06609, 885954<br>98.97238, 389349<br>99.92598, 282913<br>102.96454, 633316<br>123.08198, 1681974<br>124.11284, 713853<br>125.09763, 8414492<br>126.10094, 2021666<br>142.1203, 380829<br>142.12395, 255878<br>143.56998, 271612<br>156.9249, 1300750<br>160.84274, 902628<br>168.10355, 1135906<br>186.11346, 20690972<br>187.11765, 1705171	53.03889, 2192130<br>55.05449, 2060301<br>56.04982, 4189239<br>56.96514, 7063440<br>58.06547, 2753022<br>58.96075, 35549744<br>65.03885, 6845368<br>68.9971, 5207834<br>74.93312, 4526448<br>80.05441, 2587462<br>92.05455, 17374796<br>93.06172, 78451168<br>94.06495, 4912327<br>94.06936, 2904783<br>95.05322, 3558452<br>96.04859, 2320218<br>100.07893, 26192986<br>100.11552, 2166117<br>101.02562, 5554414<br>104.05202, 2432991<br>108.04561, 14054054<br>110.06007, 3732189<br>119.03496, 4278600<br>120.0806, 18208844<br>121.07638, 2669892<br>132.9288, 2995491<br>134.92403, 4497136<br>136.06209, 12502877<br>136.91988, 20090014<br>138.09111, 7188652<br>138.95309, 3248667<br>139.0025, 2207526<br>143.0341, 9203454<br>154.93054, 5482130<br>156.01089, 9888091<br>156.96312, 2157004<br>158.15437, 3774043<br>175.06046, 2856194<br>202.05295, 25962348<br>203.05605, 2270494	53.0389, 824581<br>65.03883, 3136401<br>92.05453, 10357848<br>93.02026, 1136645<br>93.06172, 81877888<br>94.06497, 4947354<br>94.06938, 2759347<br>96.04859, 918776<br>100.07892, 2161330<br>102.09376, 1111383<br>103.05633, 1107170<br>104.05203, 2714340<br>108.04561, 10628060<br>109.04813, 875869<br>110.06005, 1758333<br>118.0654, 1346559<br>120.08061, 22075414<br>121.07648, 5813316<br>121.08412, 1545595<br>134.9241, 1040352<br>136.91991, 1903200<br>138.09113, 6465959<br>140.01627, 1279508<br>156.01093, 6957424<br>158.00699, 894950<br>166.08612, 1080650<br>202.05305, 75151136<br>203.05617, 6421086<br>204.04924, 3068716	50.01556, 4114556<br>51.02332, 5385365<br>53.00252, 2484620<br>53.03895, 5662091<br>54.03428, 2453429<br>55.05454, 4647072<br>56.0499, 5457822<br>63.02323, 3421900<br>65.03866, 22259602<br>66.04649, 10841152<br>67.05433, 2589830<br>70.06511, 1991667<br>77.04093, 2785987<br>78.03853, 2257961<br>79.05915, 4424080<br>80.05453, 8653442<br>81.07723, 2639252<br>82.07276, 2923378<br>89.04485, 3622890<br>91.05949, 5596330<br>92.05467, 13322819<br>93.0614, 131669400<br>94.06515, 8645666<br>94.06956, 3601468<br>95.05343, 6137164<br>96.04864, 2685348<br>100.07908, 7398717<br>105.04619, 2647471<br>107.05061, 2937373<br>107.0869, 2904849<br>108.04575, 8094186<br>110.06023, 9393919<br>120.08079, 8724166<br>136.06161, 3327910<br>140.01653, 2514061<br>146.05991, 5603358<br>156.01122, 6822640<br>162.05548, 8799332<br>184.04327, 3691701	59.96742, 234432<br>76.97039, 140068<br>78.93284, 149709<br>79.96555, 263100<br>80.96273, 271329<br>82.97723, 152220<br>85.00815, 143161<br>93.96164, 273482<br>99.92618, 435041<br>116.92995, 157617<br>116.94759, 173165<br>118.92452, 634319<br>119.92485, 154141<br>122.96974, 704313<br>132.95892, 198252<br>133.06581, 126132<br>146.86949, 246043<br>155.14441, 237553<br>160.8427, 212920<br>162.83958, 173347<br>172.9539, 146510<br>178.85887, 126626<br>181.83833, 229953<br>211.17165, 252173<br>297.1528, 11716309<br>298.15601, 2030864<br>299.14789, 336911	53.03893, 425659<br>55.01808, 356585<br>55.05449, 437152<br>56.04983, 318360<br>57.03378, 867648<br>58.06547, 2577704<br>58.96076, 1626609<br>59.07322, 538130<br>61.03981, 333211<br>62.98185, 295820<br>64.97753, 19015732<br>68.99713, 3557355<br>69.03375, 356771<br>70.0653, 448843<br>74.93308, 325039<br>79.05897, 305795<br>80.05443, 591758<br>80.95534, 465320<br>80.95886, 1484218<br>84.05108, 871741<br>88.98432, 610928<br>96.04858, 432233<br>100.07899, 328580<br>100.95097, 386034<br>101.02567, 4475109<br>106.96671, 2448428<br>108.04564, 1192633<br>110.06005, 297042<br>111.00776, 990792<br>114.09142, 1179568<br>118.08609, 4709313<br>119.03421, 1920418<br>124.0394, 698204<br>125.02349, 387496<br>126.05498, 1041961<br>127.03936, 320050<br>128.95338, 843005<br>129.01849, 600442	65.03879, 20748514<br>66.04661, 5108330<br>80.05432, 4730334<br>92.05494, 66876724<br>93.06165, 322927264<br>94.06496, 20428408<br>94.06936, 10549342<br>97.07947, 14524900<br>100.07937, 41348012<br>103.05682, 5152339<br>104.05198, 7587086<br>108.04551, 44273012<br>116.04947, 4223494<br>118.06525, 4506108<br>120.08117, 65758528<br>121.07634, 10512632<br>121.08404, 5538080<br>126.09135, 4137870<br>138.09183, 28311914<br>156.01173, 34876160<br>172.16937, 3362950<br>178.0616, 5299454<br>202.05272, 36067112<br>203.05743, 3379779	79.95755, 155908528<br>81.95357, 5167886<br>119.0507, 280317472<br>120.054, 22624200<br>170.00404, 12288239<br>183.01262, 152343280<br>184.01643, 11382342<br>185.00755, 7010408<br>195.01263, 8965020<br>325.18335, 451989504<br>326.18655, 89525040<br>327.18121, 16782778

SI-3.3.4. Chemical formula and structure prediction

MS-FINDER (v.3.52)[5] and SIRIUS (v5.6.2)[6] software were used for chemical formula assignment with a mass accuracy tolerance of 3 ppm. SIRIUS generates molecular formulae for the MS1 and ranks the candidates based on their isotopic pattern and MS2 fragmentation to rank the candidates[6], while MS-FINDER uses both MS1 and MS2 to generate molecular formula, ranks these, and predicts structure considering, among others, hydrogen rearrangements rules.[5] Only the first-candidate assigned by MS-FINDER that had a match in the top 3 candidates generated by SIRIUS were retained as an unambiguous formula. Only the top score associated structures predicted by MS-FINDER are reported here.

SI-3.4. Semi-quantification

The semi-quantification was performed using the non-spiked and spiked samples that were reinjected for confirmation purpose based on a single-point standard addition. The concentrations in the samples that were not reinjected during the confirmation step were extrapolated by simple scaling.

SI-4. Results

SI-4.1. Data quality

SI-4.1.1. Labelled standards' detection and areas before and after normalization in the different samples

Some labelled standards were not detected in a few samples. This was the case for triclosan-13C6, perfluoro-n-[1,2-13C2]-dodecanoic acid, and perfluoro-n-[1,2-13C2]-tetradecanoic acid in APCI-, and metformin-d6 in both APCI+ and ESI+. For each of these labelled standards 3 samples, all different for the different standards, were concerned. These labelled standards were therefore not considered for normalization for each mode concerned. An evaluation of labelled standards' areas in the different samples was performed before and after normalization to evaluate if the normalization performed as expected before continuing with the data processing. The normalization step allowed correcting for any variation due to matrix effects, recovery and injection between samples. An example of this evaluation is available in Fig. SI-1, showing that the variability between the labelled standards' areas in the different samples is reduced after normalization, allowing a better comparison of the samples.

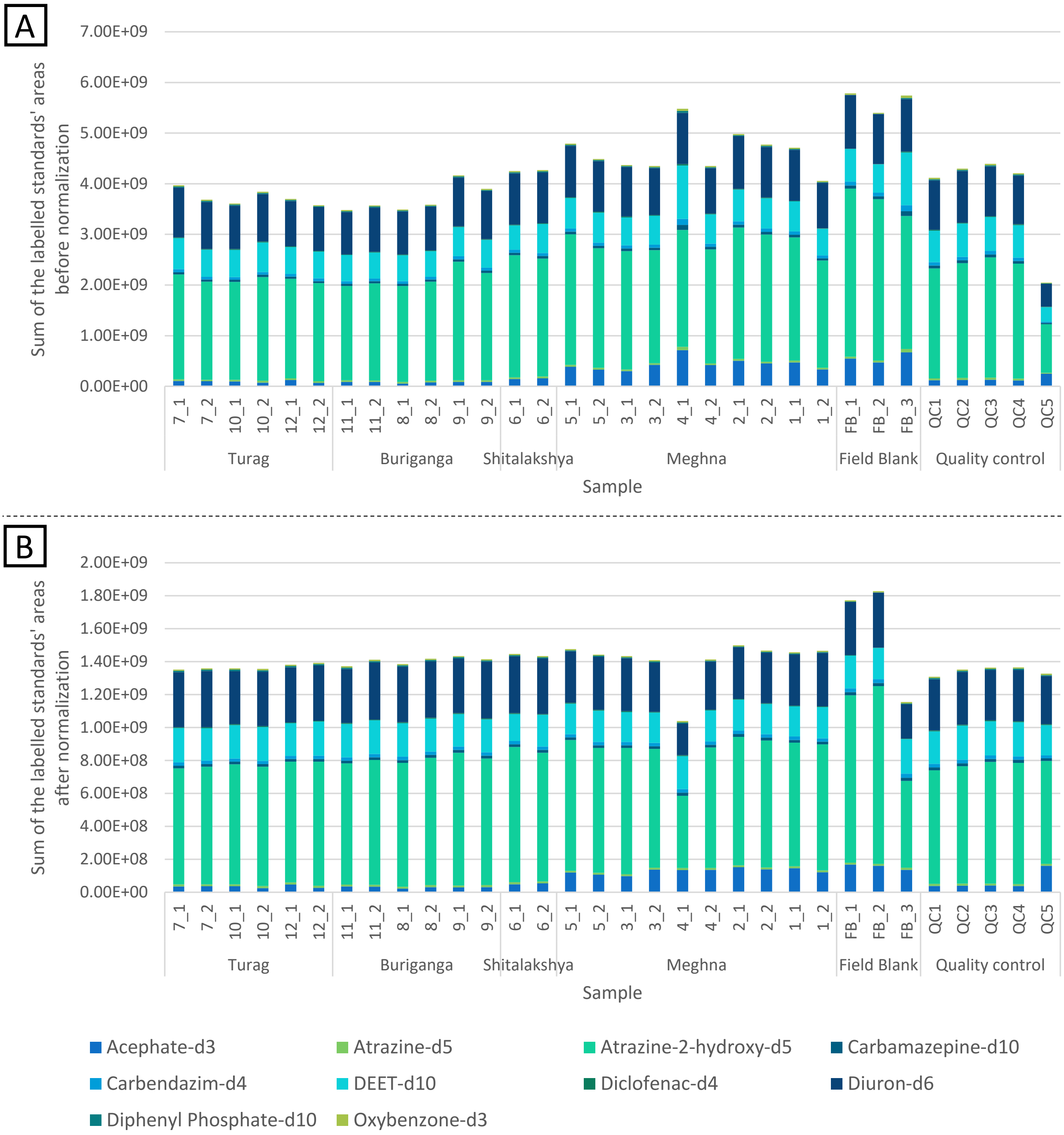

Figure S1. Impact of the normalization on the sum of the labelled standards' areas in each sample (A) before and (B) after it was performed for ESI+ data

SI-4.1.2. PCA model before and after internal standard normalization

Among QCs, the last one (QC5) was not grouped with the others for all ionization modes except APCI+ for which QC4 was the one not grouped with the others. Despite QC5 seems to be different from the others, considering the samples injections were randomised and the samples distributions on the PCA plots was similar for all ionization mode, we considered that no QC normalization was necessary. The APCI+ PCA score plot before normalization revealed one sample (T1_1) was an outlier (Fig. SI-2-B1a). A raw file inspection revealed the sample was not injected properly, and that IS normalization could not help to normalize the data. To not disturb the data processing, feature areas from sample T1_1 in APCI+ were set as "NA".

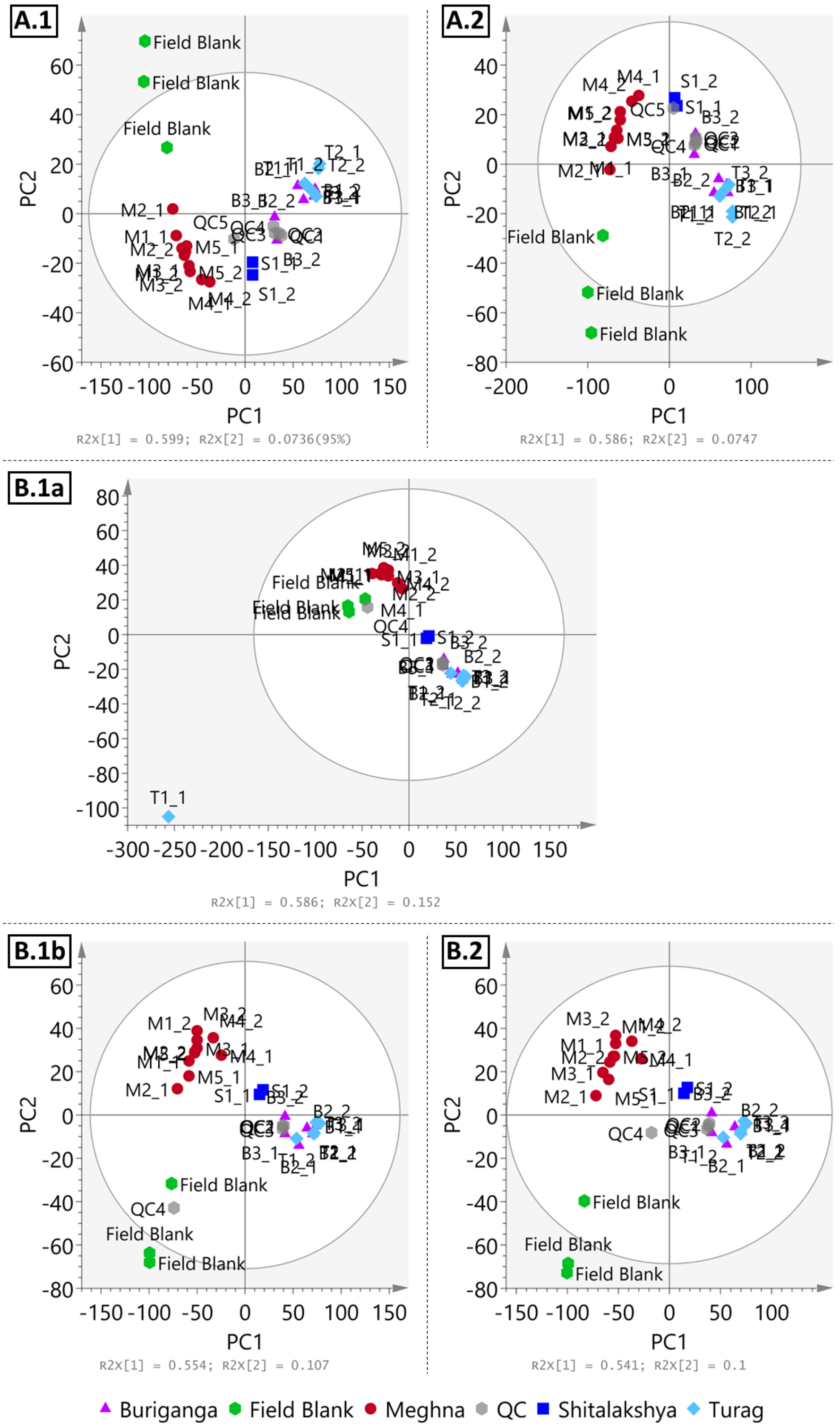

Figure S2. PCA score plots of the data acquired with the different ionization modes (A: APCI-, B: APCI+, C: ESI-, D: ESI+) (X.1) before and (X.2) after internal standard normalization. For APCI+, 2 score plots are shown before IS normalization, one (B.1a) before and one (B.1b) after discarding the outlier sample T1_1.

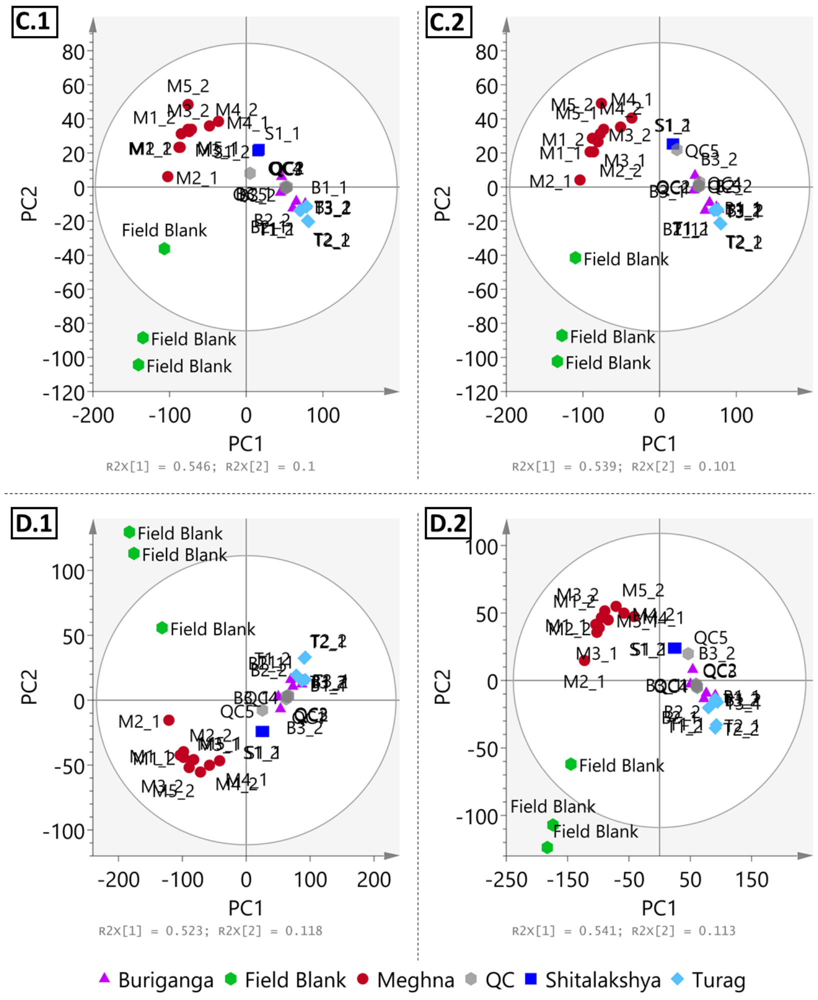

Figure S2. Continued

SI-4.2. Unsupervised and supervised multivariate analyses

SI-4.2.1. PCA loading plots for each ionization mode

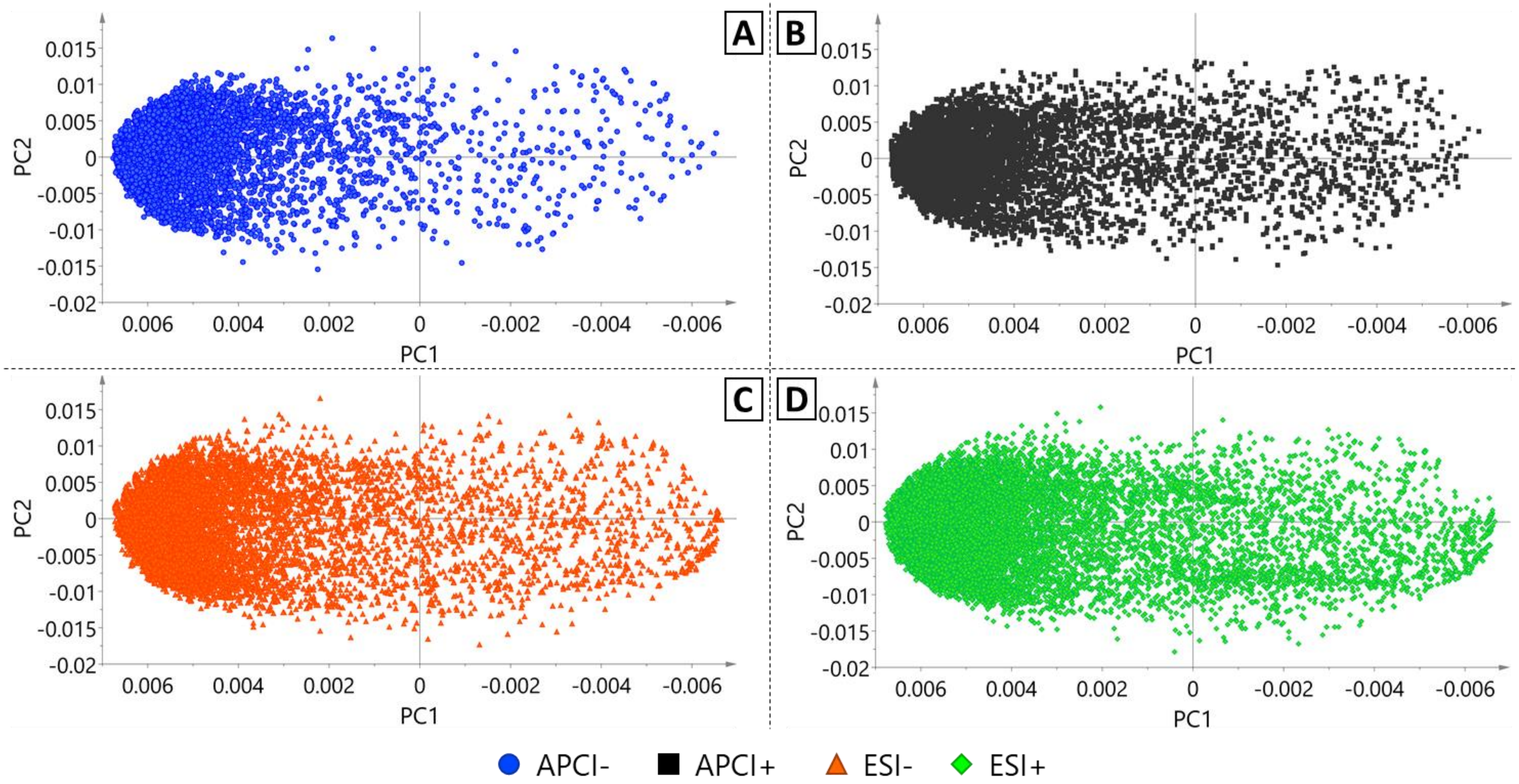

Figure S3. Surface water samples features projected on loading plots depending on their ionization mode: (A) APCI- (n = 6452), (B) APCI+ (n = 6776), (C) ESI- (n = 10,395), and (D) ESI+ (n = 15,402). Note, the x-axis directionality is inverted in all the loading plots.

SI-4.2.2. PLS model validation

Two permutation tests were performed to evaluate PLS model overfitting for both Y vectors, corresponding to the latitude and the longitude of the sampling points, respectively. One hundred random permutations were performed to compare R2 and Q2 (on the top right of each plot, Figure S4) of the original model, and R2 and Q2 of each permutation model carried out. The low values of intercepts for both R2Y and Q2, for both latitude and longitude coordinates, show that the model has high statistical significance (no over-fitting).

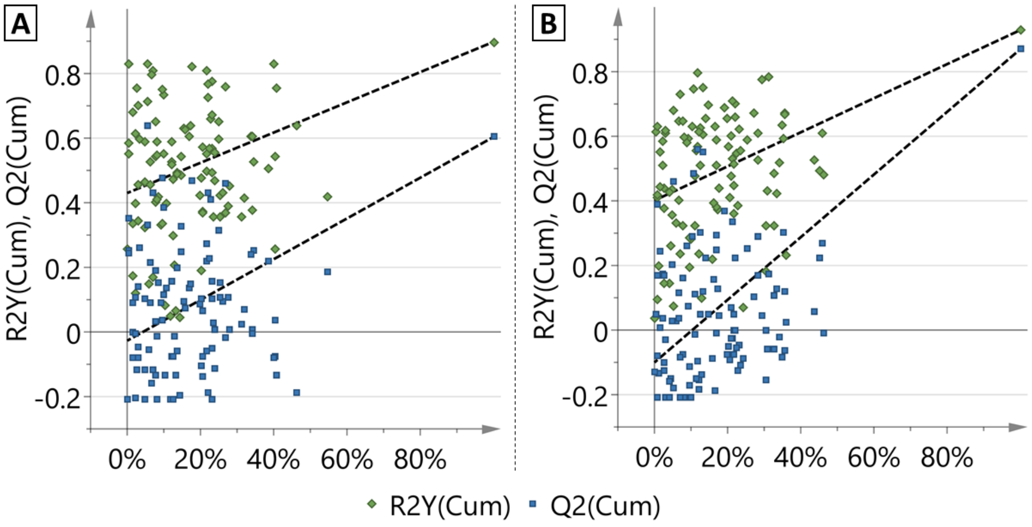

Figure S4. PLS permutation plot (n = 100 permutations) representing the correlation (x axis) between the original and permuted (A) "latitude" Y-vector and (B) "longitude" Y-vector. The green diamond is R2Y(Cum), which is the explained variance of the model. The blue square is Q2(Cum), standing for the predictive ability of the model. The values of R2(Cum) and Q2(Cum) are reported on the y axis.

SI-4.2.3. OPLS-DA model and related results

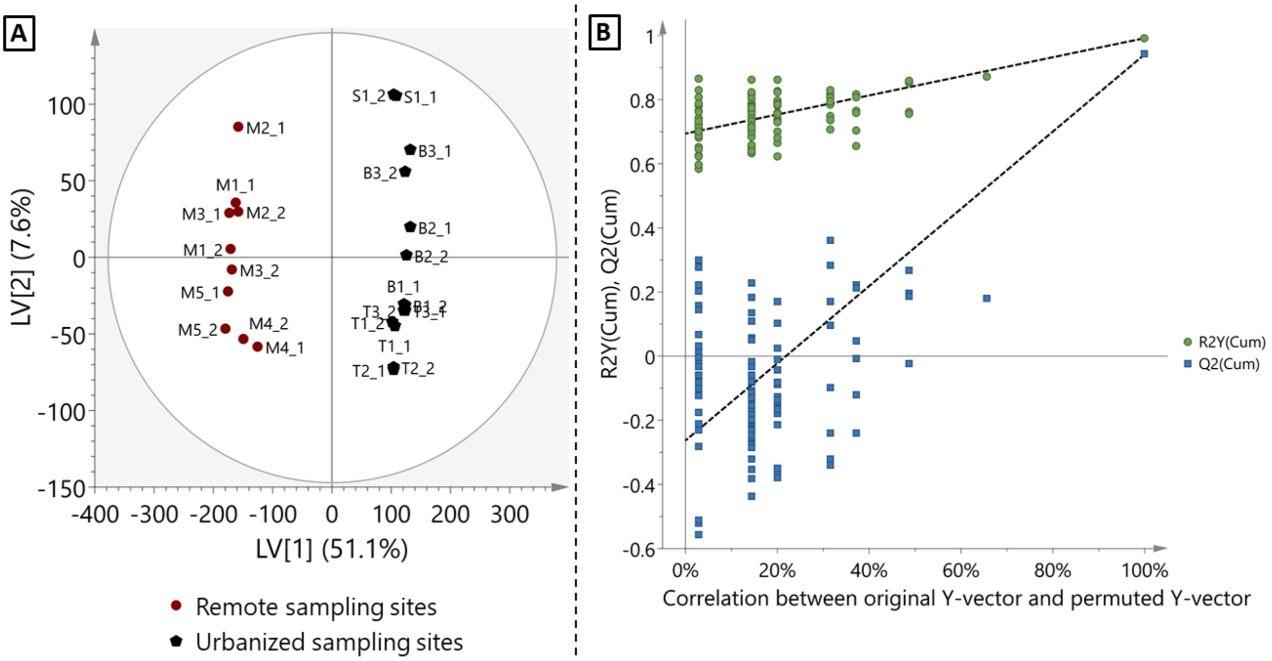

Figure S5. A) Score of the OPLS-DA model (1+1+0) explaining the fingerprint profiles of urbanized (T1-T3, B1-B3, and S1) and rural (M1-M5) sampling sites. Samples are clustered based on similarity of fingerprint profiles. CV-ANOVA p-value 2.03E-11. B) OPLS-DA permutation plot (n = 100 permutations). The green circle is R2Y(Cum), which is the explained variance of the model. The blue square is Q2(Cum), standing for the predictive ability of the model. The intercepts are 0.69 for R2(Cum) and -0.26 for Q2(Cum).

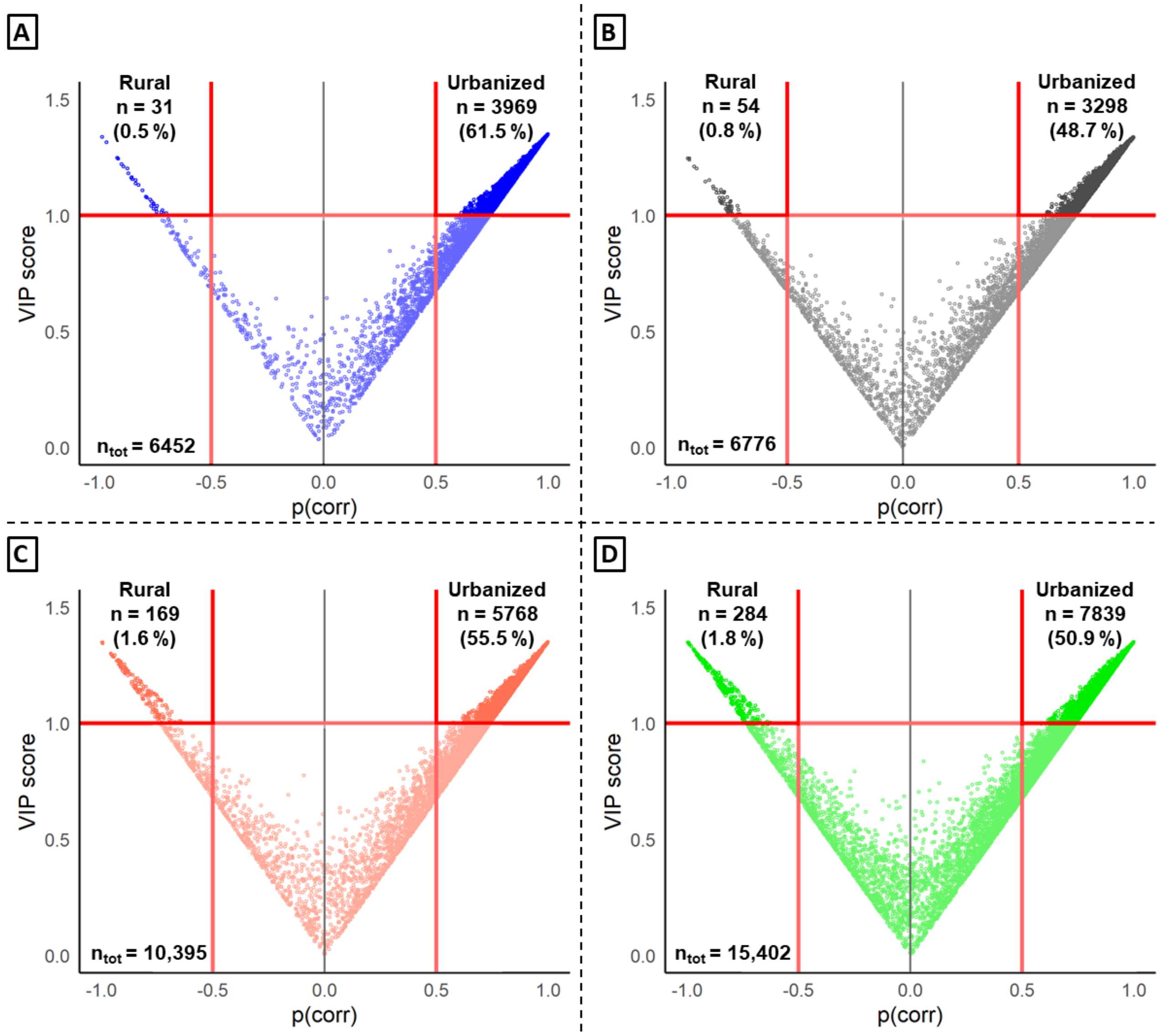

Figure S6. Contribution of all the features (reported as n_{tot} on the figure) to the rural or urbanized sampling sites highlighted in a plot for each ionization mode with A) APCI-, B) APCI+, C) ESI-, D) ESI+. Features with the highest model-correlation coefficients (p(corr) < 0.5 and p(corr) > 0.5) and the highest VIP score (VIP > 1) are features highly correlated to the geographical situation of the samples (rural vs urbanized sampling sites).

SI-4.3. Level 1 identifications

SI-4.3.1. 1-and 2-naphthalenesulfonic acid

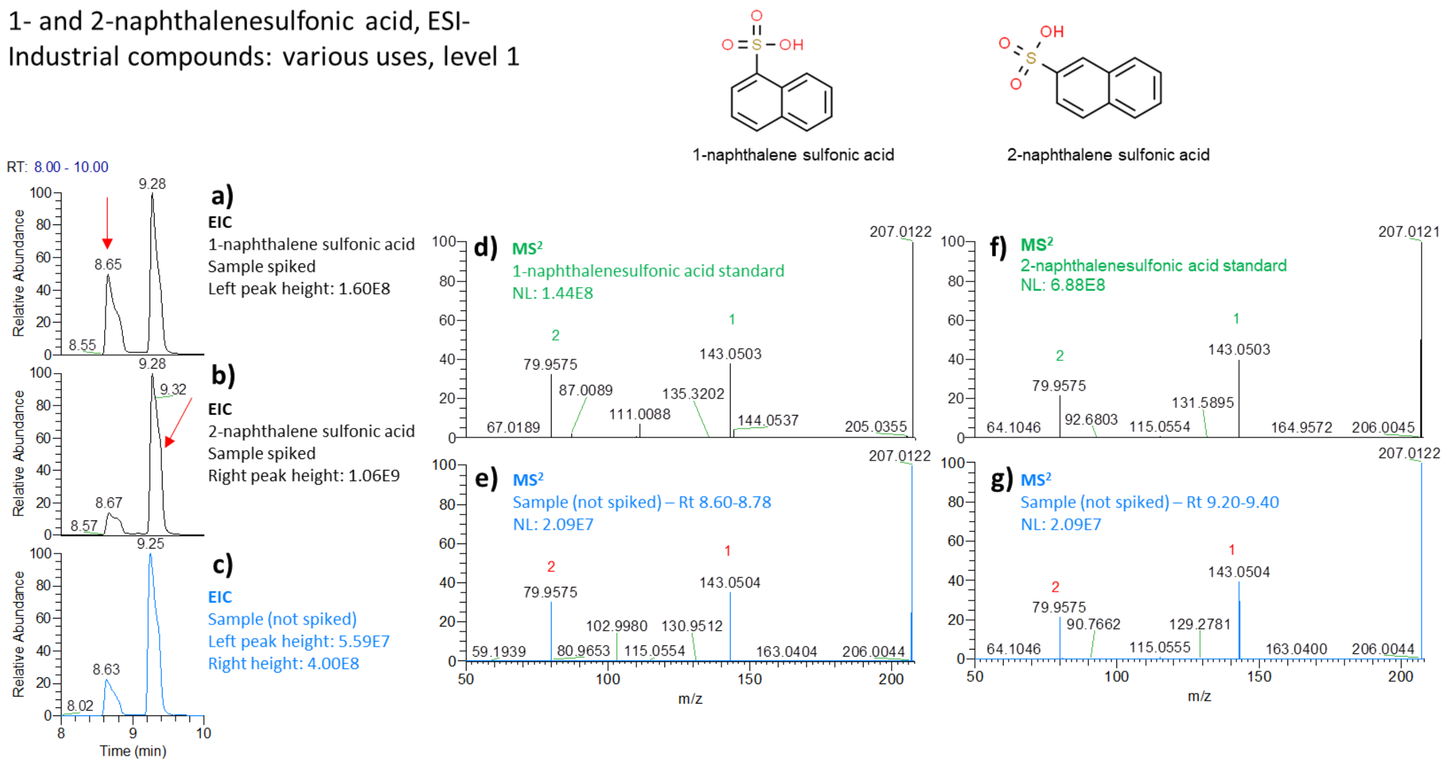

Figure S7. Confirmed identification of 1-and 2-naphthalenesulfonic acid. Comparison of extracted ion chromatogram (EIC) between a surface water sample a) spiked with 1-naphthalenesulfonic acid at 5 µg/L, b) spiked with 2-naphthalenesulfonic acid at 5 µg/L, and c) not spiked. MS² data from d) an authentic 1-naphthalenesulfonic acid standard and e) a surface water sample not spiked at the corresponding retention time, f) an authentic 2-naphthalenesulfonic acid standard and g) a surface water sample not spiked at the corresponding retention time, analysed under the same conditions. Mass deviation and references to the literature related to the fragments flagged with numbers are available in Table S7. NL: normalized level.

Table S7. 1- and 2-naphthalene sulfonic fragments expected chemical formula, theoretical and measured masses, and related mass deviation, from the DDA MS2 of the 1- and 2-naphthalene sulfonic standard and sample reinjected for confirmation

	Fragment	(Expected) Chemical formula	Theoretical mass	Measured mass for the standard	Mass error (standard) (ppm)	Measured mass for the sample	Mass error (sample) (ppm)	Reference
	Parent	[C10H8O3S-H]-	207.0121	207.0122	+0.48	207.0122	+0.48	
1-Naphthalenesulfonic acid	1	[C10H7O]-	143.0502	143.0503	+0.70	143.0504	+1.40	[7] MassBank EU Record: LU075553
	2	[SO3]-	79.9574	79.9575	+1.25	79.9575	+1.25	[7] MassBank EU Record: LU075553
	Parent	[C10H8O3S-H]-	207.0121	207.0121	0.00	207.0122	+0.48	
2-Naphthalenesulfonic acid	1	[C10H7O]-	143.0502	143.0503	+0.70	143.0504	+1.40	[7] MassBank EU Record: EA065361
				115.0554		115.0555		
	2	[SO3]-	79.9574	79.9575	+1.25	79.9575	+1.25	[7] MassBank EU Record: EA065361

SI-4.3.2. 2-hydroxyatrazine

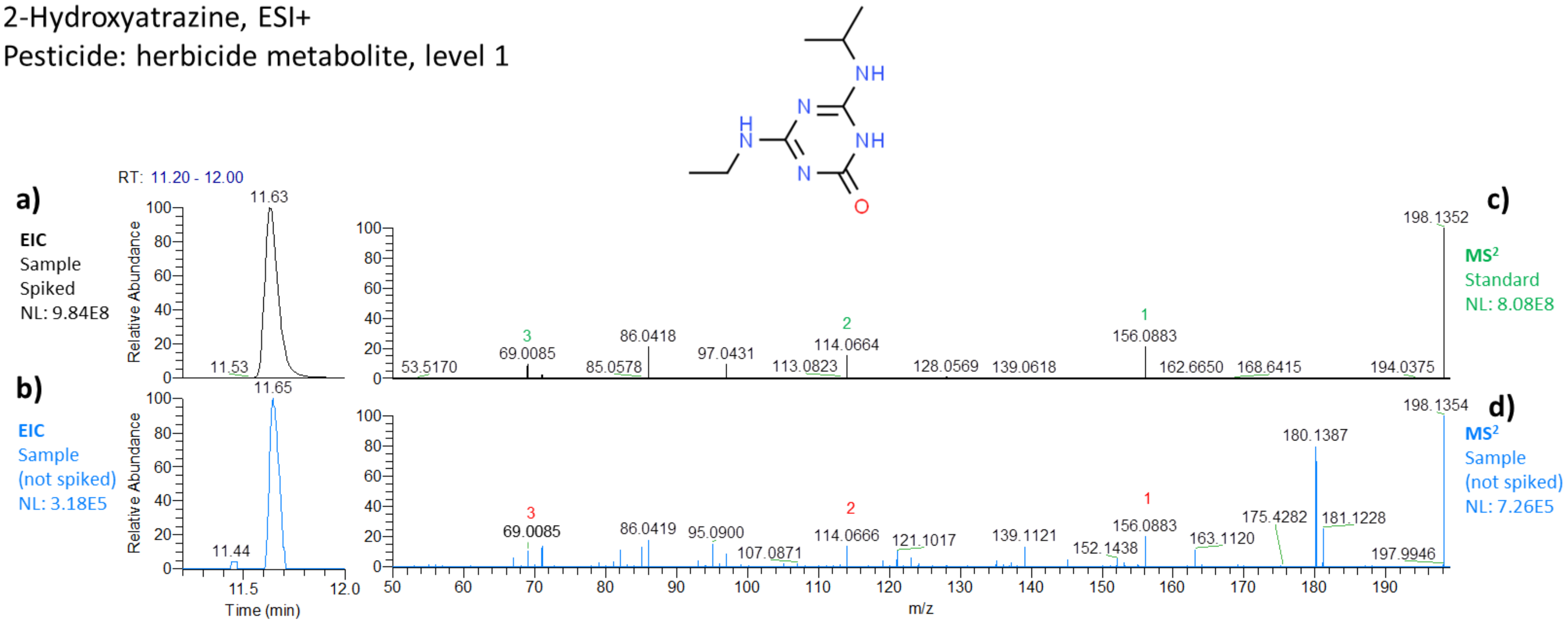

Figure S8. Confirmed identification of 2-hydroxyatrazine. Comparison of extracted ion chromatogram (EIC) between a surface water sample a) spiked with 2-hydroxyatrazine at 5 µg/L and b) not spiked, and of MS^2 data from c) authentic 2-hydroxyatrazine standard and d) a surface water sample not spiked, analysed under the same conditions. Mass deviation and references to the literature related to the fragments flagged with numbers are available in Table S8. NL: normalized level.

Table S8. 2-Hydroxyatrazine fragments expected chemical formula, theoretical and measured masses, and related mass deviation, from the DDA MS2 of the standard and sample reinjected for confirmation

Fragment	(Expected) Chemical formula	Theoretical mass	Measured mass for the standard	Mass error (standard) (ppm)	Measured mass for the sample	Mass error (sample) (ppm)	Reference
Parent	$[C_8H_{15}N_5O+H]^+$	198.1349	198.1352	+1.51	198.1354	+2.52	
1	$[C_5H_{10}N_5O]^+$	156.0880	156.0883	+1.92	156.0883	+1.92	[8] MassBank EU Record: EA027910
2	$[C_4H_8N_3O]^+$	114.0662	114.0664	+1.75	114.0666	+3.51	[8] MassBank EU Record: EA027910
			86.0418		86.0419		
3	$[C_2HN_2O]^+$	69.0083	69.0085	+2.90	69.0085	+2.90	MassBank EU Record: EA027910

SI-4.3.3. 4- and 5-methyl-1H-benzotriazole

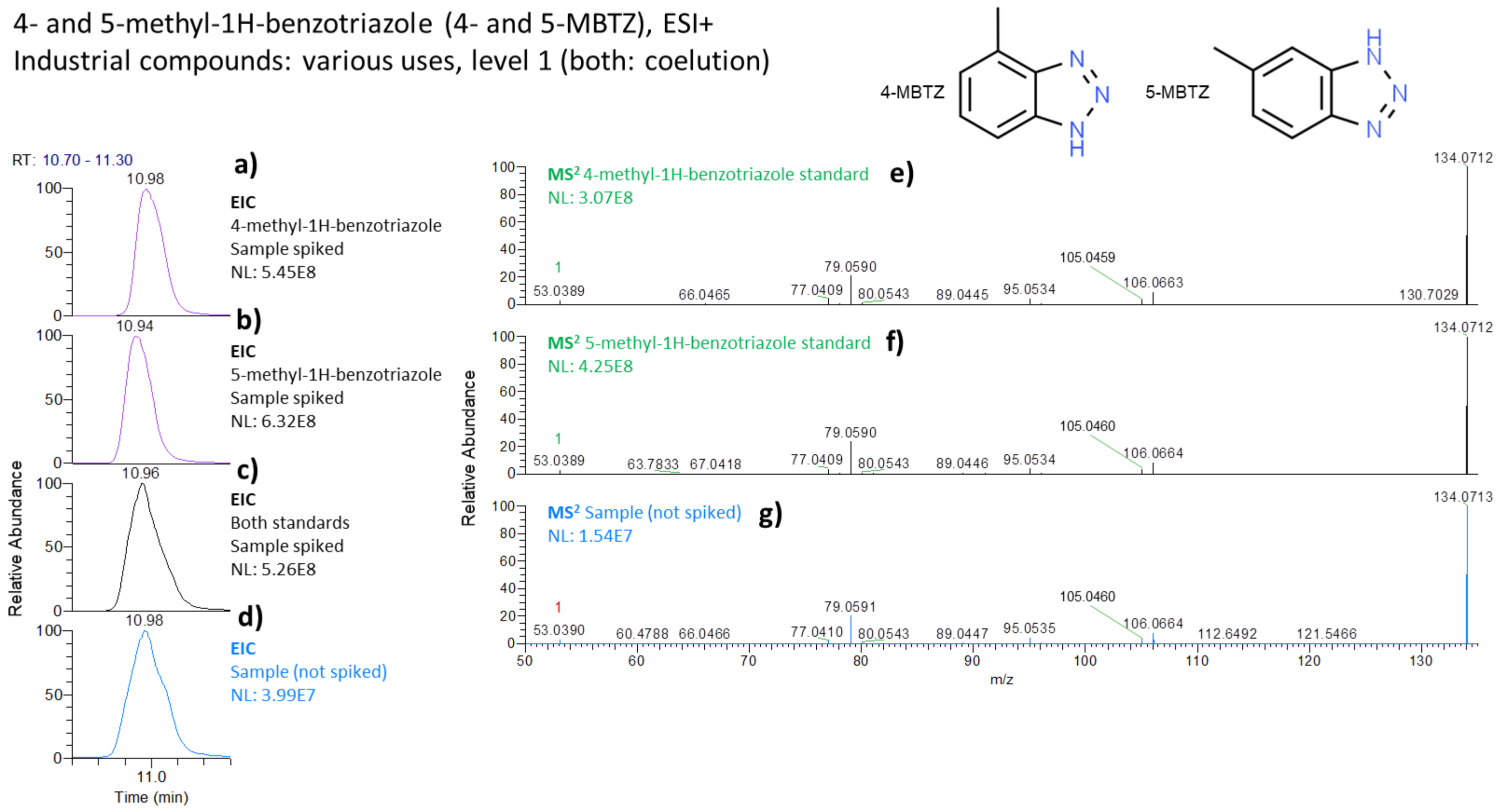

Figure S9. Confirmed identification of 4- and 5-methyl-1H-benzotriazole. Comparison of extracted ion chromatogram (EIC) between a surface water sample a) spiked with 4-methyl-1H-benzotriazole at 5 µg/L, b) spiked with 5-methyl-1H-benzotriazole at 5 µg/L, c) spiked with both spiked with 4-and 5-methyl-1H-benzotriazole at 5 µg/L, and d) not spiked, and of MS2 data from c) authentic 4-methyl-1H-benzotriazole standard, d) authentic 5-methyl-1H-benzotriazole standard and e) a surface water sample not spiked, analysed under the same conditions. Mass deviation and references to the literature related to the fragments flagged with numbers are available in Table S9. NL: normalized level.

Table S9. 4- and 5-methyl-1H-benzotriazole fragments expected chemical formula, theoretical and measured masses, and related mass deviation, from the DDA MS2 of the standard and sample reinjected for confirmation

Fragment	(Expected) Chemical formula	Theoretical mass	Measured mass for 4-MBTZ	Mass error (4-MBTZ) (ppm)	Measured mass for 5-MBTZ	Mass error (5-MBTZ) (ppm)	Measured mass for the sample	Mass error (sample) (ppm)	Reference
Parent	[C7H7N3+H]+	134.0713	134.0712	-0.75	134.0712	-0.75	134.0713	0.00	
			106.0663		106.0664		106.0664		
			105.0459		105.0460		105.0460		
			95.0534		95.0534		95.0535		
			89.0445		89.0446		89.0447		
			80.0543		80.0543		80.0543		
			79.0590		79.0590		79.0591		
			77.0409		77.0409		77.0410		
			66.0465				66.0466		
1	[C4H5]+	53.0386	53.0389	+5.66	53.0389	+5.66	53.0390	+7.54	MassBank EU Records: ETS00118, EA016712

SI-4.3.4. Acesulfame

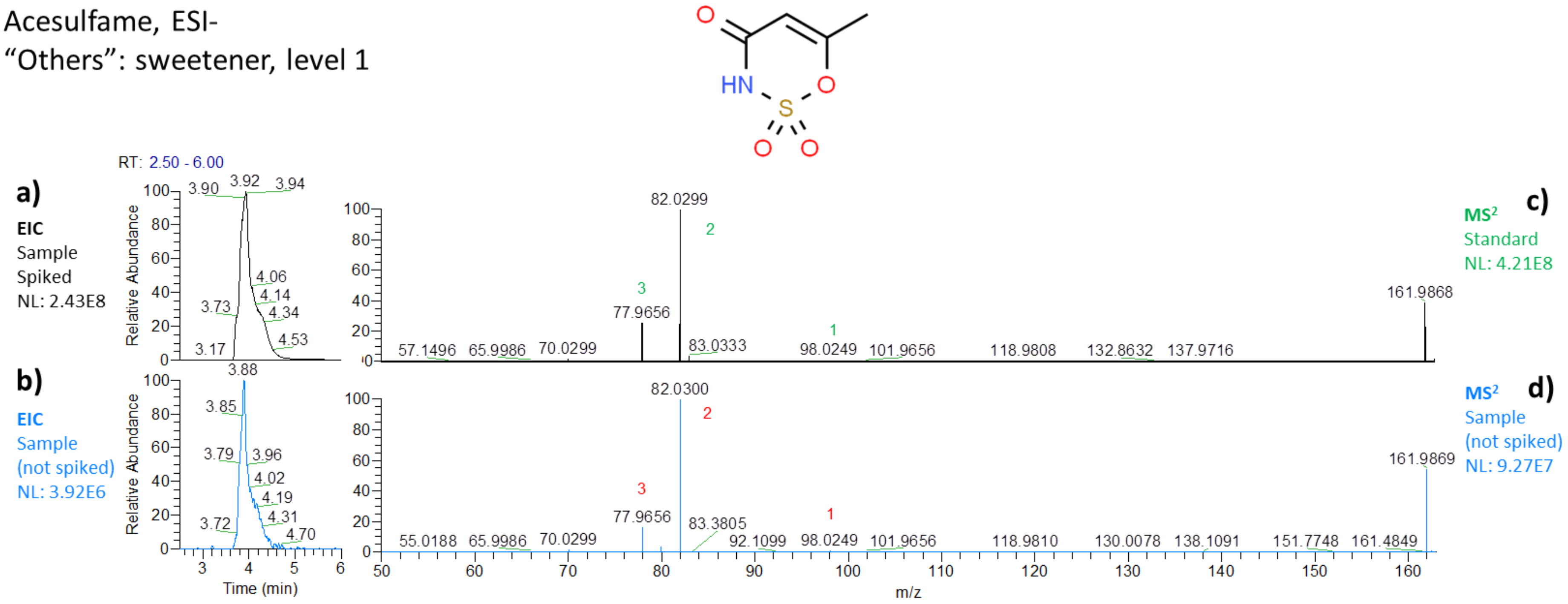

Figure S10. Confirmed identification of acesulfame. Comparison of extracted ion chromatogram (EIC) between a surface water sample a) spiked with acesulfame at 5 µg/L and b) not spiked, and of MS2 data from c) authentic acesulfame standard and d) a surface water sample not spiked, analysed under the same conditions. Mass deviation and references to the literature related to the fragments flagged with numbers are available in Table S10. NL: normalized level.

Table S10. Acesulfame fragments expected chemical formula, theoretical and measured masses, and related mass deviation, from the DDA MS2 of the standard and sample reinjected for confirmation

Fragment	(Expected) Chemical formula	Theoretical mass	Measured mass for the standard	Mass error (standard) (ppm)	Measured mass for the sample	Mass error (sample) (ppm)	Reference
Parent	[C4H5NO4S-H]-	161.9867	161.9868	+0.67	161.9869	+1.23	
			118.9808		118.9810		
1	[C4H4NO2]-	98.0248	98.0249	+1.02	98.0249	+1.02	MassBank EU Record: EA275660 [9,10]
2	[C4H4NO]-	82.0298	82.0299	+1.22	82.0300	+2.44	MassBank EU Record: EA275660 [9,10]
3	[NO2S]-	77.9655	77.9656	+1.28	77.9656	+1.28	MassBank EU Record: EA275660
			70.0299		70.0299		
			65.9986		65.9986		

SI-4.3.5. Aspirin

Aspirin, ESI+

Pharmaceutical: analgesic, level 1

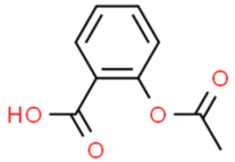

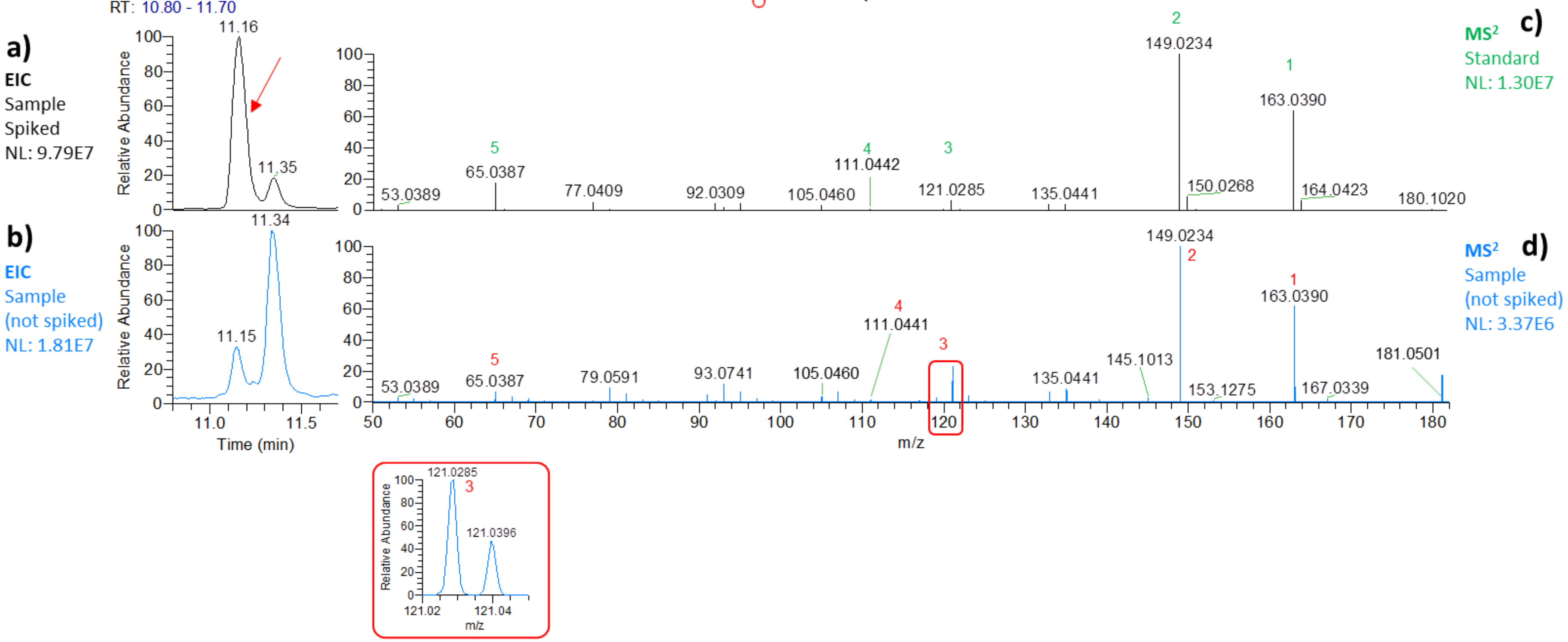

Figure S11. Confirmed identification of aspirin. Comparison of extracted ion chromatogram (EIC) between a surface water sample a) spiked with aspirin at 5 µg/L and b) not spiked, and of MS2 data from c) authentic aspirin standard and d) a surface water sample not spiked, analysed under the same conditions. Mass deviation and references to the literature related to the fragments flagged with numbers are available in Table S11. NL: normalized level.

Table S11. Aspirin fragments expected chemical formula, theoretical and measured masses, and related mass deviation, from the DDA MS2 of the standard and sample reinjected for confirmation

Fragment	(Expected) Chemical formula	Theoretical mass	Measured mass for the standard	Mass error (standard) (ppm)	Measured mass for the sample	Mass error (sample) (ppm)	Reference
Parent	[C9H8O4+H]+	181.0495	Not detected in the DDA	NA	181.0501	+3.31	
1	[C9H7O3]+	163.0390	163.0390	0.00	163.0390	0.00	[11] MassBank EU Record: EQ357803
2	[C8H5O3]+	149.0233	149.0234	0.00	149.0234	0.00	MassBank EU Record: EQ357803
3	[C7H5O2]+	121.0284	121.0285	+0.83	121.0285	+0.83	[11] MassBank EU Record: EQ357803
4	[C6H7O2]+	111.0441	111.0442	+0.90	111.0441	0.00	MassBank EU Record: EQ357803
			105.0460		105.0460		
5	[C5H5]+	65.0386	65.0387	+1.54	65.0387	+1.54	MassBank EU Record: EQ357803
			53.0389		53.0389		

SI-4.3.6. Atenolol

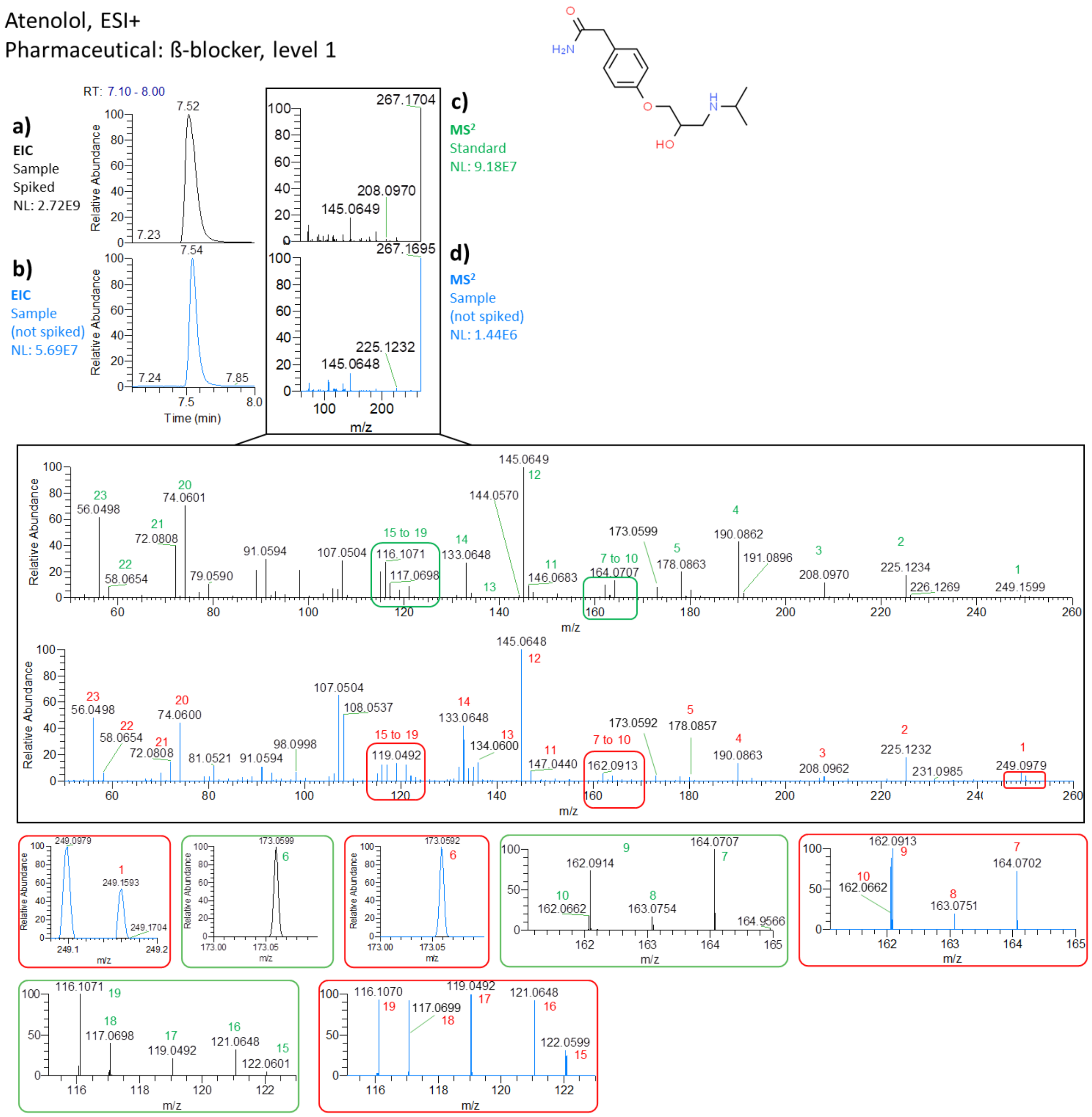

Figure S12. Confirmed identification of atenolol. Comparison of extracted ion chromatogram (EIC) between a surface water sample a) spiked with atenolol at 5 µg/L and b) not spiked, and of MS2 data from c) authentic atenolol standard and d) a surface water sample not spiked, analysed under the same conditions. Mass deviation and references to the literature related to the fragments flagged with numbers are available in Table S12. NL: normalized level.

Table S12. Atenolol fragments expected chemical formula, theoretical and measured masses, and related mass deviation, from the DDA MS2 of the standard and sample reinjected for confirmation

Fragment	(Expected) Chemical formula	Theoretical mass	Measured mass for the standard	Mass error (standard) (ppm)	Measured mass for the sample	Mass error (sample) (ppm)	Reference
Parent	[C14H22N2O3+H]+	267.1703	267.1703	0.00	267.1695	-2.99	
1	[C14H20N2O2]+	249.1598	249.1595	-1.20	249.1593	-2.01	[11]
2	[C11H17N2O3]+	225.1234	225.1234	0.00	225.1232	-0.89	[11] MassBank EU Record: EA016911
3	[C11H14NO3]+	208.0968	208.0968	0.00	208.0962	-2.88	[11] MassBank EU Record: EA016911
4	[C11H12NO2]+	190.0863	190.0861	-1.05	190.0864	+0.53	[11] MassBank EU Record: EA016911
5	[C10H12NO2]+	178.0863	178.0863	0.00	178.0857	-3.37	MassBank EU Record: EA016911
6	[C10H12NO2]+	173.0597	173.0599	+1.16	173.0592	-2.89	MassBank EU Record: EA016911
7	[C11H9O2]+	164.0706	164.0707	+0.61	164.0702	-2.44	MassBank EU Record: EA016911
8	[C9H10NO2]+	163.0754	163.0754	0.00	163.0751	-1.84	MassBank EU Record: EA016911
9	[C10H11O2]+	162.0913	162.0914	+0.62	162.0913	0.00	MassBank EU Record: EA016911
10	[C10H12NO]+	162.0662	162.0662	0.00	162.0657	-3.09	MassBank EU Record: EA016911
11	[C8H8N3O]+	147.0441	147.0441	0.00	147.0440	-0.68	MassBank EU Record: EA016911
12	[C9H7O2]+	145.0648	145.0649	+0.69	145.0648	0.00	MassBank EU Record: EA016911

Fragment	(Expected) Chemical formula	Theoretical mass	Measured mass for the standard	Mass error (standard) (ppm)	Measured mass for the sample	Mass error (sample) (ppm)	Reference
13	[C10H9O]+	134.0600	134.0600	0.00	134.0600	0.00	MassBank EU Record: EA016911
14	[C8H8NO]+	133.0648	133.0648	0.00	133.0648	0.00	MassBank EU Record: EA016911
15	[C9H9O]+	122.0600	122.0601	+0.82	122.0599	-0.82	MassBank EU Record: EA016911
16	[C7H8NO]+	121.0648	121.0648	0.00	121.0648	0.00	MassBank EU Record: EA016911
17	[C8H9O]+	119.0491	119.0492	+0.84	119.0492	+0.84	MassBank EU Record: EA016911
18	[C8H7O]+	117.0699	117.0698	-0.85	117.0699	0.00	MassBank EU Record: EA016911
19	[C6H14NO]+	116.1070	116.1071	+0.86	116.1070	0.00	[11] MassBank EU Record: EA016911
			107.0504		107.0504		
20	[C3H8NO]+	74.0600	74.0601	+1.35	74.0600	0.00	[11] MassBank EU Record: EA016911
21	[C4H10N]+	72.0808	72.0808	0.00	72.0808	0.00	[11] MassBank EU Record: EA016911
22	[C3H8N]+	58.0651	58.0498	+5.17	58.0498	+5.17	MassBank EU Record: EA016911
23	[C3H6N]+	56.0495	58.0654	+5.35	58.0654	+5.35	[11] MassBank EU Record: EA016911

SI-4.3.7. Atrazine

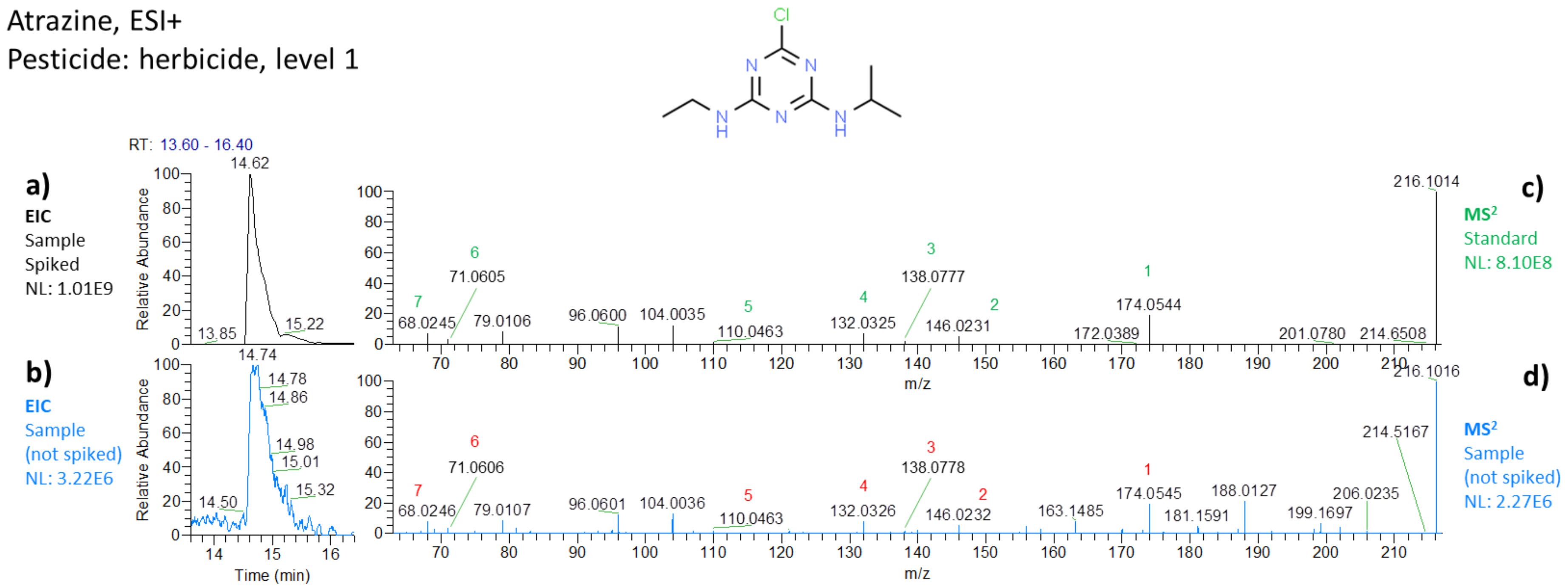

Figure S13. Confirmed identification of atrazine. Comparison of extracted ion chromatogram (EIC) between a surface water sample a) spiked with atrazine at 5 µg/L and b) not spiked, and of MS2 data from c) authentic atrazine standard and d) a surface water sample not spiked, analysed under the same conditions. Mass deviation and references to the literature related to the fragments flagged with numbers are available in Table S13. NL: normalized level.

Table S13. Atrazine fragments expected chemical formula, theoretical and measured masses, and related mass deviation, from the DDA MS2 of the standard and sample reinjected for confirmation

Fragment	(Expected) Chemical formula	Theoretical mass	Measured mass for the standard	Mass error (standard) (ppm)	Measured mass for the sample	Mass error (sample) (ppm)	Reference
Parent	$[C_8H_{14}ClN_5+H]^+$	216.1010	216.1014	+1.85	216.1016	+2.78	
1	$[C_5H_9ClN_5]^+$	174.0541	174.0544	+1.72	174.0545	+2.30	[11] MassBank EU Record: EA028804
2	$[C_3H_5ClN_5]^+$	146.0228	146.0231	+2.05	146.0232	+2.74	[11] MassBank EU Record: EA028804
3	$[C_5H_8N_5]^+$	138.0774	138.0777	+2.17	138.0778	+2.90	[11] MassBank EU Record: EA028804
4	$[C_4H_7ClN_3]^+$	132.0323	132.0325	+1.51	132.0326	+2.27	[11] MassBank EU Record: EA028804
5	$[C_3H_4N_5]^+$	110.0461	110.0463	+1.82	110.0463	+1.82	[11]
			104.0035		10.0036		
			96.0600		96.0601		
			79.0106		79.0107		
6	$[C_3H_7N_2]^+$	71.0604	71.0605	+1.41	71.0606	+2.81	[11] MassBank EU Record: EA028804
7	$[C_2H_2N_3]^+$	68.0243	68.0245	+2.94	68.0246	+4.41	[11] MassBank EU Record: EA028804

SI-4.3.8. Bis(2-ethylhexyl) phosphate

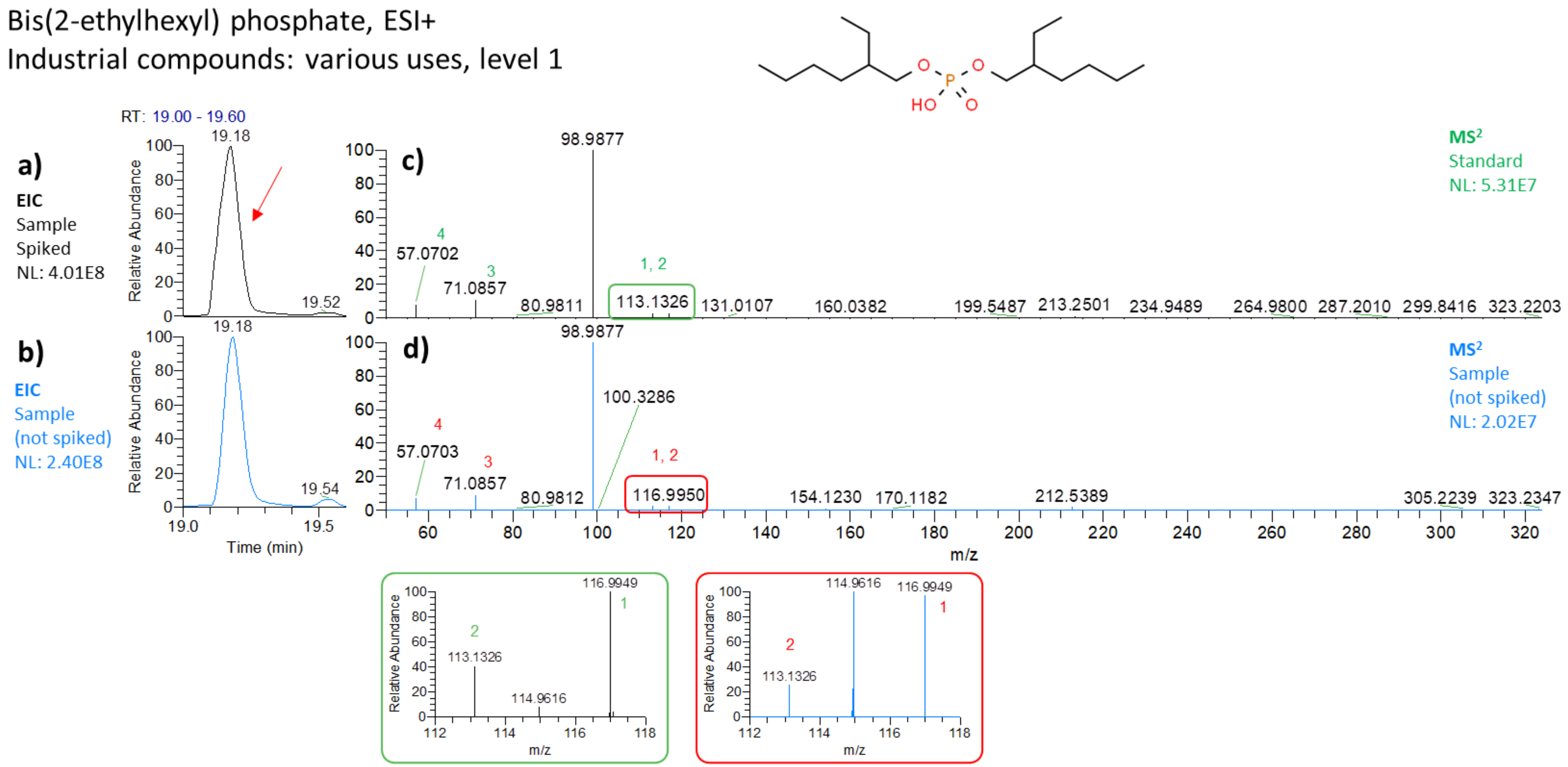

Figure S14. Confirmed identification of bis(2-ethylhexyl) phosphate. Comparison of extracted ion chromatogram (EIC) between a surface water sample a) spiked with bis(2-ethylhexyl) phosphate at 5 µg/L and b) not spiked, and of MS2 data from c) authentic bis(2-ethylhexyl) phosphate standard and d) a surface water sample not spiked, analysed under the same conditions. Mass deviation and references to the literature related to the fragments flagged with numbers are available in Table S14. NL: normalized level.

Table S14. Bis(2-ethylhexyl) phosphate fragments expected chemical formula, theoretical and measured masses, and related mass deviation, from the DDA MS2 of the standard and sample reinjected for confirmation

**Fragment**	**(Expected) Chemical formula**	**Theoretical mass**	**Measured mass for the standard**	**Mass error (standard) (ppm)**	**Measured mass for the sample**	**Mass error (sample) (ppm)**	**Reference**
Parent	[C16H35O4P+H]+	323.2346	Not detected in the MS2	-	323.2347	+0.31	
1	[H6O5P]+	116.9947	116.9949	+1.71	116.9950	+2.56	MassBank EU Record: SM883301
			114.9616		114.9616		
2	[C8H17]+	113.1325	113.1326	+0.88	113.1326	+0.88	MassBank EU Record: SM883301
			98.9877		98.9877		
			80.9811		80.9812		
3	[C5H11]+	71.0855	71.0857	+2.81	71.0857	+2.81	MassBank EU Record: SM883301
4	[C4H9]+	57.0699	57.0702	+5.26	57.0703	+7.01	MassBank EU Record: SM883301

SI-4.3.9. Caffeine

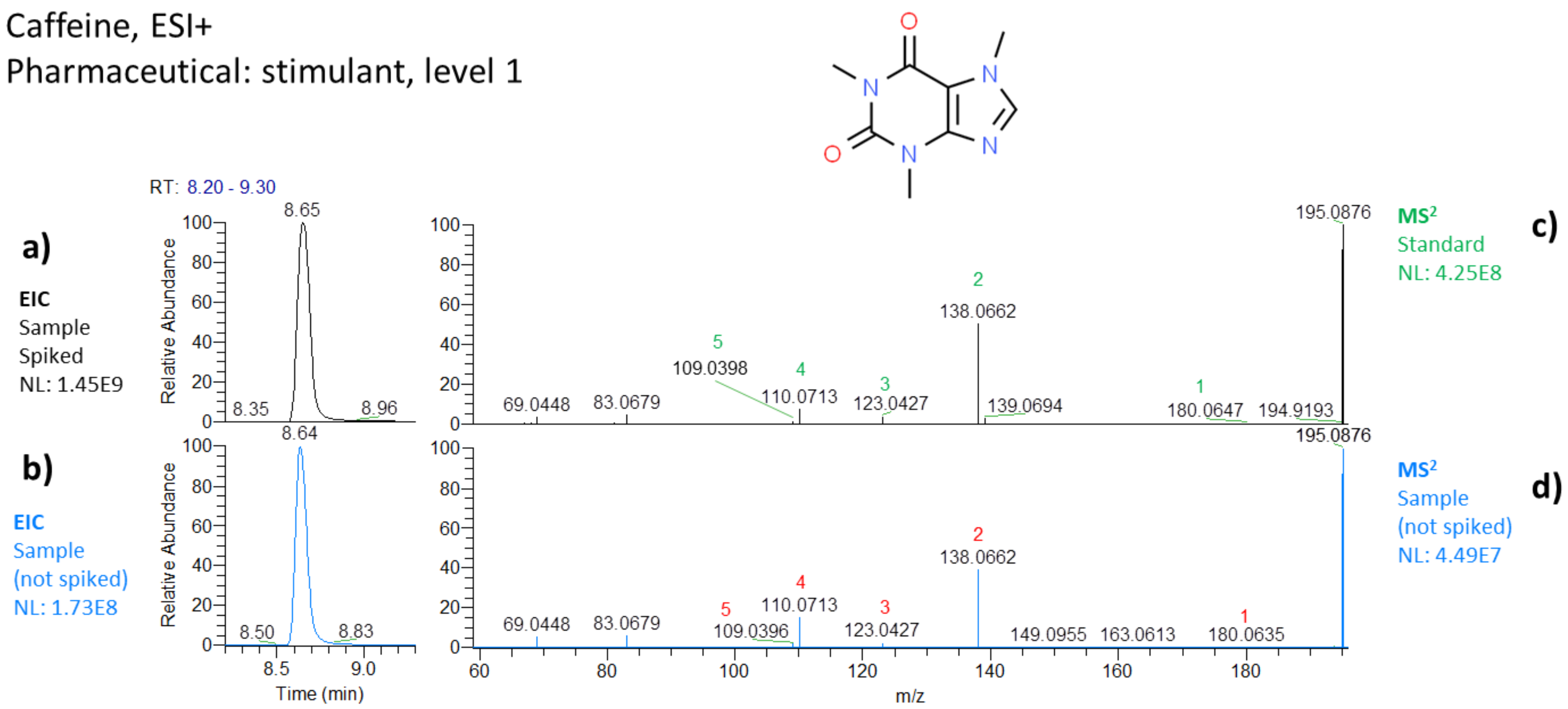

Figure S15. Confirmed identification of caffeine. Comparison of extracted ion chromatogram (EIC) between a surface water sample a) spiked with caffeine at 5 µg/L and b) not spiked, and of MS2 data from c) authentic caffeine standard and d) a surface water sample not spiked, analysed under the same conditions. Mass deviation and references to the literature related to the fragments flagged with numbers are available in Table S15. NL: normalized level.

Table S15. Caffeine fragments expected chemical formula, theoretical and measured masses, and related mass deviation, from the DDA MS2 of the standard and sample reinjected for confirmation

Fragment	(Expected) Chemical formula	Theoretical mass	Measured mass for the standard	Mass error (standard) (ppm)	Measured mass for the sample	Mass error (sample) (ppm)	Reference
Parent	[C8H10N4O2+H]+	195.0877	195.0876	-0.51	195.0876	-0.51	
1	[C7H8N4O2]+	180.0642	180.0647	+2.78	180.0635	-3.89	[12]
2	[C6H8N3O]+	138.0662	138.0662	0.00	138.0662	0.00	[12] MassBank EU Record: UF408901
3	[C5H5N3O]+	123.0427	123.0427	0.00	123.0427	0.00	MassBank EU Record: UF408901
4	[C5H8N3]+	110.0713	110.0713	0.00	110.0713	0.00	[12] MassBank EU Record: UF408901
5	[C5H5N2O]+	109.0396	109.0398	+1.83	109.0396	0.00	MassBank EU Record: UF408901

SI-4.3.10. Carbamazepine

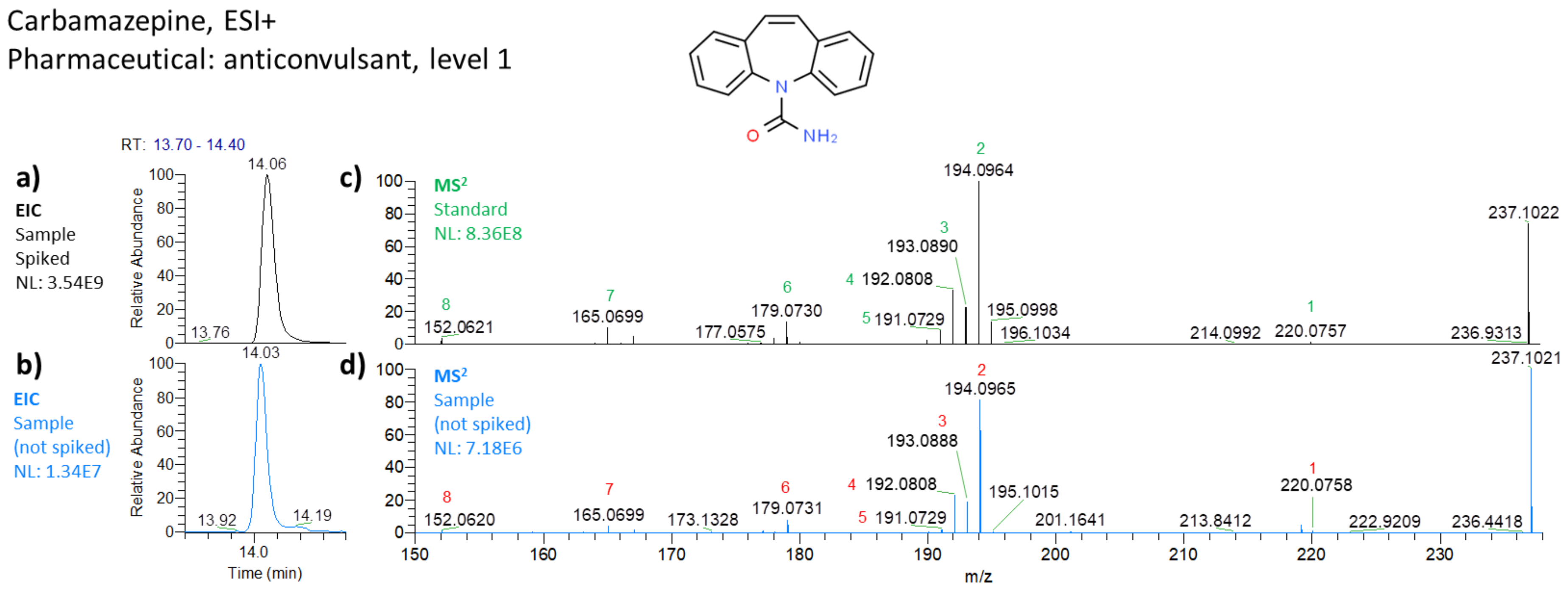

Figure S16. Confirmed identification of carbamazepine. Comparison of extracted ion chromatogram (EIC) between a surface water sample a) spiked with carbamazepine at 5 µg/L and b) not spiked, and of MS2 data from c) authentic carbamazepine standard and d) a surface water sample not spiked, analysed under the same conditions. Mass deviation and references to the literature related to the fragments flagged with numbers are available in Table S16. NL: normalized level.

Table S16. Carbamazepine fragments expected chemical formula, theoretical and measured masses, and related mass deviation, from the DDA MS2 of the standard and sample reinjected for confirmation

Fragment	(Expected) Chemical formula	Theoretical mass	Measured mass for the standard	Mass error (standard) (ppm)	Measured mass for the sample	Mass error (sample) (ppm)	Reference
Parent	[C15H12N2O+H]+	237.1022	237.1022	0.00	237.1021	-0.42	
1	[C15H10NO]+	220.0757	220.0757	0.00	220.0758	+0.45	[11]
2	[C14H12N]+	194.0964	194.0964	0.00	194.0965	+0.52	[11,13] MassBank EU Record: EA019406
3	[C14H11N]+	193.0886	193.0890	+2.07	193.0888	+1.04	MassBank EU Record: EA019406
4	[C14H10N]+	192.0808	192.0808	0.00	192.0808	0.00	[11,13] MassBank EU Record: EA019406
5	[C14H9N]+	191.0730	191.0729	-0.52	191.0729	-0.52	MassBank EU Record: EA019406
6	[C13H9N]+	179.0730	179.0730	0.00	179.0731	+0.56	[13] MassBank EU Record: EA019406
7	[C13H9]+	165.0699	165.0699	0.00	165.0699	0.00	[13] MassBank EU Record: EA019406
8	[C12H8]+	152.0621	152.0621	0.00	152.0620	-0.66	MassBank EU Record: EA019406

SI-4.3.11. Carbendazim

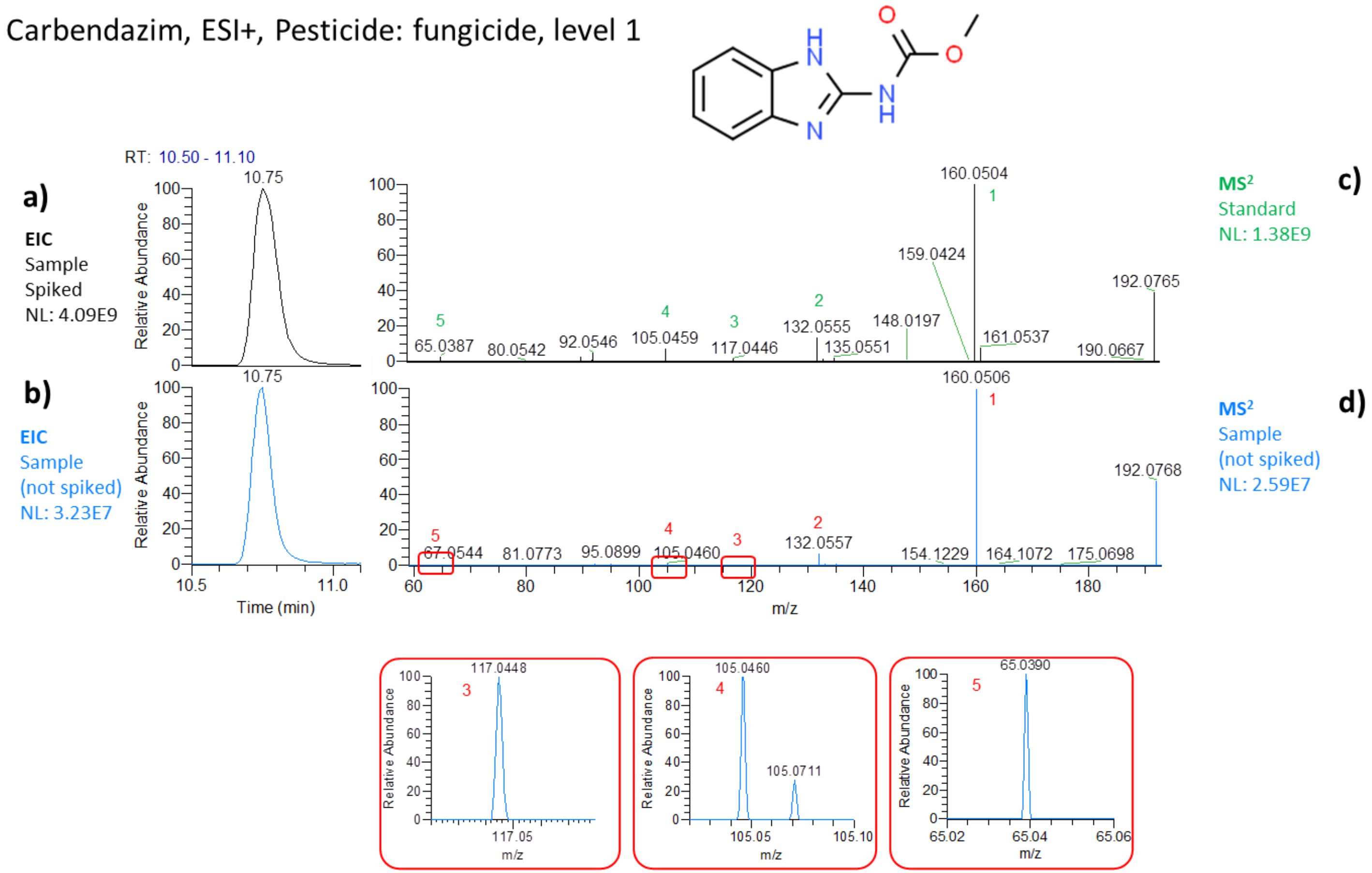

Figure S17. Confirmed identification of carbendazim. Comparison of extracted ion chromatogram (EIC) between a surface water sample a) spiked with carbendazim at 5 µg/L and b) not spiked, and of MS2 data from c) authentic carbendazim standard and d) a surface water sample not spiked, analysed under the same conditions. Mass deviation and references to the literature related to the fragments flagged with numbers are available in Table S17. NL: normalized level.

Table S17. Carbendazim fragments expected chemical formula, theoretical and measured masses, and related mass deviation, from the DDA MS2 of the standard and sample reinjected for confirmation

Fragment	(Expected) Chemical formula	Theoretical mass	Measured mass for the standard	Mass error (standard) (ppm)	Measured mass for the sample	Mass error (sample) (ppm)	Reference
Parent	[C9H9N3O2+H]+	192.0768	192.0765	-1.56	192.0768	0.00	
1	[C8H6N3O]+	160.0505	160.0504	-0.62	160.0506	+.062	[11]
2	[C7H6N3]+	132.0556	132.0555	-0.76	132.0557	+0.76	[11]
3	[C7H5N2]+	117.0447	117.0446	-0.85	117.0448	+0.85	MassBank EU Records: AU286103, EQ027805
4	[C6H5N2]+	105.0447	105.0459	+11.42	105.0460	+12.38	[11]
5	[C5H5]+	65.0386	65.0387	+1.54	65.0390	+6.15	MassBank EU Record: EQ027805

SI-4.3.12. Chlorpyrifos

Chlorpyrifos, ESI+
Pesticide: insecticide, level 1

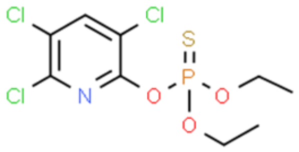

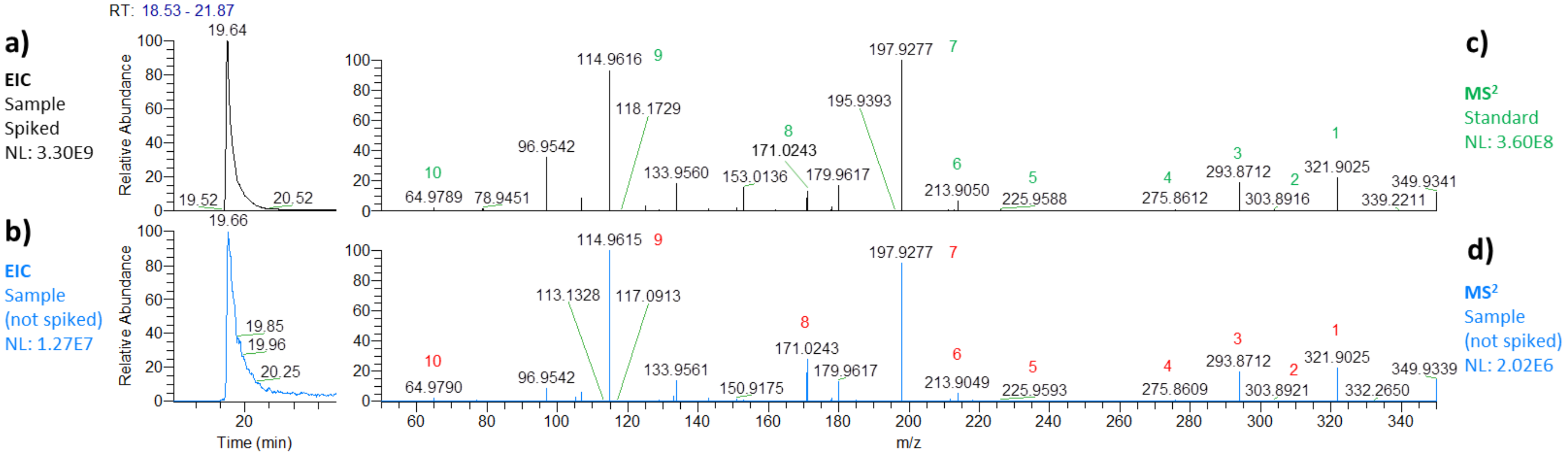

Figure S18. Confirmed identification of chlorpyrifos. Comparison of extracted ion chromatogram (EIC) between a surface water sample a) spiked with chlorpyrifos at 5 µg/L and b) not spiked, and of MS2 data from c) authentic chlorpyrifos standard and d) a surface water sample not spiked, analysed under the same conditions. Mass deviation and references to the literature related to the fragments flagged with numbers are available in Table S18. NL: normalized level.

Table S18. Chlorpyrifos fragments expected chemical formula, theoretical and measured masses, and related mass deviation, from the DDA MS2 of the standard and sample reinjected for confirmation

Fragment	(Expected) Chemical formula	Theoretical mass	Measured mass for the standard	Mass error (standard) (ppm)	Measured mass for the sample	Mass error (sample) (ppm)	Reference
Parent	[C9H11Cl3NO3PS+H]+	349.9336	349.9341	+1.43	349.9341	+1.43	[11] MassBank EU Record: EA295003
1	[C7H8Cl3NO3PS]+	321.9023	321.9025	+0.62	321.9025	+0.62	[11] MassBank EU Record: EA295003
2	[C7H6Cl3NO2PS]+	303.8917	303.8916	-0.33	303.8921	+1.32	MassBank EU Record: EA295003
3	[C5H4Cl3NO3PS]+	293.8710	293.8712	+0.68	293.8712	+0.68	[11] MassBank EU Record: EA295003
4	[C5H2Cl3NO2PS]+	275.8604	275.8612	+2.90	275.8609	+1.81	[11] MassBank EU Record: EA295003
5	[C7H7Cl3NO]+	225.9588	225.9588	0.00	225.9593	+2.12	MassBank EU Record: EA295003
6	[C5H3Cl3NS]+	213.9046	213.9050	+1.87	213.9049	+1.40	[11] MassBank EU Record: EA295003
7	[C5H3Cl3NO]+	197.9275	197.9277	+1.01	197.9277	+1.01	[11] MassBank EU Record: EA295003
			179.9617		179.9617		
8	[C4H12O3PS]+	171.0239	171.0243	+2.34	171.0243	+2.34	MassBank EU Record: EA295003
			133.9560		133.9561		
9	[H4O3PS]+	114.9613	114.9616	+2.61	114.9615	+1.74	MassBank EU Record: EA295003
			96.9542		96.9542		
10	[H2O2P]+	64.9787	64.9789	+3.08	64.9790	+4.62	[11]

SI-4.3.13. Clarithromycin

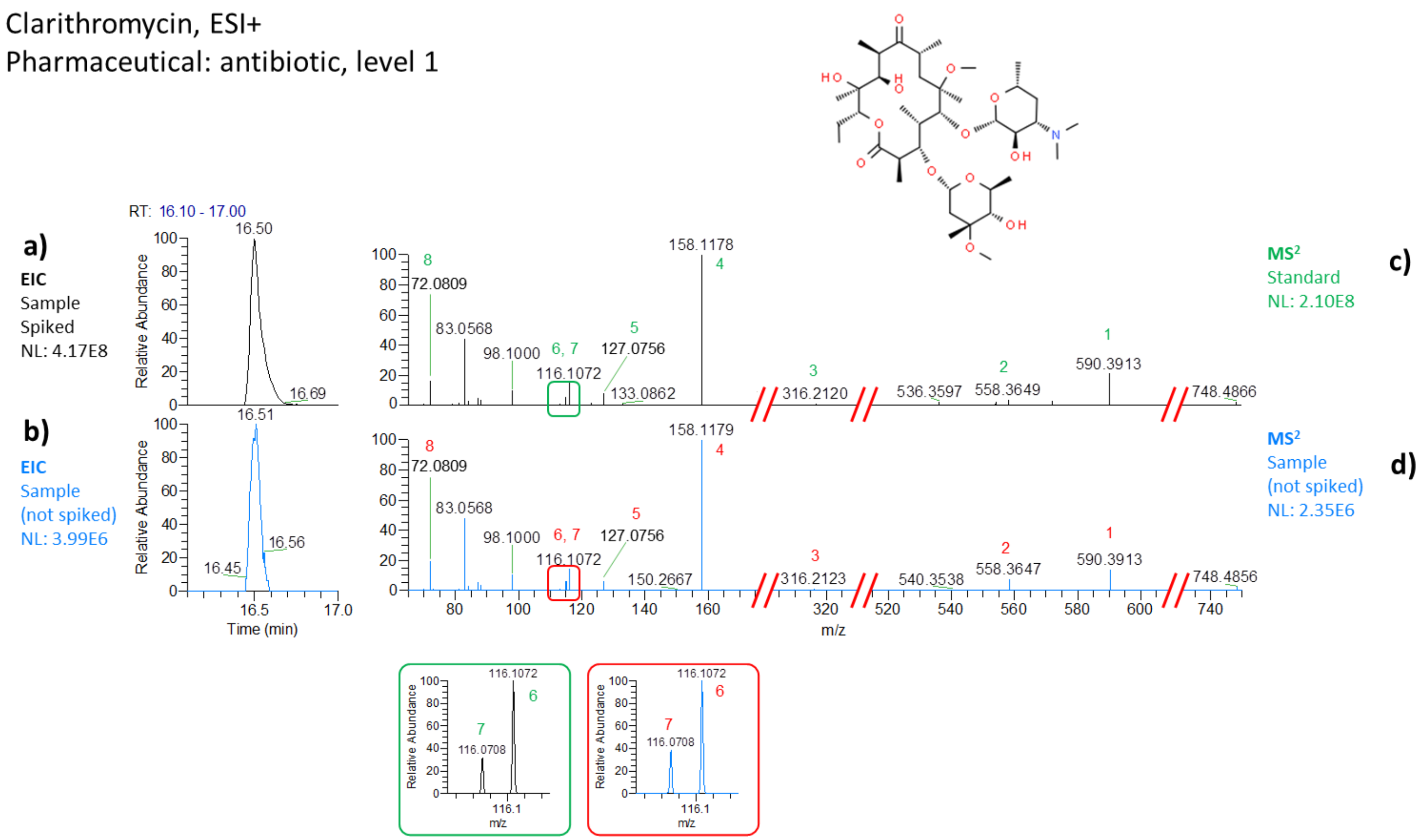

Figure S19. Confirmed identification of clarithromycin. Comparison of extracted ion chromatogram (EIC) between a surface water sample a) spiked with clarithromycin at 5 µg/L and b) not spiked, and of MS2 data from c) authentic clarithromycin standard and d) a surface water sample not spiked, analysed under the same conditions. Mass deviation and references to the literature related to the fragments flagged with numbers are available in Table S19. NL: normalized level.

Table S19. Clarithromycin fragments expected chemical formula, theoretical and measured masses, and related mass deviation, from the DDA MS2 of the standard and sample reinjected for confirmation

**Fragment**	**(Excepted) Chemical formula**	**Theoretical mass**	**Measured mass for the standard**	**Mass error (standard) (ppm)**	**Measured mass for the sample**	**Mass error (sample) (ppm)**	**Reference**
Parent	[C38H69NO13+H]+	748.4842	748.4866	+3.21	748.4856	+1.87	
1	[C30H56NO10]+	590.3899	590.3913	+2.37	590.3913	+2.37	[11] MassBank EU Record: EA019108
2	[C29H52NO9]+	558.3637	558.3649	+2.15	558.3647	+1.79	MassBank EU Record: EA019108
3	[C16H30NO5]+	316.2118	316.2120	+0.63	316.2123	+1.58	MassBank EU Record: EA019108
4	[C8H16NO2]+	158.1176	158.1178	+1.26	158.1179	+1.90	[11] MassBank EU Record: EA019108
5	[C7H11O2]+	127.0754	127.0756	+1.57	127.0756	+1.57	MassBank EU Record: EA019108
6	[C6H14NO]+	116.1070	116.1072	+1.72	116.1072	+1.72	[11] MassBank EU Record: EA019108
7	[C5H10NO2]+	116.0706	116.0708	+1.72	116.0708	+1.72	MassBank EU Record: EA019108
			98.1000		98.1000		
			83.0568		83.0568		
8	[C4H10N]+	72.0808	72.0809	+1.39	72.0809	+1.39	MassBank EU Record: EA019108

SI-4.3.14. Cotinine

Cotinine, ESI+
Pharmaceutical: stimulant, level 1

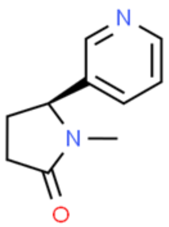

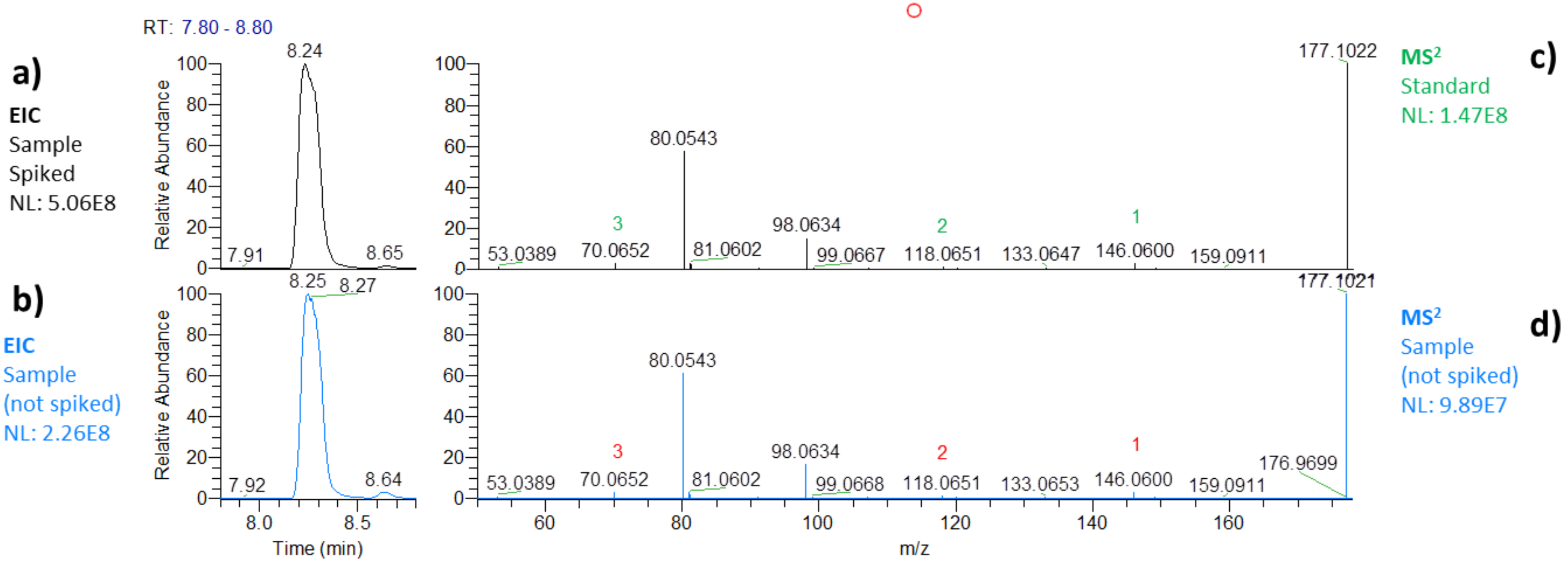

Figure S20. Confirmed identification of cotinine. Comparison of extracted ion chromatogram (EIC) between a surface water sample a) spiked with cotinine at 5 µg/L and b) not spiked, and of MS2 data from c) authentic cotinine standard and d) a surface water sample not spiked, analysed under the same conditions. Mass deviation and references to the literature related to the fragments flagged with numbers are available in Table S20. NL: normalized level.

Table S20. Cotinine fragments expected chemical formula, theoretical and measured masses, and related mass deviation, from the DDA MS2 of the standard and sample reinjected for confirmation

Fragment	(Expected) Chemical formula	Theoretical mass	Measured mass for the standard	Mass error (standard) (ppm)	Measured mass for the sample	Mass error (sample) (ppm)	Reference
Parent	[C10H12N2O+H]+	177.1022	177.1022	0.00	177.1021	-0.56	
1	[C9H8NO]+	146.0600	146.0600	0.00	146.0600	0.00	[12] MassBank EU Record: UF413003
2	[C8H8N]+	118.0651	118.0651	0.00	118.0651	0.00	[12] MassBank EU Record: UF413003
3	[C4H8N]+	70.0651	70.0652	+1.43	70.0652	+1.43	MassBank EU Record: UF413003

SI-4.3.15. Daidzein

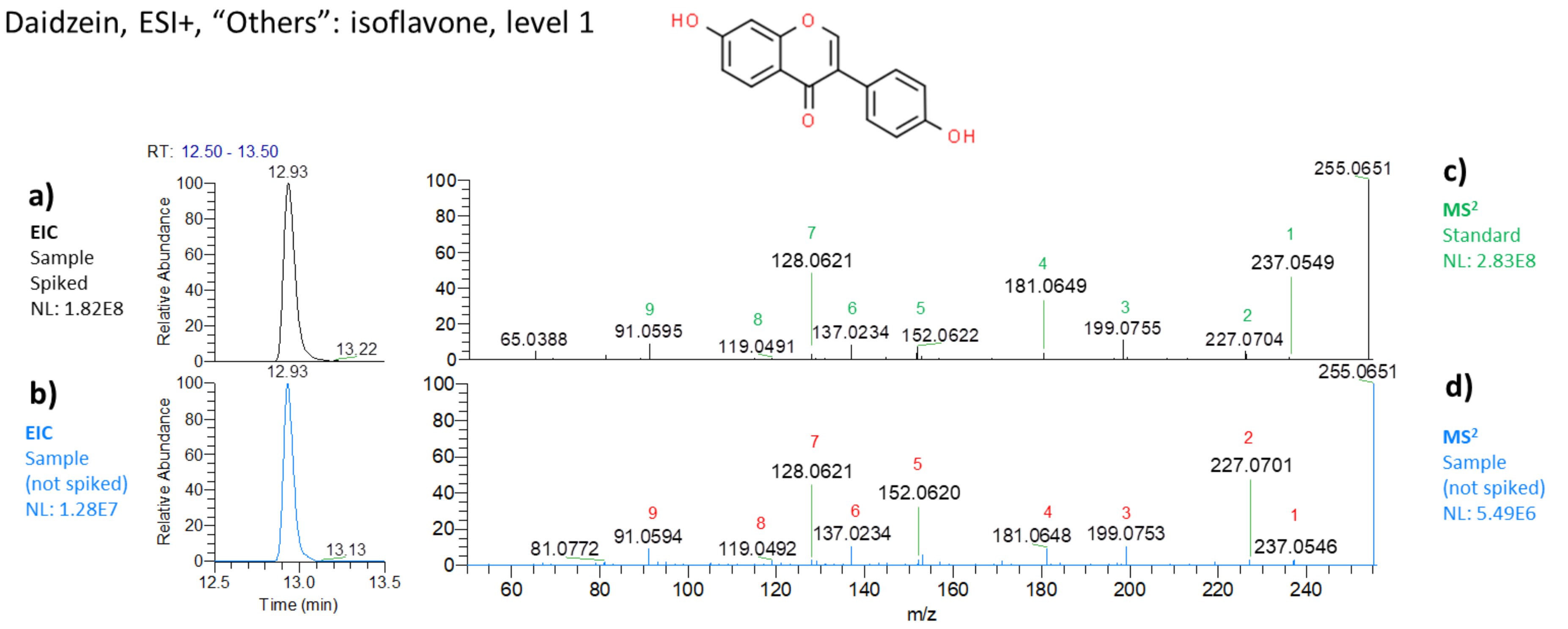

Figure S21. Confirmed identification of daidzein. Comparison of extracted ion chromatogram (EIC) between a surface water sample a) spiked with daidzein at 5 µg/L and b) not spiked, and of MS^2 data from c) authentic daidzein standard and d) a surface water sample not spiked, analysed under the same conditions. Mass deviation and references to the literature related to the fragments flagged with numbers are available in Table S21. NL: normalized level.

Table S21. Daidzein fragments expected chemical formula, theoretical and measured masses, and related mass deviation, from the DDA MS^2 of the standard and sample reinjected for confirmation

Fragment	(Expected) Chemical formula	Theoretical mass	Measured mass for the standard	Mass error (standard) (ppm)	Measured mass for the sample	Mass error (sample) (ppm)	Reference
Parent	[C15H10O4+H]+	255.0652	255.0650	-0.78	255.0650	-0.78	
1	[C15H9O3]+	237.0546	237.0549	+1.27	237.0546	0.00	[14] MassBank EU Record: UF423901
2	[C14H11O3]+	227.0703	227.0704	+0.44	227.0701	-0.88	[14] MassBank EU Record: UF423901
3	[C13H11O2]+	199.0754	199.0755	+0.50	199.0753	-0.50	[14] MassBank EU Record: UF423901
4	[C13H9O]+	181.0648	181.0649	+0.55	181.0648	0.00	[14] MassBank EU Record: UF423901
5	[C12H8]+	152.0621	152.0622	+0.66	152.0620	-0.66	MassBank EU Record: UF423901
6	[C7H5O3]+	137.0233	137.0234	+0.73	137.0234	+0.73	[14] MassBank EU Record: UF423901
7	[C10H8]+	128.0621	128.0621	0.00	128.0621	0.00	MassBank EU Record: UF423901
8	[C8H7O]+	119.0491	119.0491	0.00	119.0492	+0.84	[14] MassBank EU Record: UF423901

SI-4.3.16. Diazinon

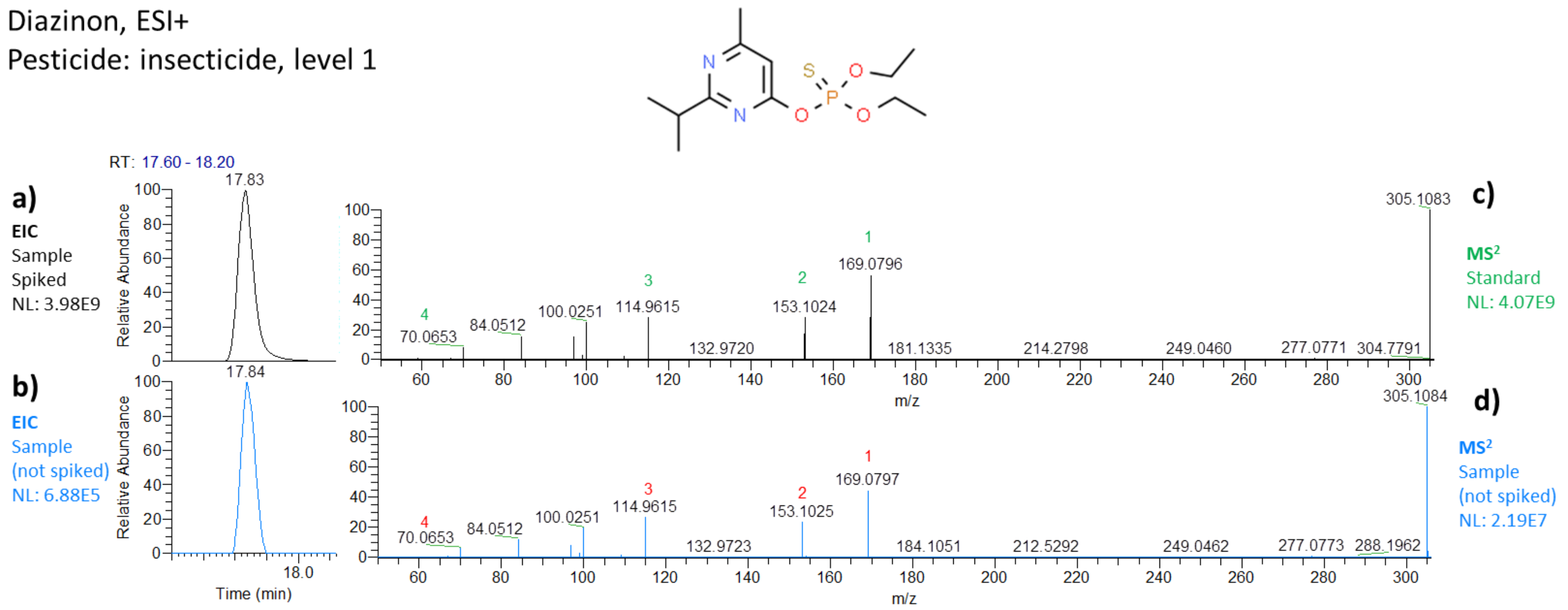

Figure S22. Confirmed identification of diazinon. Comparison of extracted ion chromatogram (EIC) between a surface water sample a) spiked with diazinon at 5 µg/L and b) not spiked, and of MS2 data from c) authentic diazinon standard and d) a surface water sample not spiked, analysed under the same conditions. Mass deviation and references to the literature related to the fragments flagged with numbers are available in Table S22. NL: normalized level.

Table S22. Diazinon fragments expected chemical formula, theoretical and measured masses, and related mass deviation, from the DDA MS2 of the standard and sample reinjected for confirmation

Fragment	(Expected) Chemical formula	Theoretical mass	Measured mass for the standard	Mass error (standard) (ppm)	Measured mass for the sample	Mass error (sample) (ppm)	Reference
Parent	[C12H21N2O3PS+H]+	305.1083	305.1084	+0.33	305.1084	+0.33	
1	[C8H13N2S]+	169.0794	169.0797	+1.77	169.0797	+1.77	[15] MassBank EU Record: EA029304
2	[C8H13N2O]+	153.1022	153.1025	+1.96	153.1025	+1.96	[15] MassBank EU Record: EA029304
			132.9720		132.9723		
3	[H4O3PS]+	114.9613	114.9615	+1.74	114.9615	+1.74	[15] MassBank EU Record: EA029304
			100.0251		100.0251		
			84.0512		84.0512		
4	[C4H8N]+	70.0651	70.0653	+2.85	70.0653	+2.85	MassBank EU Record: EA029304

SI-4.3.17. Diclofenac

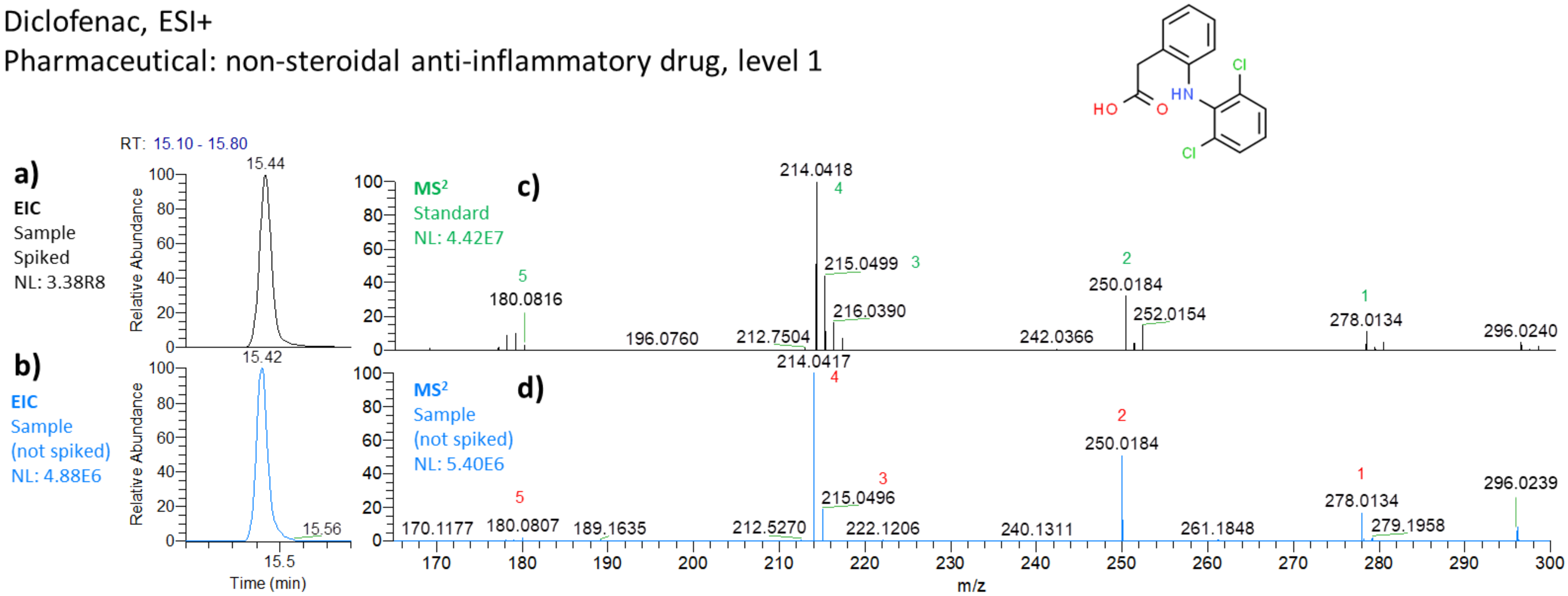

Figure S23. Confirmed identification of diclofenac. Comparison of extracted ion chromatogram (EIC) between a surface water sample a) spiked with diclofenac at 5 µg/L and b) not spiked, and of MS2 data from c) authentic diclofenac standard and d) a surface water sample not spiked, analysed under the same conditions. Mass deviation and references to the literature related to the fragments flagged with numbers are available in Table S23. NL: normalized level.

Table S23. Diclofenac fragments expected chemical formula, theoretical and measured masses, and related mass deviation, from the DDA MS2 of the standard and sample reinjected for confirmation

Fragment	(expected) Chemical formula	Theoretical mass	Measured mass for the standard	Mass error (standard) (ppm)	Measured mass for the sample	Mass error (sample) (ppm)	Reference
Parent	$[C_{14}H_{11}NO_2Cl_2+H]^+$	296.0240	296.0240	0.00	296.0239	-0.34	
1	$[C_{14}H_{10}Cl_2NO]^+$	278.0134	278.0134	0.00	278.0134	0.00	[11] MassBank EU Record: EA020109
2	$[C_{13}H_{10}Cl_2N]^+$	250.0185	250.0184	-0.40	250.0184	-0.40	[11] MassBank EU Record: EA020109
3	$[C_{13}H_{10}ClN]^+$	215.0496	215.0499	+1.40	215.0496	0.00	[11] MassBank EU Records: EA020109, EA020112
4	$[C_{13}H_9ClN]^+$	214.0418	214.0418	0.00	214.0417	-0.47	[11] MassBank EU Records: EA020109, EA020112
5	$[C_{13}H_{10}N]^+$	180.0808	180.0816	+4.44	180.0807	-0.56	MassBank EU Record: EA020112

SI-4.3.18. Dimethoate

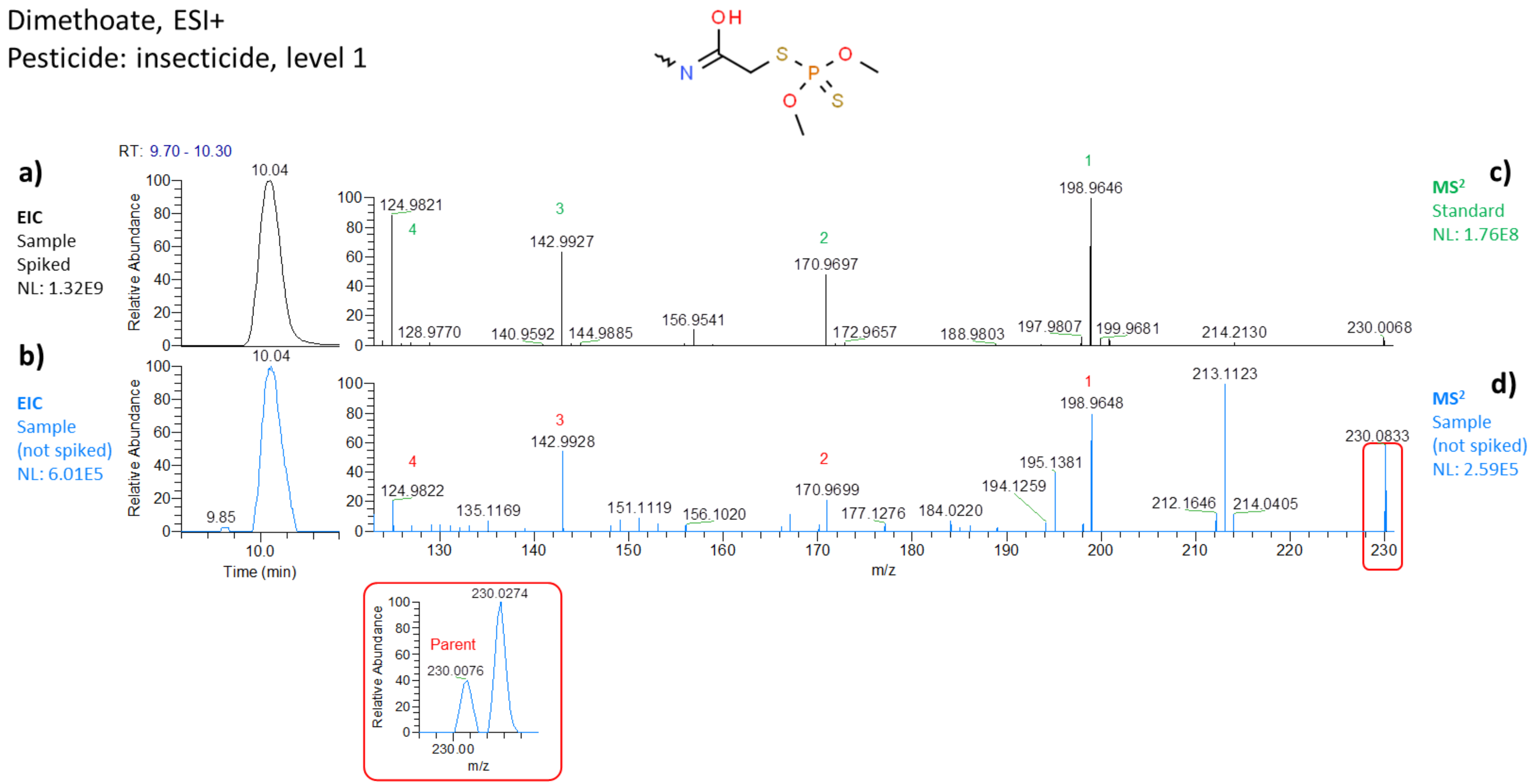

Figure S24. Confirmed identification of dimethoate. Comparison of extracted ion chromatogram (EIC) between a surface water sample a) spiked with dimethoate at 5 µg/L and b) not spiked, and of MS2 data from c) authentic dimethoate standard and d) a surface water sample not spiked, analysed under the same conditions. Mass deviation and references to the literature related to the fragments flagged with numbers are available in Table S24. NL: normalized level.

Table S24. Dimethoate fragments expected chemical formula, theoretical and measured masses, and related mass deviation, from the DDA MS2 of the standard and sample reinjected for confirmation

Fragment	(Expected) Chemical formula	Theoretical mass	Measured mass for the standard	Mass error (standard) (ppm)	Measured mass for the sample	Mass error (sample) (ppm)	Reference
Parent	[C5H12NO3PS2+H]+	230.0069	230.0068	-0.43	230.0076	+3.04	
1	[C4H8O3PS2]+	198.9647	198.9646	-0.50	198.9648	+0.50	[11] MassBank EU Record: EA276109
2	[C3H8O2PS2]+	170.9698	170.9697	-0.58	170.9699	+0.58	[11] MassBank EU Record: EA276109
3	[C2H8O3PS]+	142.9926	142.9927	+0.70	142.9928	+1.40	[11] MassBank EU Record: EA276109
4	[C2H6O2PS]+	124.9821	124.9821	0.00	124.9822	+0.80	MassBank EU Record: EA276109

SI-4.3.19. Diphenyl phosphate

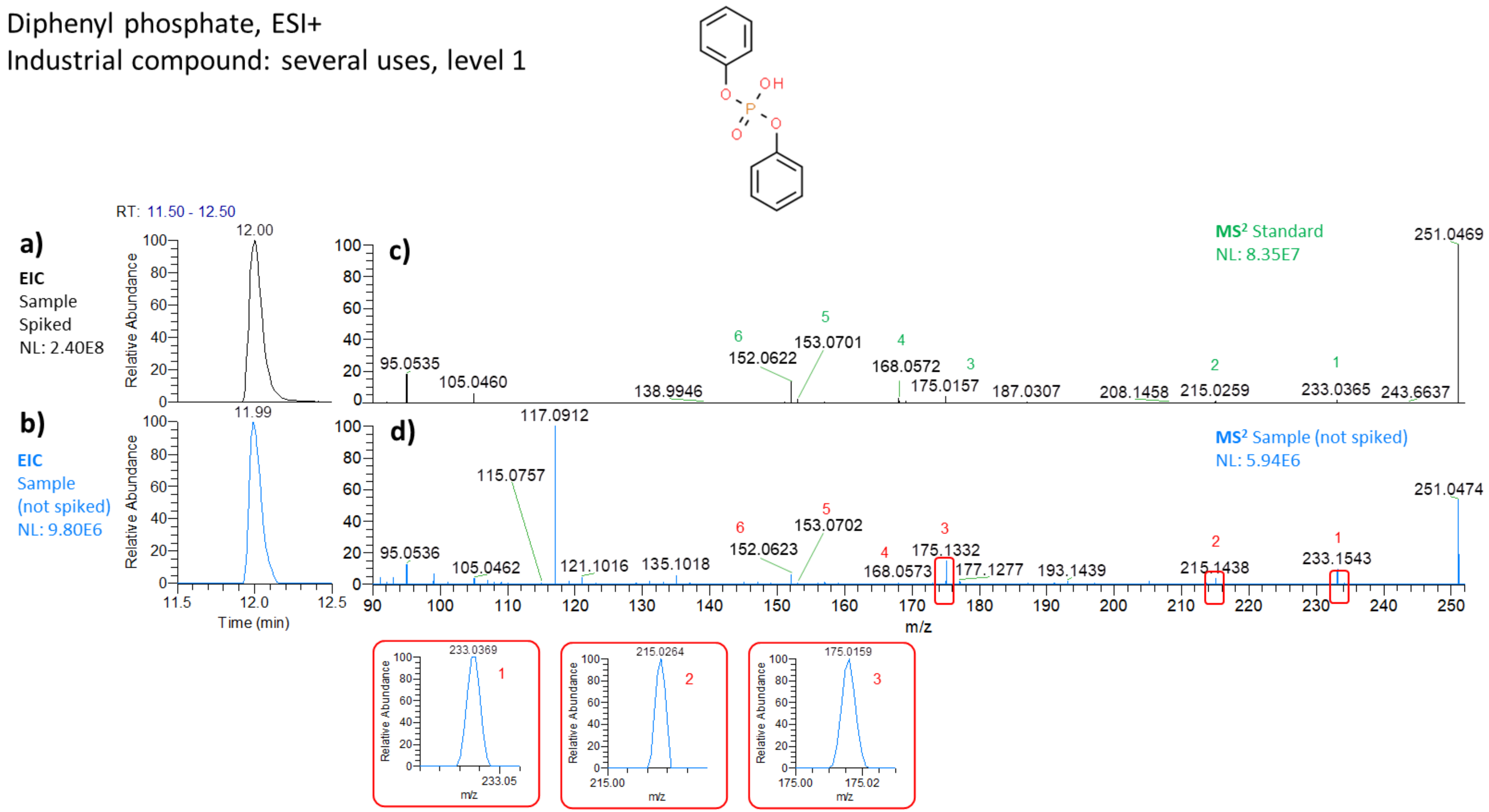

Figure S25. Confirmed identification of diphenyl phosphate. Comparison of extracted ion chromatogram (EIC) between a surface water sample a) spiked with diphenyl phosphate at 5 µg/L and b) not spiked, and of MS2 data from c) authentic diphenyl phosphate standard and d) a surface water sample not spiked, analysed under the same conditions. Mass deviation and references to the literature related to the fragments flagged with numbers are available in Table S25. NL: normalized level.

Table S25. Diphenol phosphate fragments expected chemical formula, theoretical and measured masses, and related mass deviation, from the DDA MS2 of the standard and sample reinjected for confirmation

Fragment	(Expected) Chemical formula	Theoretical mass	Measured mass for the sample	Mass error (standard) (ppm)	Mass error (sample) (ppm)	Mass deviation for the sample (ppm)	Reference
Parent	[C12H11O4P+H]+	251.0468	251.0469	+0.40	251.0474	+2.39	
1	[C12H10O3P]+	233.0362	233.0365	+1.29	233.0369	+3.00	[16] MassBank EU Record: SM822501
2	[C12H8O2P]+	215.0256	215.0259	+1.40	215.0264	+3.72	MassBank EU Record: SM822501
3	[C6H8O4P]+	175.0155	175.0157	+1.14	175.0159	+2.29	MassBank EU Record: SM822501
4	[C12H8O]+	168.0570	168.0572	+1.19	168.0573	+1.79	MassBank EU Record: SM822501
5	[C12H9]+	153.0699	153.0701	+1.31	153.0702	+1.96	[16] MassBank EU Record: SM822501
6	[C12H8]+	152.0621	152.0622	+0.68	152.0623	+1.32	MassBank EU Record: SM822501
			105.0460		105.0462		
			95.0535		95.0536		

SI-4.3.20. Diuron

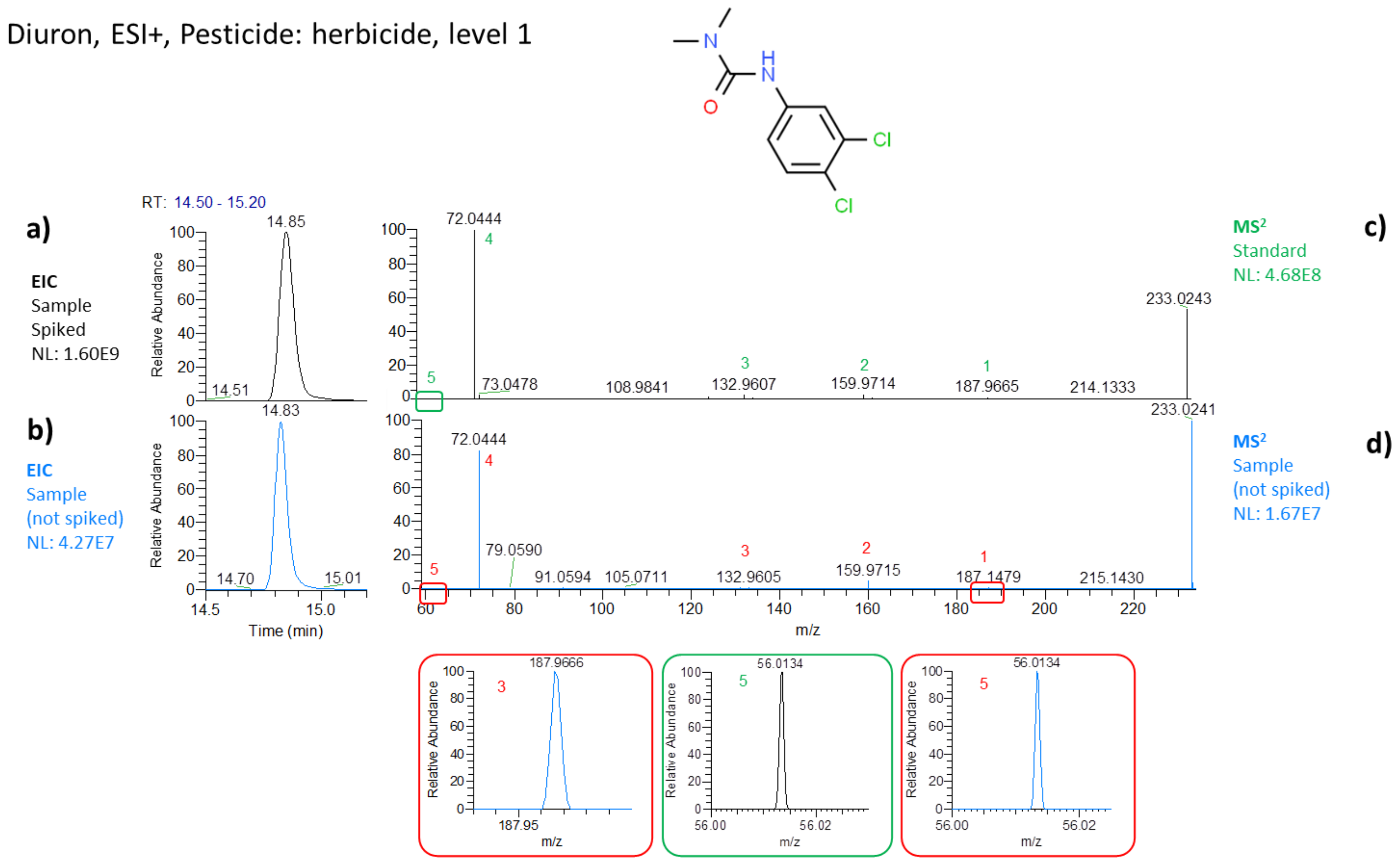

Figure S26. Confirmed identification of diuron. Comparison of extracted ion chromatogram (EIC) between a surface water sample a) spiked with diuron at 5 µg/L and b) not spiked, and of MS^2 data from c) authentic diuron standard and d) a surface water sample not spiked, analysed under the same conditions. Mass deviation and references to the literature related to the fragments flagged with numbers are available in Table S26. NL: normalized level.

Table S26. Diuron fragments expected chemical formula, theoretical and measured masses, and related mass deviation, from the DDA MS^2 of the standard and sample reinjected for confirmation

Fragment	(Expected) Chemical formula	Theoretical mass	Measured mass for the standard	Mass error (standard) (ppm)	Measured mass for the sample	Mass error (sample) (ppm)	Reference
Parent	[C9H10Cl2N2O+H]+	233.0243	233.0243	0.00	233.0241	-0.86	
1	[C7H4Cl2NO]+	187.9665	187.9665	0.00	187.9666	+0.53	[11] MassBank EU Record: EA029210
2	[C6H4NCl2]+	159.9715	159.9714	-0.63	159.9715	0.00	[11] MassBank EU Record: EA029210
3	[C5H3Cl2]+	132.9606	132.9607	+0.75	132.9605	-0.75	[10]
4	[C3H6NO]+	72.0444	72.0444	0.00	72.044	0.00	[11] MassBank EU Record: EA029210
5	[C2H2NO]+	56.0131	56.0134	+5.36	56.01314	+5.36	MassBank EU Record: EA029210

SI-4.3.21. Erythromycin

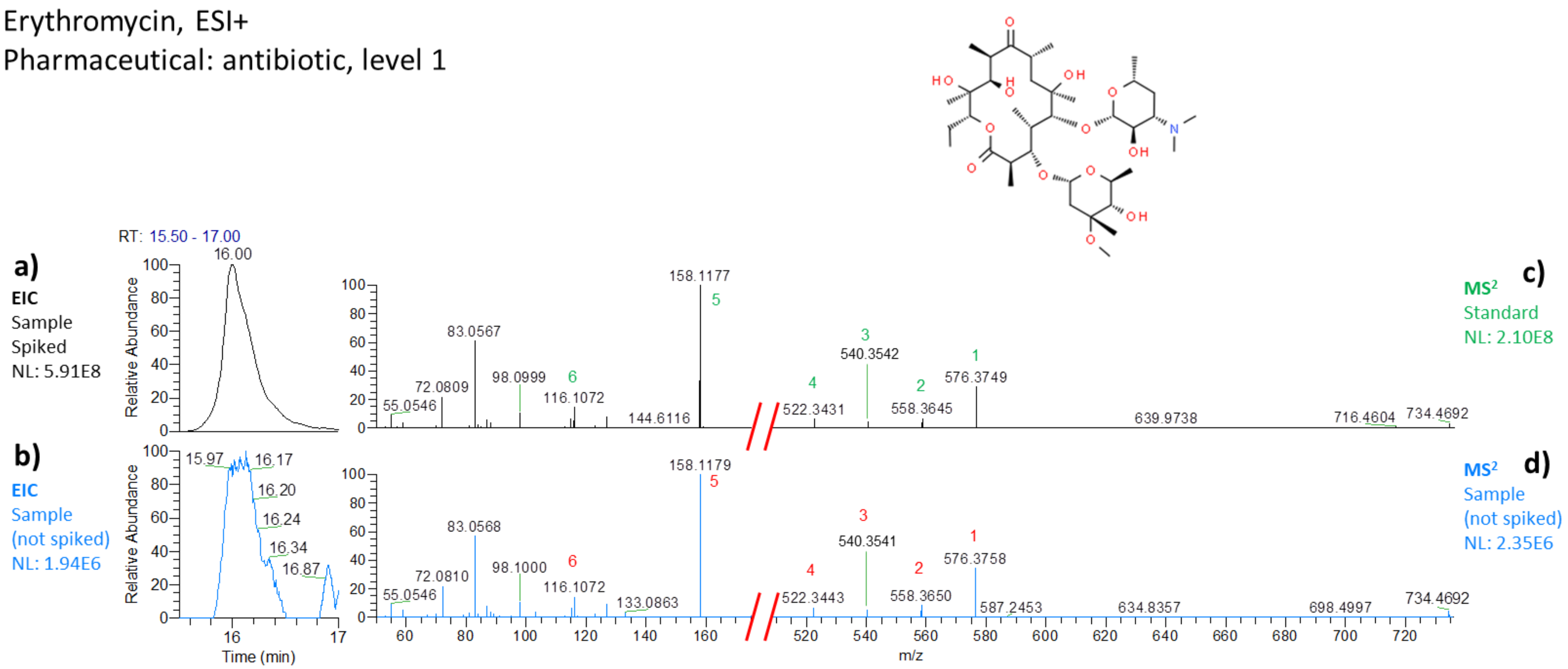

Figure S27. Confirmed identification of erythromycin. Comparison of extracted ion chromatogram (EIC) between a surface water sample a) spiked with erythromycin at 5 µg/L and b) not spiked, and of MS2 data from c) authentic erythromycin standard and d) a surface water sample not spiked, analysed under the same conditions. Mass deviation and references to the literature related to the fragments flagged with numbers are available in Table S27. NL: normalized level.

Table S27. Erythromycin fragments expected chemical formula, theoretical and measured masses, and related mass deviation, from the DDA MS2 of the standard and sample reinjected for confirmation

Fragment	(Expected) Chemical formula	Theoretical mass	Measured mass for the standard	Mass error (standard) (ppm)	Measured mass for the sample	Mass error (sample) (ppm)	Reference
Parent	[C37H67NO13+H]+	734.4685	734.4692	+0.95	734.4692	+0.95	[11] MassBank EU Record: EA018909
1	[C29H54NO10]+	576.3742	576.3749	+1.21	576.3758	+2.78	[11] MassBank EU Record: EA018909
2	[C29H52NO9]+	558.3637	558.3645	+1.43	558.3650	+2.33	[11]
3	[C29H50NO8]+	540.3531	540.3542	+2.04	540.3541	+1.85	[11]
4	[C29H48NO7]+	522.3425	522.3431	+1.15	522.3443	+3.45	[11]
5	[C8H16NO2]+	158.1176	158.1177	+0.63	158.1179	+1.90	[11] MassBank EU Record: EA018909
6	[C6H14NO]+	116.1070	116.1072	+1.72	116.1072	+1.72	[11] MassBank EU Record: EA018909
			98.0999		98.1000		
			83.0567		83.0568		
			72.0809		72.0810		
			55.0546		55.0546		

SI-4.3.22. Fluconazole

Fluconazole, ESI+
Pharmaceutical: antifungal, level 1

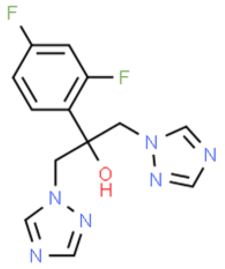

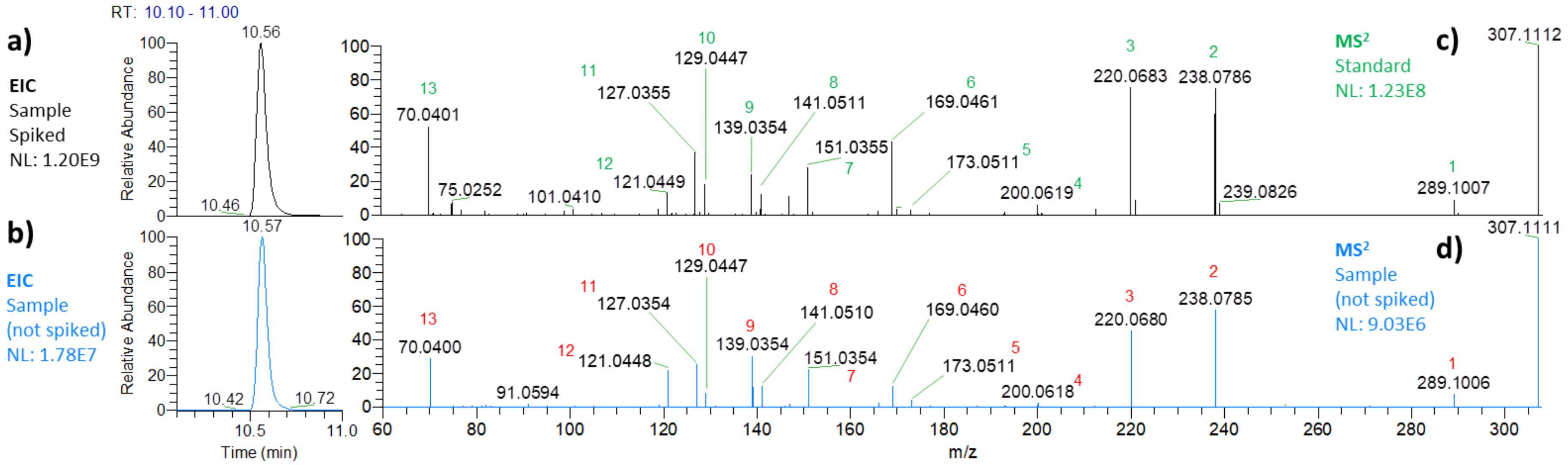

Figure S28. Confirmed identification of fluconazole. Comparison of extracted ion chromatogram (EIC) between a surface water sample a) spiked with fluconazole at 5 µg/L and b) not spiked, and of MS2 data from c) authentic fluconazole standard and d) a surface water sample not spiked, analysed under the same conditions. Mass deviation and references to the literature related to the fragments flagged with numbers are available in Table S28. NL: normalized level.

Table S28. Fluconazole fragments expected chemical formula, theoretical and measured masses, and related mass deviation, from the DDA MS2 of the standard and sample reinjected for confirmation

Fragment	Chemical formula	Theoretical mass	Measured mass for the standard	Mass error (standard) (ppm)	Measured mass for the sample	Mass error (sample) (ppm)	Reference
Parent	[C13H12F2N6O+H]+	307.1113	307.1112	-0.33	307.1111	-0.65	
1	[C13H11F2N6]+	289.1008	289.1007	-0.35	289.1006	-0.69	MassBank EU Record: EA032803
2	[C11H10F2N3O]+	238.0786	238.0786	0.00	238.0785	-0.42	[17] MassBank EU Records: EA032803, EA032805
3	[C11H8F2N3]+	220.0681	220.0683	+0.91	220.0680	-0.45	[17] MassBank EU Records: EA032803, EA032805
4	[C11H7FN3]+	200.0619	200.0619	0.00	200.0618	-0.50	MassBank EU Record: EA032805
5	[C10H6FN2]+	173.0510	173.0511	+0.58	173.0511	+0.58	MassBank EU Record: EA032805
6	[C9H7F2O]+	169.0460	169.0461	+0.59	169.0460	0.00	[17] MassBank EU Records: EA032803, EA032805
7	[C9H5F2]+	151.0354	151.0355	+0.66	151.0354	0.00	MassBank EU Records: EA032803, EA032805
8	[C8H7F2]+	141.0510	141.0511	+0.71	141.0510	0.00	MassBank EU Records: EA032803, EA032805
9	[C8H5F2]+	139.0354	139.0354	0.00	139.0354	0.00	[17] MassBank EU Record: EA032805
10	[C8H5N2]+	129.0447	129.0447	0.00	129.0447	0.00	MassBank EU Record: EA032805
11	[C7H5F2]+	127.0354	127.0355	+0.79	127.0354	0.00	MassBank EU Records: EA032803, EA032805
12	[C8H6F]+	121.0448	121.0449	+0.83	121.0448	0.00	MassBank EU Record: EA032805
13	[C2H4N3]+	70.0400	70.0401	+1.43	70.0400	0.00	[17] MassBank EU Records: EA032803, EA032805

SI-4.3.23. Imidacloprid

Imidacloprid, ESI+

Pesticide: insecticide, level 1

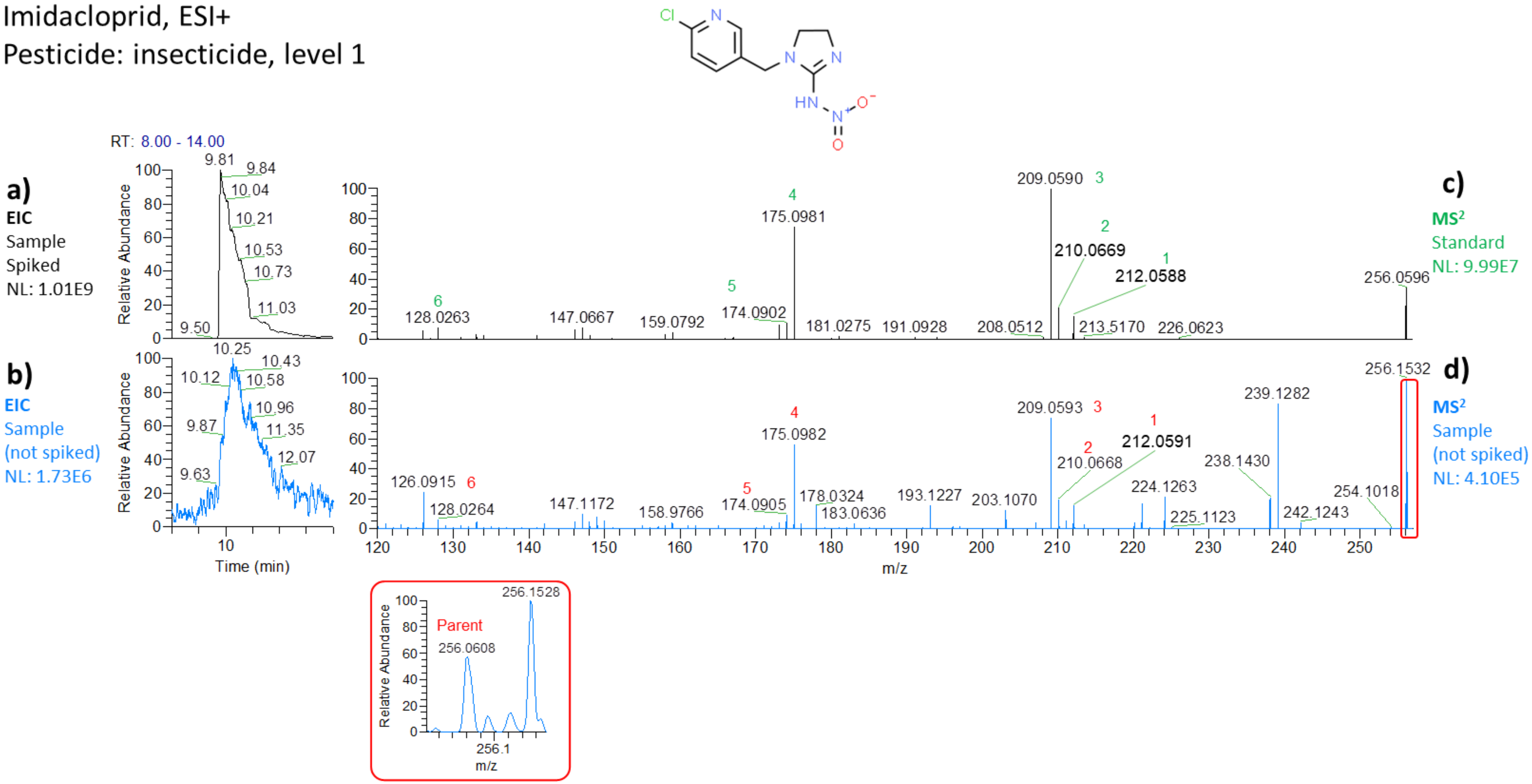

Figure S29. Confirmed identification of imidacloprid. Comparison of extracted ion chromatogram (EIC) between a surface water sample a) spiked with imidacloprid at 5 µg/L and b) not spiked, and of MS2 data from c) authentic imidacloprid standard and d) a surface water sample not spiked, analysed under the same conditions. Mass deviation and references to the literature related to the fragments flagged with numbers are available in Table S29. NL: normalized level.

Table S29. Imidacloprid fragments expected chemical formula, theoretical and measured masses, and related mass deviation, from the DDA MS2 of the standard and sample reinjected for confirmation

Fragment	(Expected) Chemical formula	Theoretical mass	Measured mass for the standard	Mass error (standard) (ppm)	Measured mass for the sample	Mass error (sample) (ppm)	Reference
Parent	[C9H10ClN5O2+H]+	256.0596	256.0597	+0.39	256.0608	+4.69	
1	[C9H11ClN3O]+	212.0585	212.0588	+1.41	212.0591	+2.83	[8] MassBank EU Record: EA270909
2	[C9H11ClN4]+	210.0667	210.0670	+1.43	210.0668	+0.48	[8] MassBank EU Record: EA270909
3	[C9H10ClN4]+	209.0589	209.0592	+1.44	209.0593	+1.91	[8,10] MassBank EU Record: EA270909
4	[C9H11N4]+	175.0978	175.0980	+1.14	175.0982	+2.28	[8,10] MassBank EU Record: EA270909
5	[C9H10N4]+	174.0900	174.0903	+1.72	174.0905	+2.87	MassBank EU Record: EA270909
	[C6H7ClN]+	128.0262	128.0263	+0.78	128.0264	+1.56	[10] MassBank EU Record: EA270909

SI-4.3.24. Losartan

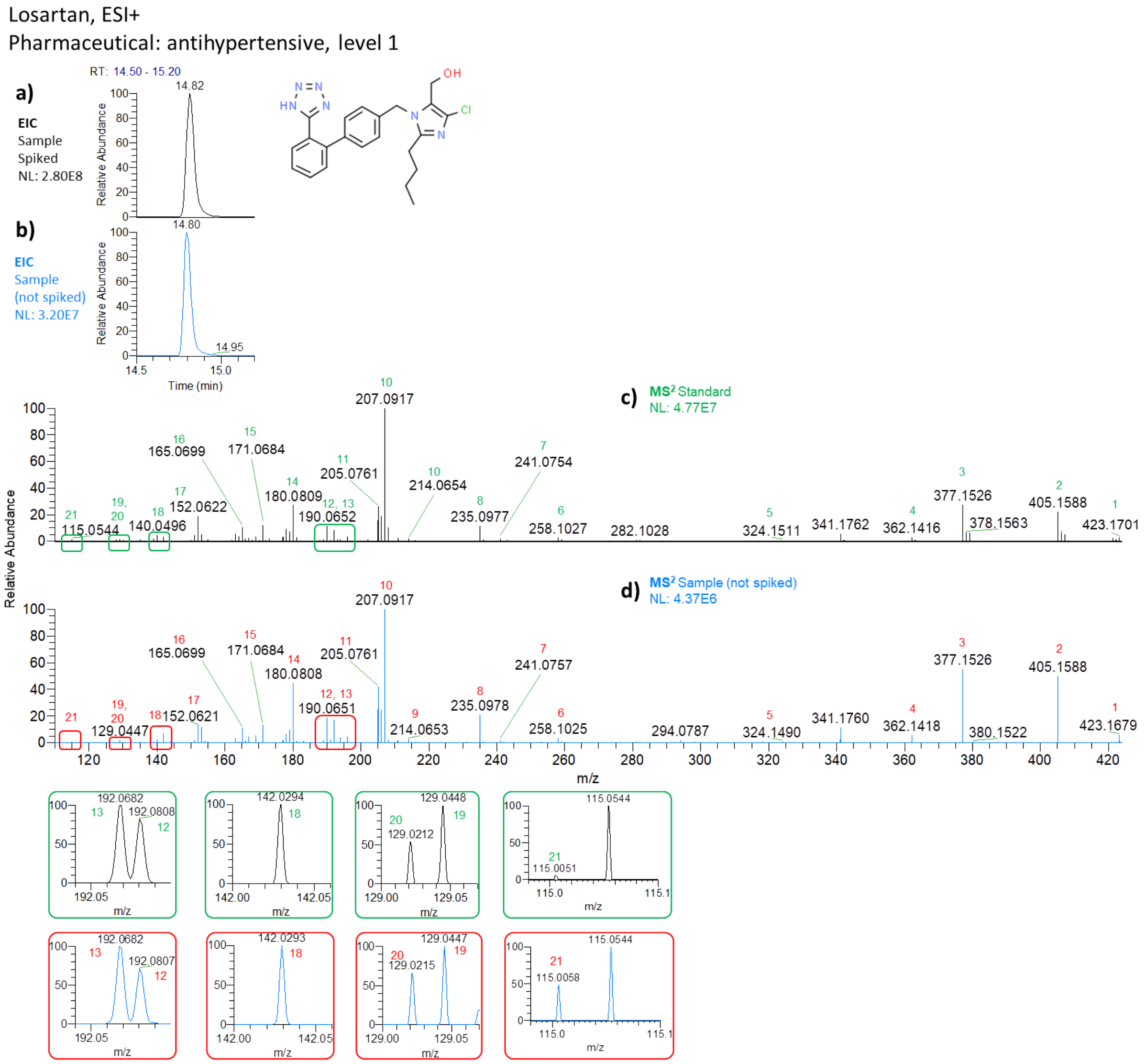

Figure S30. Confirmed identification of losartan. Comparison of extracted ion chromatogram (EIC) between a surface water sample a) spiked with losartan at 5 µg/L and b) not spiked, and of MS2 data from c) authentic losartan standard and d) a surface water sample not spiked, analysed under the same conditions. Mass deviation and references to the literature related to the fragments flagged with numbers are available in Table S30. NL: normalized level.

Table S30. Losartan fragments expected chemical formula, theoretical and measured masses, and related mass deviation, from the DDA MS2 of the standard and sample reinjected for confirmation

Fragment	(Expected) Chemical formula	Theoretical mass	Measured mass for the standard	Mass error (standard) (ppm)	Measured mass for the sample	Mass error (sample) (ppm)	Reference
Parent	[C22H23ClN6O+H]+	423.1695	423.1701	+1.42	423.1679	-3.78	
1	[C22H22ClN6]+	405.1589	405.1588	-0.25	405.1588	-0.25	[11] MassBank EU Record: SM818901
2	[C22H22ClN4]+	377.1528	377.1526	-0.53	377.1526	-0.53	[11] MassBank EU Record: SM818901
3	[C22H21ClN3]+	362.1419	362.1416	-0.83	362.1418	-0.28	MassBank EU Record: SM818901
4	[C22H21N4]+	341.1761	341.1762	+0.29	341.1760	-0.29	MassBank EU Record: SM818901
5	[C17H12N3]+	258.1026	258.1024	-0.77	258.1025	-0.39	MassBank EU Record: SM818901
6	[C17H9N2]+	241.0760	241.0754	-2.49	241.0757	-1.24	MassBank EU Record: SM818901
7	[C14H11N4]+	235.0978	235.0977	-0.43	235.0978	0.00	[11] MassBank EU Record: SM818901
8	[C16H8N]+	214.0651	214.0654	+1.40	214.0653	+0.93	MassBank EU Record: SM818901
9	[C14H11N2]+	207.0917	207.0917	0.00	207.0917	0.00	[11] MassBank EU Record: SM818901
10	[C14H9N2]+	205.0760	205.0761	+0.49	205.0761	+0.49	MassBank EU Record: SM818901
11	[C14H10N]+	192.0808	192.0808	0.00	192.0807	-0.52	[11] MassBank EU Record: SM818901

Fragment	(Expected) Chemical formula	Theoretical mass	Measured mass for the standard	Mass error (standard) (ppm)	Measured mass for the sample	Mass error (sample) (ppm)	Reference
12	[C13H8N2]+	192.0682	192.0682	0.00	192.0682	0.00	[11] MassBank EU Record: SM818901
13	[C14H8N]+	190.0651	190.0653	+1.05	190.0651	0.00	MassBank EU Record: SM818901
14	[C13H10N]+	180.0808	180.0809	+0.56	180.0808	0.00	MassBank EU Record: SM818901
15	[C8H12ClN2]+	171.0684	171.0684	0.00	171.0684	0.00	MassBank EU Record: SM818901
16	[C13H9]+	165.0699	165.0699	0.00	165.0699	0.00	MassBank EU Record: SM818901
17	[C12H8]+	152.0621	152.0622	+0.66	152.0621	0.00	MassBank EU Record: SM818901
18	[C6H7ClN2]+	142.0292	142.0294	+1.41	142.0293	+0.70	MassBank EU Record: SM818901
19	[C8H5N2]+	129.0447	129.0448	+0.77	129.0447	0.00	MassBank EU Record: SM818901
20	[C5H6ClN2]+	129.0214	129.0212	-1.55	129.0215	+0.78	MassBank EU Record: SM818901
21	[C4H4ClN2]+	115.0058	115.0051	-6.09	115.0058	0.00	MassBank EU Record: SM818901

SI-4.3.25. Malathion

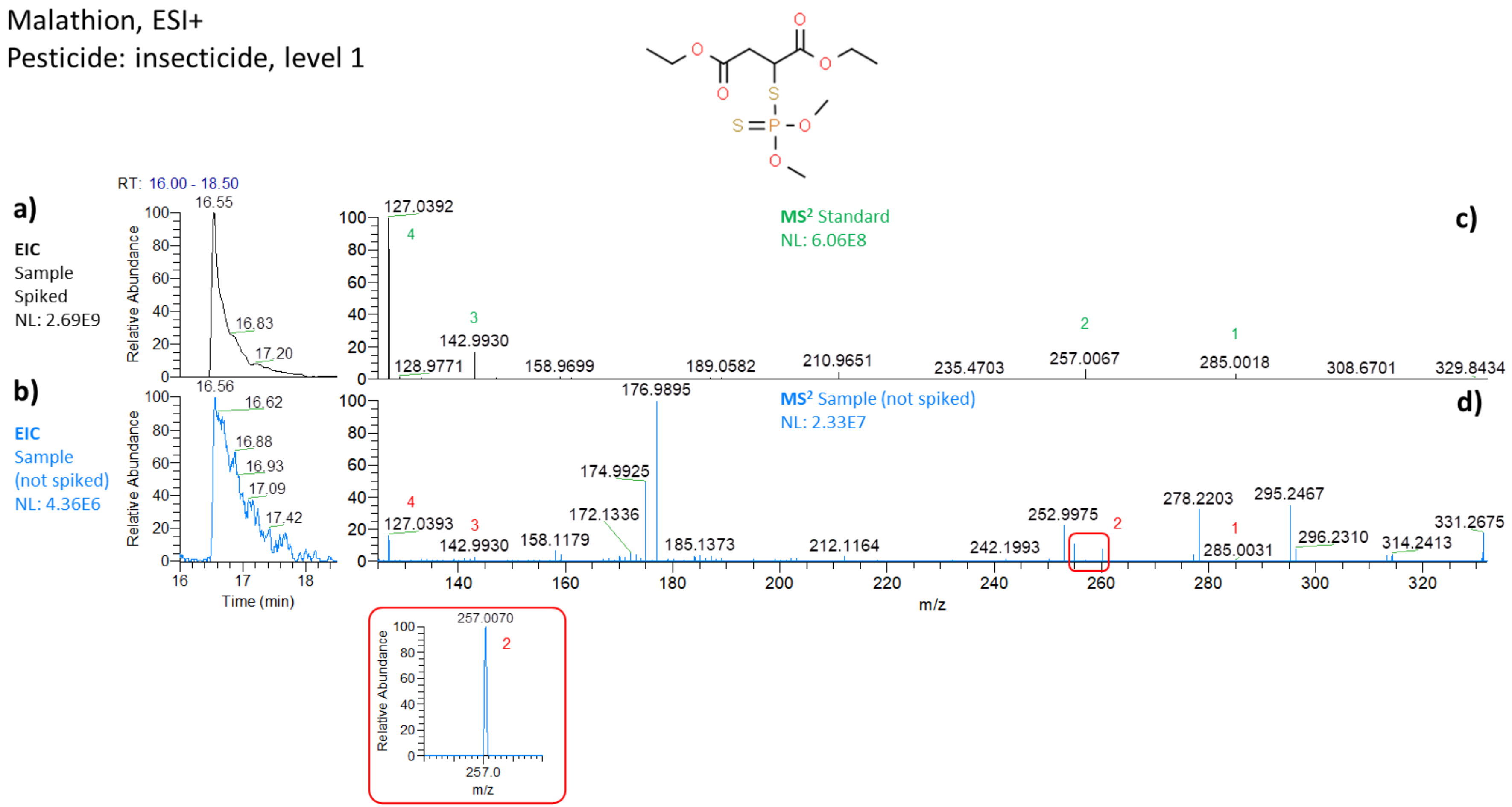

Figure S31. Confirmed identification of malathion. Comparison of extracted ion chromatogram (EIC) between a surface water sample a) spiked with malathion at 5 µg/L and b) not spiked, and of MS2 data from c) authentic malathion standard and d) a surface water sample not spiked, analysed under the same conditions. Mass deviation and references to the literature related to the fragments flagged with numbers are available in Table S31. NL: normalized level.

Table S31. Malathion fragments expected chemical formula, theoretical and measured masses, and related mass deviation, from the DDA MS2 of the standard and sample reinjected for confirmation

Fragment	(Expected) Chemical formula	Theoretical mass	Measured mass for the standard	Mass error (standard) (ppm)	Measured mass for the sample	Mass error (sample) (ppm)	Reference
Parent	$[C_{10}H_{19}O_6PS_2+H]^+$	331.0434	Not detected in MS2	-	Not detected in MS2	-	
1	$[C_8H_{14}O_5PS_2]^+$	285.0015	285.0018	+1.05	285.0031	+5.61	[8,18]
2	$[C_7H_{14}O_4PS_2]^+$	257.0066	257.0067	+0.39	257.0070	+1.56	[8,18]
3	$[C_2H_8O_3PS]^+$	142.9926	142.9930	+2.80	142.9930	+2.80	[8,19]
4	$[C_6H_7O_3]^+$	127.0389	127.0392	+2.36	127.0393	+3.15	[8,18,20]

SI-4.3.26. Metformin

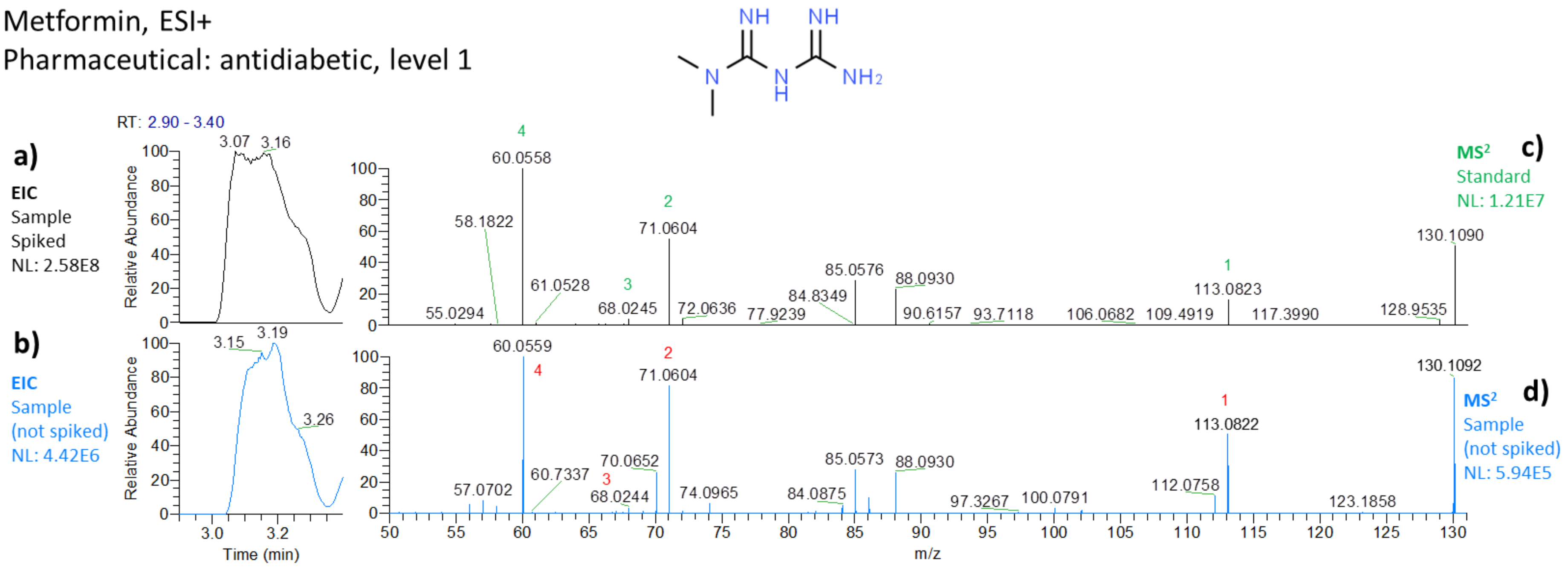

Figure S32. Confirmed identification of metformin. Comparison of extracted ion chromatogram (EIC) between a surface water sample a) spiked with metformin at 5 µg/L and b) not spiked, and of MS2 data from c) authentic metformin standard and d) a surface water sample not spiked, analysed under the same conditions. Mass deviation and references to the literature related to the fragments flagged with numbers are available in Table S32. NL: normalized level.

Table S32. Metformin fragments expected chemical formula, theoretical and measured masses, and related mass deviation, from the DDA MS2 of the standard and sample reinjected for confirmation

Fragment	(Expected) Chemical formula	Theoretical mass	Measured mass for the standard	Mass error (standard) (ppm)	Measured mass for the sample	Mass error (sample) (ppm)	Reference
Parent	[C4H11N5+H]+	130.1087	130.1090	+2.31	130.1092	+3.84	
1	[C4H9N4]+	113.0822	113.0823	+0.88	113.0822	0.00	[11] MassBank EU Record: EA255011
			88.0930		88.0930		
			85.0576		85.0573		
2	[C3H7N2]+	71.0604	71.0604	0.00	71.0604	0.00	[11] MassBank EU Record: EA255011
3	[C2H2N3]+	68.0243	68.0245	+2.94	68.0244	+1.47	MassBank EU Record: EA255011
4	[CH6N3]+	60.0556	60.0558	+3.33	60.0559	+5.00	[11] MassBank EU Record: EA255011

SI-4.3.27. Nicotine

Nicotine, ESI+
Pharmaceutical: stimulant, level 1

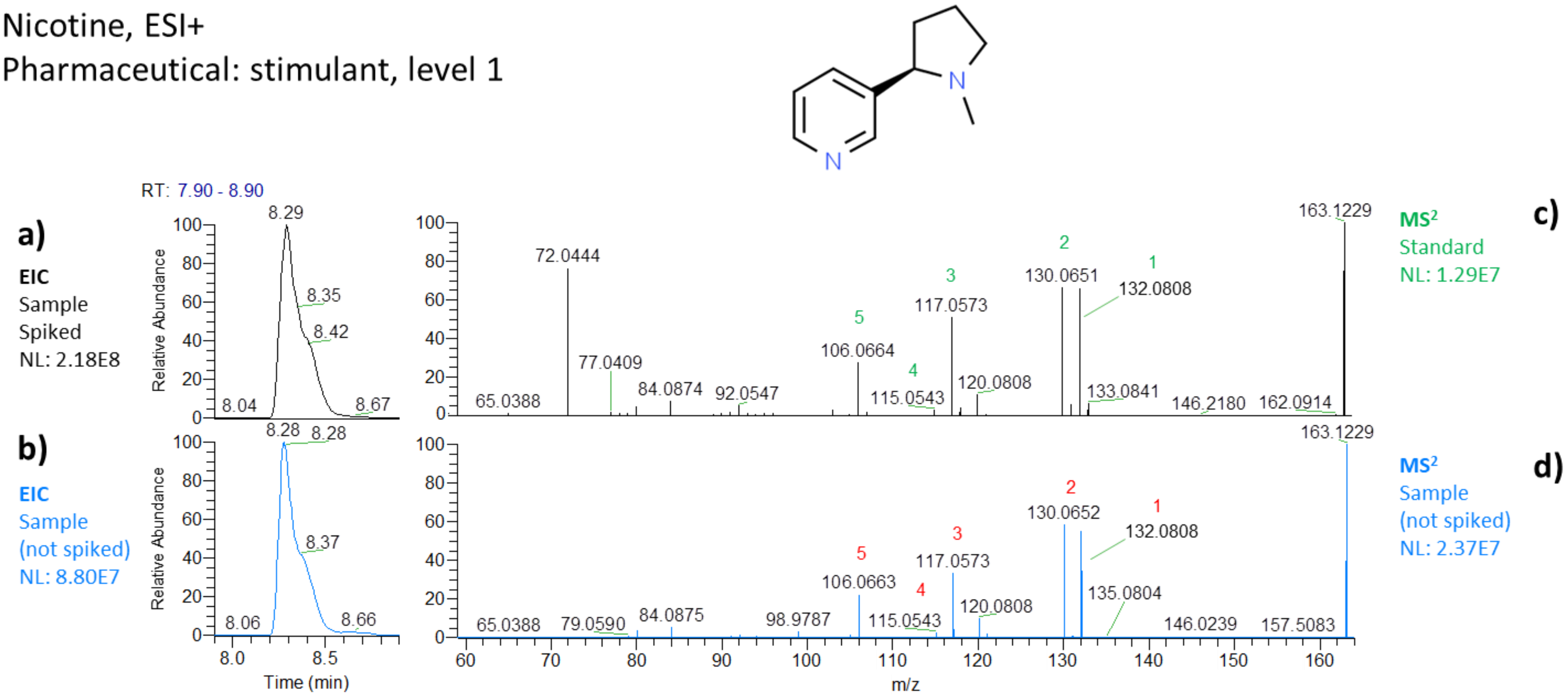

Figure S33. Confirmed identification of nicotine. Comparison of extracted ion chromatogram (EIC) between a surface water sample a) spiked with nicotine at 5 µg/L and b) not spiked, and of MS2 data from c) authentic nicotine standard and d) a surface water sample not spiked, analysed under the same conditions. Mass deviation and references to the literature related to the fragments flagged with numbers are available in Table S33. NL: normalized level.

Table S33. Nicotine fragments expected chemical formula, theoretical and measured masses, and related mass deviation, from the DDA MS2 of the standard and sample reinjected for confirmation

Fragment	(Expected) Chemical formula	Theoretical mass	Measured mass for the standard	Mass error (standard) (ppm)	Measured mass for the sample	Mass error (sample) (ppm)	Reference
Parent	[C10H14N2+H]+	163.1230	163.1229	-0.61	163.1229	-0.61	
1	[C9H10N]+	132.0807	132.0808	+0.76	132.0808	+0.76	[12] MassBank EU Record: NA003449
2	[C9H8N]+	130.0651	130.0651	0.00	130.0652	+0.77	MassBank EU Record: NA003449
3	[C8H10N]+	120.0808	120.0808	0.00	120.0808	0.00	MassBank EU Record: NA003449
4	[C8H7N]+	117.0573	117.0573	0.00	117.0573	0.00	[12] MassBank EU Record: NA003449
5	[C9H7]+	115.0542	115.0543	-0.87	115.0543	-0.87	MassBank EU Record: NA003449
			106.0664		106.0663		
			84.0874		84.0875		

SI-4.3.28. Oxybenzone

Oxybenzone, ESI+

Personal care product: UV filter, level 1

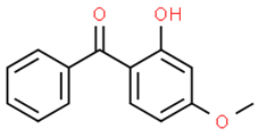

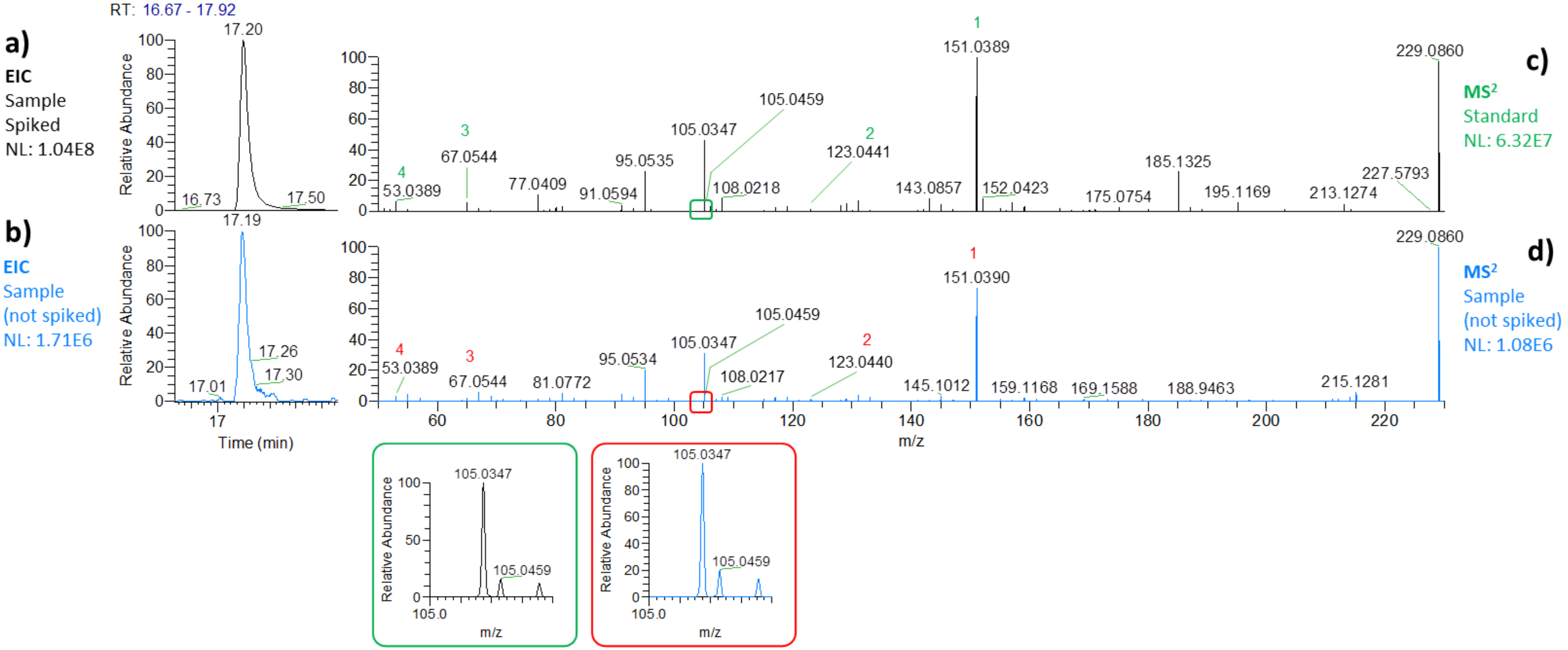

Figure S34. Confirmed identification of oxybenzone. Comparison of extracted ion chromatogram (EIC) between a surface water sample a) spiked with oxybenzone at 5 µg/L and b) not spiked, and of MS2 data from c) authentic oxybenzone standard and d) a surface water sample not spiked, analysed under the same conditions. Mass deviation and references to the literature related to the fragments flagged with numbers are available in Table S34. NL: normalized level.

Table S34. Oxybenzone fragments expected chemical formula, theoretical and measured masses, and related mass deviation, from the DDA MS2 of the standard and sample reinjected for confirmation

Fragment	(Expected) Chemical formula	Theoretical mass	Measured mass for the standard	Mass error (standard) (ppm)	Measured mass for the sample	Mass error (sample) (ppm)	Reference
Parent	$[C_{14}H_{12}O_3+H]^+$	229.0859	229.0860	+0.44	229.0860	+0.44	
1	$[C_8H_7O_3]^+$	151.0390	151.0389	-0.66	151.0390	0.00	[21] MassBank EU Record: EA023003
2	$[C_7H_7O_2]^+$	123.0441	123.0441	0.00	123.0440	-0.81	[21] MassBank EU Record: EA023003
			108.0218		108.0217		
			105.0459		105.0459		
			105.0347		105.0347		
			95.0535		95.0534		
3	$[C_5H_7]^+$	67.0542	67.0544	+2.98	67.0544	+2.98	[21] MassBank EU Record: EA023003
4	$[C_4H_5]^+$	53.0386	53.0389	+5.66	53.0389	+5.66	[21] MassBank EU Record: EA023003

SI-4.3.29. Panthenol

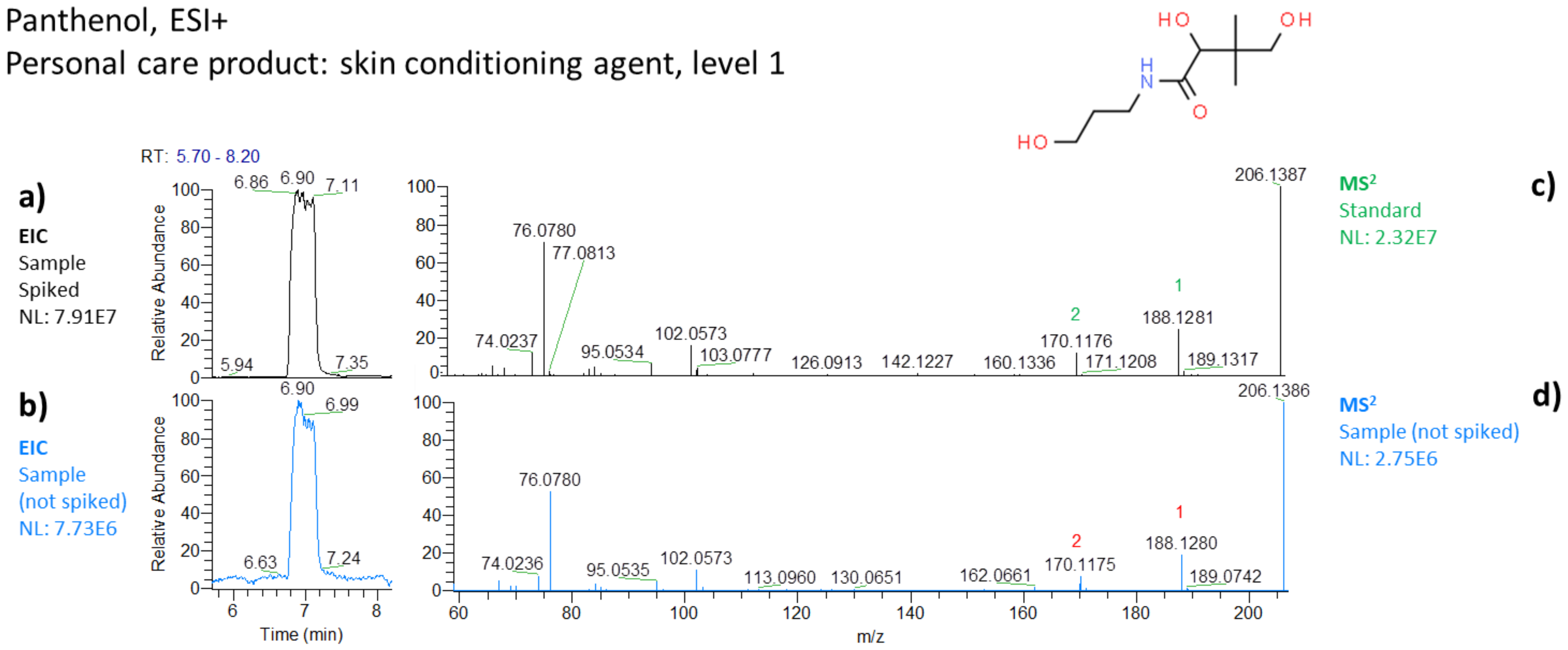

Figure S35. Confirmed identification of panthenol. Comparison of extracted ion chromatogram (EIC) between a surface water sample a) spiked with panthenol at 5 µg/L and b) not spiked, and of MS2 data from c) authentic panthenol standard and d) a surface water sample not spiked, analysed under the same conditions. Mass deviation and references to the literature related to the fragments flagged with numbers are available in Table S35. NL: normalized level.

Table S35. Panthenol fragments expected chemical formula, theoretical and measured masses, and related mass deviation, from the DDA MS2 of the standard and sample reinjected for confirmation

**Fragment**	**(Expected) Chemical formula**	**Theoretical mass**	**Measured mass for the standard**	**Mass error (standard) (ppm)**	**Measured mass for the sample**	**Mass error (sample) (ppm)**	**Reference**
Parent	[C9H19NO4+H]+	206.1387	206.1387	0.00	206.1386	-0.49	
1	[C9H18NO3]+	188.1281	188.1281	0.00	188.1280	-0.53	[22] MassBank EU Record: BML01113
2	[C9H16NO2]+	170.1176	170.1176	0.00	170.1175	-0.59	[22] MassBank EU Record: BML01113
			102.0573		102.0573		
			95.0534		95.0535		
			76.0780		76.0780		
			74.0237		74.0236		

SI-4.3.30. Paracetamol

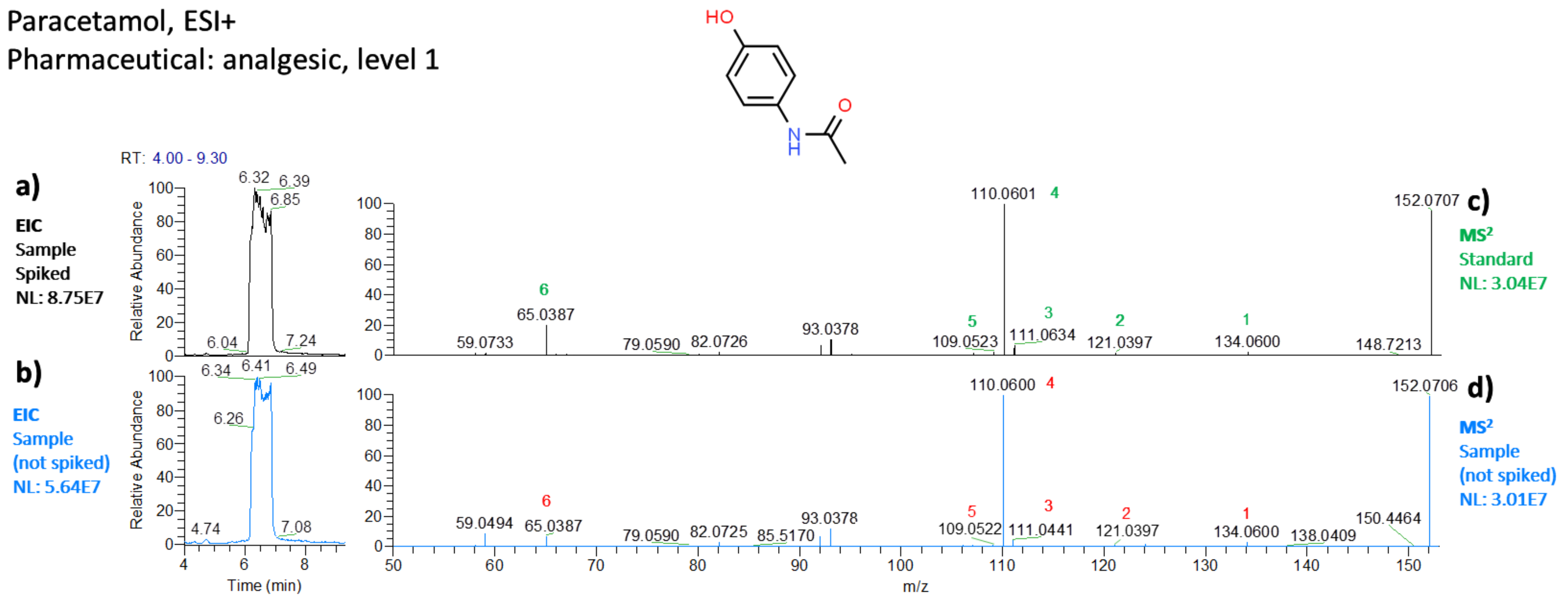

Figure S36. Confirmed identification of paracetamol. Comparison of extracted ion chromatogram (EIC) between a surface water sample a) spiked with paracetamol at 5 µg/L and b) not spiked, and of MS2 data from c) authentic paracetamol standard and d) a surface water sample not spiked, analysed under the same conditions. Mass deviation and references to the literature related to the fragments flagged with numbers are available in Table S36. NL: normalized level.

Table S36. Paracetamol fragments expected chemical formula, theoretical and measured masses, and related mass deviation, from the DDA MS2 of the standard and sample reinjected for confirmation

Fragment	(Expected) Chemical formula	Theoretical mass	Measured mass for the standard	Mass error (standard) (ppm)	Measured mass for the sample	Mass error (sample) (ppm)	Reference
Parent	[C8H9NO2+H]+	152.0706	152.0707	+0.66	157.0606	0.00	
1	[C8H8NO]+	134.0600	134.0600	0.00	134.0600	0.00	MassBank EU Record: EA024311
2	[C6H5N2O]+	121.0396	121.0397	+0.83	121.0397	+0.83	MassBank EU Record: EA024311
3	[C6H7O2]+	111.0441	111.0441	0.00	111.0441	0.00	MassBank EU Record: EA024311
4	[C6H8NO]+	110.0600	110.0601	+0.91	110.0600	0.00	[11] MassBank EU Record: EA024311
5	[C6H7NO]+	109.0522	109.0523	+0.92	109.0522	0.00	MassBank EU Record: EA024311
			93.0378		93.0378		
			82.0726		82.0725		
			79.0590		79.0590		
6	[C5H5]+	65.0386	65.0387	+1.54	65.0387	+1.54	[11] MassBank EU Record: EA024311

SI-4.3.31. Propylparaben

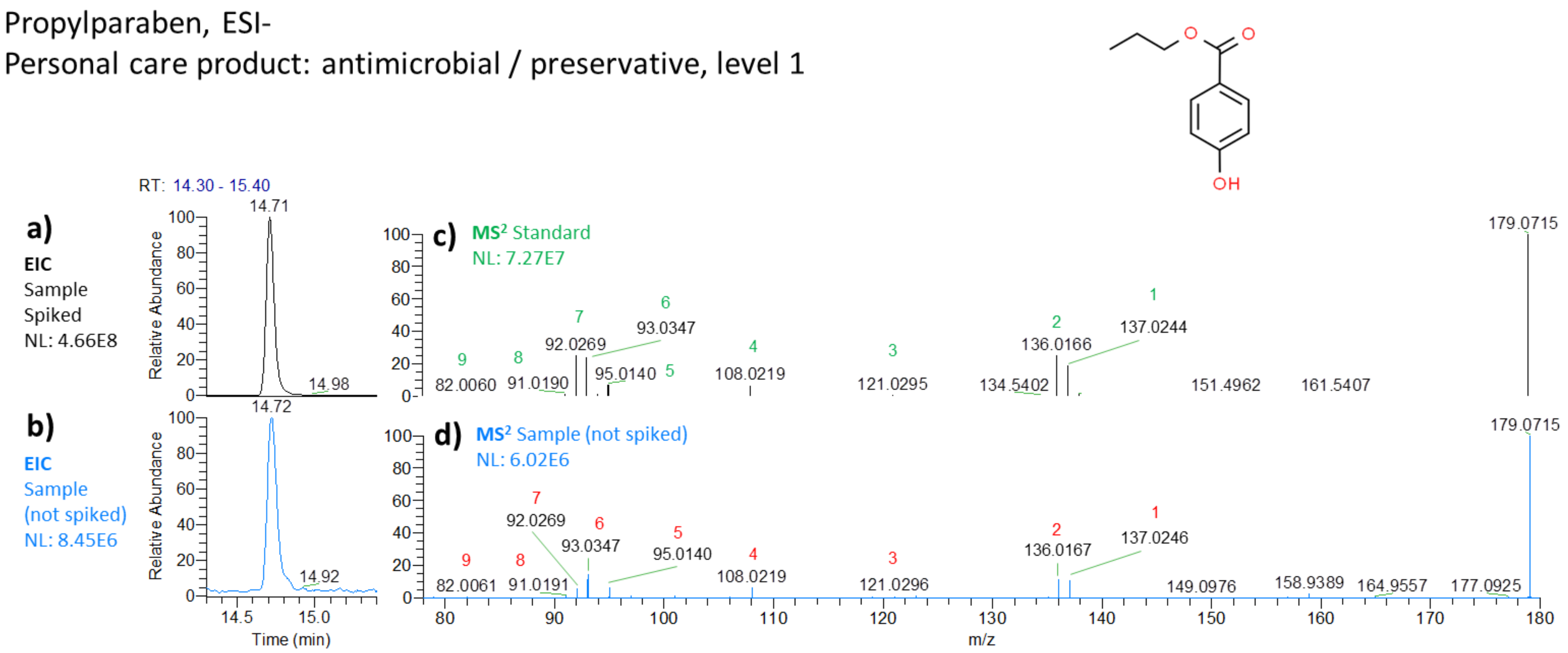

Figure S37. Confirmed identification of propylparaben. Comparison of extracted ion chromatogram (EIC) between a surface water sample a) spiked with propylparaben at 5 µg/L and b) not spiked, and of MS2 data from c) authentic propylparaben standard and d) a surface water sample not spiked, analysed under the same conditions. Mass deviation and references to the literature related to the fragments flagged with numbers are available in Table S37. NL: normalized level.

Table S37. Propylparaben fragments expected chemical formula, theoretical and measured masses, and related mass deviation, from the DDA MS2 of the standard and sample reinjected for confirmation

Fragment	(Expected) Chemical formula	Theoretical mass	Measured mass for the standard	Mass error (standard) (ppm)	Measured mass for the sample	Mass error (sample) (ppm)	Reference
Parent	[C10H12O3-H]-	179.0714	179.0715	+0.56	179.0715	+0.56	
1	[C7H5O3]-	137.0244	137.0244	0.00	137.0246	+1.46	[23] MassBank EU Record: LU098951
2	[C7H4O3]-	136.0166	136.0166	0.00	136.0167	+0.74	MassBank EU Record: LU098951
3	[C7H5O2]-	121.0295	121.0295	0.00	121.0296	+0.83	MassBank EU Record: LU098951
4	[C6H4O2]-	108.0217	108.0219	+1.85	108.0219	+1.85	MassBank EU Record: LU098951
5	[C5H3O2]-	95.0139	95.0140	+1.05	95.0140	+1.05	MassBank EU Record: LU098951
6	[C6H5O]-	93.0346	93.0347	+1.07	93.0347	+1.07	MassBank EU Record: LU098951
7	[C6H4O]-	92.0268	92.0269	+1.07	92.0269	+1.07	[23] MassBank EU Record: LU098951
8	[C6H3O]-	91.0189	91.0190	+1.10	91.0191	+2.20	MassBank EU Record: LU098951
9	[C4H2O2]-	82.0060	82.0060	0.00	82.0061	+1.22	MassBank EU Record: LU098951

SI-4.3.32. Quinoline

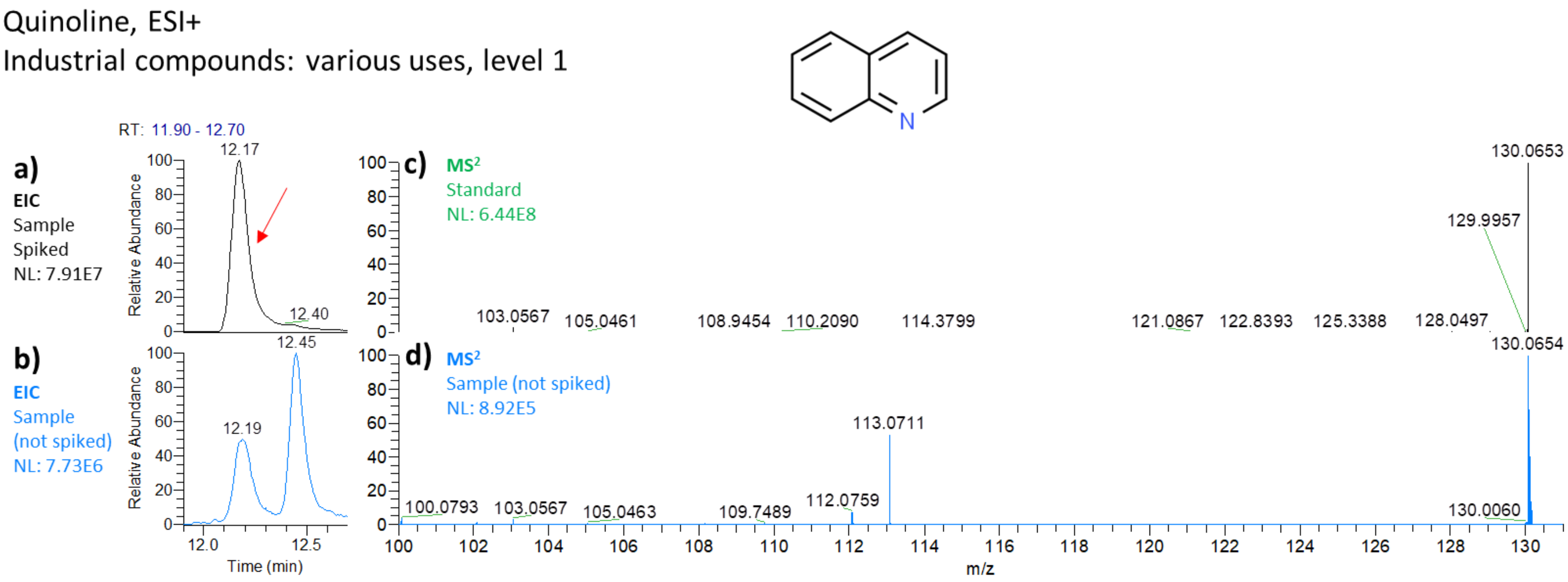

Figure S38. Confirmed identification of quinoline. Comparison of extracted ion chromatogram (EIC) between a surface water sample a) spiked with quinoline at 5 µg/L and b) not spiked, and of MS2 data from c) authentic quinoline standard and d) a surface water sample not spiked, analysed under the same conditions. Mass deviation and references to the literature related to the fragments flagged with numbers are available in Table S38. NL: normalized level.

Table S38. Quinoline fragments expected chemical formula, theoretical and measured masses, and related mass deviation, from the DDA MS2 of the standard and sample reinjected for confirmation

**Fragment**	**(Expected) Chemical formula**	**Theoretical mass**	**Measured mass for the standard**	**Mass error (standard) (ppm)**	**Measured mass for the sample**	**Mass error (sample) (ppm)**	**Reference**
Parent	[C9H7N+H]+	130.0651	130.0653	+1.54	130.0654	+2.31	
			105.0461		105.0463		
			103.0567		103.0567		

SI-4.3.33. Salbutamol

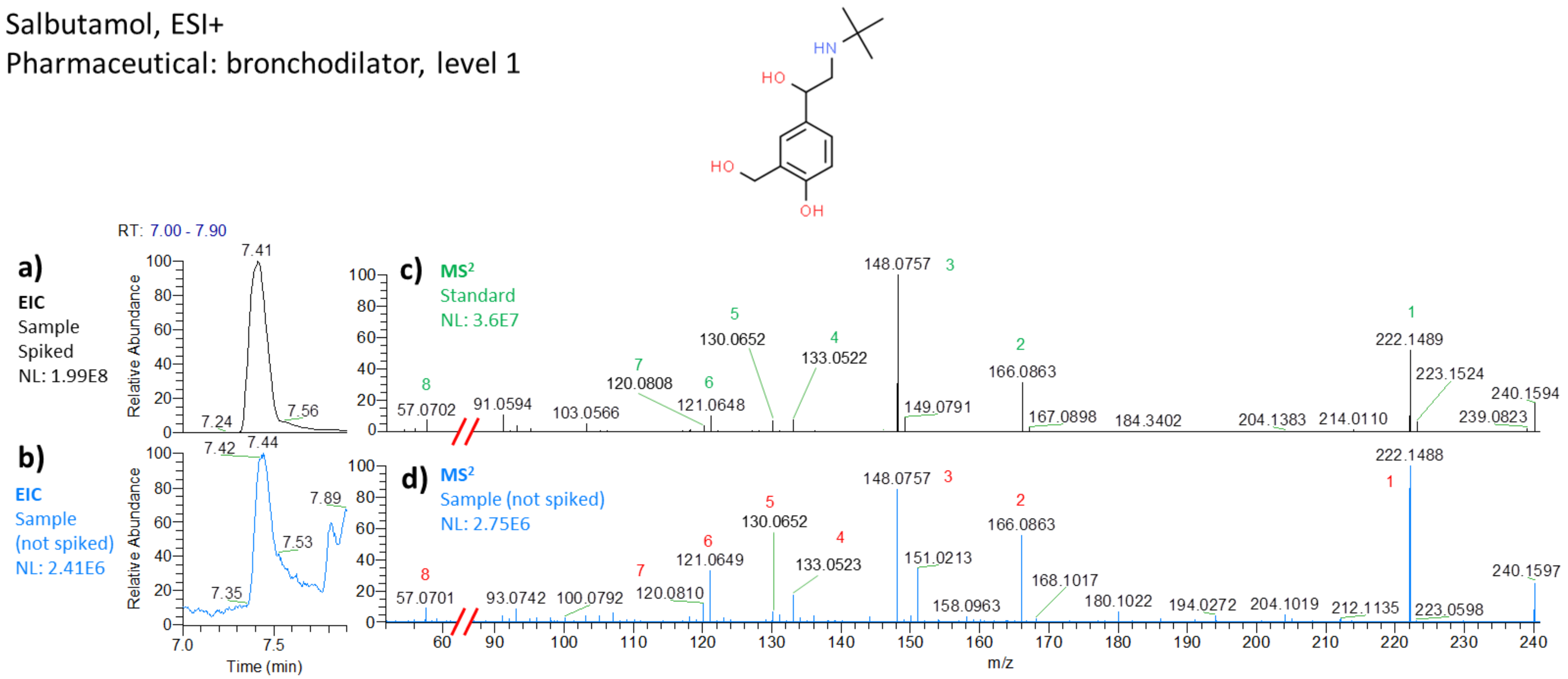

Figure S39. Confirmed identification of salbutamol. Comparison of extracted ion chromatogram (EIC) between a surface water sample a) spiked with salbutamol at 5 µg/L and b) not spiked, and of MS2 data from c) authentic salbutamol standard and d) a surface water sample not spiked, analysed under the same conditions. Mass deviation and references to the literature related to the fragments flagged with numbers are available in Table S39. NL: normalized level.

Table S39. Salbutamol fragments expected chemical formula, theoretical and measured masses, and related mass deviation, from the DDA MS2 of the standard and sample reinjected for confirmation

Fragment	(Expected) Chemical formula	Theoretical mass	Measured mass for the standard	Mass error (standard) (ppm)	Measured mass for the sample	Mass error (sample) (ppm)	Reference
Parent	[C13H21NO3+H]+	240.1594	240.1594	0.00	240.1597	+1.25	
1	[C13H20NO2]+	222.1489	222.1489	0.00	222.1488	-0.45	[11] MassBank EU Record: EA285109
2	[C9H12NO2]+	166.0863	166.0863	0.00	166.0863	0.00	[11] MassBank EU Record: EA285111
3	[C9H10NO]+	148.0757	148.0757	0.00	148.0757	0.00	[11] MassBank EU Record: EA285111
4	[C8H7NO]+	133.0522	133.0522	0.00	133.0523	+0.75	MassBank EU Record: EA285111
5	[C9H8N]+	130.0651	130.0652	+0.77	130.0652	+0.77	MassBank EU Record: EA285111
6	[C8H9O]+	121.0648	121.0648	0.00	121.0649	+0.83	MassBank EU Record: EA285111
7	[C8H10N]+	120.0808	120.0808	0.00	120.0810	+1.67	MassBank EU Record: EA285111
8	[C4H9]+	57.0699	57.0702	+5.26	57.0701	+3.50	[11] MassBank EU Record: EA285111

SI-4.3.34. Sucralose

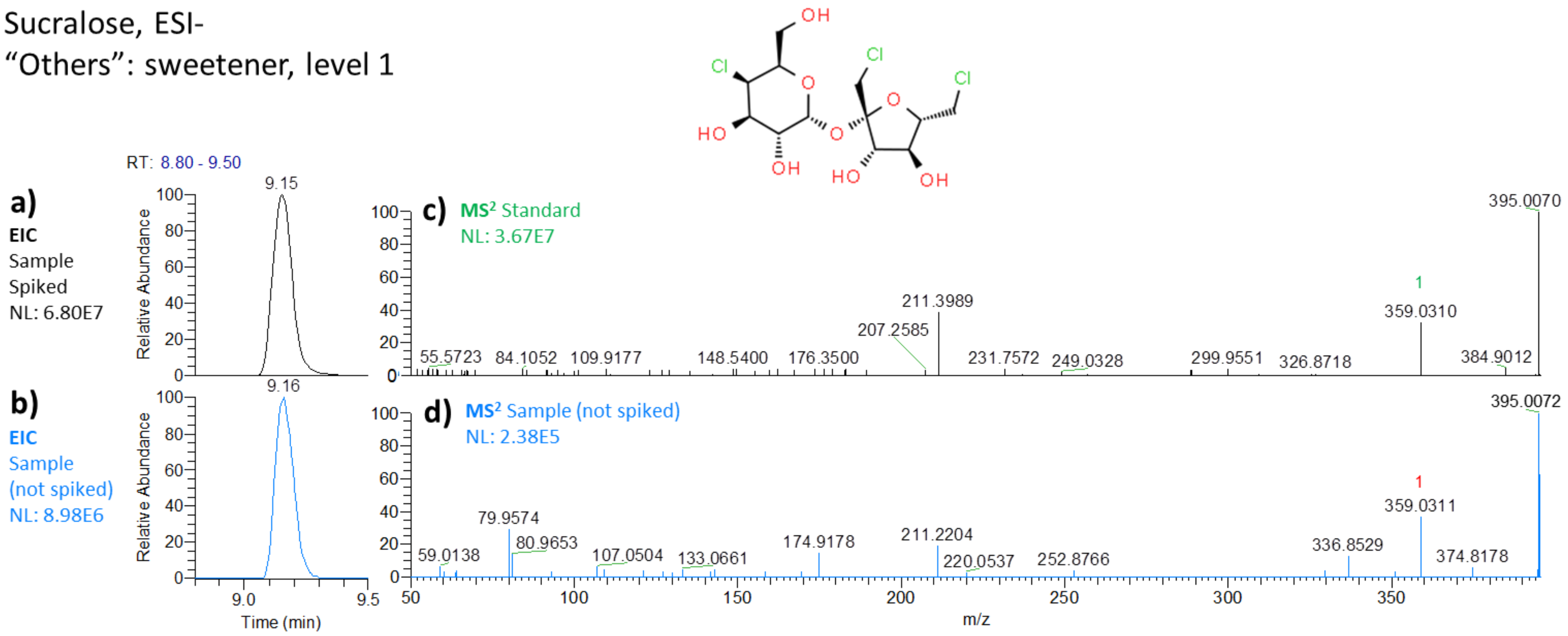

Figure S40. Confirmed identification of sucralose. Comparison of extracted ion chromatogram (EIC) between a surface water sample a) spiked with sucralose at 5 µg/L and b) not spiked, and of MS2 data from c) authentic sucralose standard and d) a surface water sample not spiked, analysed under the same conditions. Mass deviation and references to the literature related to the fragments flagged with numbers are available in Table S40. NL: normalized level.

Table S40. Sucralose fragments expected chemical formula, theoretical and measured masses, and related mass deviation, from the DDA MS2 of the standard and sample reinjected for confirmation

**Fragment**	**(Expected) Chemical formula**	**Theoretical mass**	**Measured mass for the standard**	**Mass error (standard) (ppm)**	**Measured mass for the sample**	**Mass error (sample) (ppm)**	**Reference**
Parent	[C12H19Cl3O8-H]-	395.0073	395.0070	-0.76	395.0072	-0.25	
1	[C12H17Cl2O8]-	359.0306	359.0310	+1.11	359.0311	+1.39	[24] MassBank EU Record: TUE00666

SI-4.3.35. Sulfamethazine

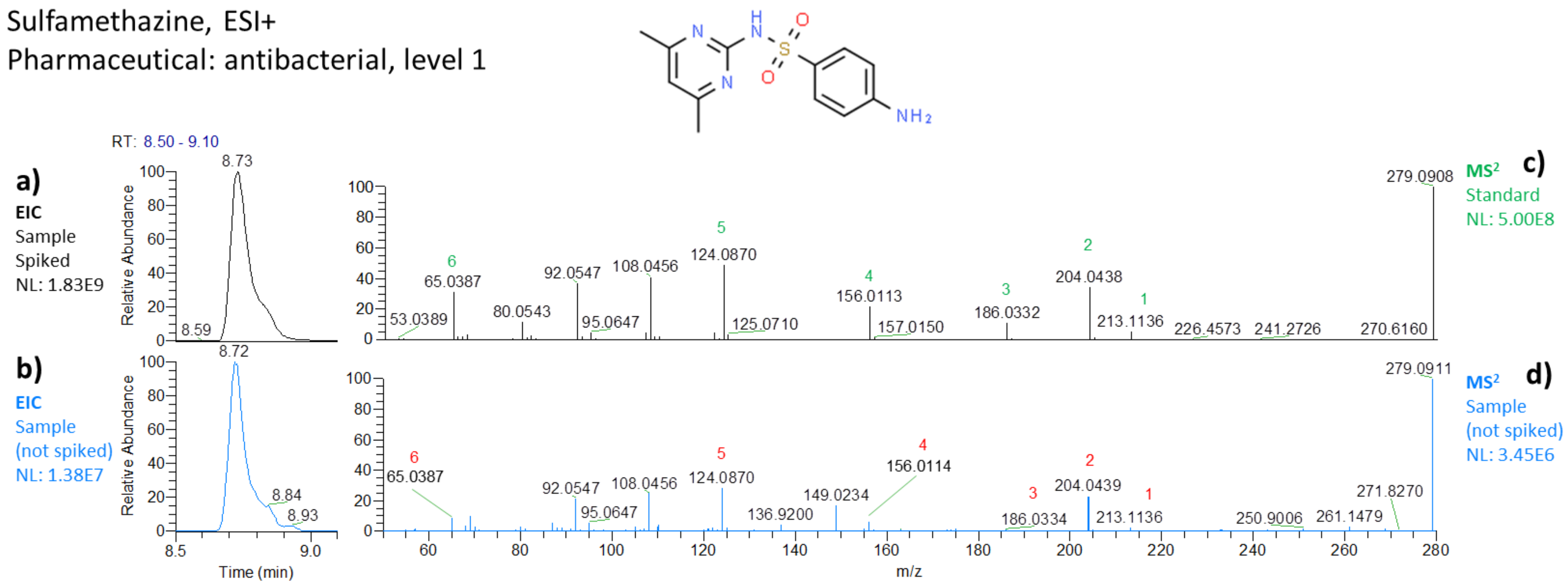

Figure S41. Confirmed identification of sulfamethazine. Comparison of extracted ion chromatogram (EIC) between a surface water sample a) spiked with sulfamethazine at 5 µg/L and b) not spiked, and of MS2 data from c) authentic sulfamethazine standard and d) a surface water sample not spiked, analysed under the same conditions. Mass deviation and references to the literature related to the fragments flagged with numbers are available in Table S41. NL: normalized level.

Table S41. Sulfamethazine fragments expected chemical formula, theoretical and measured masses, and related mass deviation, from the DDA MS2 of the standard and sample reinjected for confirmation

Fragment	(Expected) Chemical formula	Theoretical mass	Measured mass for the standard	Mass error (standard) (ppm)	Measured mass for the sample	Mass error (sample) (ppm)	Reference
Parent	[C12H14N4O2S+H]+	279.0910	279.0908	-0.72	279.0911	+0.36	
1	[C12H13N4]+	213.1135	213.1136	+0.47	213.1136	+0.47	[11] MassBank EU Record: EA018110
2	[C12H4N4]+	204.0431	204.0438	+3.43	204.0439	+3.92	MassBank EU Record: EA018110
3	[C6H8N3O2S]+	186.0332	186.0332	0.00	186.0334	+1.08	[11] MassBank EU Record: EA018110
4	[C6H6NO2S]+	156.0114	156.0113	+0.64	156.0114	0.00	[11] MassBank EU Record: EA018110
5	[C6H10N3]+	124.0869	124.0870	+0.81	124.0870	+0.81	[11] MassBank EU Record: EA018110
			108.0456		108.0456		
			95.0647		95.0647		
			92.0547		92.0547		
6	[C5H5]+	65.0386	65.0387	+1.54	65.0387	+1.54	[11] MassBank EU Record: EA018110

SI-4.3.36. Sulfamethoxazole

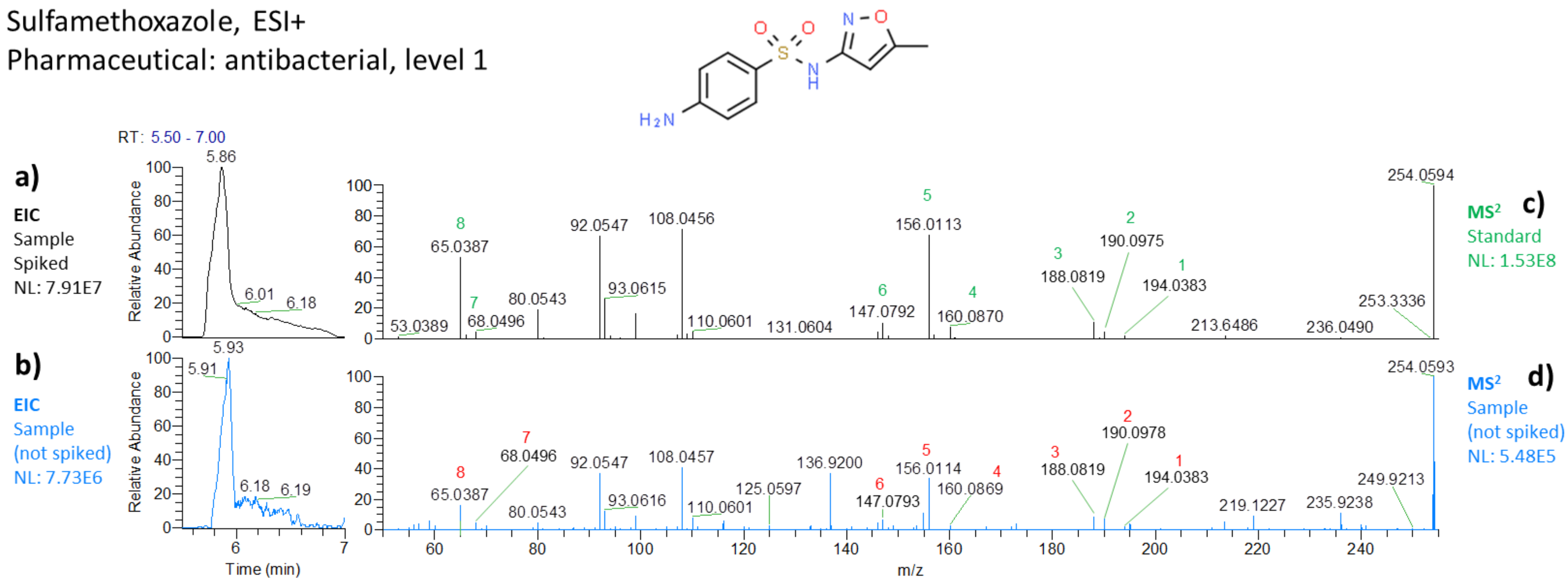

Figure S42. Confirmed identification of sulfamethoxazole. Comparison of extracted ion chromatogram (EIC) between a surface water sample a) spiked with sulfamethoxazole at 5 µg/L and b) not spiked, and of MS2 data from c) authentic sulfamethoxazole standard and d) a surface water sample not spiked, analysed under the same conditions. Mass deviation and references to the literature related to the fragments flagged with numbers are available in Table S42. NL: normalized level.

Table S42. Sulfamethoxazole fragments expected chemical formula, theoretical and measured masses, and related mass deviation, from the DDA MS2 of the standard and sample reinjected for confirmation

Fragment	(Expected) Chemical formula	Theoretical mass	Measured mass for the standard	Mass error (standard) (ppm)	Measured mass for the sample	Mass error (sample) (ppm)	Reference
Parent	[C10H11N3O3S+H]+	254.0594	254.0594	0.00	254.0593	-0.39	
1	[C8H8N3OS]+	194.0383	194.0383	0.00	194.0383	0.00	MassBank EU Record: EA029809
2	[C10H12N3O]+	190.0975	190.0975	0.00	190.0978	+1.58	MassBank EU Record: EA029809
3	[C10H10N3O]+	188.0818	188.0819	+0.53	188.0819	+0.53	[11] MassBank EU Record: EA029809
4	[C9H10N3]+	160.0869	160.0870	+0.62	160.0869	0.00	MassBank EU Record: EA029809
5	[C6H6NO2S]+	156.0114	156.0113	-0.64	156.0114	0.00	[11] MassBank EU Record: EA029809
6	[C8H9N3]+	147.0791	147.0792	+0.68	147.0793	+1.36	MassBank EU Record: EA029809
			110.0601		110.0601		
			108.0456		108.0457		
			93.0615		93.0616		
			92.0547		92.0547		
			80.0543		80.0543		
7	[C4H6N]+	68.0495	68.0496	+1.47	68.0496	+1.47	MassBank EU Record: EA029809
8	[C5H5N]+	65.0386	65.0387	+1.54	65.0387	+1.54	MassBank EU Record: EA029809

SI-4.3.37. Triclosan

No fragmentation is observed at the collision energy applied for the standard. Therefore, the fragments observed in the samples might come from parent with mass 286.9688 considering the DIA minimum window for parent mass selection is 0.04 Da. The retention time fitting very well between the sample not spiked and the sample spiked, and the detection of triclosan specific isotopic pattern in the sample MS1, with very low mass deviation, led us to confirm the detection of triclosan in the samples with level 1 confidence.

Triclosan, ESI-

Personal care product: antimicrobial / preservative, level 1

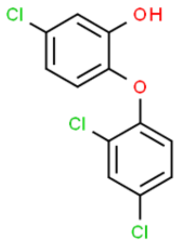

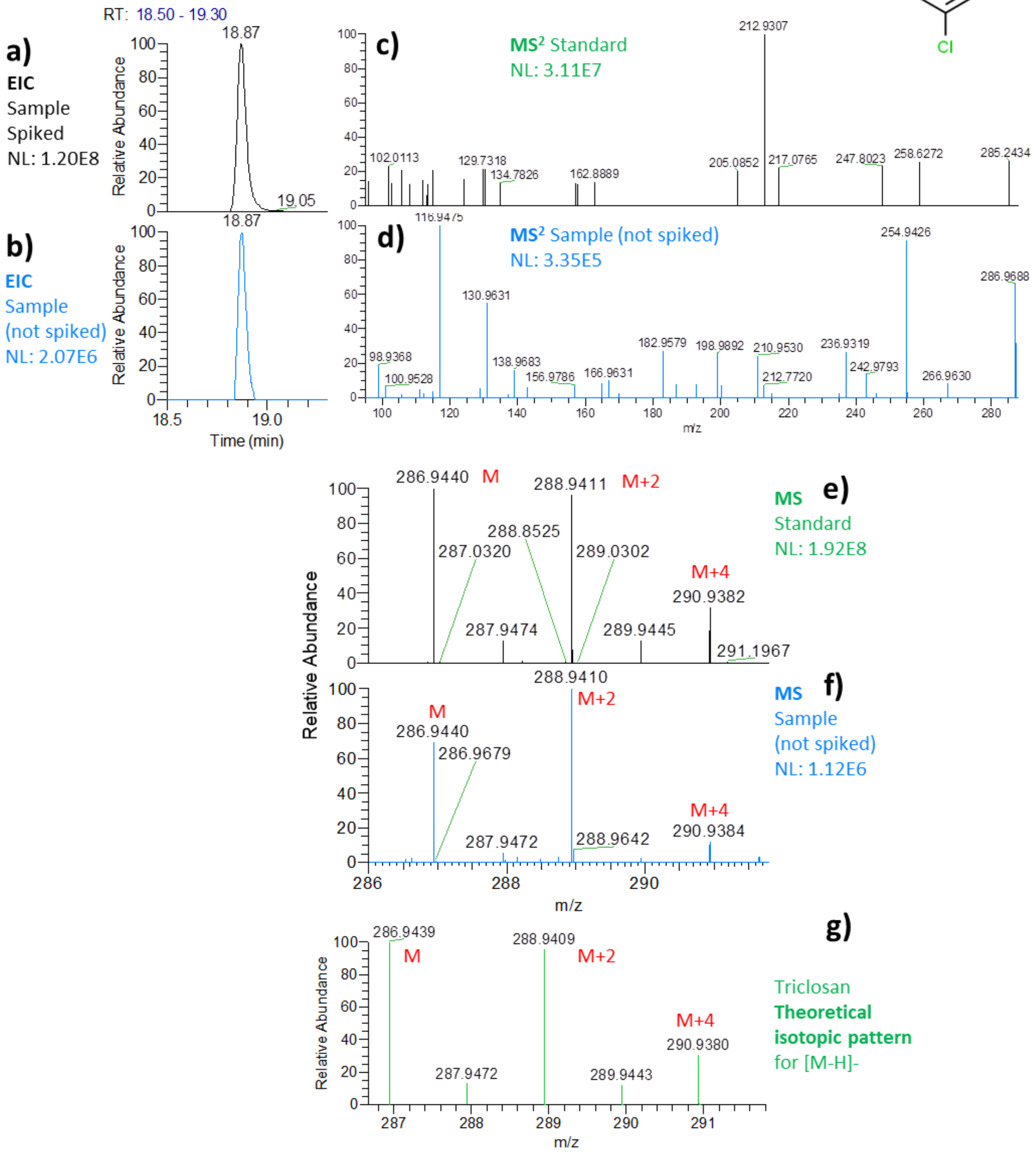

Figure S43. Confirmed identification of triclosan. Comparison of extracted ion chromatogram (EIC) between a surface water sample a) spiked with triclosan at 5 µg/L and b) not spiked, and of MS2 data from c) authentic triclosan standard and d) a surface water sample not spiked, analysed under the same conditions. Mass deviation and references to the literature related to the fragments flagged with numbers are available in Table S43. NL: normalized level.

Table S43. Triclosan isotopic pattern theoretical and measured masses, and their related mass deviation, the standard injection and the sample reinjected for confirmation.

Isotopic pattern	(Expected) Chemical formula	Theoretical mass	Measured mass for the standard	Mass error (standard) (ppm)	Measured mass for the sample	Mass error (sample) (ppm)
[M-H]- i.e. M	[C12H7Cl3O2-H]-	286.9439	286.9440	+0.35	286.9440	+0.35
M+2	[C12H7Cl2[37Cl]O2-H]-	288.9409	288.9411	+0.69	288.9410	+0.35
M+4	[C12H7Cl[37Cl]2O2-H]-	290.9380	290.9382	+0.69	290.9384	+1.37

SI-4.3.38. Trimethoprim

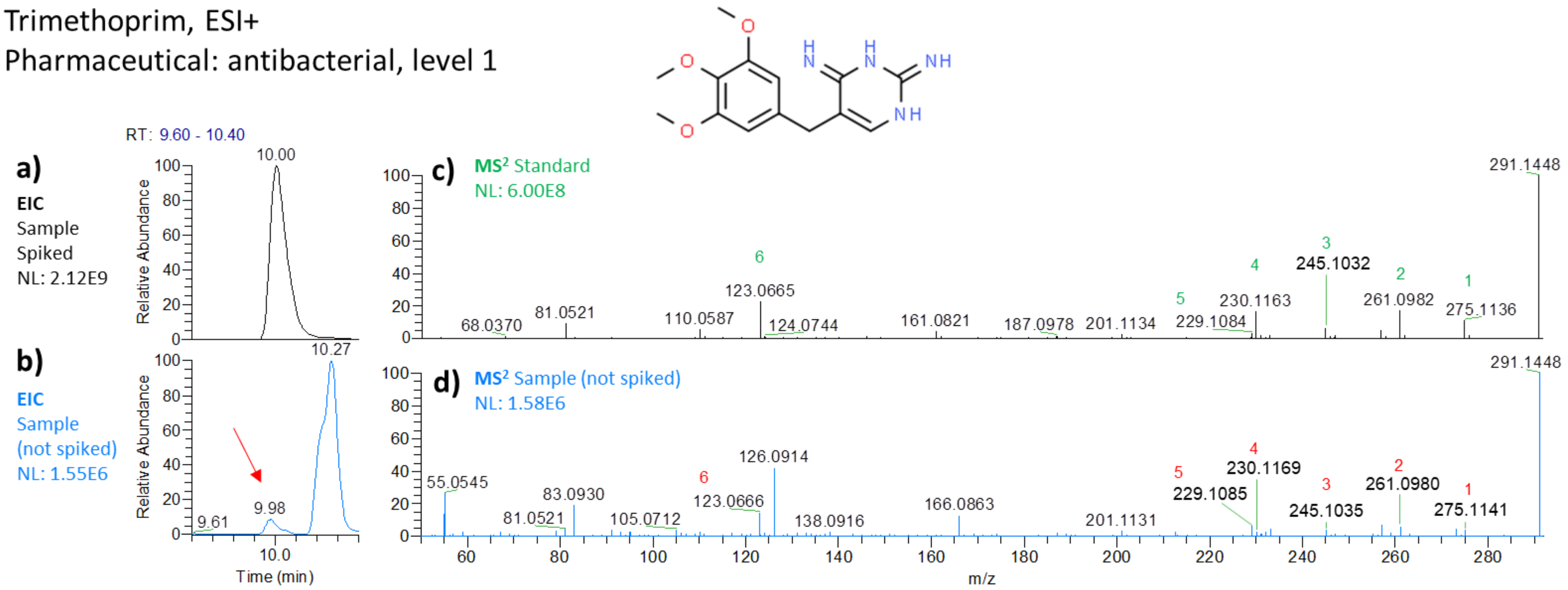

Figure S44. Confirmed identification of trimethoprim. Comparison of extracted ion chromatogram (EIC) between a surface water sample a) spiked with trimethoprim at 5 µg/L and b) not spiked, and of MS2 data from c) authentic trimethoprim standard and d) a surface water sample not spiked, analysed under the same conditions. Mass deviation and references to the literature related to the fragments flagged with numbers are available in Table S44. NL: normalized level.

Table S44. Trimethoprim fragments expected chemical formula, theoretical and measured masses, and related mass deviation, from the DDA MS2 of the standard and sample reinjected for confirmation

Fragment	(Expected) Chemical formula	Theoretical mass	Measured mass for the standard	Mass error (standard) (ppm)	Measured mass for the sample	Mass error (sample) (ppm)	Reference
Parent	[C14H18N4O3+H]+	291.1452	291.1448	-1.37	291.1448	-1.37	
1	[C13H15N4O3]+	275.1139	275.1136	-1.09	275.1141	+0.73	[11] MassBank EU Record: EA019910
2	[C12H13N4O3]+	261.0982	261.0982	0.00	261.0980	-0.77	[11] MassBank EU Record: EA019910
3	[C12H13N4O2]+	245.1033	245.1032	-0.41	245.1035	-0.82	MassBank EU Record: EA019910
4	[C12H14N4O]+	230.1162	230.1163	+0.43	230.1169	+3.04	[11] MassBank EU Record: EA019910
5	[C12H13N4O]+	229.1084	229.1084	0.00	229.1085	+0.44	MassBank EU Record: EA019910
6	[C5H7N4]+	123.0665	123.0665	0.00	123.0666	+0.81	[11] MassBank EU Record: EA019910

SI-4.3.39. Tris(2-butoxyethyl) phosphate

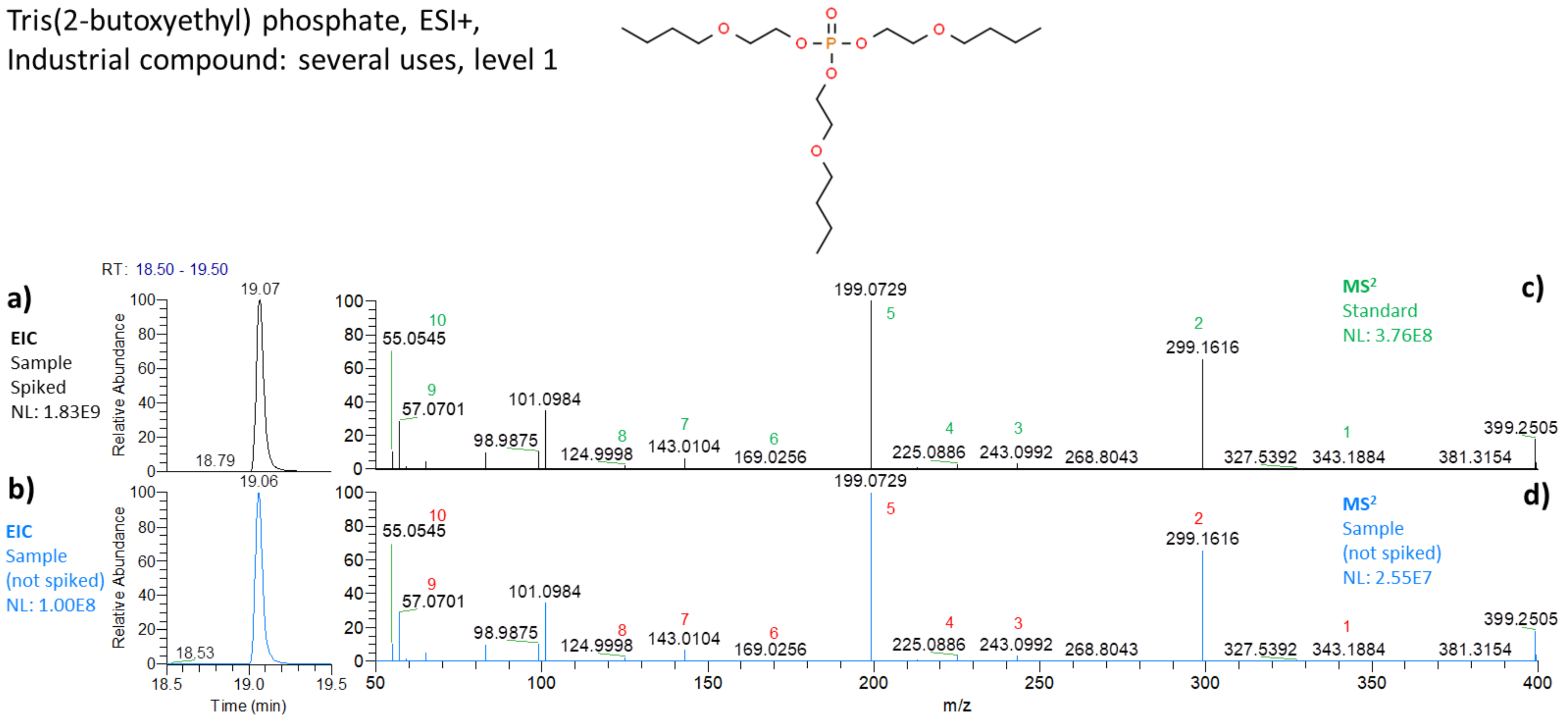

Figure S45. Confirmed identification of tris(2-butoxyethyl) phosphate. Comparison of extracted ion chromatogram (EIC) between a surface water sample a) spiked with tris(2-butoxyethyl) phosphate at 5 µg/L and b) not spiked, and of MS^2 data from c) authentic tris(2-butoxyethyl) phosphate standard and d) a surface water sample not spiked, analysed under the same conditions. Mass deviation and references to the literature related to the fragments flagged with numbers are available in Table S45. NL: normalized level.

Table S45. Tris(2-butoxyethyl) phosphate fragments expected chemical formula, theoretical and measured masses, and related mass deviation, from the DDA MS^2 of the standard and sample reinjected for confirmation

Fragment	(Expected) Chemical formula	Theoretical mass	Measured mass for the standard	Mass error (standard) (ppm)	Measured mass for the sample	Mass error (sample) (ppm)	Reference
Parent	[C18H39O7P+H]+	399.2506	399.2505	-0.25	399.2505	-0.25	
1	[C14H32O7P]+	343.1880	343.1884	+1.17	343.1884	+1.17	MassBank EU Record: SM880602
2	[C12H28O6P]+	299.1618	299.1616	-0.67	299.1616	-0.67	[25] MassBank EU Record: SM880602
			268.8043		268.8043		
3	[C8H20O6P]+	243.0992	243.0992	0.00	243.0992	0.00	MassBank EU Record: SM880602
4	[C8H18O5P]+	225.0886	225.0886	0.00	225.0886	0.00	MassBank EU Record: SM880602
5	[C6H16O5P]+	199.0730	199.0729	-0.50	199.0729	-0.50	[25] MassBank EU Record: SM880602
6	[C4H10O5P]+	169.0260	169.0256	-2.37	169.0256	-2.37	MassBank EU Record: SM880602
7	[C2H8O5P]+	143.0104	143.0104	0.00	143.0104	0.00	[25] MassBank EU Record: SM880602
8	[C2H6O4P]+	124.9998	124.9998	0.00	124.9998	0.00	MassBank EU Record: SM880602
			101.0984		101.0984		
			98.9875		98.9875		
9	[C4H9]+	57.0699	57.0701	+3.50	57.0701	+3.50	MassBank EU Record: SM880602
10	[C4H7]+	55.0542	55.0545	+5.45	55.0545	+5.45	MassBank EU Record: SM880602

SI-4.4. Level 2 annotations and there MS/MS spectrum match

SI-4.4.1. Pharmaceuticals

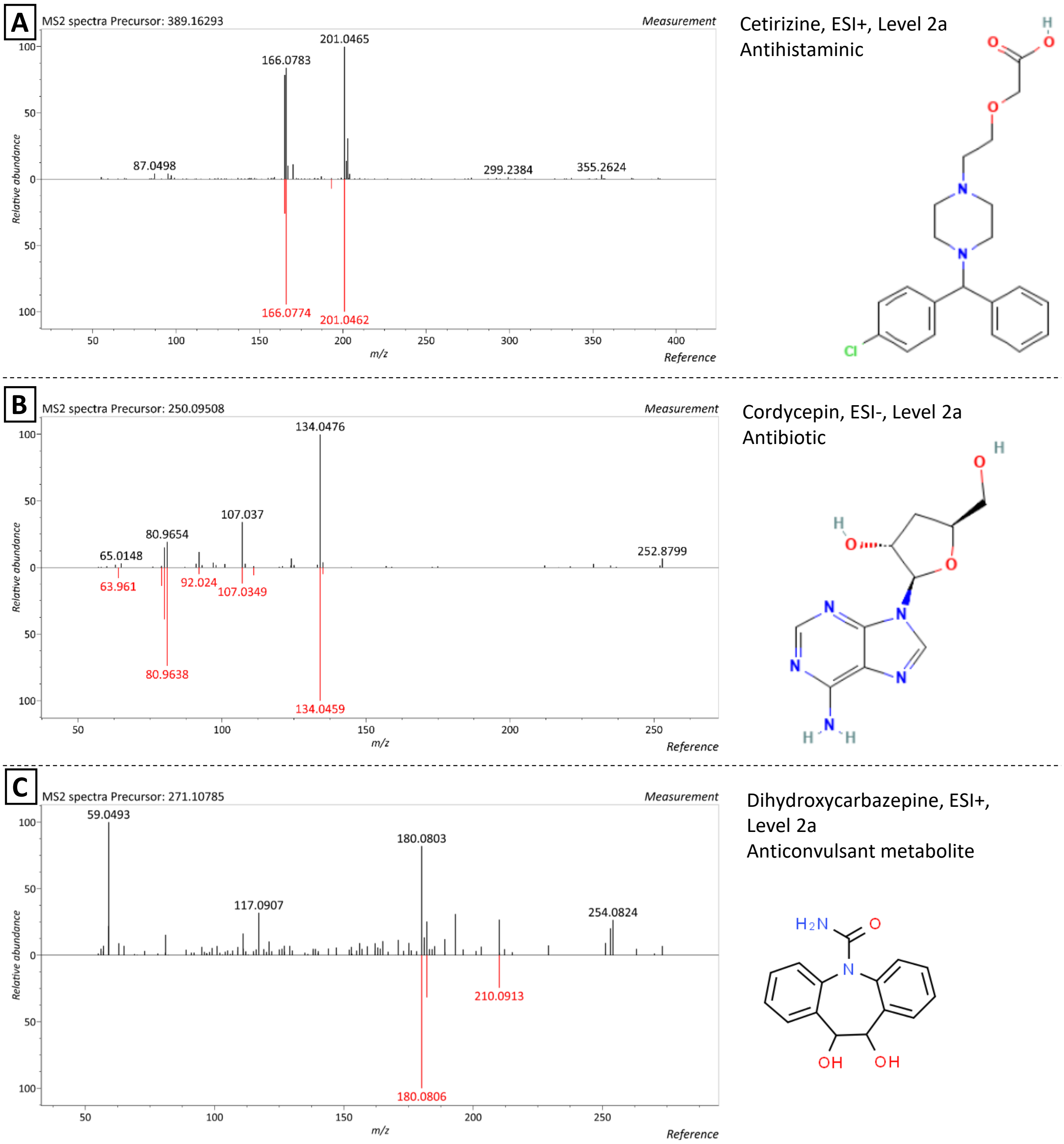

Figure S46. MS/MS spectrum match between the samples (top) and the MS/MS library (bottom) for the level 2a identifications of A) cetirizine, B) cordycepin, and C) dihydroxycarbazepine

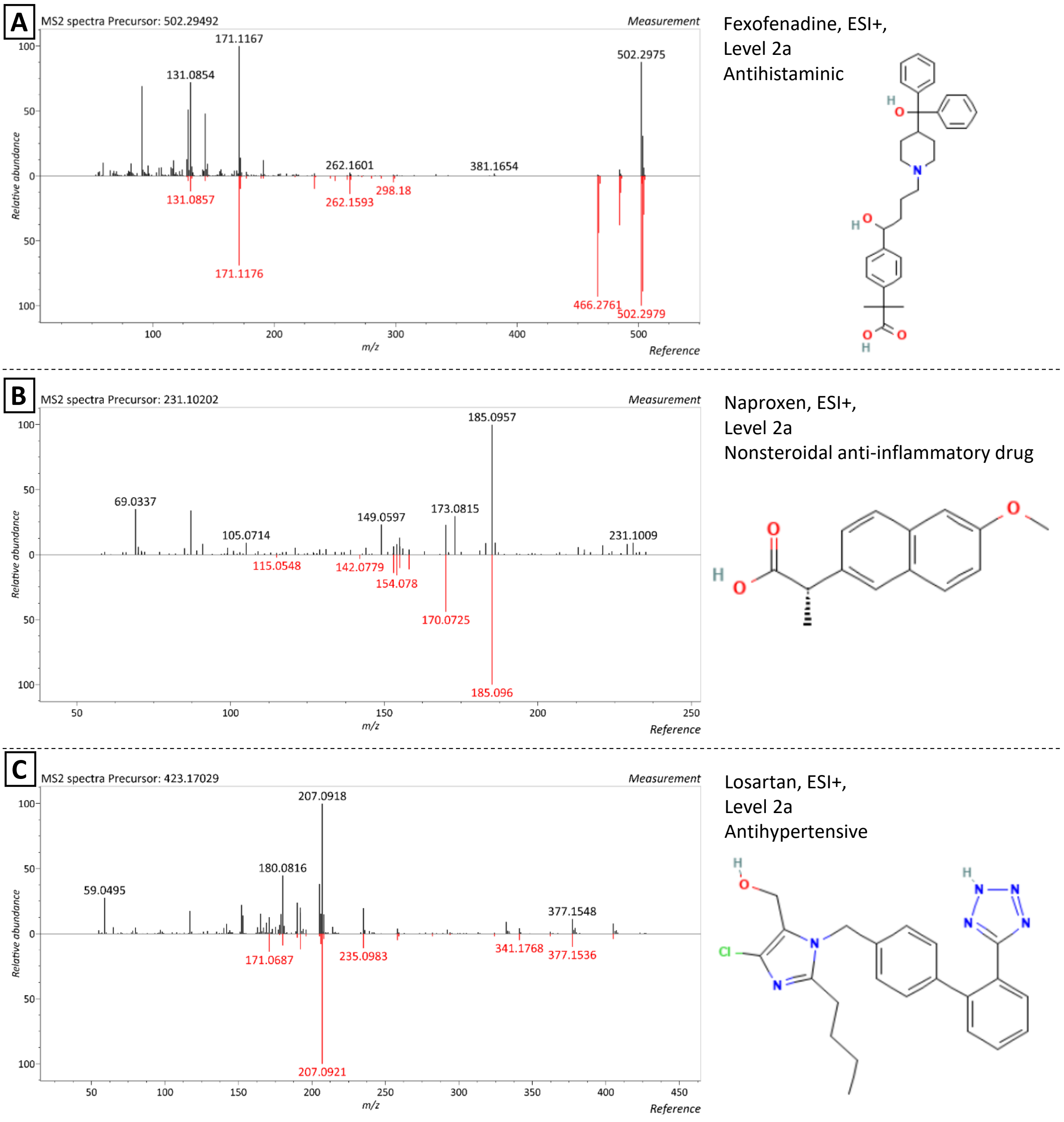

Figure S47. MS/MS spectrum match between the samples (top) and the MS/MS library (bottom) for the level 2a identifications of A) fexofenadine, B) naproxen, and C) losartan

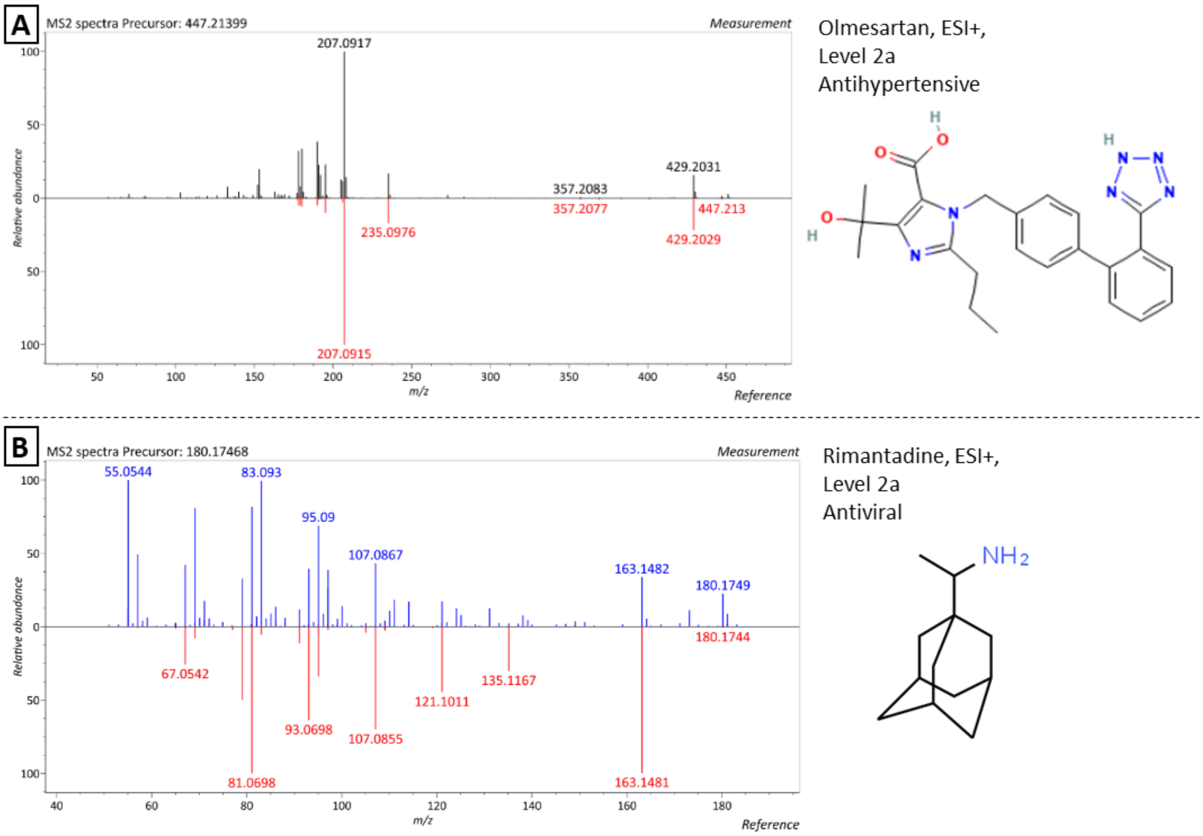

Figure S48. MS/MS spectrum match between the samples (top) and the MS/MS library (bottom) for the level 2a identifications of A) olmesartan, and B) rimantadine

SI-4.4.2. Personnal care products

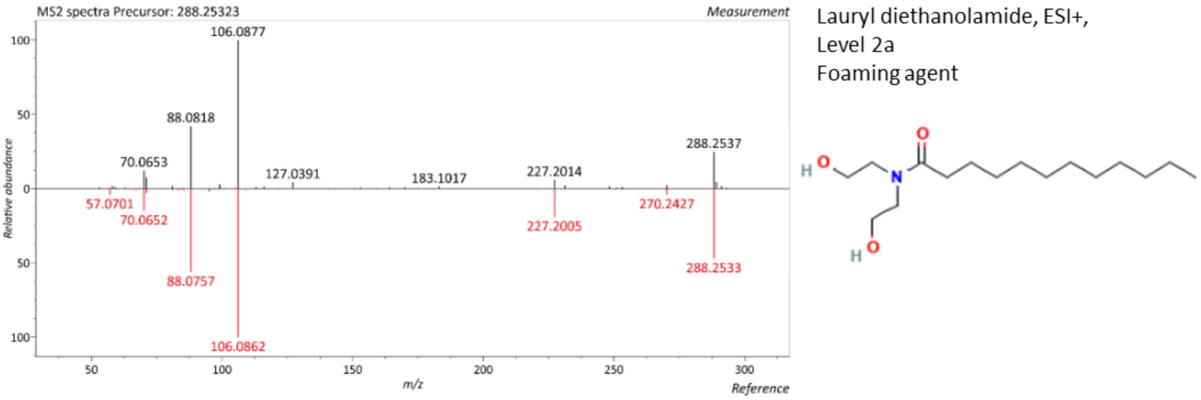

Figure S49. MS/MS spectrum match between the samples (top) and the MS/MS library (bottom) for the level 2a identifications of lauryl diethanolamide

SI-4.4.3. Industrial compounds

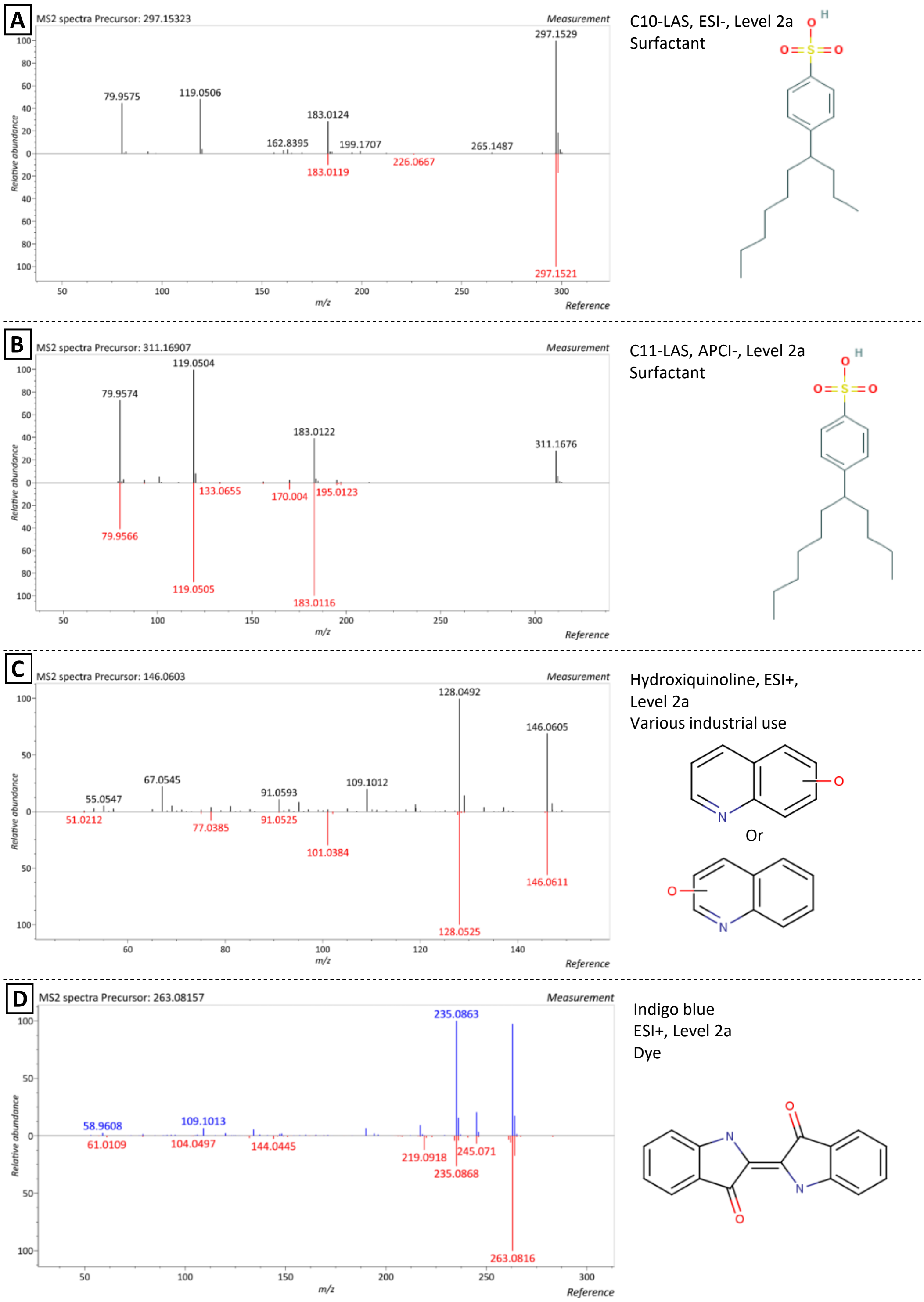

Figure S50. MS/MS spectrum match between the samples (top) and the MS/MS library (bottom) for the level 2a identifications of A) C10-LAS, B) C11-LAS, C) hydroxyquinoline, and D) indigo blue

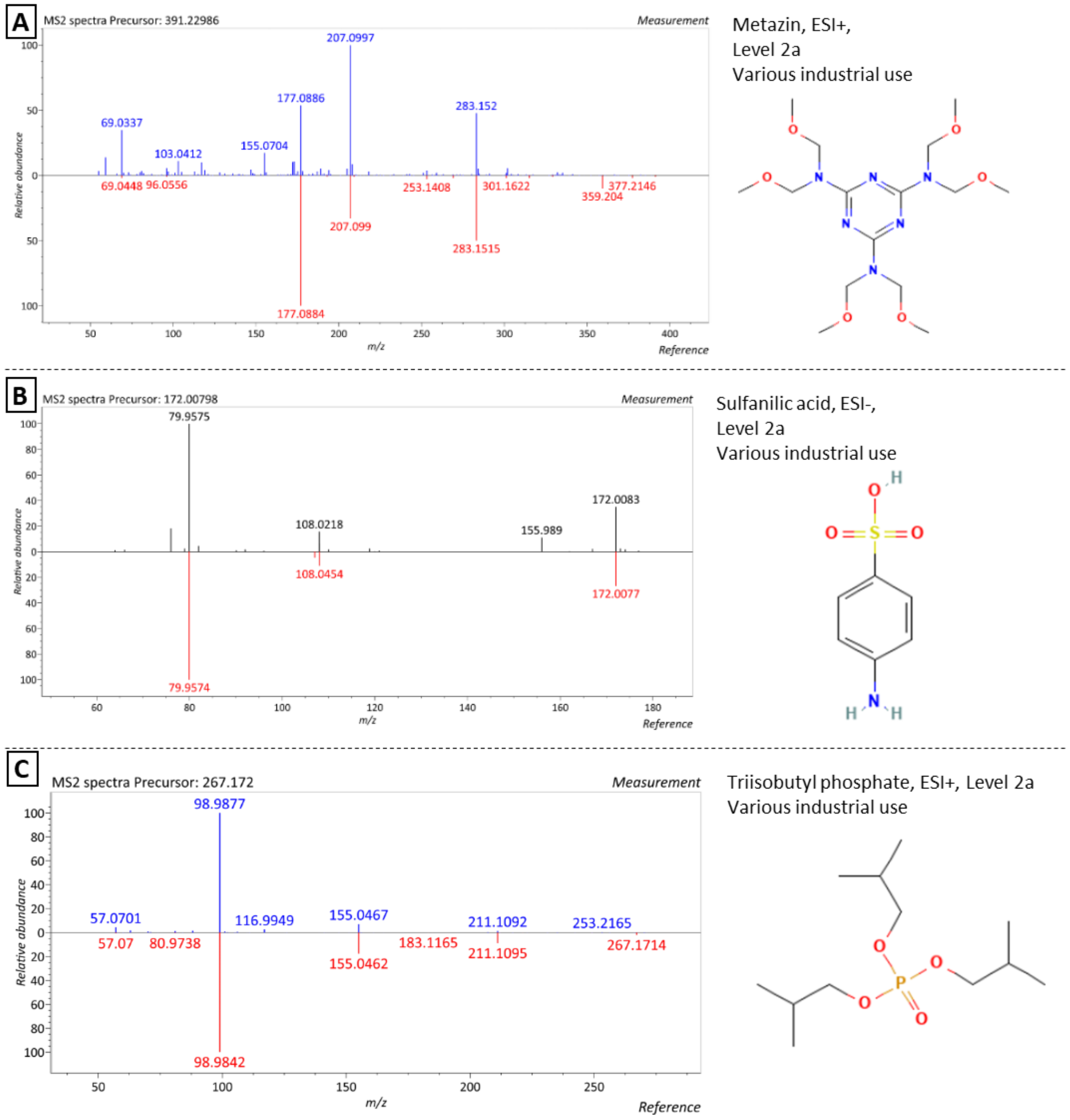

Figure S51. MS/MS spectrum match between the samples (top) and the MS/MS library (bottom) for the level 2a identifications of A) metazin, B) sulfanilic acid, and C) triisobutyl phosphate

SI-4.4.4. Miscellaneous

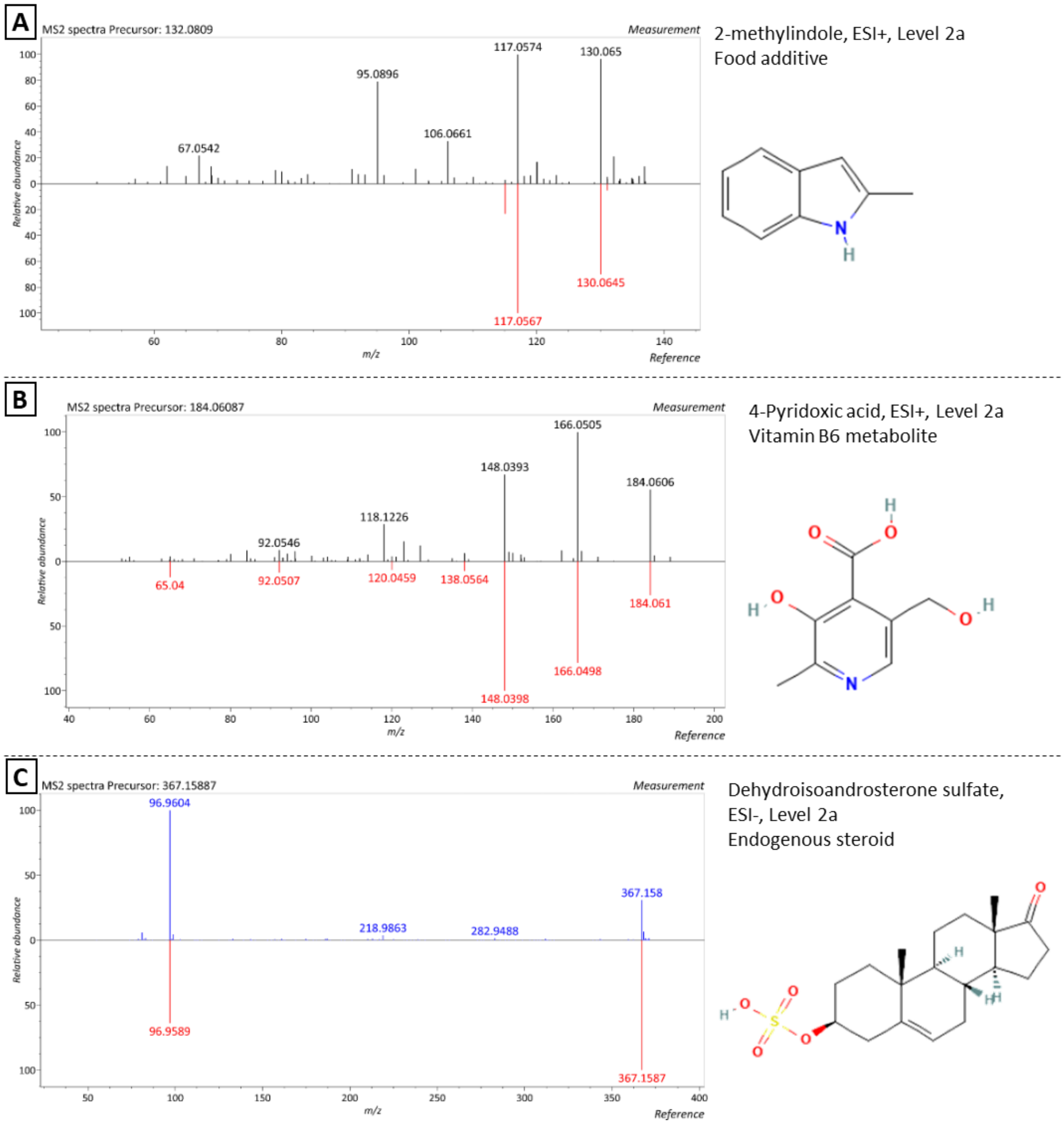

Figure S52. MS/MS spectrum match between the samples (top) and the MS/MS library (bottom) for the level 2a identifications of A) 2-methylindole, B) 4-pyridoxic acid, and C) dehydroisoandrosterone sulfate

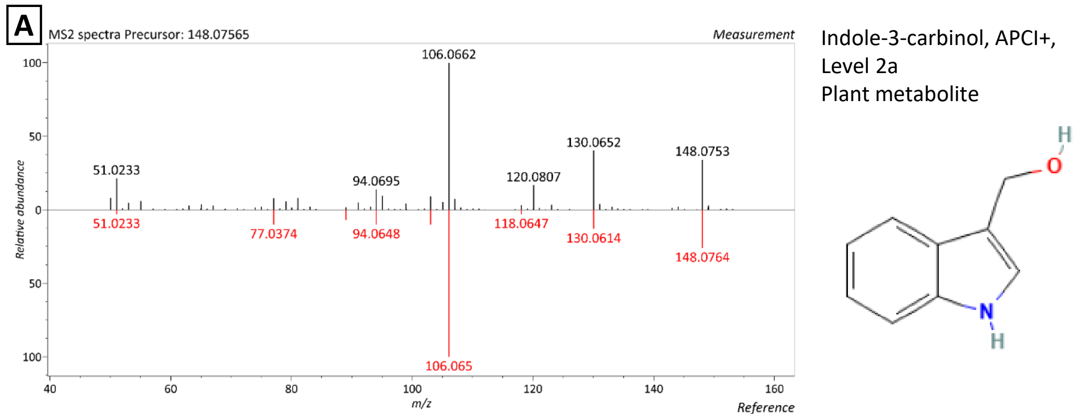

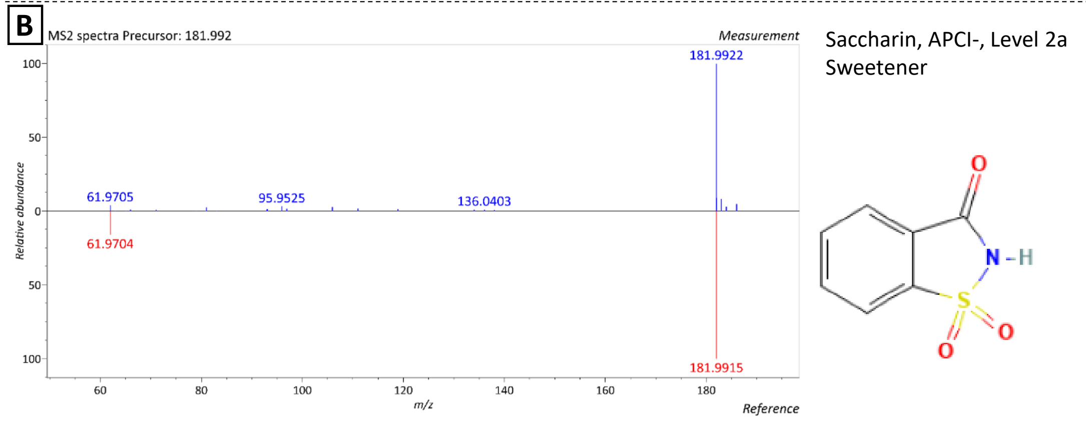

Figure S53. MS/MS spectrum match between the samples (top) and the MS/MS library (bottom) for the level 2a identifications of A) indole-3-carbinol, and B) saccharin

SI-4.5. Annotated compounds' correlation with sampling sites and detection trends

Table S46. Annotated and identified compounds correlated with the urban samples (VIP > 1 and $p_{(corr)}$ > 0.5 with the OPLS-DA model)

Compound	Ionization mode	VIP score (OPLS-DA model)	$p_{(corr)}$ (OPLS-DA model)
1-Naphthalenesulfonic acid	APCI-	1.117	0.819
2-Naphthalenesulfonic acid	ESI-	1.109	0.807
	APCI-	1.136	0.827
4- and 5-Methyl-1H-benzotriazole	ESI+	1.023	0.740
	ESI-	1.107	0.812
	APCI-	1.257	0.896
4-Pyridoxic acid	ESI-	1.107	0.804
	ESI+	1.177	0.857
Acesulfame	APCI-	1.067	0.768
	APCI-	1.197	0.876
Aspirin	APCI+	1.161	0.855
Atenolol	APCI+	1.103	0.765
	ESI+	1.205	0.893
Bis(2-ethylhexyl)phosphate	APCI+	1.021	0.724
	APCI-	1.124	0.801
	ESI+	1.147	0.811
	ESI-	1.278	0.939
C10-LAS	APCI-	1.224	0.888
	ESI-	1.289	0.952
C11-LAS	ESI-	1.314	0.968
	APCI-	1.351	0.997
Caffeine	ESI+	1.320	0.972
Cetirizine	APCI-	1.038	0.748
	ESI+	1.194	0.867
	APCI+	1.348	0.994
Clarithromycin	ESI+	1.287	0.954
Cordycepin	ESI+	1.024	0.757
	ESI-	1.097	0.796
	APCI+	1.110	0.808
Cotinine	ESI+	1.088	0.791
	APCI+	1.302	0.957
Daidzein	ESI+	1.085	0.801
	ESI-	1.137	0.830
	APCI-	1.306	0.961
	APCI+	1.317	0.970
Dehydroisoandrosterone sulfate	ESI-	1.097	0.806
Diazinon	ESI+	1.147	0.839
Diclofenac	APCI+	1.146	0.840
	APCI-	1.156	0.830
	ESI+	1.184	0.861
	ESI-	1.248	0.913
Dihydroxycarbazepine	APCI+	1.234	0.918
Dimethoate	ESI+	1.057	0.748
Diphenyl phosphate	APCI+	1.046	0.765
	ESI+	1.095	0.802
	APCI-	1.147	0.843

Compound	Ionization mode	VIP score (OPLS-DA model)	$p_{(corr)}$ (OPLS-DA model)
	ESI-	1.290	0.951
Diuron	ESI+	1.187	0.879
	ESI-	1.286	0.943
Fexofenadine	APCI+	1.216	0.892
	ESI+	1.252	0.916
Fluconazole	APCI+	1.081	0.795
	APCI-	1.179	0.863
	ESI+	1.209	0.892
	ESI-	1.269	0.929
Hydroxyquinoline (OH position not known)	APCI-	1.086	0.781
	ESI+	1.118	0.788
	ESI-	1.312	0.965
Imidacloprid	APCI+	1.267	0.927
Indigo blue	ESI-	1.101	0.766
	ESI+	1.102	0.821
	APCI+	1.149	0.814
Indole-3-carbinol	APCI+	1.115	0.777
	ESI+	1.265	0.915
Losartan	APCI-	1.041	0.753
	ESI+	1.231	0.891
	APCI+	1.297	0.955
Naproxen	ESI+	1.252	0.923
Nicotine	ESI+	1.052	0.750
Olmesartan	APCI-	1.111	0.810
	APCI+	1.173	0.864
	ESI+	1.247	0.919
Paracetamol	ESI-	1.289	0.953
Propylparaben	ESI-	1.135	0.797
Quinoline	ESI+	1.037	0.763
	APCI+	1.200	0.868
Saccharin	APCI-	1.271	0.922
	ESI-	1.350	0.996
Sucralose	APCI-	1.314	0.970
Sulfamethazine	ESI+	1.044	0.751
Sulfamethoxazole	ESI+	1.128	0.821
	APCI+	1.263	0.926
Sulfanilic acid	APCI-	1.205	0.882
	ESI-	1.226	0.903
Telmisartan	APCI-	1.161	0.856
	ESI+	1.162	0.855
	ESI-	1.346	0.993
Trimethoprim	ESI-	1.001	0.715
	APCI+	1.173	0.861
	ESI+	1.180	0.849
Tris(2-butoxyethyl) phosphate	APCI+	1.260	0.914

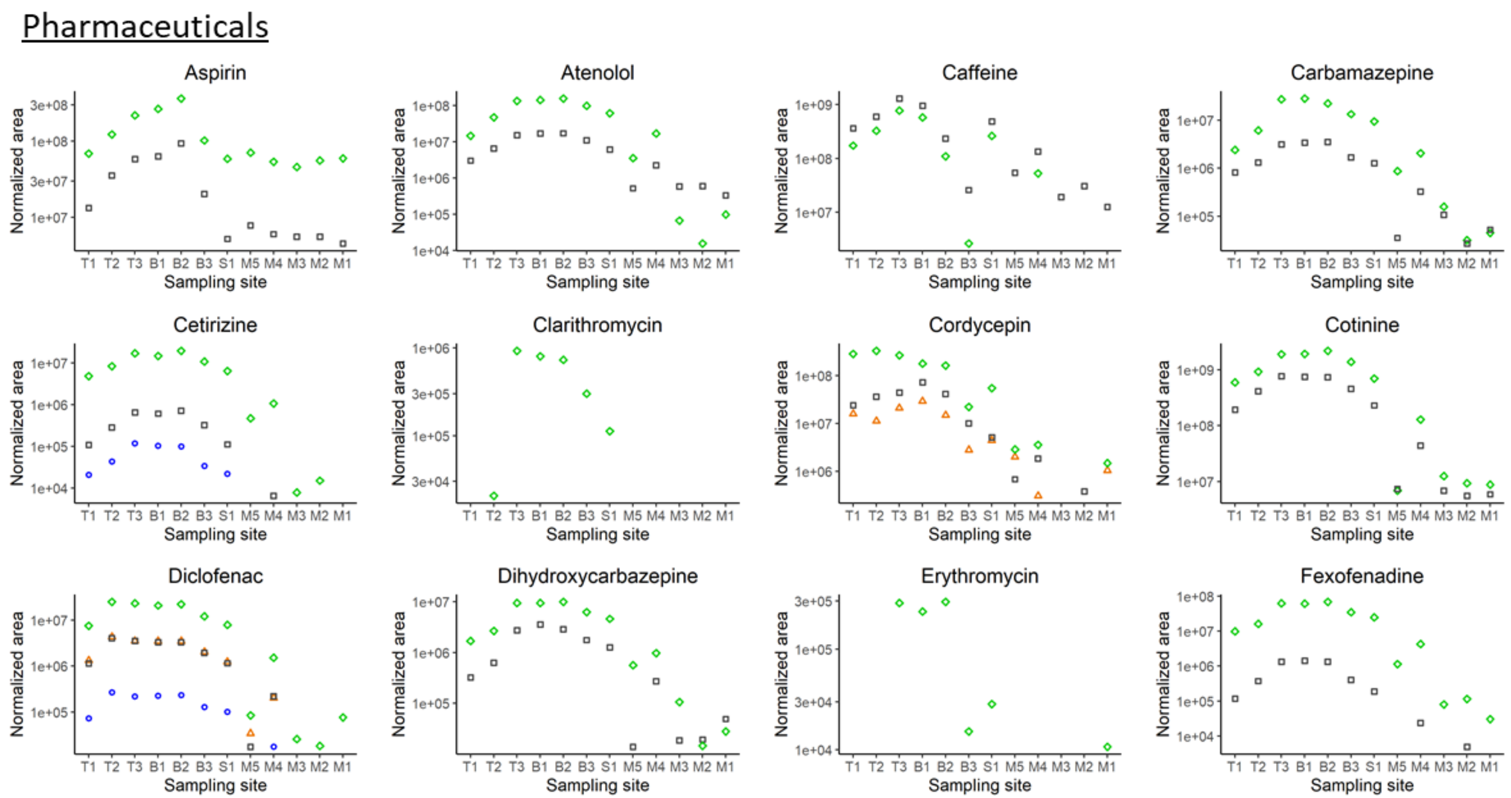

Figure S54. Distribution of the normalized area at different sampling sites for the annotated and identified compounds in the surface water samples, organized by compounds classes and alphabetic order. Sampling sites T1 to T3 are on the Turag river; B1 to B3 are on the Buriganga river; S1 is on the Shitalakshya river, and M1 to M5 are on the Meghna river. Their order on the graphs is related to their spatial distribution (see Figure S1). Blue circles correspond to APCI-, black squares to APCI+, orange triangles to ESI-, and green diamonds to ESI+.

<u>Pharmaceuticals</u> - *continued*

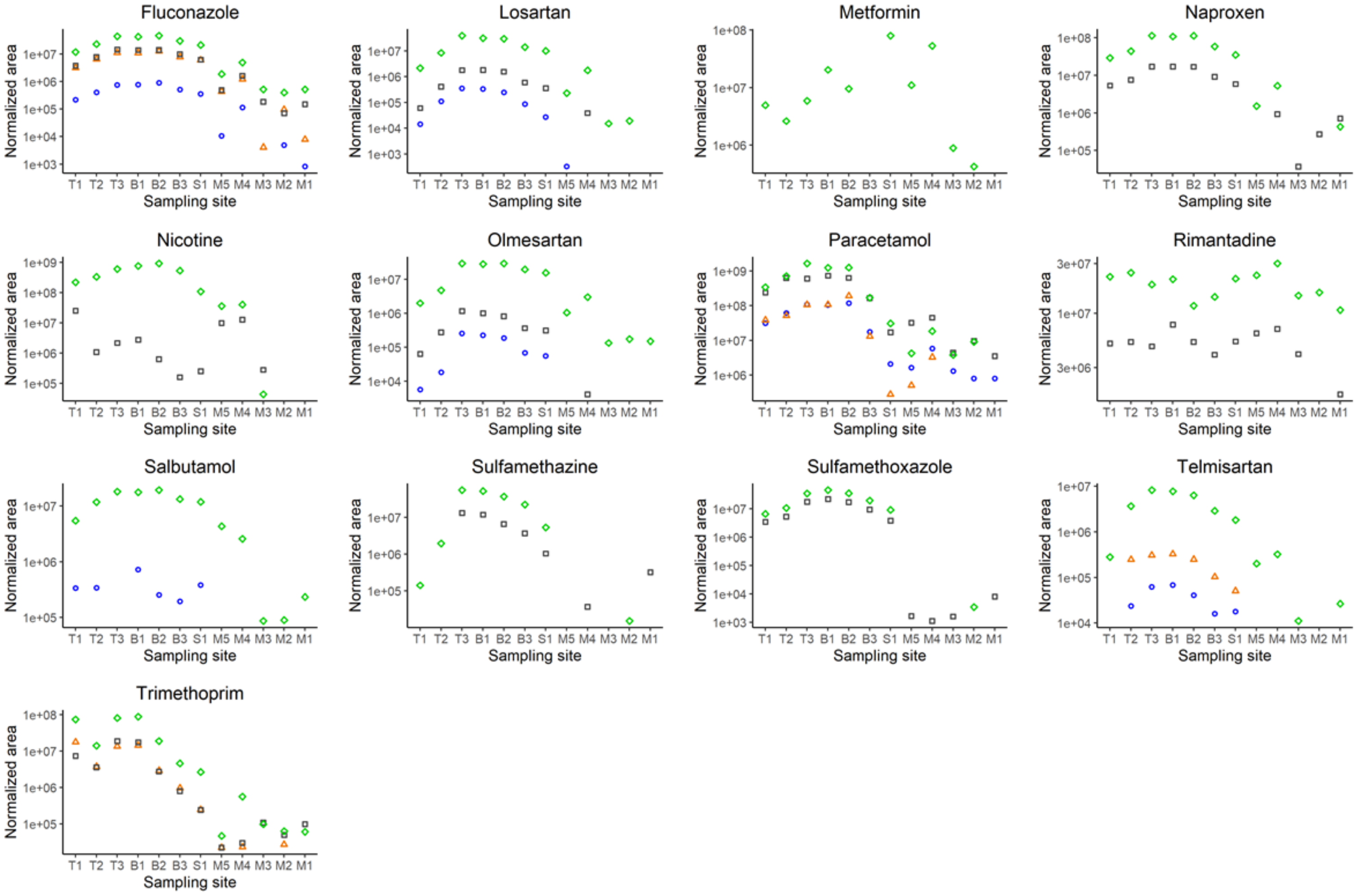

Figure S54. Continued

Industrial compounds

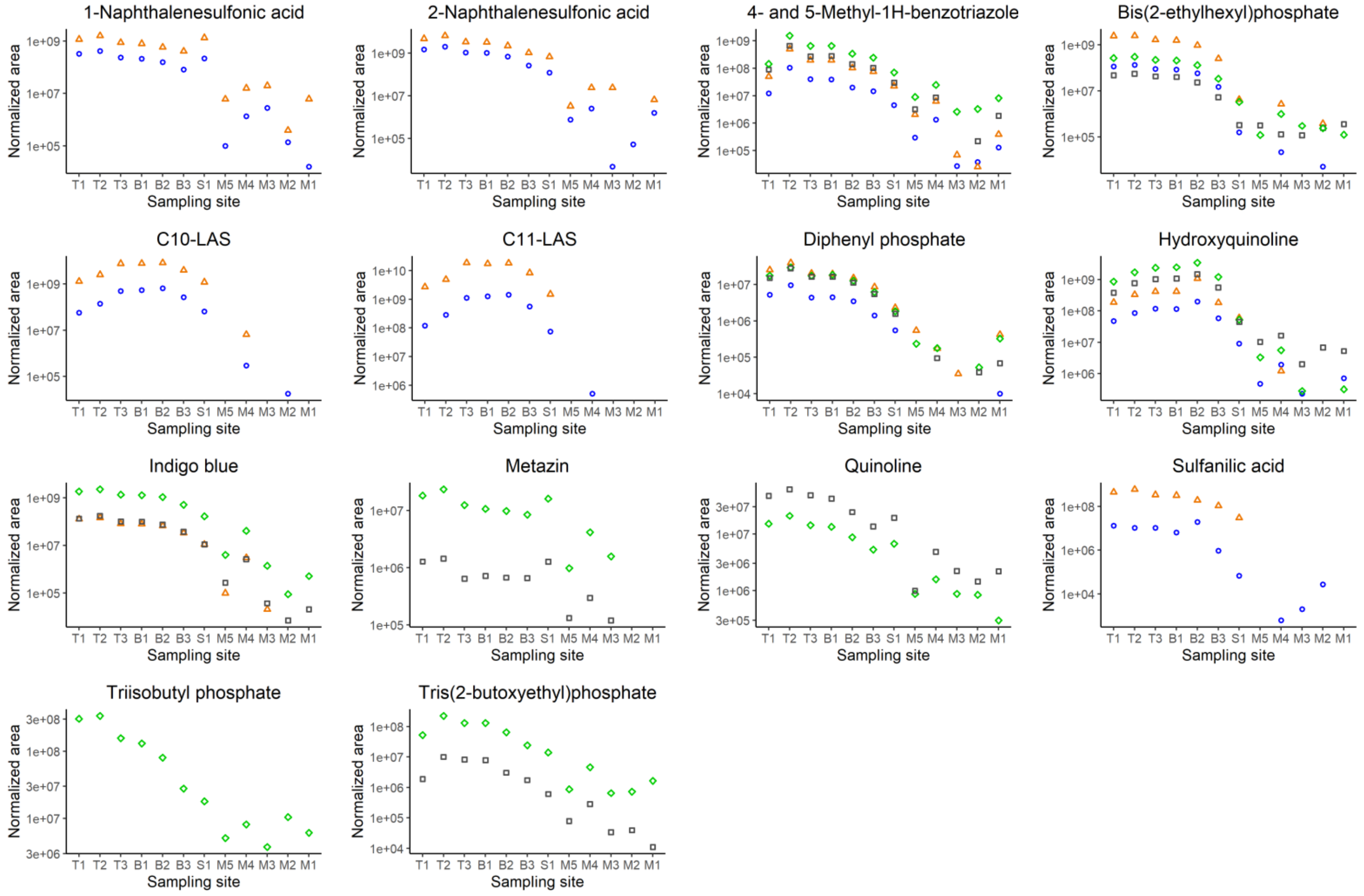

Figure S54. Continued

Pesticides

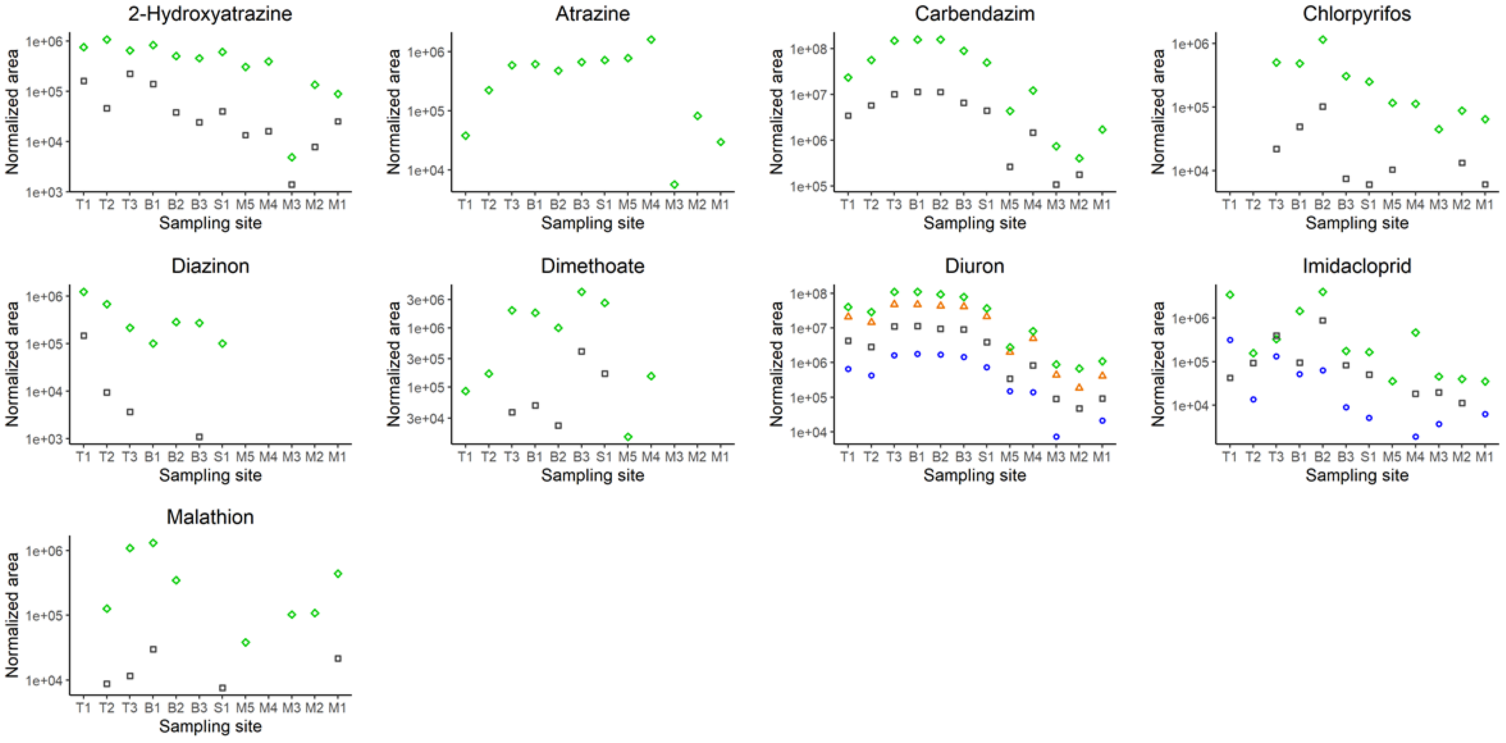

Figure S54. Continued

Miscellaneous

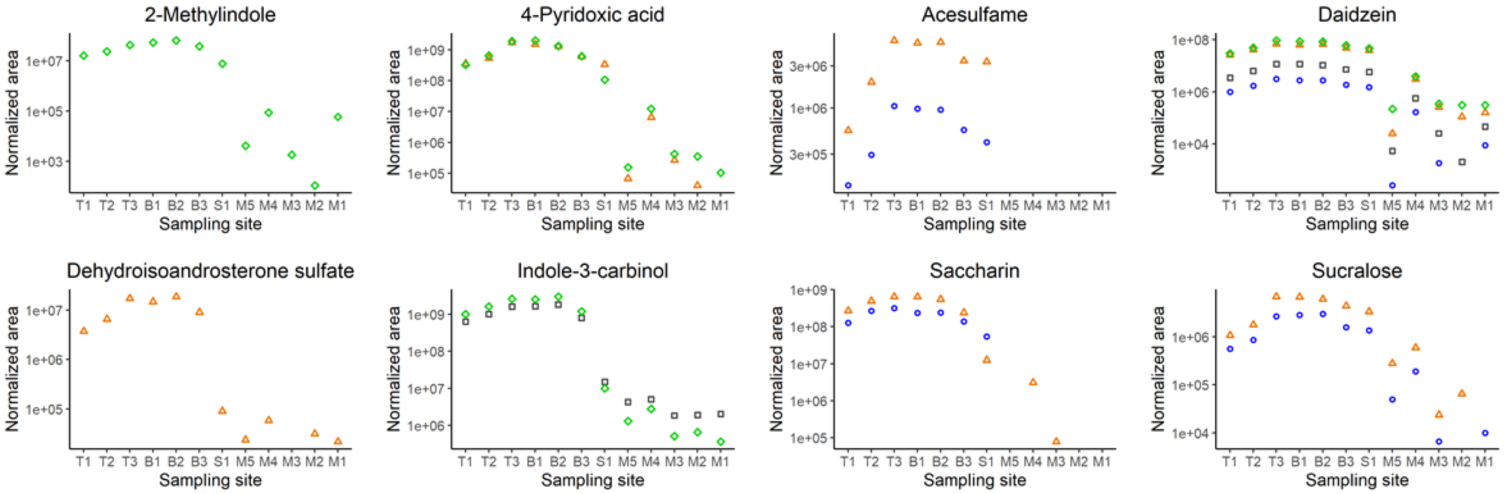

Personal care products

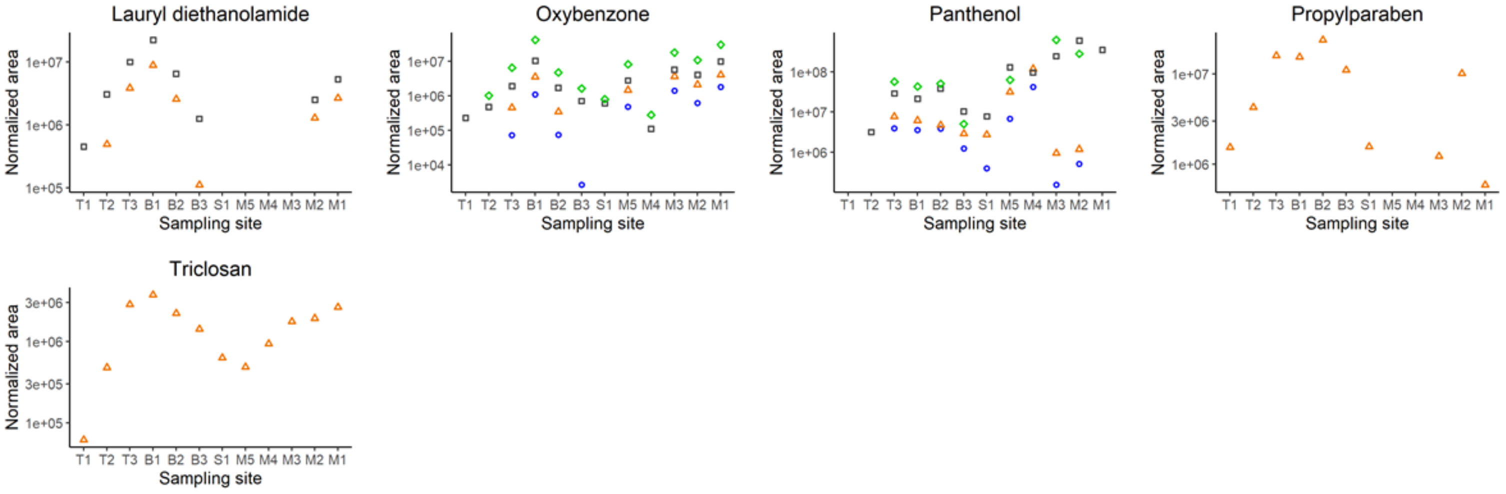

Figure S54. Continued

SI-4.6. Retention time index model

SI-4.6.1. Calibration curves obtained for samples injections

Positive ionization mode

The RTI mix developed for the ESI+ ionization mode was analysed in both ESI+ and APCI+. The calibrants Rt showed a relative standard deviation (RSD) lower than 0.6 % between the two batch of analysis, therefore the data were combined to build the positive ionization calibration curve that will be applied to both ESI+ and APCI+. The calibration mode chosen was "auto-calibrate", none of the calibrants were discarded among those detected (Table S47). The calibration curve equation is the following one:

RTI = 52.6382 * (tR) - 216.589 (R^2= 0.962)

RTI is the calculated retention time index and tR is the observed retention time.

The calibration curve is plotted in Figure S53.

Table S47. List of calibrants observed and included to build the RTI calibration curve in positive ionization mode

Calibrants	Rt	Status
Guanylurea		Not observed
Amitrole		Not observed
Histamine		Not observed
Chlormequate	3.28	Accepted
Methamidophos	4.7	Accepted
Vancomycin	8.27	Accepted
Cefoperazone	9.49	Accepted
Trichlorfon	9.84	Accepted
Butocarboxim	11.42	Accepted
Dichlorvos	12.73	Accepted
Tylosin	16.2	Accepted
TCMTB	15.56	Accepted
Rifaximin	16.8	Accepted
Spinosad A	20.02	Accepted
Emamectin	20.05	Accepted
Avermectin	20.7	Accepted
Nigericin	21.49	Accepted
Ivermectin	21.54	Accepted

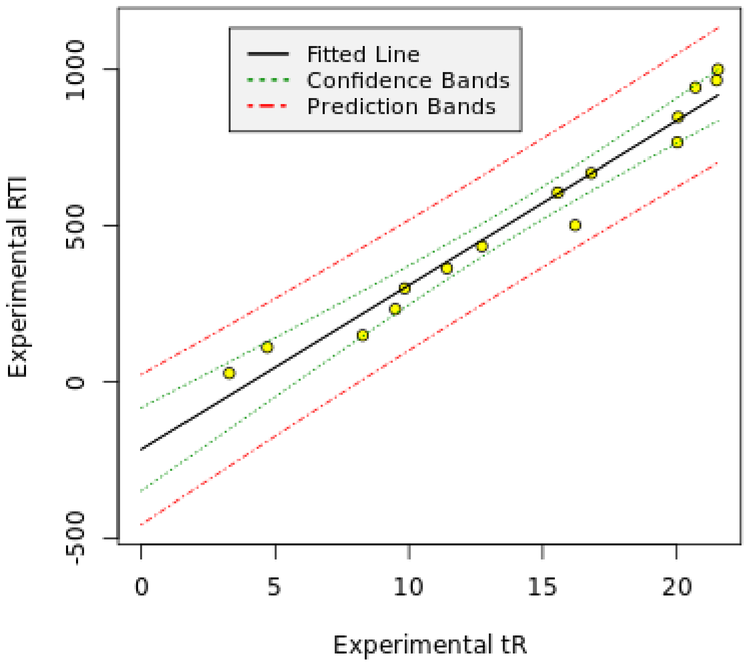

Figure S55. Retention time index versus retention time of the calibrants in positive ionization mode

Negative ionization mode

The RTI mix developed for the ESI- ionization mode was analysed in both ESI- and APCI-. The calibrants Rt showed a relative standard deviation (RSD) lower than 0.5 % between the two batch of analysis, therefore the data were combined to build the positive ionization calibration curve that will be applied to both ESI- and APCI-. The calibration mode chosen was "auto-calibrate", none of the calibrants were discarded among those detected (Table S48). The calibration curve equation is the following one:

RTI = 57.1468 * (tR) - 296.4467 (R^2= 0.96)

RTI is the calculated retention time index and tR is the observed retention time.

The calibration curve is plotted in Figure S54.

Table S48. List of calibrants observed and included to build the RTI calibration curve in negative ionization mode

Calibrants	tR	Status
Amitrole		Not observed
Benzoic acid		Not observed
Acephate	6.51	Accepted
Salicylic acid	6.17	Accepted
Simazine-2-Hydroxy	9.67	Accepted
Tepraloxydim	10.87	Accepted
Bromoxynil	9.38	Accepted
MCPA	11.56	Accepted
Valproic acid	13.74	Accepted
Phenytoin	13.51	Accepted
Flamprop	13.97	Accepted
Benodanil	14.28	Accepted
Dinoterb	14.54	Accepted
Inabenfide	15.87	Accepted
Coumaphos	17.72	Accepted
Triclosan	18.8	Accepted
Abamectin	20.69	Accepted
Salinomycin	20.81	Accepted

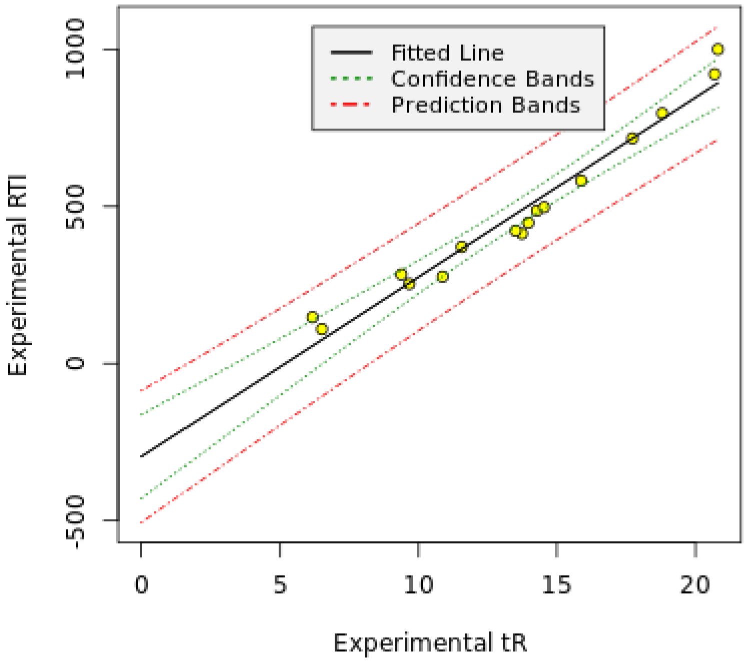

Figure S56. Retention time index versus retention time of the calibrants in negative ionization mode

SI-4.6.2. Comparison between exprimental and predicted RTI

For increased confidence in Level 2a annotations, experimental and predicted RTI were compared for Level 1 and Level 2a compounds. In most cases, observed and expected RTIs were in agreement, with uncertainty falling in box 1 ("candidate accepted") or box 2 ("although there is an error, the candidate is accepted"), meaning that the annotations can be trusted. A few compounds, mostly in negative mode, had higher uncertainty (3 and 4, "not reliable" and "not reliable, it could be a false positive", respectively).

Some compounds, such as paracetamol (Level 1) had the same Rt retention time in both positive (1) and negative (3) ionization modes. Something similar is observed for losartan and olmesartan. We therefore decided to consider these annotations as reliable, and considered we were facing problems with our model in negative ionization mode. An explanation could be related to the RTI model development itself. Except the 3 compounds cited earlier, all the compounds showing high uncertainty between experimental and predicted RTI contained a sulfur atom, and where mostly only detected in negative ionization mode. But the model that was developed for RTI prediction did include compounds containing sulfur in their structure in both test and training sets, for both ionization modes.[26] However, the authors reported that the model uncertainty is higher in negative ionization mode, partially due to the lack of optimal LC settings for this ionization mode.[26] This could explain the poor RTI uncertainty score of our 6 compounds showing box 3 and 4 uncertainties. In addition, the model was developed using 5 mM ammonium acetate as a modifier in both mobile phases, while we used 1 mM ammonium fluoride, only in water for negative ionization.[26] Therefore, we decided to not discard the annotations with higher RTI uncertainty, considering the comparison of their observed and predicted RTIs.

SI-4.6.3. Calibration curves obtained for annotations confirmation

Positive ionization mode

The RTI mix was along with the reinjection of samples and injection of standards to confirm the annotation. As for the first injections, a calibration curve was built choosing "auto-calibrate" calibration mode. None of the calibrants detected were discarded (Table S49). The calibration curve equation is the following one:

RTI = 52.5603 * (tR) -218.6757 (R^2= 0.961)

RTI is the calculated retention time index and tR is the observed retention time.

The new calibration curve is plotted in Figure S55.

Table S49. List of calibrants observed and included to build the RTI calibration curve in positive ionization mode with injections performed to confirm the annotations

Calibrants	tR	Status
Guanylurea	NA	Not observed
Amitrole	NA	Not observed
Histamine	NA	Not observed
Chlormequate	3.29	Accepted
Methamidophos	4.74	Accepted
Vancomycin	8.35	Accepted
Cefoperazone	9.55	Accepted
Trichlorfon	9.89	Accepted
Butocarboxim	11.47	Accepted
Dichlorvos	12.81	Accepted
Tylosin	16.30	Accepted
TCMTB	15.67	Accepted
Rifaximin	16.90	Accepted
Spinosad_A	20.11	Accepted
Emamectin	20.14	Accepted
Avermectin	20.73	Accepted
Nigericin	21.50	Accepted
Ivermectin	21.55	Accepted

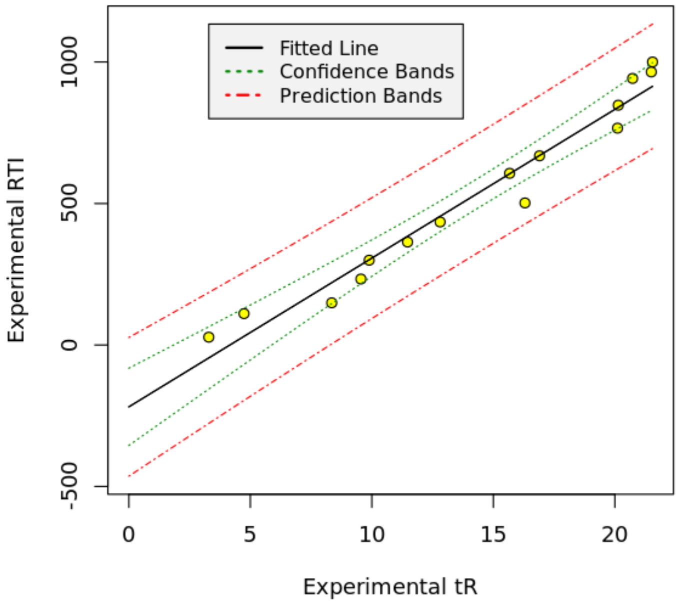

Figure S57. Retention time index versus retention time of the calibrants in positive ionization mode for injections performed along those to confirm the annotations

Negative ionization mode

The RTI mix was along with the reinjection of samples and injection of standards to confirm the annotation. As for the first injections, a calibration curve was built choosing "auto-calibrate" calibration mode. None of the calibrants detected were discarded (Table S50). The calibration curve equation is the following one:

RTI = 57.4167 * (tR) - 304.324 (R^2= 0.96)

RTI is the calculated retention time index and tR is the observed retention time.

The new calibration curve is plotted in Figure S56.

Table S50. List of calibrants observed and included to build the RTI calibration curve in positive ionization mode with injections performed to confirm the annotations

Calibrants	tR	Status
Amitrole	NA	Not observed
Benzoic acid	NA	Not observed
Acephate	6.52	Accepted
Salicylic acid	6.43	Accepted
Simazine-2-Hydroxy	9.72	Accepted
Tepraloxydim	10.97	Accepted
Bromoxynil	9.40	Accepted
MCPA	11.65	Accepted
Valproic acid	13.74	Accepted
Phenytoin	13.61	Accepted
Flamprop	14.05	Accepted
Benodanil	14.38	Accepted
Dinoterb	14.60	Accepted
Inabenfide	15.98	Accepted
Coumaphos	17.80	Accepted
Triclosan	18.85	Accepted
Abamectin	20.73	Accepted
Salinomycin	20.83	Accepted

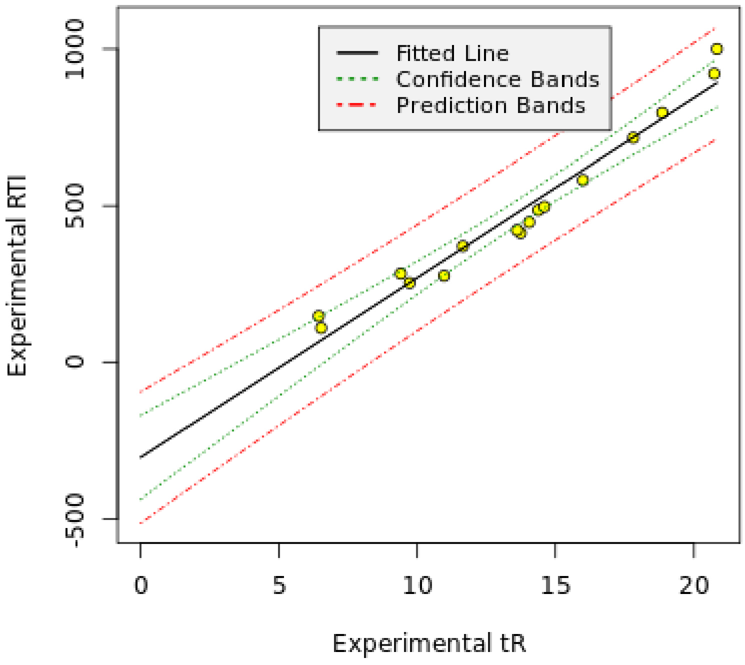

Figure S58. Retention time index versus retention time of the calibrants in negative ionization mode for injections performed along those to confirm the annotations

Due to retention time shift between the time the samples were injected and the time the confirmation of the annotations was performed, the RTIs of the annotated features were compared to the RTIs of the chromatographic peaks formally identified. The RTI variations between the two injection times are lower than │20│, except for erythromycin. However, no other proper chromatographic peak corresponding to erythromycin m/z was detected in the .raw data during annotation confirmation.

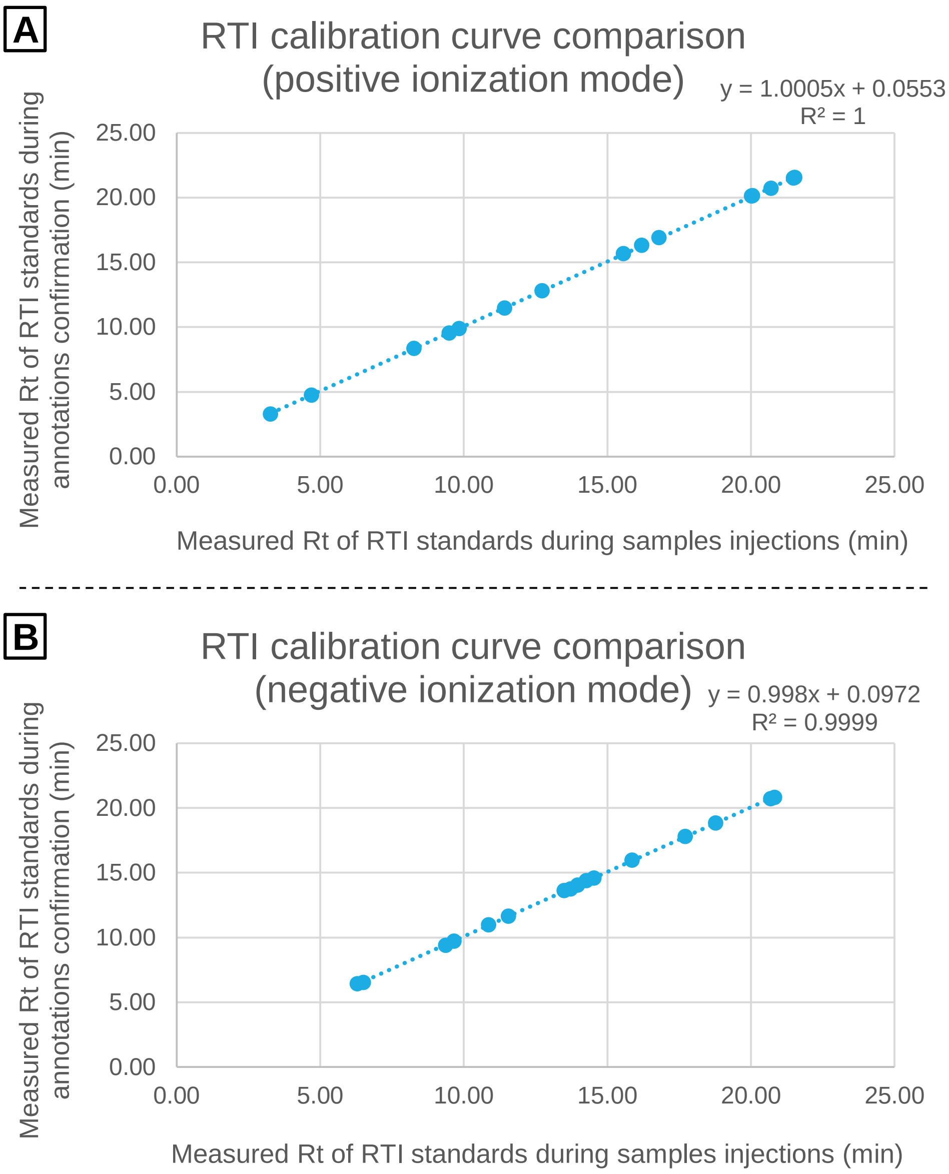

Figure S59. Linear fir between the retention times (Rt) of the RTI mixture of standards injected during samples analysis and annotations confirmations in A) positive and B) negative ionization modes.

SI-4.6.4. Comparison between the RTIs between non-spiked and spiked samples for Level 1 confirmations

Table S51. Retention time index (RTI) comparison between the non-spiked and spiked samples injections for Level 1 confirmations

Name	Ionization mode	RTI in non-spiked sample	RTI in spiked sample	Δ RTI
1-Naphthalenesulfonic acid	ESI-	191.18	192.33	-1.15
2-Hydroxyatrazine	ESI+	392.60	393.65	-1.05
2-Naphthalenesulfonic acid	ESI-	226.78	228.50	-1.72
4-Methyl-1H-benzotriazole	ESI+	358.44	358.44	0.00
5-Methyl-1H-benzotriazole	ESI+	356.33	358.44	-2.10
Acesulfame	ESI-	-81.55	-79.25	-2.30
Aspirin	ESI+	367.37	367.90	-0.53
Atenolol	ESI+	177.63	176.58	1.05
Atrazine	ESI+	556.06	549.76	6.31
Bis(2-ethylhexyl) phosphate	ESI+	789.43	789.43	0.00
Caffeine	ESI+	235.45	235.97	-0.53
Carbamazepine	ESI+	518.75	520.32	-1.58
Carbendazim	ESI+	346.35	346.35	0.00
Chlorpyrifos	ESI+	814.66	813.61	1.05
Clarithromycin	ESI+	649.09	648.57	0.53
Cotinine	ESI+	214.95	214.42	0.53
Daidzein	ESI+	460.93	460.93	0.00
Diazinon	ESI+	719.00	718.47	0.53
Diclofenac	ESI+	591.80	592.86	-1.05
Dimethoate	ESI+	309.03	309.03	0.00
Diphenyl phosphate	ESI+	411.52	412.05	-0.53
Diuron	ESI+	560.79	561.84	-1.05
Erythromycin	ESI+	625.97	622.29	3.68
Fluconazole	ESI+	336.89	336.36	0.53
Imidacloprid	ESI+	320.07	296.94	23.13
Losartan	ESI+	559.22	560.27	-1.05
Malathion	ESI+	651.72	651.20	0.53
Metformin	ESI+	-51.01	-52.59	1.58
Nicotine	ESI+	216.52	217.05	-0.53
Oxybenzone	ESI+	684.84	685.36	-0.53
Panthenol	ESI-	91.85	91.85	0.00
Paracetamol	ESI+	118.24	113.51	4.73
Propylparaben	ESI-	540.85	540.28	0.57
Quinoline	ESI+	422.03	420.98	1.05
Salbutamol	ESI+	172.37	170.80	1.58
Sucralose	ESI-	221.61	221.61	0.00
Sulfamethazine	ESI+	239.65	240.18	-0.53
Sulfamethoxazole	ESI+	93.01	89.33	3.68
Triclosan	ESI-	779.13	779.13	0.00

Name	Ionization mode	RTI in non-spiked sample	RTI in spiked sample	Δ RTI
Trimethoprim	ESI+	305.88	306.93	-1.05
Tris(2-butoxyethyl) phosphate	ESI+	783.12	783.65	-0.53

SI-4.7. Further investigation of detected compounds

SI-4.7.1. Correlation between linear alkyl benzene sulfonate and pesticides

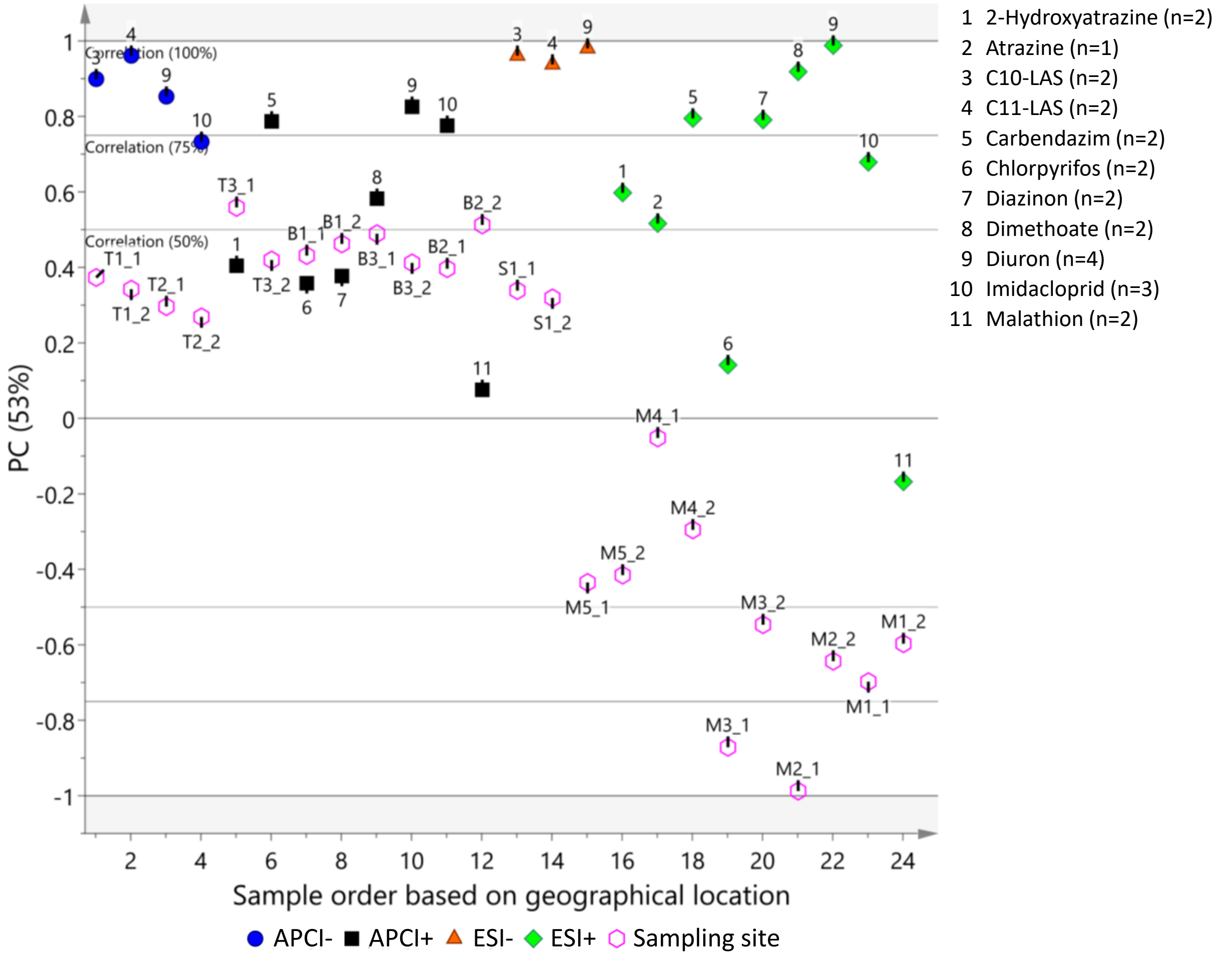

Figure S60. Correlation between C10-LAS and C11-LAS with the pesticides detected in the samples highlighted in a PCA biplot (one component)

SI-4.7.2. Chemical formula prediction

Among the 100 molecular features selected based by top VIP scores in the OPLS-DA model, 30 of these had a chemical formula confidently predicted. For these 30 features, their in silico predicted chemical formula's and structure's (Level 3) are reported in Table S52.

Table S52. Predicted chemical formula of 100 molecular features with highest VIP scores in the OPLS-DA model

Ionization	Measured m/z (Da)	Rt (min)	VIP score	p_{corr}	Predicted chemical formula [M]	Most probable structure (chemical name)	Most probable structure score (/10)	Class and/or subclass	PubChem CID
APCI-	349.1120	11.72	1.351	0.997	C18H22O5S	Estrone sulfate	5.13	Pharmaceutical metabolite	3001028
APCI-	145.0870	6.94	1.351	0.997	C7H14O3	Butyl lactate	4.72	Industrial compound	8738
APCI-	175.1340	11.60	1.351	0.997	C9H20O3	3-(hexyloxy)propane-1,2-diol	3.7	Industrial compound	11229094
APCI-	175.1340	11.40	1.351	0.997	C9H20O3	1-(1-methyl-2-propoxyethoxy)propan-2-ol	3.83	Industrial compound	121752
APCI-	213.1500	12.30	1.350	0.996	C12H22O3	3-Oxododecanoic acid	5.12	Endogenous metabolite	439717
APCI-	319.1910	13.01	1.350	0.996	C19H28O4	5'-Carboxy-alpha-chromanol	5.25	Plant metabolite	53481524
APCI-	199.0080	5.73	1.350	0.996	C8H8O4S	4-Vinylphenol sulfate	4.3	Human xenobiotic metabolite	6426766
APCI-	181.9920	5.41	1.350	0.996	C7H5NO3S	Saccharin	4.87	Sweetener	5143
APCI-	108.0220	7.34	1.350	0.996	C3H8ClNO	#			
APCI-	311.1690	18.98	1.349	0.996	C17H28O3S	N-Undecylbenzenesulfonic acid *	4.46	Industrial compound (surfactant)	38222
ESI-	195.0490	12.79	1.351	0.997	C10H12O2S	3-Methyl-4,5,6,7-tetrahydro-1-benzothiophene-2-carboxylic acid	4.06	Investigated for its potential as antimicrobial[27]	22011966
ESI-	165.0920	11.96	1.351	0.997	C10H14O2	Perillic acid	4.93	Endogenous metabolite	1256
ESI-	311.1690	18.84	1.351	0.997	C17H28O3S	N-Undecylbenzenesulfonic acid *	3.93	Industrial compound (surfactant)	38222
ESI-	142.0670	7.41	1.351	0.997	C10H9N	2-Methylquinoline	4.45	Industrial compound (various use)	7060
ESI-	183.0120	18.82	1.350	0.997	C8H8O3S	4-Ethenylbenzenesulfonic Acid	4.54	Pharmaceutical and personal care product	75905

Ionization	Measured m/z (Da)	Rt (min)	VIP score	p_{corr}	Predicted chemical formula [M]	Most probable structure (chemical name)	Most probable structure score (/10)	Class and/or subclass	PubChem CID
ESI-	241.0540	10.85	1.350	0.996	C11H14O4S	Penicillone	4.32	Natural product	23651016
ESI-	154.9670	3.09	1.350	-0.997	C4H6Cl2O2	Methyl 2,2-dichloropropionate	2.47	Herbicide (dalapon metabolite)	87207
ESI-	369.1280	18.83	1.350	0.997	C20H22N2O3S	2-(1-Benzylpyrrolidin-3-yl)-5-ethylsulfonyl-1,3-benzoxazole	4.16		75615126
ESI-	222.0490	10.47	1.350	0.997	C10H7F2N3O	1-(2,4-Difluorophenyl)-2-(1H-1,2,4-triazol-1-yl)ethanone	3.86	Fluconazole related compound	588080
ESI-	158.0610	10.35	1.350	0.996	C10H9NO	Indoleacetaldehyde	5.38	Endogenous metabolite	800
ESI-	243.0810	5.44	1.350	0.996	C10H16N2O3S	Biotin	5.55	Endogenous metabolite	171548
ESI-	351.2180	12.54	1.350	0.996	C20H32O5	Prostaglandin E2	5.54	Endogenous metabolite	5280360
ESI-	353.2160	19.60	1.350	0.996	C20H34O3S	4-tetradecylbenzenesulfonic acid	4.01	Industrial compound	34219
ESI-	260.1870	7.39	1.350	0.996	C13H27NO4	N-(1,3,5-trihydroxynonan-2-yl)butanamide	4.24		163108075
ESI-	317.2120	16.05	1.350	0.996	C20H30O3	12-Hydroxyeicosapentaenoic Acid	5.24	Endogenous metabolite	10041593
ESI-	245.1400	10.80	1.349	0.996	C12H22O5	3-Hydroxydodecanedioic acid	4.26	Natural product, plant metabolite	16663321
ESI-	161.0720	12.32	1.349	0.996	C9H10N2O	Norcotinine	3.9	Nicotine metabolite	413
ESI+	295.1660	4.65	1.351	0.997	C15H22N2O4	Tyrosyl-Leucine	4.72	Endogenous metabolite	87071
ESI+	377.1280	13.30	1.350	0.996	C17H20N4O4S	4-Methyl-8-(1-methylpyrazol-4-yl)-2-methylsulfonyl-1,3,3a,10a-tetrahydropyrrolo[3,4-b][1,4]benzoxazepin-5-one	4.58		162901485
ESI+	183.1130	5.79	1.350	0.996	C9H14N2O2	N-Formylloline	4.44	Natural product	14313946

No structure was predicted, but the presence of chlorine in the structure is confirmed by the parent isotopic pattern

* These features were annotated as C11-LAS (Level 2a) using MS2 comparison to public databases. The compounds are isomers and both are surfactants.

SI-4.7.3. Semi-quantification results

Table S53. Detection frequency and semi-quantified concentrations (minimum, maximum, average) of Level 1 confirmed substances in surface water samples.

Chemical	Detection frequency	Minimum (ng/L)	Maximum (ng/L)	Average (ng/L)
1-Naphthalenesulfonic acid	100.0%	2.3	9300	3300
2-Hydroxyatrazine	100.0%	0.01	1.4	0.6
2-Naphthalenesulfonic acid	91.7%	15	29000	8900
4- and 5-Methyl-1H-benzotriazole	100.0%	3.2	1900	380
Acesulfame	58.3%	4.7	49	31
Aspirin	100.0%	37	290	99
Atenolol	100.0%	0.01	80	29
Atrazine	100.0%	0.06	17	5.2
Bis(2-ethylhexyl)phosphate	75.0%	1.8	12000	5000
Caffeine	66.7%	1.9	540	200
Carbamazepine	100.0%	0.02	15	5.0
Carbendazim	100.0%	0.1	31	12
Chlorpyrifos	83.3%	1.2	30	8.2
Clarithromycin	50.0%	0.5	24	13
Cotinine	100.0%	12	3700	1400
Daidzein	100.0%	0.7	295	120
Diazinon	58.3%	5.4	66	22
Diclofenac	100.0%	0.05	70	28
Dimethoate	75.0%	0.01	3.6	1.2
Diphenyl phosphate	91.7%	0.5	510	160
Diuron	100.0%	0.6	100	40
Erythromycin	50.0%	0.7	18	8.9
Fluconazole	100.0%	0.6	68	28
Imidacloprid	100.0%	2.6	300	65
Losartan	91.7%	0.2	540	170
Malathion	66.7%	0.2	8.5	2.9
Metformin	83.3%	4.6	870	200
Nicotine	83.3%	0.2	3700	1400
Oxybenzone	91.7%	3.0	450	120
Panthenol	75.0%	80	10000	1600
Paracetamol	91.7%	4.5	2000	590
Propylparaben	83.3%	3.3	130	48
Quinoline	100.0%	0.5	35	12
Salbutamol	100.0%	0.2	47	21
Sucralose	91.7%	2.0	580	240
Sulfamethazine	66.7%	0.01	32	13
Sulfamethoxazole	58.3%	2.2	15	7.7
Triclosan	100.0%	0.6	36	15
Trimethoprim	66.7%	0.02	2.8	1.1
Tris(2-butoxyethyl)phosphate	100.0%	1.3	435	110

References

(1) International Steering Committee for Global Mapping; Sārve aba Bāṃlādeśa. Rivers, Bangladesh, 2016, 2016. http://purl.stanford.edu/pq592pj5250.

(2) International Steering Committee for Global Mapping; Sārve aba Bāṃlādeśa. Inland Waters, Bangladesh, 2016, 2016. http://purl.stanford.edu/js773kw6163.

(3) *Free Spatial Data | DIVA-GIS*. https://www.diva-gis.org/Data (accessed 2021-11-17).

(4) *OpenStreetMap*. © OpenStreetMap contributors, CC BY-SA. https://www.openstreetmap.org/ (accessed 2021-11-17).

(5) Tsugawa, H.; Kind, T.; Nakabayashi, R.; Yukihira, D.; Tanaka, W.; Cajka, T.; Saito, K.; Fiehn, O.; Arita, M. Hydrogen Rearrangement Rules: Computational MS/MS Fragmentation and Structure Elucidation Using MS-FINDER Software. *Anal. Chem.* **2016**, *88* (16), 7946–7958. https://doi.org/10.1021/acs.analchem.6b00770.

(6) Dührkop, K.; Fleischauer, M.; Ludwig, M.; Aksenov, A. A.; Melnik, A. V.; Meusel, M.; Dorrestein, P. C.; Rousu, J.; Böcker, S. SIRIUS 4: A Rapid Tool for Turning Tandem Mass Spectra into Metabolite Structure Information. *Nat. Methods* **2019**, *16* (4), 299–302. https://doi.org/10.1038/s41592-019-0344-8.

(7) Alonso, M. C.; Barceló, D. Tracing Polar Benzene- and Naphthalenesulfonates in Untreated Industrial Effluents and Water Treatment Works by Ion-Pair Chromatography-Fluorescence and Electrospray-Mass Spectrometry. *Anal. Chim. Acta* **1999**, *400* (1), 211–231. https://doi.org/10.1016/S0003-2670(99)00705-9.

(8) Wode, F.; van Baar, P.; Dünnbier, U.; Hecht, F.; Taute, T.; Jekel, M.; Reemtsma, T. Search for over 2000 Current and Legacy Micropollutants on a Wastewater Infiltration Site with a UPLC-High Resolution MS Target Screening Method. *Water Res.* **2015**, *69*, 274–283. https://doi.org/10.1016/j.watres.2014.11.034.

(9) Montes, R.; Aguirre, J.; Vidal, X.; Rodil, R.; Cela, R.; Quintana, J. B. Screening for Polar Chemicals in Water by Trifunctional Mixed-Mode Liquid Chromatography–High Resolution Mass Spectrometry. *Environ. Sci. Technol.* **2017**, *51* (11), 6250–6259. https://doi.org/10.1021/acs.est.6b05135.

(10) Hinnenkamp, V.; Balsaa, P.; Schmidt, T. C. Quantitative Screening and Prioritization Based on UPLC-IM-Q-TOF-MS as an Alternative Water Sample Monitoring Strategy. *Anal. Bioanal. Chem.* **2019**. https://doi.org/10.1007/s00216-019-01994-w.

(11) Niessen, W. M. A.; Correa C., R. A. *Interpretation of MS-MS Mass Spectra of Drugs and Pesticides*; John Wiley & Sons, Inc.: Hoboken, New Jersey, 2017. https://doi.org/10.1002/9781119294269.

(12) Pellegrini, M.; Marchei, E.; Rossi, S.; Vagnarelli, F.; Durgbanshi, A.; García-Algar, Ó.; Vall, O.; Pichini, S. Liquid Chromatography/Electrospray Ionization Tandem Mass Spectrometry Assay for Determination of Nicotine and Metabolites, Caffeine and Arecoline in Breast Milk. *Rapid Commun. Mass Spectrom.* **2007**, *21* (16), 2693–2703. https://doi.org/10.1002/rcm.3137.

(13) Kern, S.; Fenner, K.; Singer, H. P.; Schwarzenbach, R. P.; Hollender, J. Identification of Transformation Products of Organic Contaminants in Natural Waters by Computer-Aided Prediction and High-Resolution Mass Spectrometry. *Environ. Sci. Technol.* **2009**, *43* (18), 7039–7046. https://doi.org/10.1021/es901979h.

(14) Madeira, P. J. A.; Borges, C. M.; Florêncio, M. H. Electrospray Ionization Fourier Transform Ion Cyclotron Resonance Mass Spectrometric and Semi-Empirical Calculations Study of Five Isoflavone Aglycones. *Rapid Commun. Mass Spectrom.* **2010**, *24* (23), 3432–3440. https://doi.org/10.1002/rcm.4791.

(15) Wang, F.; Li, S.; Feng, H.; Yang, Y.; Xiao, B.; Chen, D. An Enhanced Sensitivity and Cleanup Strategy for the Nontargeted Screening and Targeted Determination of Pesticides in Tea Using Modified Dispersive Solid-Phase Extraction and Cold-Induced Acetonitrile Aqueous Two-Phase Systems Coupled with Liquid Chromatography-High Resolution Mass Spectrometry. *Food Chem.* **2019**, *275*, 530–538. https://doi.org/10.1016/j.foodchem.2018.09.142.

(16) Massei, R.; Byers, H.; Beckers, L.-M.; Prothmann, J.; Brack, W.; Schulze, T.; Krauss, M. A Sediment Extraction and Cleanup Method for Wide-Scope Multitarget Screening by Liquid Chromatography–High-Resolution Mass Spectrometry. *Anal. Bioanal. Chem.* **2018**, *410* (1), 177–188. https://doi.org/10.1007/s00216-017-0708-9.

(17) Stravs, M. A.; Mechelke, J.; Ferguson, P. L.; Singer, H.; Hollender, J. Microvolume Trace Environmental Analysis Using Peak-Focusing Online Solid-Phase Extraction–Nano-Liquid Chromatography–High-Resolution Mass Spectrometry. *Anal. Bioanal. Chem.* **2016**, *408* (7), 1879–1890. https://doi.org/10.1007/s00216-015-9294-x.

(18) Thurman, E. M.; Ferrer, I. Identification of Unknown Pesticides in Food Using Both LC/MSD TOF and Ion Trap MSn, 2005. https://www.agilent.com/cs/library/applications/5989-1924EN_low.pdf.
(19) Lacina, O.; Urbanova, J.; Poustka, J.; Hajslova, J. Identification/Quantification of Multiple Pesticide Residues in Food Plants by Ultra-High-Performance Liquid Chromatography-Time-of-Flight Mass Spectrometry. *J. Chromatogr. A* **2010**, *1217* (5), 648–659. https://doi.org/10.1016/j.chroma.2009.11.098.
(20) Thurman, E. M.; Ferrer, I.; Pozo, O. J.; Sancho, J. V.; Hernandez, F. The Even-Electron Rule in Electrospray Mass Spectra of Pesticides. *Rapid Commun. Mass Spectrom.* **2007**, *21* (23), 3855–3868. https://doi.org/10.1002/rcm.3271.
(21) Ziarrusta, H.; Mijangos, L.; Montes, R.; Rodil, R.; Anakabe, E.; Izagirre, U.; Prieto, A.; Etxebarria, N.; Olivares, M.; Zuloaga, O. Study of Bioconcentration of Oxybenzone in Gilt-Head Bream and Characterization of Its by-Products. *Chemosphere* **2018**, *208*, 399–407. https://doi.org/10.1016/j.chemosphere.2018.05.154.
(22) Kuki, Á.; Zelei, G.; Nagy, L.; Nagy, T.; Zsuga, M.; Kéki, S. Rapid Mapping of Various Chemicals in Personal Care and Healthcare Products by Direct Analysis in Real Time Mass Spectrometry. *Talanta* **2019**, *192*, 241–247. https://doi.org/10.1016/j.talanta.2018.09.054.
(23) Díaz, R.; Ibáñez, M.; Sancho, J. V.; Hernández, F. Qualitative Validation of a Liquid Chromatography–Quadrupole-Time of Flight Mass Spectrometry Screening Method for Organic Pollutants in Waters. *J. Chromatogr. A* **2013**, *1276*, 47–57. https://doi.org/10.1016/j.chroma.2012.12.030.
(24) Lee, H. M.; Yang, J.-S.; Lee, H.-W.; Hwang, I. M.; Hwang, Y. S.; You, S.-Y.; Ha, J.-H.; Kim, S. H. Simultaneous Determination of Preservatives, Artificial Sweeteners, and Synthetic Dyes in Kimchi by Ultra-Performance Liquid Chromatography Electrospray Ionization Tandem Mass Spectrometry (UPLC-ESI-MS/MS). *Anal. Lett.* **2019**, *52* (16), 2472–2483. https://doi.org/10.1080/00032719.2019.1612906.
(25) Gago-Ferrero, P.; Krettek, A.; Fischer, S.; Wiberg, K.; Ahrens, L. Suspect Screening and Regulatory Databases: A Powerful Combination To Identify Emerging Micropollutants. *Environ. Sci. Technol.* **2018**. https://doi.org/10.1021/acs.est.7b06598.
(26) Aalizadeh, R.; Alygizakis, N. A.; Schymanski, E. L.; Krauss, M.; Schulze, T.; Ibáñez, M.; McEachran, A. D.; Chao, A.; Williams, A. J.; Gago-Ferrero, P.; Covaci, A.; Moschet, C.; Young, T. M.; Hollender, J.; Slobodnik, J.; Thomaidis, N. S. Development and Application of Liquid Chromatographic Retention Time Indices in HRMS-Based Suspect and Nontarget Screening. *Anal. Chem.* **2021**. https://doi.org/10.1021/acs.analchem.1c02348.
(27) Jayaraman, S. R.; Sridharan, M.; Nagappan, R. 3-Methyl-4,5,6,7-Tetrahydro-1-Benzothiophene-2-Carboxylic Acid. *Molbank* **2010**, *2010* (1), M648. https://doi.org/10.3390/M648.
